# Lithium Enables Pd-Catalyzed 5*-endo-dig* Cyclization/Coupling of α-Homopropargyl-β-ketoesters with Aryl Bromides and Triflates

**DOI:** 10.1021/acs.orglett.4c02846

**Published:** 2024-09-19

**Authors:** Bartosz Bisek, Katarzyna Kochaniak, Wojciech Chaładaj

**Affiliations:** Institute of Organic Chemistry, Polish Academy of Sciences, Kasprzaka 44/52, 01-224 Warsaw, Poland

## Abstract

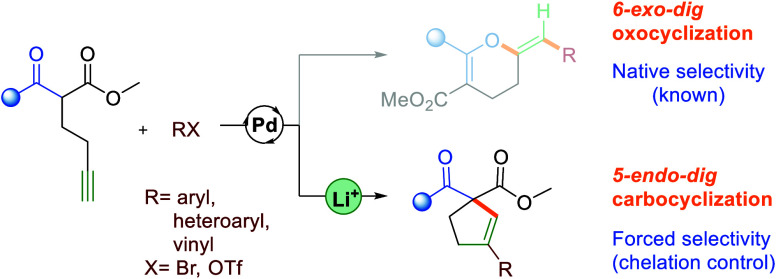

Tight chelation of enolate by lithium alters the selectivity of tandem palladium-catalyzed cyclization/coupling of terminal α-homopropargyl-β-ketoesters with aryl halides. In the presence of LiOH, substituted cyclopentenes are preferentially formed via 5-*endo-dig* carbocyclization, in contrast to the 6*-exo-dig* oxocyclization exclusively observed in the absence of a hard, chelating metal center. The disclosed transformation, featuring mild conditions and broad functional group tolerance, can be applied for a variety of (hetero)aryl bromides as well as aryl and vinyl triflates.

Intramolecular addition of the enolates of β-dicarbonyl compounds to alkynes constitutes a powerful tool for constructing carbo- and heterocyclic scaffolds of high utility.^1^ Numerous protocols have been developed for 5*-exo-dig* Conia-ene cyclization, taking advantage of catalytic activation of the alkyne or carbonyl functionalities.^2^ In contrast, 5*-endo-dig* cyclizations are considerably less common. This is notably attributed to the challenges posed by revised Baldwin’s rules, which dictate that additions of nucleophiles to unactivated alkynes along the 5-*endo*-*dig* path are inherently difficult due to stereoelectronic reasons (Figure 1a).^3^ However, the coordination of a carbophilic Lewis acid to the acetylenic moiety generates a LUMO with appropriate symmetry, thus enabling facile *endo* and *exo* attack of the nucleophile (“LUMO umpolung”). Efficient methodologies for 5*-endo-dig* cyclization of α-homopropargyl-β-ketoesters triggered by a gold^4^ or palladium^5^ catalyst as well as those utilizing a stoichiometric amount of iodine^6^ have been disclosed (Figure 1b). Approaches based on dual activation of alkyne and carbonyl have also proven to be successful in this context.^7,8^

**Figure 1:**
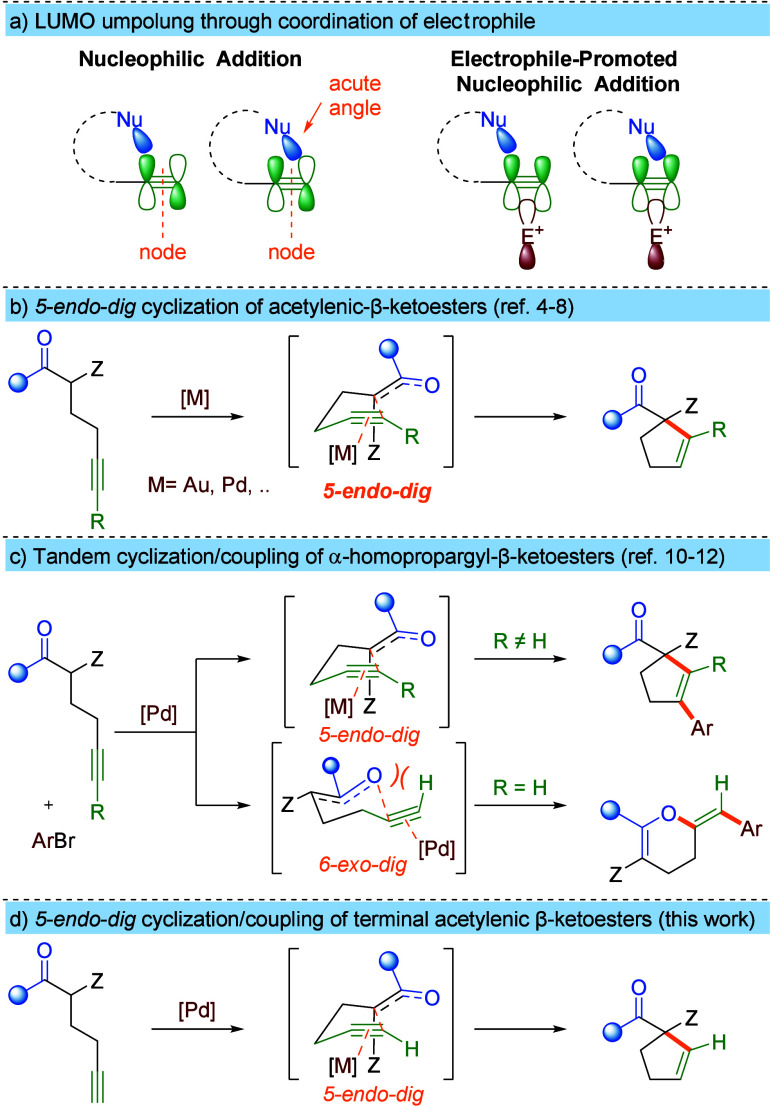
Cyclizations of α-homopropargyl-β-ketoesters.

For metal-catalyzed reactions, utilization of the vinyl–metal intermediate for further functionalization via cross-coupling renders a possibility for introducing additional molecular complexity, along with the excellent atom economy of Conia-ene cyclization.^9^ Elegant routes leading to substituted cyclopentenes^10^ and indenes^11^ were developed, leveraging palladium-catalyzed 5-*endo*-*dig* cyclization (Figure 1c). Noticeably, the reported scope covered only substrates bearing internal alkyne moieties. More recently, we unexpectedly discovered that a similar strategy applied for terminal α-homopropargyl-β-ketoesters cleanly delivers benzylidene-dihydropyrans through 6*-exo-dig* oxcyclization, rather than the expected cyclopentenes.^12^ Notably, analogous substrates bearing an internal alkyne moiety underwent 5*-endo-dig* carbocyclization under identical reaction conditions. Simple cyclization initiated by a cationic gold(I) complex results in the formation of cyclopentene, regardless of whether a terminal or internal alkyne is present.

We anticipated that the dichotomy in the regioselectivity of processes triggered by the coordination of a different soft metal center to alkyne could arise from whether the cyclization is carried out with or without a base. Employing a base is essential for Pd-catalyzed cyclizations accompanied by cross-coupling. Therefore, we hypothesized that the proper choice of a base or the addition of a hard Lewis acid for concomitant activation of the ketone moiety can alter the trajectory of the transformation toward 5-*endo-dig* carbocyclization leading to cyclopentenes.

A hypothesis concerning the different regioselectivity of cyclization involving an acetylenic ketoester (in keto or enol form) and its enolate in a model cyclization of methyl 2-acetylhex-5-ynoate triggered by coordination of the electrophile was investigated computationally at the M06/def2-TZVPP/SMD(DMF)//B3LYP-D3/def2-SVP level of theory.^13^^*i*^Pr-XPhosPd(Ph)Br, a product of the oxidative addition of bromobenzene to ^*i*^Pr-XPhosPd(0), was chosen as a benchmark carbophilic Lewis acid, where cyclohexyl groups at the P center of XPhos ligand were trimmed to isopropyls. The calculated activation barrier (Δ*G*^⧧^) for the 5*-endo-dig* carbocyclization of ketoester **1** (115.0 kJ/mol) is lower by 15.4 kJ/mol than that of 6*-exo-dig* oxocyclization (130.4 kJ/mol) (Table 1, entry 1). The relatively high activation barrier (Δ*G*^⧧^ > 110 kJ/mol) is consistent with the reported lack of reactivity in the absence of a base. In contrast, the deprotonated form of **1** prefers cyclization toward dihydropyran via the 6*-exo-dig* manifold with a considerably lower barrier (entry 2; Δ*G*^⧧^ = 49.3 kJ/mol). However, the investigation of a “naked” enolate omits potentially important interactions with an accompanying cation. Thus, transformations of potassium, sodium, and lithium enolates of **1** were also investigated (entries 3–5, respectively). The role of the counterion turned out to be crucial for the selectivity. Potassium enolate resembles naked enolate, for which 6*-exo-dig* oxocyclization is favored. Decreasing the ionic radius of the metal cation slightly increases the activation barriers and gradually changes the selectivity toward 5*-endo-dig* carbocyclization. Sodium enolates exhibit practically no selectivity (entry 4), while lithium switches the preference to 5*-endo-dig* carbocyclization and formation of cyclopentenes. It can be attributed to the small ionic radius of the lithium cation, enabling tighter interaction with the enolate and better chelation of the dicarbonyl system.^14^ This hypothesis is further supported by the equilibrium between enolate in which the β-dicarbonyl system is chelated by an alkali metal cation and its open form (*E-*enolate). The former can undergo carbocycliation, while oxocyclization requires a change in the configuration to *E*. In all cases, the chelated form is preferred but considerably stronger for enolate bearing smaller cations (by 13.5, 23.6, and 44.4 kJ/mol for K, Na, and Li, respectively). On the contrary, terminal alkyne coordinates unsymmetrically to the Pd center causing a natural preference for *exo* cyclization. Slippage of Pd along the π-system of the alkyne associated with the *endo* attack of the nucleophile requires some energy. The interplay of these two phenomena presumably determines the selectivity of the discussed process.

**Table 1:**
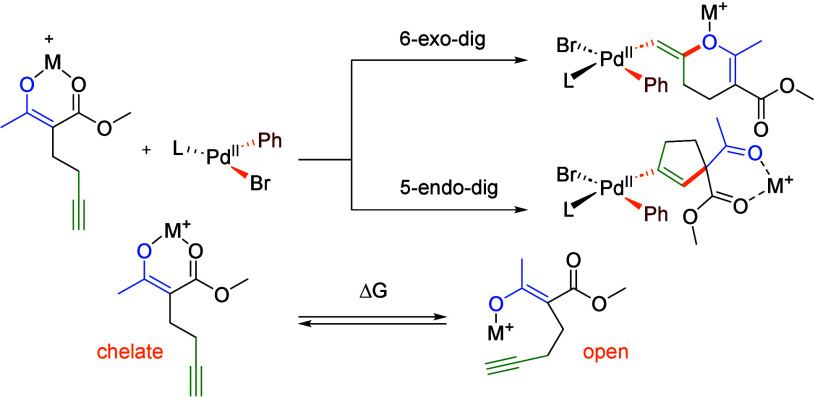
Density Functional Theory Insight into the Regioselectivity of the Cyclization of Acetylenic Ketoester Triggered by ^*i*^Pr-XPhosPd(Ph)Br

		Δ*G*^⧧^ (kJ/mol)^a^,^b^		
entry	M	5-*endo*	6-*exo*	Δ*G* (kJ/mol) (chelate/open^b^,^c^)
1	H	115.0	130.4	
2	–	61.1	49.3	
3	K	82.9	67.3	13.5
4	Na	85.5	84.7	23.6
5	Li	91.6	118.5	44.2
6	Li(DMF)_2_	96.2	112.9	37.7

a Gibbs free energy of activation [difference between Gibbs free energies of the transition states and the sum of iPrXphosPd(Ph)Br and the corresponding enolate].

b Calculated at the M06/def2-TZVPP/SMD(DMF)//B3LYP-D3/def2-SVP level of theory.

c Δ*G* of the equilibrium of the chelated and open form of the enolate.

For the sake of simplicity and easier comparison, alkali metal cations significantly differing in ionic radii and preferred coordination sphere complexes with “naked” cations were investigated. Considering that the solvation of metal centers can alter the electronic and steric properties of the metal, structures bearing two additional solvent (DMF) molecules in the coordination sphere of lithium were also computed (entry 6). This modification only slightly altered the calculated activation barriers and did not impact the predicted selectivity pattern.

This prompted us to experimentally test whether the presence of a hard Lewis acid can switch the selectivity in the Pd-catalyzed cyclization/coupling of terminal α-homopropargyl-β-ketoesters. Preliminary investigation of the model reaction of methyl 2-acetylhex-5-ynoate **1** with bromobenzene, conducted in DMF in the presence of K_2_CO_3_ and Pd/Xphos, revealed that the addition of a hard Lewis acid (e.g., ZnBr_2_ or FeCl_3_) resulted in the formation of some amount of cyclopentene **2** along with benzylidenedihydropyran **3**. The latter is the sole product in the absence of the additive. Further evaluation of a range of reaction variables (Pd catalyst, base, additive, solvent, stoichiometry, and temperature) established satisfactory reaction conditions, employing 2 mol % XPhos Pd G3 as a precatalyst, 10 mol % FeCl_3_, and LiOH as a base (Table 2).^15^ The use of a lithium base (LiOH performed best) turned out to be crucially important in providing high selectivity toward 5*-endo-dig* cyclization, even in the absence of FeCl_3_, which contrasts with all of the other bases tested. The reaction was conducted at 50 °C in a 1:1 mixture of dimethylformamide (DMF) and dichloroethane (DCE) in the presence of 4 Å molecular sieves. The reactions performed solely in DMF feature poor selectivity, while the use of DCE improved selectivity, albeit at the cost of a considerably lower yield. It should be stated here that no byproduct resulting from Sonogashira coupling was observed during the optimization process, presumably due to the absence of copper salts.

**Table 2:**
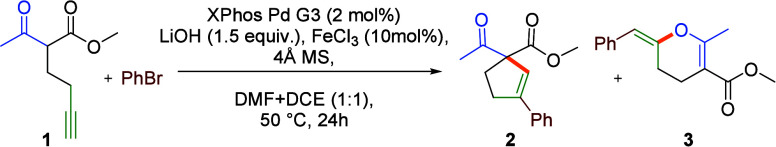
Evaluation of the Reaction Conditions

entry	variable	yield of **2**^b^ (%)	yield of **3**^b^ (%)
1^a^	none	86	10
2^a^	no FeCl_3_	58	13
3^a^	no molecular sieves	52	10
4^a^	room temperature	45	9
5^a^	80 °C	37	8
6^a^	only DCE (1 mL)	50	0
7^a^	only DMF (1 mL)	57	43

a Reaction conditions: **1** (0.15 mmol), PhBr (0.10 mmol), LiOH (0.15 mmol), FeCl (0.01 mmol), XPhos Pd G3 (2.00 μmol), powdered 4 Å MS (80 mg), DCE (0.5 mL), DMF (0.5 mL), 24 h, 50 °C.

b Yield calculated via GC with mesitylene as the internal standard.

Having established satisfactory conditions for the benchmark reaction, we initially investigated the scope with respect to aryl bromides (Table 3). With methyl 2-acetylhex-5-ynoate **1**, both electron-rich and electron-deficient aryl halides were well tolerated, giving rise to substituted cyclopentenes with good yields.

**Table 3:**
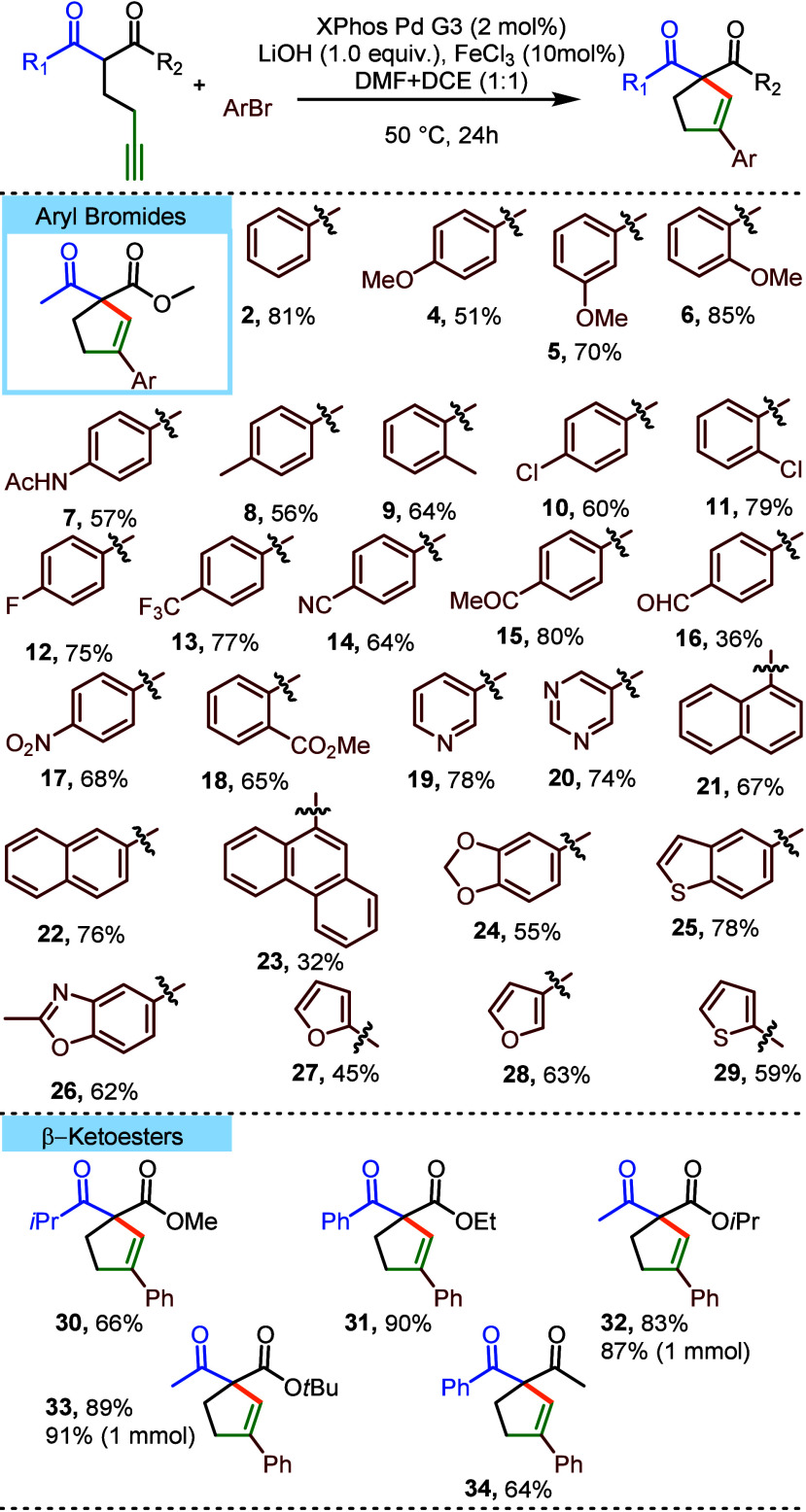
Scope of Synthesized Products^a^

a Reaction conditions: dicarbonyl compound (0.60 mmol), ArBr (0.40 mmol), XPhos Pd G3 (6.77 mg, 8.00 μmol), LiOH (0.44 mmol), FeCl_3_ (0.04 mmol), DMF (1 mL), DCE (1 mL), powdered 4 Å MS (320 mg), 50 °C, 24 h.

Sterically demanding *ortho*-substituted bromoarenes served as competent reaction partners, and their reactions were slightly more productive than those of *para*-substituted analogues (cf. **4** with **6** and **10** with **11**). The transformation exhibits excellent functional group tolerance, including unprotected aldehydes, enolizable ketones, esters, amides, aryl chlorides, nitroarenes, and nitriles, among others. Moreover, heteroaryl bromides, including pharmaceutically relevant pyridines and pyrimidines, were also compatible with the reaction conditions.

Subsequently, the applicability of other β-dicarbonyl substrates was tested in the reaction with bromobenzene. Swapping of the methyl group in the ketone moiety of model ketoester **1** with a branched ^*i*^Pr (**30**) or phenyl (**31**) group was well tolerated, in contrast to bulky ^*t*^Bu whose presence completely stifles the reactivity. Installation of sterically demanding *^i^*Pr or *^t^*Bu groups on the ester fragment of the acetylenic ketoester had little impact on the reaction outcome (**32** and **33**). Furthermore, acetylenic β-diketone also produced the expected cyclopentene (**34**) in a satisfactory yield.

We also considered the possibility that aryl and vinyl triflates could serve as electrophilic partners of the developed reaction (Table 4). Interestingly, the addition of FeCl_3_ had no impact on the reaction outcome in this case, and the transformation proceeded efficiently and selectively in DCE. Reaction with phenyl triflate delivered the expected cyclopentene **2** in an excellent 92% yield, higher than that of the model reaction with bromobenzene. Vinyl triflates proved to be competent reaction partners, leading to the corresponding dienes (**36** and **37**) in good yields. Finally, the suitability of the method for the functionalization of naturally occurring scaffolds was demonstrated through the efficient reaction of triflates derived from 4-hydroxycoumarin (**38**), eugenol (**39**), and estrone (**40**).

**Table 4:**
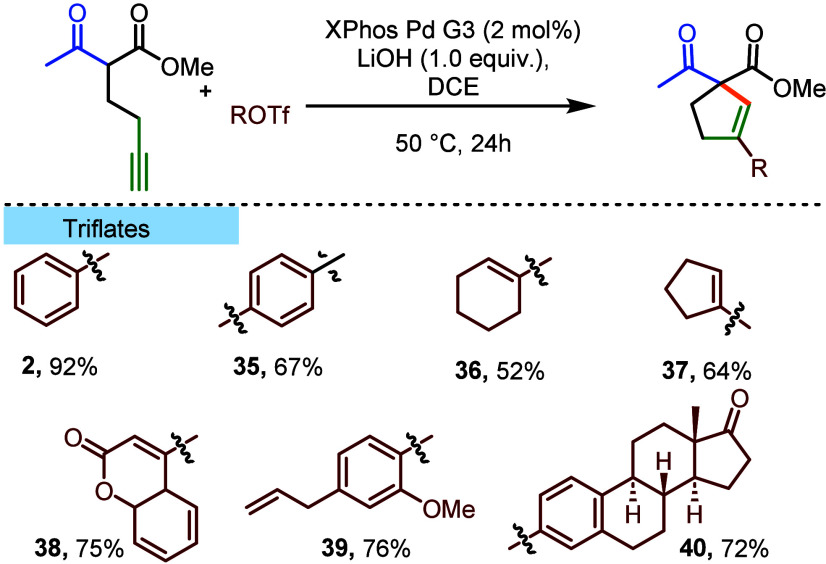
Scope of the Synthesized Products^a^

a Reaction conditions: dicarbonyl compound (0.60 mmol), ROTf (0.40 mmol), XPhos Pd G3 (6.77 mg, 8.00 μmol), LiOH (0.44 mmol), DCE (2 mL), powdered 4 Å MS (320 mg), 50 °C, 24 h.

The postulated reaction mechanism involves the oxidative addition of an aryl halide to the Pd(0) complex, intramolecular nucleophilic addition triggered by the coordination of the resulting aryl-Pd(II) to the alkyne moiety of the acetylenic ketoester, and, finally, reductive elimination. The crucial role in the process, enabling 5*-endo-dig* carbocyclization rather than naturally preferred 6*-exo-dig* oxocyclization, is associated with the formation of lithium enolate. Presumably, lithium tightly chelates a β-dicarbonyl system impeding the attack of the O center of the enolate on the alkyne. Formation of the lithium enolate is not a rate-limiting step, which can be inferred from the absence of a kinetic isotope effect in the parallel reactions of **1** and its α-deuterated isotopologue **1-D** (Scheme 1). Finally, the model reaction was also investigated computationally. All of the calculations were performed with Gaussian 16 at the M06/def2-TZVPP/SMD(DMF)//B3LYP-D3/def2-SVP level of theory. The model reaction involved the reaction of lithium enolate of **1** with bromobenzene mediated by the ^*i*^Pr-Xphos-Pd complex (cyclohexyl groups at the P center of XPhos were trimmed to ^*i*^Pr). To better reflect the first coordination sphere, two molecules of DMF binding to the Li center were included. The Gibbs free energy profile of the model reaction is depicted in Figure 2.

**Figure 2:**
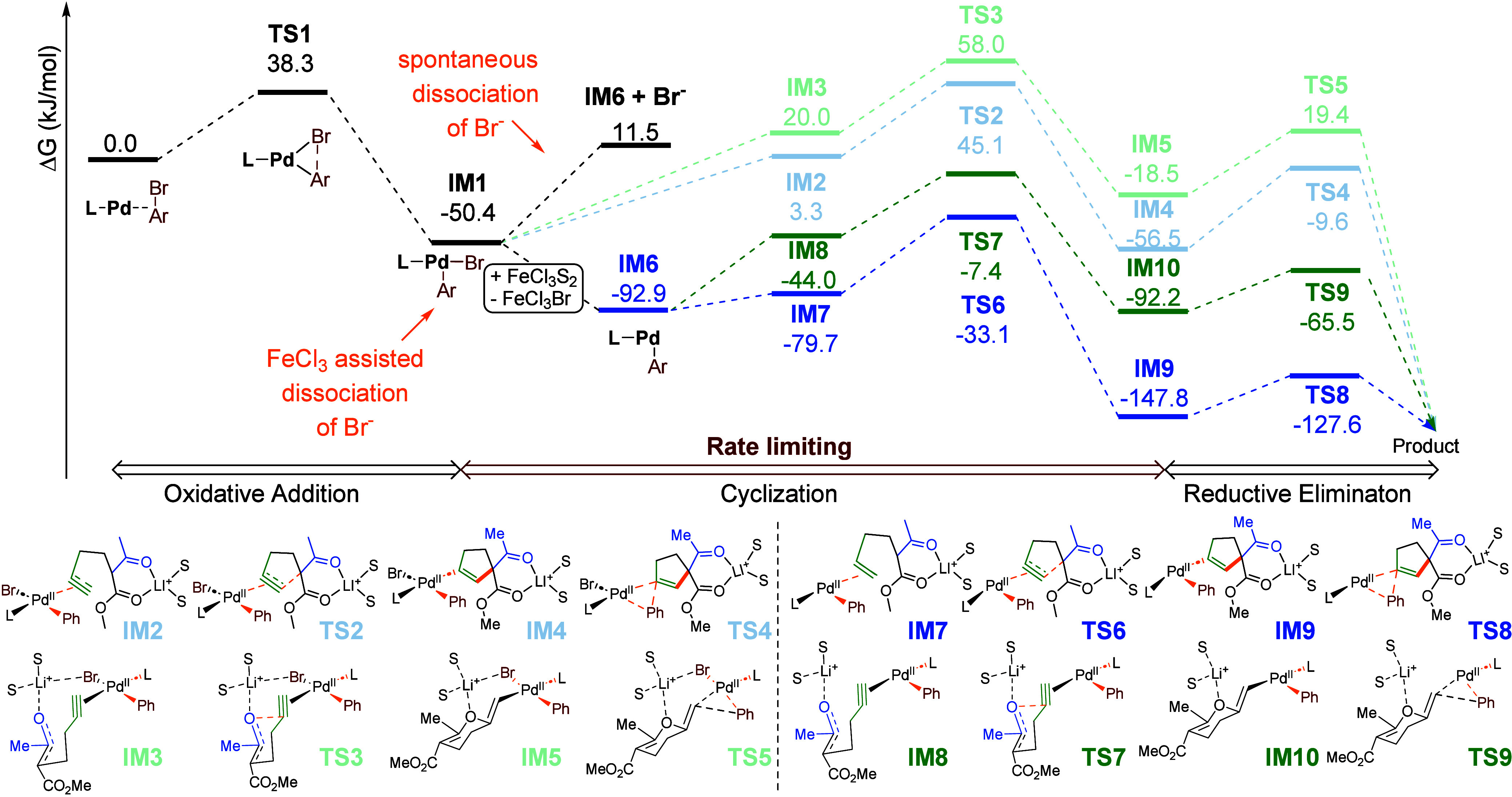
Gibbs free energy profile of the model reaction.

**Scheme 1:**
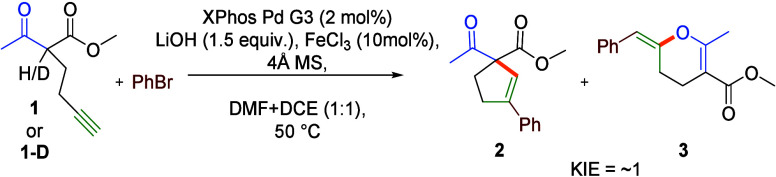
Kinetic Isotope Effect of the Model Reaction

Facile oxidative addition via **TS1** (Δ*G*^⧧^ = 38.3 kJ/mol) leads to aryl-Pd complex **IM1**, in which the aryl group of the phosphine ligand weakly binds to the Pd center; such a rare weak η^1^ Pd–C(ipso) interaction, responsible for the reversible shielding of the metal center, is typical for biaryl phosphine ligands.^16^ A change in the conformation of the phosphine ligand in **IM1** opens a coordination site on the metal center, enabling coordination of the alkyne fragment to trigger subsequent intramolecular attack of the enolate. Carbocyclization through a 5*-endo-dig* manifold (pale blue) was identified as being more viable (**TS2**; Δ*G*^⧧^ = 95.4 kJ/mol) compared to the attack of the O center of the enolate along the 6-*exo*-*dig* pathway (pale green), proceeding through **TS3** and associated with an activation barrier *G*^⧧^ of 108.4 kJ/mol (almost 13 kJ/mol higher than that of **TS2**). The catalytic cycle is completed with facile reductive elimination from resulting complex **IM4** or **IM5** via **TS4** (Δ*G*^⧧^ = 46.9 kJ/mol) or **TS5** (Δ*G*^⧧^ = 37.9 kJ/mol), respectively.

The other mechanistic scenario considered involves the coordination of the alkyne to the Pd center, proceeding through the substitution of bromide. Dissociation of bromide, leading to cationic Pd species **IM6**, is energetically unfavorable (Δ*G* = 61.9 kJ/mol). The departure of the bromide may, however, be assisted by interaction with FeCl_3_, leading to a weakly coordinating tetrahaloferrate. Its dissociation leading to **IM6** is highly favorable (Δ*G* = −42.5 kJ/mol), comparable to that of triflate from the analogous complex (Δ*G* = −42.9 kJ/mol). Binding of the alkyne moiety to **IM6** triggers cyclization. 5*-endo-dig* carbocyclization (blue path) proceeding through **TS6** (Δ*G*^⧧^ = 59.8 kJ/mol) is preferred over 6-*exo*-*dig* oxocyclization (green path; **TS7**; Δ*G*^⧧^ = 85.5 kJ/mol). Finally, reductive elimination delivering cyclopetenes (via **TS8**) or dihydropyrans (via **TS9**) is associated with only small barriers (Δ*G*^⧧^ = 20.2 or 26.7 kJ/mol, respectively).

In conclusion, the inherent preference for 6*-exo-dig* oxocyclization of α-homopropargyl-β-ketoesters toward dihydropyrans in Pd-catalyzed cyclization/coupling with aryl bromides is overcome by the application of a lithium base. The 5*-endo-dig* attack of a C center of the enolate on the alkyne fragment, activated by coordination of the aryl-palladium species, is enabled by the tight chelation of the β-dicarbonyl system by a lithium cation. The developed protocol delivering substituted cyclopentenes features mild conditions, high yields, and functional group tolerance. The methodology can be applied to various α-homopropargyl-β-ketoesters, a broad range of aryl and heteroaryl bromides, and aryl and vinyl triflates.

## Data Availability

The data underlying this study are available in the published article and its Supporting Information.

## Supporting Information Available

Evaluation of the reaction conditions, details of optimization and control experiments, experimental procedures, characterization of all compounds, and details of computational studies (PDF)

## References

[ref1] aBalme G.; Bouyssi D.; Lomberget T.; Monteiro N. Cyclisations Involving Attack of Carbo- and Heteronucleophiles on Carbon-Carbon π-Bonds Activated by Organopalladium Complexes. Synthesis 2003, 2115–2134. 10.1055/s-2003-42082.

[ref2] Hack D.; Blümel M.; Chauhan P.; Philipps A. R.; Enders D. Catalytic Conia-Ene and Related Reactions. Chem. Soc. Rev. 2015, 44, 6059–6093. 10.1039/C5CS00097A. 26031492

[ref3] aAlabugin I. V.; Gilmore K.; Manoharan M. Rules for Anionic and Radical Ring Closure of Alkynes. J. Am. Chem. Soc. 2011, 133, 12608–12623. 10.1021/ja203191f. 21675773

[ref4] aStaben S. T.; Kennedy-Smith J. J.; Toste F. D. Gold(I)-Catalyzed 5-Endo-Dig Carbocyclization of Acetylenic Dicarbonyl Compounds. Angew. Chem., Int. Ed. 2004, 43, 5350–5352. 10.1002/anie.200460844. 15468061

[ref5] Corkey B. K.; Toste F. D. Catalytic Enantioselective Conia-Ene Reaction. J. Am. Chem. Soc. 2005, 127, 17168–17169. 10.1021/ja055059q. 16332048

[ref6] Barluenga J.; Palomas D.; Rubio E.; González J. M. Iodocarbocyclization Reaction of β-Ketoesters and Alkynes. Org. Lett. 2007, 9, 2823–2826. 10.1021/ol0710459. 17585771

[ref7] aSuzuki S.; Tokunaga E.; Reddy D. S.; Matsumoto T.; Shiro M.; Shibata N. Enantioselective 5-Endo-Dig Carbocyclization of β-Ketoesters with Internal Alkynes Employing a Four-Component Catalyst System. Angew. Chem., Int. Ed. 2012, 51, 4131–4135. 10.1002/anie.201201060. 22422729

[ref8] aChan L. Y.; Kim S.; Park Y.; Lee P. H. Iron(III)-Catalyzed Conia–Ene Cyclization of 2-Alkynic 1,3-Dicarbonyl Compounds. J. Org. Chem. 2012, 77, 5239–5244. 10.1021/jo300957q. 22632410

[ref9] aHolman K. R.; Stanko A. M.; Reisman S. E. Palladium-Catalyzed Cascade Cyclizations Involving C–C and C–X Bond Formation: Strategic Applications in Natural Product Synthesis. Chem. Soc. Rev. 2021, 50, 7891–7908. 10.1039/D0CS01385D. 34037626

[ref10] aFujino D.; Yorimitsu H.; Osuka A. Synthesis of 1,2-Disubstituted Cyclopentenes by Palladium-Catalyzed Reaction of Homopropargyl-Substituted Dicarbonyl Compounds with Organic Halides via 5-Endo-Dig Cyclization. Org. Lett. 2012, 14, 2914–2917. 10.1021/ol301257m. 22612561

[ref11] aDuan X.-H.; Guo L.; Bi H.-P.; Liu X.-Y.; Liang Y.-M. Synthesis of 2-Substitued 3-Aroylindenes via Palladium-Catalyzed Carbonylative Cyclization of Diethyl 2-(2-(1-Alkynyl)Phenyl)Malonates with Aryl Halides. Org. Lett. 2006, 8, 3053–3056. 10.1021/ol061019v. 16805550

[ref12] aKołodziejczyk A.; Domański S.; Chaładaj W. Tandem Palladium-Catalyzed 6-Exo-Dig Oxocyclization Coupling of δ-Acetylenic β-Ketoesters with Aryl Bromides and Chlorides: Route to Substituted Dihydropyrans. J. Org. Chem. 2018, 83, 12887–12896. 10.1021/acs.joc.8b01832. 30204434

[ref13] Frisch M. J.; Trucks G. W.; Schlegel H. B.; Scuseria G. E.; Robb M. A.; Cheeseman J. R.; Scalmani G.; Barone V.; Petersson G. A.; Nakatsuji H.; Li X.; Cari-Cato M.; Marenich A. V.; Bloino J.; Janesko B. G.; Gomperts R.; Mennucci B.; Hratchian H. P.; Ortiz J. V.; Izmaylov A. F.; Sonnenberg J. L.; Williams-Young D.; Ding F.; Lipparini F.; Egidi F.; Goings J.; Peng B.; Petrone A.; Henderson T.; Ranasinghe D.; Zakrzewski V. G.; Gao J.; Rega N.; Zheng G.; Liang W.; Hada M.; Ehara M.; Toyota K.; Fukuda R.; Hasegawa J.; Ishida M.; Nakajima T.; Honda Y.; Kitao O.; Nakai H.; Vreven T.; Throssell K.; Montgomery J. A. Jr.; Peralta J. E.; Ogliaro F.; Bearpark M. J.; Heyd J. J.; Brothers E. N.; Kudin K. N.; Staroverov V. N.; Keith T. A.; Kobayashi R.; Normand J.; Raghavachari K.; Rendell A. P.; Burant J. C.; Iyengar S. S.; Tomasi J.; Cossi M.; Millam J. M.; Klene M.; Adamo C.; Cammi R.; Ochterski J. W.; Martin R. L.; Morokuma K.; Farkas O.; Foresman J. B.; Fox D. J. Gaussian 16, rev. C.01; Gaussian, Inc.: Wallingford, CT, 2019.

[ref14] Raban M.; Noe E. A.; Yamamoto G. Stereochemical Consequences of Ionic Bonding in Alkali Metal Acetylacetonates. J. Am. Chem. Soc. 1977, 99, 6527–6531. 10.1021/ja00462a010.

[ref15] See the Supporting Information for more details.

[ref16] aReid S. M.; Boyle R. C.; Mague J. T.; Fink M. J. A Dicoordinate Palladium (0) Complex with an Unusual Intramolecular η-Arene Coordination. J. Am. Chem. Soc. 2003, 125, 7816–7817. 10.1021/ja0361493. 12822996

