# Supplementary material for: Lithium Enables Pd-Catalyzed 5-endo-dig Cyclization/Coupling of α-Homopropargyl-β-ketoesters with Aryl Bromides and Triflates
Source: Org Lett. 2024 Sep 19;26(39):8254–9. doi: 10.1021/acs.orglett.4c02846 (PMC11459523; doi:10.1021/acs.orglett.4c02846)
Supplement: Supplementary file 1 — ol4c02846_si_001.pdf [file ol4c02846_si_001.pdf]

Supporting information

Lithium enables Pd-catalyzed *5-endo-dig* cyclization-coupling of α-homopropargyl-β-ketoesters with aryl bromides and triflates

Bartosz Bisek, Katarzyna Kochaniak, Wojciech Chaładaj*

Institute of Organic Chemistry, Polish Academy of Sciences, Kasprzaka 44/52, 01-224 Warsaw, Poland
wojciech.chaladaj@icho.edu.pl

Table of contents

Supporting information 1
Table of contents 1
General Information 2
Materials 2
Evaluation of reaction conditions for Pd-catalyzed 5-endo-dig cyclization/coupling of terminal alkynes 3
Control experiments 7
Reaction procedures 9
Computational Studies 20
Copies of ^{1}H and ^{13}C{1H} NMR spectra of isolated compounds 111
References 149

General Information

All the manipulations were performed in a nitrogen-filled glovebox or under an argon atmosphere using Schlenk techniques, unless mentioned otherwise. Flash chromatography was performed using Merck silica gel 60 (230-400 mesh). TLC analysis of reaction mixtures was performed on Merck silica gel 60 F254 TLC plates and visualized with cerium molybdate stain (Hanessian's stain). ^{1}H, ^{13}C{1H}, and ^{19}F NMR spectra were recorded with a Bruker AV 400 spectrometer. ^{1}H and ^{13}C chemical shifts are given in ppm relative to TMS. The solvent signals were used as references ($CDCl_3 \delta_H$ = 7.26 ppm, δ_C = 77.0 ppm) and the chemical shift converted to the TMS scale. Coupling constants (*J*) are reported in Hz, and the following abbreviations were used to denote multiplets: s = singlet, d = doublet, t = triplet, q = quartet, quint = quintet, m = multiplet (denotes complex pattern), dd = doublet of doublets, dt = doublet of triplets and br = broad signal. Infrared spectra were recorded with a Jasco FTIR-6200 spectometer. Electron ionization high-resolution mass spectra (EI-HR) were recorded with an Autospec Premier (Waters Inc) mass spectrometer equipped with an electron impact (EI) ion source and the EBE double focusing geometry mass analyzer, using the narrow-range high-voltage scan technique with low-boiling perfluorokerosene (PFK) as internal standard. Samples were introduced by using a heated direct insertion probe. The instrument was controlled and recorded data were processed using MassLynx 4.1 software package (Waters Inc). Electrospray ionization high-resolution mass spectra (ESI-HR) were carried out using Synapt G2-S mass spectrometer (*Waters Inc*) equipped with the electrospray (ESI) ion source and quadrupole-Time-of-flight (qTOF) mass analyzer (*Waters Inc)*. The measurements were performed with the resolving power of TOF analyzer 20000 FWHM. The lock-spray spectrum of Leucine-enkephalin was generated by the lock-spray source and the correction was performed for the recorded spectrum in the mass range of m/z = 50-1200. The exact mass measurements were performed within 3 mDa mass error. The instrument was controlled and recorded data were processed using MassLynx V4.1 software package (Waters Inc).

Materials

Unless otherwise noted, all commercially available compounds (ABCR, Acros, Fluorochem, TCI, Sigma-Aldrich, Strem) were used as received. Buchwald-type 3rd-generation palladacyclic precatalysts (Ligand Pd G3) were prepared following literature procedure[1], and showed similar reactivity to the commercial samples. Trifluoromethanesulfonates were prepared according to the literature procedure[2,3]. Dry solvents were acquired by the use of a solvent purification system (SPS).

Evaluation of reaction conditions for Pd-catalyzed 5-endo-dig cyclization/coupling of terminal alkynes

General procedure for evaluation of reaction conditions: In a glovebox, to a 4-mL screw-capped vial containing catalyst and stirring bar following reagents ware added in exact order: $FeCl_3$ (1.62 mg, 0.01 mmol, 0.1 eq), base (0.15 mmol, 1.5 eq), molecular sieves 4Å (80 mg), solvent (1 mL), bromobenzene (15.7 mg, 0.1 mmol, 1 eq), methyl 2-acetylhex-5-ynoate (25.2 mg, 0.15 mmol, 1.5 eq). Then, the vial was sealed with a cap containing Teflon seal and removed from glovebox. The reaction mixture was stirred at given temperature in the heating block for 24 h. After that, the vial was opened at normal atmosphere. The mixture was diluted with DCM (1 mL) quenched with sat. aquas NH_4Cl (1 mL) and mezytylene (15 µl) was added as an internal standard.

1. Effect of catalyst

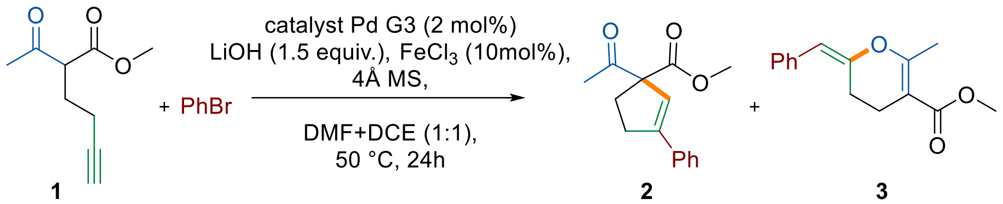

Entry	Catalyst Pd G3 2mol%[a]	Yield 2[b]	Yield 3[b]
**1**	**XPhos 2mol%**	**86%**	**10%**
2	XPhos 5mol%	86%	13%
3	XPhos 1mol%	73%	12%
4	XPhos 0.5mol%	75%	13%
5	XPhos 0.2mol%	53%	10%
2	APhos	43%	5%
3	DavePhos	29%	3%
4	BrettPhos	21%	7%
5	MonoPhos	7%	0%
6	SPhos	47%	8%
7	RuPhos	26%	3%
8	JackiePhos	29%	4%
9	t-butPhos	0%	0%
10	CyJohnPhos	6%	0%
11	DPePhos	14%	2%
12	DCyPf	14%	0%
13	Cy_2PPh	34%	0%
14	PPh_3	16%	0%
15	PCy_3	15%	0%
16	$P(o\text{-}tol)_3$	13%	0%
17	Cataxium	51%	7%
18	BINAP	9%	2%
19	TolBINAP	10%	2%
20	DPPE	5%	2%
21	DPPF	6%	1%
22	DPPM	22%	4%
23	DPPB	2%	0%

[a]Conditions: catalyst Pd G3 (2 mol%), methyl 2-acetylhex-5-ynoate (0.15 mmol, 1.5 eq), bromobenzene (0.10 mmol, 1.0 eq), LiOH (0.15 mmol, 1.5 eq), $FeCl_3$ (0.01 mmol, 0.1 eq), molecular sieves 4Å (80

mg), DMF (0.5 mL), DCE (0.5 mL), 50°C, 24h; [b]determined by GC with mezytylene as an internal standard.

2. Effect of base

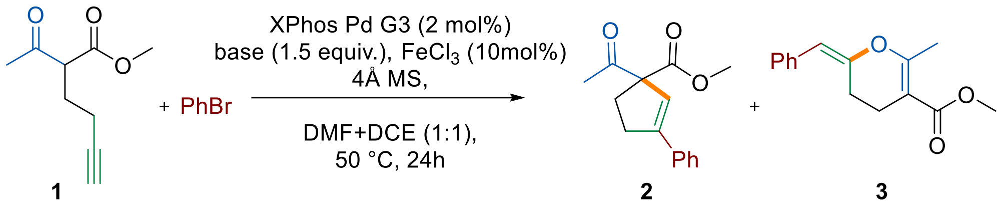

Entry	base[a]	Yield 2[b]	Yield 3[b]
**1**	**LiOH**	**86%**	**10%**
2	LiOtBu	52%	6%
3	MeOLi	52%	15%
4	Li_2CO_3	1%	0%
5	LiHMDS	13%	2%
6	LiH	0%	0%
7	NaOH	14%	16%
8	NaOtBu	2%	5%
9	MeONa	15%	58%
10	Na_2CO_3	1%	0%
11	NaHMDS	17%	63%
12	NaH	3%	6%
13	KOH	2%	9%
14	KOtBu	2%	30%
15	MeOK	0%	3%
16	K_2CO_3	2%	17%
17	K_3PO_4	0%	15%
18	KHMDS	2%	2%
19	KOAc	1%	0%
20	Cs_2CO_3	2%	15%
21	CsF	0%	0%
22	Et_3N	2%	2%
23	DMAP	0%	0%
24	DBU	0%	0%

[a]Conditions: XPhos Pd G3 (2 mol%), methyl 2-acetylhex-5-ynoate (0.15 mmol, 1.5 eq), bromobenzene (0.10 mmol, 1.0 eq), base (0.15 mmol, 1.5 eq), $FeCl_3$ (0.01 mmol, 0.1 eq), molecular sieves 4Å (80 mg), DMF (0.5 mL), DCE (0.5 mL), 50°C, 24h; [b]determined by GC with mezytylene as an internal standard.

3. Effect of solvent

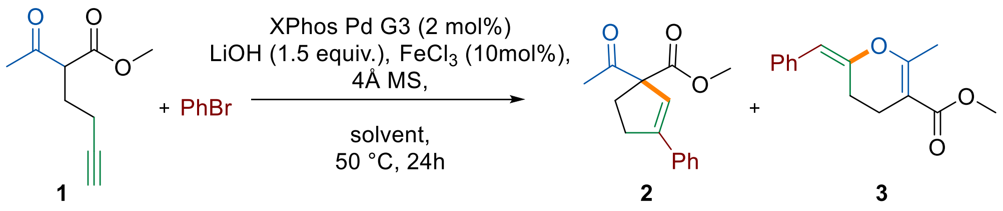

Entry	Solvent[a]	Yield 2[b]	Yield 3[b]
**1**	**DMF (0.5 mL) + DCE (0.5 mL)**	**86%**	**10%**
2	DMF (1.0 mL) + DCE (1.0 mL)	80%	18%
3	DMF (0.25 mL) + DCE (0.25 mL)	83%	11%
4	DMF (0.8 mL) + DCE (0.2 mL)	68%	30%
5	DMF (0.2 mL) + DCE (0.8 mL)	63%	3%
6	DMF (1.0 mL)	57%	43%
7	DCE (1.0 mL)	50%	0%
8	NMP (1.0 mL)	53%	35%
9	NMP (0.5 mL) + DCE (0.5 mL)	82%	14%
10	DMSO (1.0 mL)	17%	51%
11	MeCN (1.0 mL)	29%	0%
12	DCM (1.0 mL)	32%	0%
13	THF (1.0 mL)	10%	0%
14	Dioxane (1.0 mL)	50%	0%
15	Toluene (1.0 mL)	38%	0%
16	Ethyl Acetate (1.0 mL)	13%	0%
17	MeOH (1.0 mL)	38%	0%

[a]Conditions: XPhos Pd G3 (2 mol%), methyl 2-acetylhex-5-ynoate (0.15 mmol, 1.5 eq), bromobenzene (0.10 mmol, 1.0 eq), LiOH (0.15 mmol, 1.5 eq), $FeCl_3$ (0.01 mmol, 0.1 eq), molecular sieves 4Å (80 mg), solvent, 50°C, 24h; [b]determined by GC with mezytylene as an internal standard.

4. Effect of lewis acid

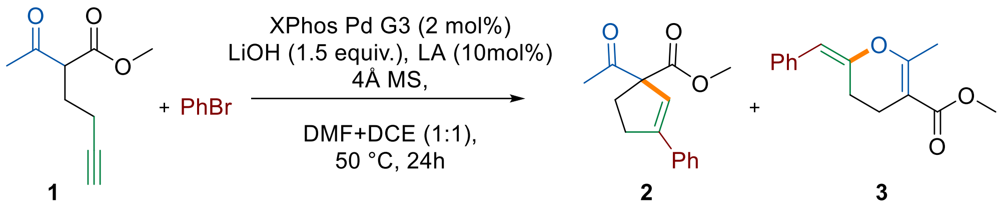

Entry	Lewis Acid[a]	Yield 2[b]	Yield 3[b]
**1**	**$FeCl_3$**	**86%**	**10%**
2	$FeCl_2$	63%	10%
3	$FeOTf_3$	78%	13%
4	$FeOTf_2$	77%	13%
5	$FeBr_3$	37%	6%
6	FeI_2	44%	8%
7	$ZnCl_2$	64%	12%
8	CuI	0%	0%

[a]Conditions: XPhos Pd G3 (2 mol%), methyl 2-acetylhex-5-ynoate (0.15 mmol, 1.5 eq), bromobenzene (0.10 mmol, 1.0 eq), LiOH (0.15 mmol, 1.5 eq), lewis acid (0.01 mmol, 0.1 eq), molecular sieves 4Å (80 mg), DMF (0.5 mL), DCE (0.5 mL), 50°C, 24h; [b]determined by GC with mezytylene as an internal standard.

1. Variation from reaction conditions

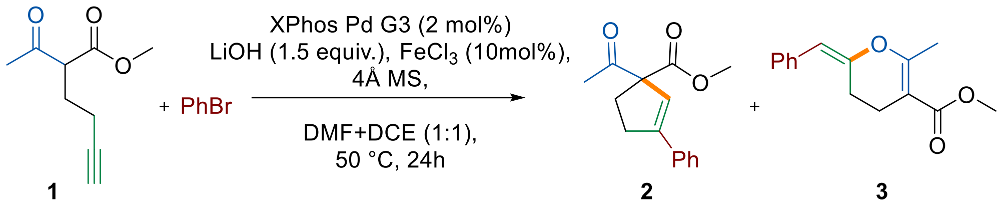

Entry	Modification[a]	Yield 2[b]	Yield 3[b]
**0**	**- - -**	**86%**	**10%**
1	No $FeCl_3$	58%	13%
2	5mol% $FeCl_3$	64%	12%
3	20mol% $FeCl_3$	71%	12%
4	No molecular sieves	52%	10%
5	20mg of molecular sieves	56%	10%
6	160mg of molecular sieves	78%	15%
7	Room temperature	45%	9%
8	80°C	37%	8%
9	Only DCE (1 mL), 80°C	28%	9%
10	PhI	28%	5%

[a]Conditions: XPhos Pd G3 (2 mol%), methyl 2-acetylhex-5-ynoate (0.15 mmol, 1.5 eq), bromobenzene (0.1 mmol, 1.0 eq), LiOH (0.15 mmol, 1.5 eq), $FeCl_3$ (0.01 mmol, 0.1 eq), molecular sieves 4Å (80 mg), DMF (0.5 mL), DCE (0.5 mL), 50°C, 24h; [b]determined by GC with mezytylene as an internal standard.

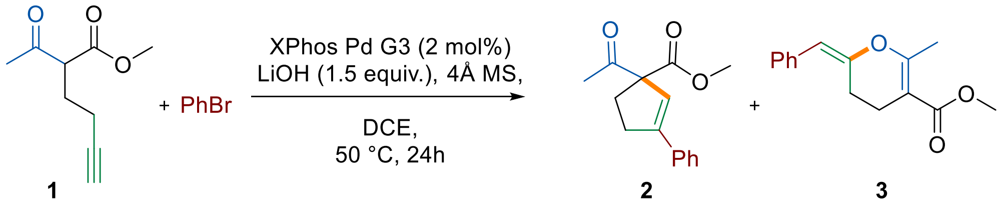

Entry	Modification[a]	Yield 2[b]	Yield 3[b]
**0**	**NONE**	**92%**	**0%**
1	+ $FeCl_3$ (10 mol%)	91%	0%
2	+ DMF (1 mL)	85%	10%
3	DMF only (2 mL)	51%	48%

[a]Conditions: XPhos Pd G3 (2 mol%), methyl 2-acetylhex-5-ynoate (0.6 mmol, 1.5 eq), bromobenzene (0.4 mmol, 1.0 eq), LiOH (0.6 mmol, 1.5 eq), molecular sieves 4Å (320 mg), DCE (2 mL), 50°C, 24h; [b]yield of isolated product.

Control experiments

1. Reaction profile

In a glovebox, to a 10-mL Schlenk flask containing XPhos Pd G3 (16.93 mg, 20.0 μmol) and magnetic stirring bar following reagents were added in exact order: LiOH (36.0 mg, 1.50 mmol), $FeCl_3$ (16.20 mg, 0.04 mmol), blend molecular sieves 4 Å (1.2 g), DMF (2.5 mL), DCE (2.5 mL). Content of the vial was briefly stirred followed by addition of aryl bromide (157.0 mg, 1.0 mmol), dicarbonyl compound (252.3 mg, 0.60 mmol) and mezytylene (0.15 mL). Then, the Schlenk flask was sealed with a septum and removed from glovebox. The reaction mixture was stirred at 50°C in the heating block for 24 h and portions of reaction mixture were collected at the specified time. The mixture was diluted with DCM (1 mL) quenched with sat. aquas NH_4Cl (1 mL).

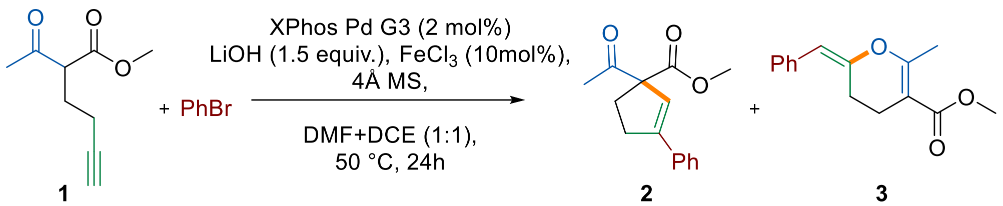

Entry	Time (h)	Yield 2[b]	Yield 3[b]
1	0.25	0%	0%
2	0.5	6%	1%
3	1.0	21%	2%
4	2.0	50%	5%
5	4.0	73%	7%
6	6.0	79%	8%
7	8.0	80%	8%
8	12.0	82%	8%
9	24.0	83%	8%

[a]Conditions: XPhos Pd G3 (2.0 mol%), methyl 2-acetylhex-5-ynoate (1.5 mmol, 1.5 equiv), bromobenzene (1.0 mmol, 1.0 equiv), LiOH (1.5 mmol, 1.5 equiv), $FeCl_3$ (0.1 mmol, 0.1 eq) molecular sieves 4Å (1 200 mg), DMF (2.5 mL), DCE (2.5 mL); [b]determined by GC with mesitylene as an internal standard.

2. Competition experiment

In a glovebox, to a measuring cylinder containing XPhos Pd G3 (3.38 mg, 4.0 μmol) following reagents were added in exact order: $FeCl_3$ (3.36 mg, 0.02 mmol), DMF (1.0 mL), DCE (1.0 mL) followed by aryl bromide (31.4 mg, 0.2 mmol), and mezytylene (30 μL). Content of the measuring flask was briefly mixed. 1 mL of the resulting mixture was added to the two separate 4-mL vials each containing magnetic stirring bar, LiOH (3.60 mg, 0.15 mmol) and molecular sieves 4 Å (80 mg). Content of the vials was stirred briefly, followed by addition of methyl 2-acetylhex-5-ynoate (25.23 mg, 0.15 mmol) and deuterated analogue (25.38 mg, 0.15 mmol) to the separate vials. Then, the vials were sealed with a cap containing a PTFE septum and removed from glovebox. The reaction mixture was stirred at 50°C in the heating block and portions of reaction mixture were collected at the specified time and given for GC measurements.

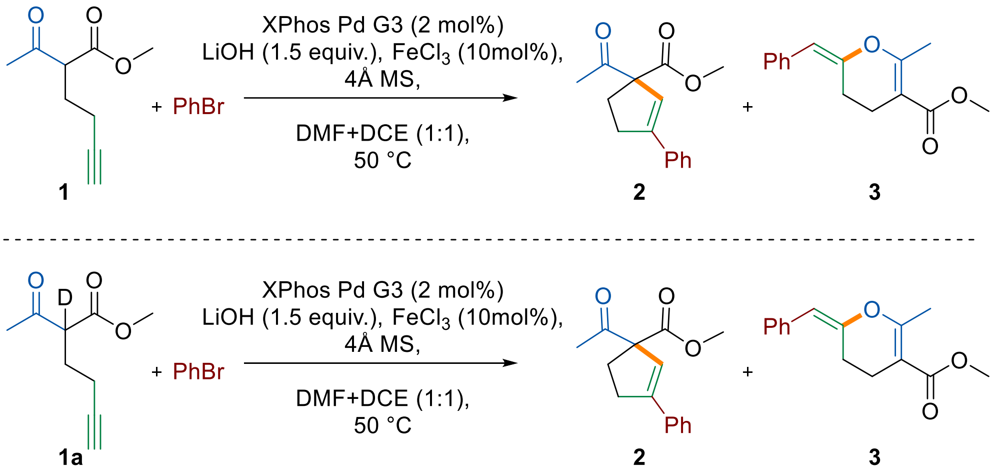

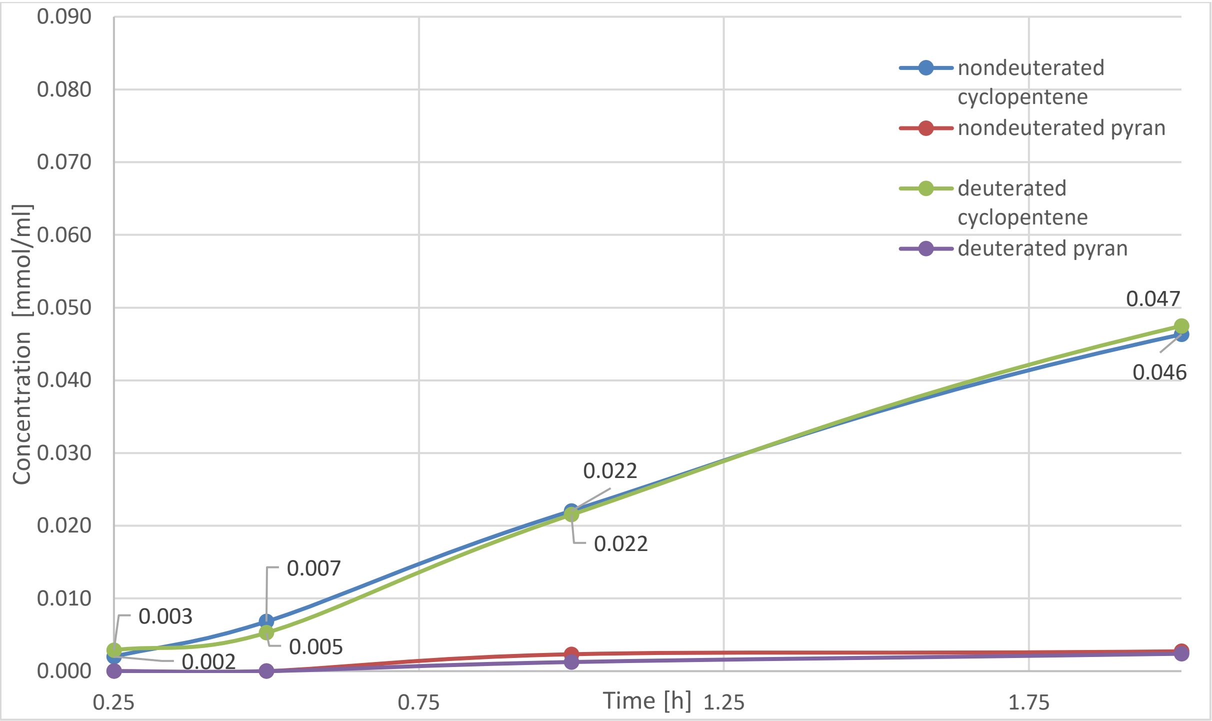

[a]Conditions: XPhos Pd G3 (2.0 mol%), dicarbonyl compound (0.15 mmol, 1.5 equiv), bromobenzene (0.1 mmol, 1.0 equiv), LiOH (0.15 mmol, 1.5 equiv), $FeCl_3$ (0.01 mmol, 0.1 eq) molecular sieves 4Å (80 mg), DMF (0.5 mL), DCE (0.5 mL); [b]determined by GC with mesitylene as an internal standard.

Reaction procedures

Synthesis of substituted cyclopentenes

General notice. The reactions set up in a glovebox can also be performed using standard techniques of work under inert atmosphere (e.g. Schlenk techniques).

Conditions A: In a glovebox, to a 4-mL glass screw-capped vial containing XPhos Pd G3 (6.76 mg, 8.0 μmol) and magnetic stirring bar following reagents were added in exact order: LiOH (14.4 mg, 0.60 mmol), $FeCl_3$ (6.48 mg, 0.04 mmol), blend molecular sieves 4 Å (320 mg), DMF (1 mL), DCE (1 mL). Content of the vial was briefly stirred followed by addition of aryl bromide (0.40 mmol) and dicarbonyl compound (0.60 mmol). Then, the vial was sealed with a cap containing a PTFE septum and removed from glovebox. The reaction mixture was stirred at 50°C in the heating block for 24 h. After that time, vial was opened in air atmosphere, Then, mixture was diluted with DCM (50 mL) and transferred to conical flask containing large stirring bar. Solid NH_4Cl was added and mixture was stirred for 10 minutes, then solid Na_2SO_4 was added and mixture was stirred again for 10 minutes in order to dry it. After that, mixture was filtered through celite pad with the addition of DCM (50 mL), solvent was evaporated and residue purified on chromatography column containing silica gel.

Procedure for reaction run at 1 mmol scale for compounds 32 and 33: In a glovebox, to a 10-mL vacuum dried schlenk flask containing XPhos Pd G3 (16.93 mg, 20.0 μmol) and magnetic stirring bar following reagents were added in exact order: LiOH (36.0 mg, 1.50 mmol), $FeCl_3$ (16.2 mg, 0.1 mmol), blend molecular sieves 4 Å (800 mg), DMF (2.5 mL), DCE (2.5 mL). Content of the vial was briefly stirred followed by addition of bromobenzene (157.0 mg, 1.0 mmol) and dicarbonyl compound (1.50 mmol). Then, the schlenk flask was sealed with a septum and removed from glovebox. The reaction mixture was stirred at 50°C in the heating block for 24 h. After that time, flask was opened in air atmosphere, Then, mixture was diluted with DCM (50 mL) and transferred to conical flask containing large stirring bar. Solid NH_4Cl was added and mixture was stirred for 10 minutes, then solid Na_2SO_4 was added and mixture was stirred again for 10 minutes in order to dry it. After that, mixture was filtered through celite pad with the addition of DCM (50 mL), solvent was evaporated and residue purified on chromatography column containing silica gel.

Conditions B: In a glovebox, to a 4-mL glass screw-capped vial containing XPhos Pd G3 (6.76 mg, 8.0 μmol) and magnetic stirring bar following reagents were added in exact order: LiOH (14.4 mg, 0.60 mmol), blend molecular sieves 4 Å (320 mg), DCE (2 mL). Content of the vial was briefly stirred followed by addition of aryl bromide (0.40 mmol) and dicarbonyl compound (0.60 mmol). Then, the vial was sealed with a cap containing a PTFE septum and removed from glovebox. The reaction mixture was stirred at 50°C in the heating block for 24 h. After that time, vial was opened in air atmosphere, Then, mixture was diluted with DCM (50 mL) and transferred to conical flask containing large stirring bar. Solid NH_4Cl was added and mixture was stirred for 10 minutes, then solid Na_2SO_4 was added and mixture was stirred again for 10 minutes in order to dry it. After that, mixture was filtered through celite pad with the addition of DCM (50 mL), solvent was evaporated and residue purified on chromatography column containing silica gel.

General procedures substrate synthesis

Conditions C: A 50-mL Schlenk tube containing 60% NaH (816.0 mg, 20.4 mmol) and stirring bar was evacuated and backfilled with argon three times. Then, THF (60 ml) was added and mixture was cooled down to 0 °C. Then dicarbonyl compound (20.0 mmol) was slowly added. Resulting mixture was stirred for approximately 30 minutes, until hydrogen bubbles were not visible. Then alkyne iodide (20.4 mmol) was added. Reaction mixture was heated to 60°C in the heating block, stirred for 72h, and then cooled to room temperature. The mixture was quenched with NH_4Cl solution (30 ml) and water (10 ml) ,

extracted with MTBE (3x30 ml), dried (Na_2SO_4), concentrated and crude product was purified by column chromatography on silica gel.

Conditions D: A 50-mL Schlenk tube containing K_2CO_3 (1.29 g, 9.3 mmol) and stirring bar was evacuated and backfilled with argon three times. Then, acetone (50 ml) was added, followed by dicarbonyl compound (10.0 mmol). Resulting mixture was stirred for 10 minutes. Then alkyne iodide (9.5 mmol) was added. Reaction was heated to 60°C in the heating block, stirred for 72h, then cooled to room temperature. The mixture was filtered through celite and concentrated. Crude product was purified by column chromatography on silica gel.

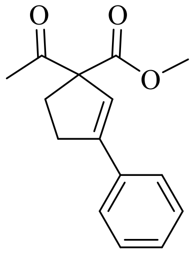

(2) methyl 1-acetyl-3-phenylcyclopent-2-ene-1-carboxylate Prepared in reaction of methyl 2-acetylhex-5-ynoate (100.9 mg, 0.6 mmol) with bromobenzene (62.8 mg, 0.4 mmol) under **conditions A**. The title compound was isolated as yellowish solid (81%, 79.0 mg, 0.32 mmol) after chromatography on silica gel (15g column, Hexane:Etyl Acetate 9:1) ^{1}HNMR (400 MHz, $CDCl_3$) 7.47 (d, 2H), 7.36-7.28 (m, 3H), 6.26 (t, J=1.8 Hz, 1H), 3.76 (s, 3H), 2.89-2.84 (m, 2H), 2.64-2.50 (m, 2H), 2.23 (s, 3H); ^{13}C NMR (101 MHz, $CDCl_3$) 203.6, 172.1, 147.5, 134.9, 128.5, 128.3, 126.1, 122.6, 73.8, 52.6, 32.4, 30.2, 26.5; IR (CH_2Cl_2): 1740 (C=O), 1713 (C=O), 1233 (C-O), cm^{-1}; HRMS (EI-EBE) m/z: $[M]^+$ Calcd for $C_{15}H_{16}O_3$: 244.1099; Found 244.1101

Additionally prepared in reaction of methyl 2-acetylhex-5-ynoate (100.9 mg, 0.6 mmol) with Phenyl trifluoromethanesulfonate (90.5 mg, 0.4 mmol) under **conditions B**. The title compound was isolated as yellowish solid (92%, 90.1 mg, 0.37 mmol) after chromatography on silica gel (15g column, Hexane:Etyl Acetate 9:1)

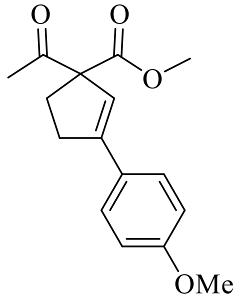

(4) methyl 1-acetyl-3-(4-methoxyphenyl)cyclopent-2-ene-1-carboxylate Prepared in reaction of methyl 2-acetylhex-5-ynoate (100.9 mg, 0.6 mmol) with 1-bromo-4-methoxybenzene (74.8 mg, 0.4 mmol) under **conditions A**. The title compound was isolated as yellowish solid (51%, 56.0 mg, 0.20 mmol) after chromatography on silica gel (15g column, Hexane:Etyl Acetate 9:1) ^{1}H NMR (400 MHz, $CDCl_3$) 7.44 – 7.38 (m, 2H), 6.89 – 6.84 (m, 2H), 6.12 (t, J = 1.8 Hz, 1H), 3.81 (s, 3H), 3.75 (s, 3H), 2.91 – 2.76 (m, 2H), 2.62 – 2.47 (m, 2H), 2.22 (s, 3H); ^{13}C NMR (101 MHz, $CDCl_3$) 203.8, 172.3, 159.7, 146.9, 127.7, 127.4, 120.4, 113.8, 73.7, 55.2, 52.5, 32.5, 30.2, 26.4.; IR (CH_2Cl_2): 1729 (C=O), 1713 (C=O), 1256 (C-O) cm^{-1}; HRMS (EI-EBE) m/z: $[M]^+$ Calcd for $C_{16}H_{18}O_4$: 274.1205; Found 274.1207

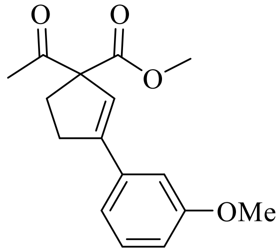

(5) methyl 1-acetyl-3-(3-methoxyphenyl)cyclopent-2-ene-1-carboxylate Prepared in reaction of methyl 2-acetylhex-5-ynoate (100.9 mg, 0.6 mmol) with 1-bromo-3-methoxybenzene (74.8 mg, 0.4 mmol) under **conditions A**. The title compound was isolated as transparent oil (70%, 76.6 mg, 0.28 mmol) after chromatography on silica gel (15g column, Hexane:Etyl Acetate 95:5) ^{1}H NMR (400 MHz, $CDCl_3$) 7.25 (t, J = 8.0 Hz, 1H), 7.09 – 7.04 (m, 1H), 7.02 – 6.98 (m, 1H), 6.86 – 6.82 (m, 1H), 6.25 (t, J = 1.8 Hz, 1H), 3.81 (s, 3H), 3.75 (s, 3H), 2.92 – 2.76 (m, 2H), 2.64 – 2.48 (m, 2H), 2.22 (s, 3H); ^{13}C NMR (101 MHz, $CDCl_3$) 203.4, 172.0, 159.6, 147.3, 136.3, 129.4, 122.9, 118.6, 113.7, 111.7, 73.7, 55.2, 52.6, 32.5, 30.1, 26.5; IR (CH_2Cl_2): 1740 (C=O), 1713 (C=O), 1239 (C-O) cm^{-1}; HRMS (EI-EBE) m/z: $[M]^+$ Calcd for $C_{16}H_{18}O_4$: 274.1205; Found 274.1208

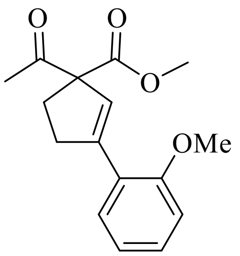

(6) methyl 1-acetyl-3-(2-methoxyphenyl)cyclopent-2-ene-1-carboxylate Prepared in reaction of methyl 2-acetylhex-5-ynoate (100.9 mg, 0.6 mmol) with 1-bromo-2-methoxybenzene (74.8 mg, 0.4 mmol) under **conditions A**. The title compound was isolated as orange oil (85%, 93.3 mg, 0.34 mmol) after chromatography on silica gel (15g column, Hexane:Etyl Acetate 95:5) ^{1}H NMR (400 MHz, $CDCl_3$) 7.32 – 7.21 (m, 2H), 6.96 – 6.87 (m, 2H), 6.54 (t, J = 1.7 Hz, 1H), 3.87 (s, 3H), 3.75 (s, 3H), 2.98 – 2.82 (m, 2H), 2.55 – 2.43 (m, 2H), 2.24 (s, 3H); ^{13}C NMR (101 MHz, $CDCl_3$) 204.0, 172.3, 157.8, 144.1, 129.0, 128.9, 126.6, 124.2, 120.3, 110.9, 74.2, 55.2, 52.4, 34.3, 29.6, 26.5; IR (CH_2Cl_2): 1740 (C=O), 1713 (C=O), 1244 (C-O) cm^{-1}; HRMS (EI-EBE) m/z: [M]$^+$ Calcd for $C_{16}H_{18}O_4$: 274.1205; Found 274.1211

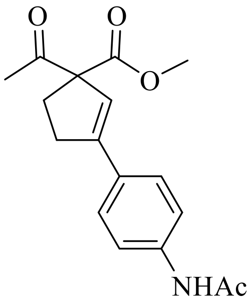

(7) methyl 3-(4-acetamidophenyl)-1-acetylcyclopent-2-ene-1-carboxylate Prepared in reaction of methyl 2-acetylhex-5-ynoate (100.9 mg, 0.6 mmol) with N-(4-bromophenyl)acetamide (85.6 mg, 0.4 mmol) under **conditions A**. The title compound was isolated as orange oil (57%, 69.1 mg, 0.23 mmol) after chromatography on silica gel (15g column, Hexane:Etyl Acetate 8:2) ^{1}H NMR (400 MHz, $CDCl_3$) 8.00 (s, 1H), 7.49 (d, J = 8.6 Hz, 2H), 7.35 (d, J = 8.6 Hz, 2H), 6.14 (br s, 1H), 3.73 (s, 3H), 2.86 – 2.71 (m, 2H), 2.61 – 2.45 (m, 2H), 2.21 (s, 3H), 2.14 (s, 3H); ^{13}C NMR (101 MHz, $CDCl_3$) 204.0, 172.2, 168.6, 146.9, 138.2, 130.6, 126.7, 121.4, 119.5, 73.7, 52.6, 32.3, 30.1, 26.5, 24.4; IR (CH_2Cl_2): 1738 (C=O), 1712 (C=O), 1233 (C-O) cm^{-1}; HRMS (EI-EBE) m/z: [M]$^+$ Calcd for $C_{17}H_{19}NO_4$: 301.1314; Found 301.1315

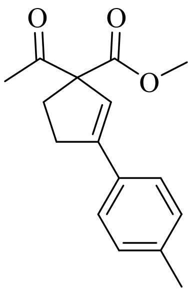

(8) methyl 1-acetyl-3-(p-tolyl)cyclopent-2-ene-1-carboxylate Prepared in reaction of methyl 2-acetylhex-5-ynoate (100.9 mg, 0.6 mmol) with 1-bromo-4-methylbenzene (68.4 mg, 0.4 mmol) under **conditions A**. The title compound was isolated as orange oil (56%, 57.9 mg, 0.22 mmol) after chromatography on silica gel (15g column, Hexane:Etyl Acetate 95:5) ^{1}H NMR (400 MHz, $CDCl_3$) 7.37 (d, J = 8.2 Hz, 2H), 7.18 – 7.12 (m, 2H), 6.20 (t, J = 1.9 Hz, 1H), 3.75 (s, 3H), 2.93 – 2.77 (m, 2H), 2.64 – 2.48 (m, 2H), 2.35 (s, 3H), 2.22 (s, 3H); ^{13}C NMR (101 MHz, $CDCl_3$) 203.7, 172.2, 147.4, 138.2, 132.1, 129.1, 126.0, 121.5, 73.7, 52.5, 32.4, 30.2, 26.5, 21.2; IR (CH_2Cl_2): 1740 (C=O), 1713 (C=O), 1232 (C-O) cm^{-1}; HRMS (EI-EBE) m/z: [M]$^+$ Calcd for $C_{16}H_{18}O_3$: 258.1260; Found 258.1256

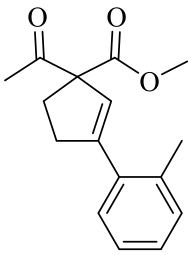

(9) methyl 1-acetyl-3-(o-tolyl)cyclopent-2-ene-1-carboxylate Prepared in reaction of methyl 2-acetylhex-5-ynoate (100.9 mg, 0.6 mmol) with 1-bromo-2-methylbenzene (68.4 mg, 0.4 mmol) under **conditions A**. The title compound was isolated as yellowish oil (64%, 66.6 mg, 0.26 mmol) after chromatography on silica gel (15g column, Hexane:Etyl Acetate 95:5) ^{1}H NMR (400 MHz, $CDCl_3$) 7.22 – 7.16 (m, 4H), 5.89 (t, J = 1.9 Hz, 1H), 3.77 (s, 3H), 2.89 – 2.75 (m, 2H), 2.62 – 2.48 (m, 2H), 2.38 (s, 3H), 2.25 (s, 3H); ^{13}C NMR (101 MHz, $CDCl_3$) 203.5, 172.2, 148.5, 136.0, 135.5, 130.6, 127.9, 127.6, 126.3, 125.6, 74.0, 52.5, 35.8, 30.3, 26.5, 21.0; IR (CH_2Cl_2): 1742 (C=O), 1714 (C=O), 1232 (C-O) cm^{-1}; HRMS (EI-EBE) m/z: [M]$^+$ Calcd for $C_{16}H_{18}O_3$: 258.1260; Found 258.1256

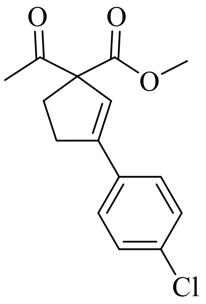

(10) methyl 1-acetyl-3-(4-chlorophenyl)cyclopent-2-ene-1-carboxylate Prepared in reaction of methyl 2-acetylhex-5-ynoate (100.9 mg, 0.6 mmol) with 1-bromo-4-chlorobenzene (76.6 mg, 0.4 mmol) under **conditions A**. The title compound was isolated as yellowish oil (60%, 66.8 mg, 0.24 mmol) after chromatography on silica gel (15g column, Hexane:Etyl Acetate 9:1) ^{1}H NMR (400 MHz, $CDCl_3$) 7.41 – 7.36 (m, 2H), 7.32 – 7.28 (m, 2H), 6.24 (t, J = 1.7 Hz, 1H), 3.76 (s, 3H), 2.91 – 2.75 (m, 2H), 2.64 – 2.49 (m, 2H), 2.22 (s, 3H); ^{13}C NMR (101 MHz, $CDCl_3$) 203.2, 172.0, 146.3, 134.1, 133.4, 128.6, 127.3, 123.2, 73.8, 52.7, 32.4, 30.2, 26.5; IR (CH_2Cl_2): 1740 (C=O), 1713 (C=O), 1232 (C-O) cm^{-1}; HRMS (EI-EBE) m/z: [M]$^+$ Calcd for $C_{15}H_{15}O_3Cl$: 278.0710; Found 278.0713

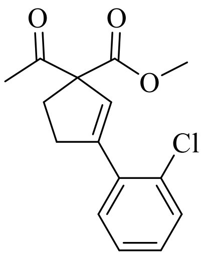

(11) methyl 1-acetyl-3-(2-chlorophenyl)cyclopent-2-ene-1-carboxylate Prepared in reaction of methyl 2-acetylhex-5-ynoate (100.9 mg, 0.6 mmol) with 1-bromo-2-chlorobenzene (76.6 mg, 0.4 mmol) under **conditions A**. The title compound was isolated as transparent oil (79%, 88.3 mg, 0.32 mmol) after chromatography on silica gel (15g column, Hexane:Etyl Acetate 8:2) ^{1}H NMR (400 MHz, $CDCl_3$) 7.41 – 7.35 (m, 1H), 7.30 – 7.27 (m, 1H), 7.25 – 7.18 (m, 2H), 6.21 (t, *J* = 1.9 Hz, 1H), 3.77 (s, 3H), 2.96 – 2.82 (m, 2H), 2.64 – 2.48 (m, 2H), 2.26 (s, 3H); ^{13}C NMR (101 MHz, $CDCl_3$) 203.5, 171.9, 146.3, 135.1, 132.5, 130.2, 129.7, 128.9, 128.3, 126.6, 73.9, 52.6, 35.1, 30.4, 26.6; IR (CH_2Cl_2): 1742 (C=O), 1714 (C=O), 1230 (C-O) cm^{-1}; HRMS (EI-EBE) m/z: $[M]^+$ Calcd for $C_{15}H_{15}O_3Cl$: 278.0710; Found 278.0712

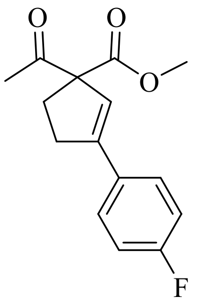

(12) methyl 1-acetyl-3-(4-fluorophenyl)cyclopent-2-ene-1-carboxylate Prepared in reaction of methyl 2-acetylhex-5-ynoate (100.9 mg, 0.6 mmol) with 1-bromo-4-fluorobenzene (70.0 mg, 0.4 mmol) under **conditions A**. The title compound was isolated as yellowish solid (75%, 78.8 mg, 0.30 mmol) after chromatography on silica gel (15g column, Hexane:Etyl Acetate 9:1) ^{1}H NMR (400 MHz, $CDCl_3$) 7.46-7.40 (m, 2H), 7.05 – 6.98 (m, 2H), 6.18 (t, *J* = 1.8 Hz, 1H), 3.75 (s, 3H), 2.90 – 2.75 (m, 2H), 2.63 – 2.48 (m, 2H), 2.22 (s, 3H); ^{13}C NMR (101 MHz, $CDCl_3$) 203.3, 172.0, 162.7 (d, *J* = 249.1 Hz), 131.1 (d, *J* = 3.4 Hz), 127.8 (d, *J* = 8.4 Hz), 122.2 (d, *J* = 1.9 Hz), 115.4, 115.2, 73.7, 52.6, 32.6, 30.2, 26.5; IR (CH_2Cl_2): 1741 (C=O), 1714 (C=O), 1234 (C-O) cm^{-1}; HRMS (ESI-TOF) m/z: $[M+Na]^+$Calcd for $C_{15}H_{15}O_3FNa$: 285.0908; Found 285.0903

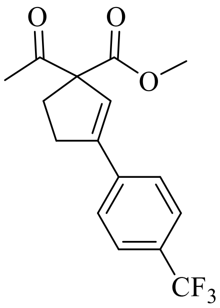

(13) methyl 1-acetyl-3-(4-(trifluoromethyl)phenyl)cyclopent-2-ene-1-carboxylate Prepared in reaction of methyl 2-acetylhex-5-ynoate (100.9 mg, 0.6 mmol) with 1-bromo-4-(trifluoromethyl)benzene (90.0 mg, 0.4 mmol) under **conditions A**. The title compound was isolated as yellowish solid (77%, 95.9 mg, 0.31 mmol) after chromatography on silica gel (15g column, Hexane:Etyl Acetate 95:5) ^{1}HNMR (400 MHz, $CDCl_3$) 7.57 (q, 4H), 6.36 (s, 1H), 3.76 (s, 3H), 2.93-2.80 (m, 2H), 2.66-2.52 (m, 2H), 2.23 (s, 3H); ^{13}C NMR (101 MHz, $CDCl_3$) 202.9, 171.8, 146.1, 138.3, 130.1 (q, *J* = 25.5 Hz), 126.3, 125.4 (q, *J* = 3.8 Hz), 125.11, 73.8, 52.7, 32.4, 30.2, 26.6; IR (CH_2Cl_2): 1742 (C=O), 1715 (C=O), 1236 (C-O) cm^{-1}; HRMS (ESI-TOF) m/z: $[M+Na]^+$ Calcd for $C_{16}H_{15}O_3F_3Na$: 335.0871; Found 335.0874

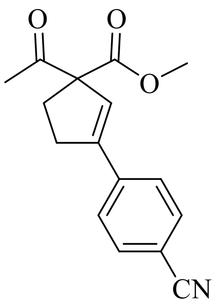

(14) methyl 1-acetyl-3-(4-cyanophenyl)cyclopent-2-ene-1-carboxylate Prepared in reaction of methyl 2-acetylhex-5-ynoate (100.9 mg, 0.6 mmol) with 4-bromobenzonitrile (72.8 mg, 0.4 mmol) under **conditions A**. The title compound was isolated as yellowish solid (64%, 68.5 mg, 0.25 mmol) after chromatography on silica gel (15g column, Hexane:Etyl Acetate 8:2) ^{1}H NMR (400 MHz, $CDCl_3$) 7.63 – 7.58 (m, 2H), 7.55 – 7.50 (m, 2H), 6.39 (t, *J* = 1.8 Hz, 1H), 3.76 (s, 3H), 2.91 – 2.76 (m, 2H), 2.67 – 2.49 (m, 2H), 2.22 (s, 3H); ^{13}C NMR (101 MHz, $CDCl_3$) 202.6, 171.5, 145.7, 139.2, 132.2, 126.6, 118.6, 116.6, 111.6, 73.8, 52.8, 32.2, 30.1, 26.6; IR (CH_2Cl_2): 2226 (C≡N), 1738 (C=O), 1712 (C=O), 1235 (C-O) cm^{-1}; HRMS (ESI-TOF) m/z: $[M+Na]^+$ calc'd for $C_{16}H_{15}NO_3Na$: 292.0950; found 292.0953

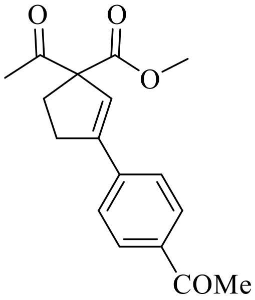

(15) methyl 1-acetyl-3-(4-acetylphenyl)cyclopent-2-ene-1-carboxylate Prepared in reaction of methyl 2-acetylhex-5-ynoate (100.9 mg, 0.6 mmol) with 1-(4-bromophenyl)ethan-1-one (79.6 mg, 0.4 mmol) under **conditions A**. The title compound was isolated as yellowish oil (80%, 91.6 mg, 0.32 mmol) after chromatography on silica gel (15g column, Hexane:Etyl Acetate 8:2) ^{1}H NMR (400 MHz, $CDCl_3$) 7.94 – 7.87 (m, 2H), 7.55 – 7.49 (m, 2H), 6.37 (t, J = 1.8 Hz, 1H), 3.75 (s, 3H), 2.92 – 2.78 (m, 2H), 2.64 – 2.49 (m, 2H), 2.57 (s, 3H), 2.22 (s, 3H); ^{13}C NMR (101 MHz, $CDCl_3$) 202.9, 197.3, 171.7, 146.4, 139.3, 136.5, 128.5, 126.2, 125.3, 73.8, 52.7, 32.3, 30.1, 26.5, 26.5; IR (CH_2Cl_2): 1739 (C=O), 1713 (C=O), 1234 (C-O) cm^{-1}; HRMS (ESI-TOF) m/z: $[M+Na]^+$ Calcd for $C_{17}H_{18}O_4Na$: 309.1103; Found 309.1108

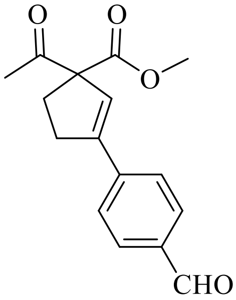

(16) methyl 1-acetyl-3-(4-formylphenyl)cyclopent-2-ene-1-carboxylate Prepared in reaction of methyl 2-acetylhex-5-ynoate (100.9 mg, 0.6 mmol) with 4-bromobenzaldehyde (74.0 mg, 0.4 mmol) under **conditions A**. The title compound was isolated as transparent oil (36%, 38.7 mg, 0.14 mmol) after chromatography on silica gel (15g column, Hexane:Etyl Acetate 9:1) ^{1}H NMR (400 MHz, $CDCl_3$) 9.98 (s, 1H), 7.84 (d, J = 8.4 Hz, 2H), 7.60 (d, J = 8.3 Hz, 2H), 6.42 (t, J = 1.9 Hz, 1H), 3.76 (s, 3H), 2.96 – 2.80 (m, 2H), 2.68 – 2.50 (m, 2H), 2.23 (s, 3H); ^{13}C NMR (101 MHz, $CDCl_3$) 202.8, 191.5, 171.7, 146.4, 140.7, 135.9, 129.9, 126.6, 126.1, 73.9, 52.8, 32.4, 30.2, 26.6;IR (CH_2Cl_2): 1740 (C=O), 1713 (C=O), 1700 (C=O), 1236 (C-O) cm^{-1}; HRMS (EI-EBE) m/z: $[M]^+$ Calcd for $C_{16}H_{16}O_4$: 272.1049; Found 272.1054

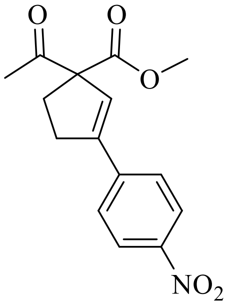

(17) methyl 1-acetyl-3-(4-nitrophenyl)cyclopent-2-ene-1-carboxylate Prepared in reaction of methyl 2-acetylhex-5-ynoate (100.9 mg, 0.6 mmol) with 4-bromonitrobenzene (80.8 mg, 0.4 mmol) under **conditions A**. The title compound was isolated as beige solid (68%, 79.2 mg, 0.27 mmol) after chromatography on silica gel (15g column, Hexane:Etyl Acetate 9:1) ^{1}H NMR (400 MHz, $CDCl_3$) 8.16 (d, J = 8.9 Hz, 2H), 7.58 (d, J = 8.9 Hz, 2H), 6.49 – 6.41 (m, 1H), 3.76 (s, 3H), 2.95 – 2.79 (m, 2H), 2.68 – 2.50 (m, 2H), 2.23 (s, 3H); ^{13}C NMR (101 MHz, $CDCl_3$) 202.5, 171.5, 147.3, 145.4, 141.2, 127.3, 126.8, 123.8, 73.9, 52.8, 32.4, 30.2, 26.6; IR (CH_2Cl_2): 1740 (C=O), 1713 (C=O), 1700 (C=O), 1236 (C-O) cm^{-1}; HRMS (EI-EBE) m/z: $[M]^+$ Calcd for $C_{15}H_{15}NO_5$: 289.0950; Found 289.0956

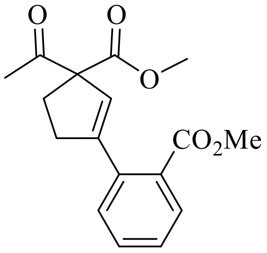

(18) methyl 2-(3-acetyl-3-(methoxycarbonyl)cyclopent-1-en-1-yl)benzoate Prepared in reaction of methyl 2-acetylhex-5-ynoate (100.9 mg, 0.6 mmol) with methyl 2-bromobenzoate (85.6 mg, 0.4 mmol) under **conditions A**. The title compound was isolated as yellowish oil (65%, 78.2 mg, 0.26 mmol) after chromatography on silica gel (15g column, Hexane:Etyl Acetate 8:2) ^{1}H NMR (400 MHz, $CDCl_3$) 7.81 – 7.75 (m, 1H), 7.45 (td, J = 7.6, 1.3 Hz, 1H), 7.34 (td, J = 7.6, 1.2 Hz, 1H), 7.29 – 7.23 (m, 1H), 5.77 (s, 1H), 3.83 (s, 3H), 3.76 (s, 3H), 2.83 – 2.69 (m, 2H), 2.64 – 2.49 (m, 2H), 2.25 (s, 3H); ^{13}C NMR (101 MHz, $CDCl_3$) 203.8, 172.1, 168.2, 149.5, 137.7, 131.5, 130.0, 129.8, 129.2, 127.7, 125.4, 73.9, 52.5, 52.2, 35.9, 31.0, 26.6; IR (CH_2Cl_2): 1717 (C=O), 1235 (C-O), cm^{-1}; HRMS (EI-EBE) m/z: $[M]^+$ Calcd for $C_{17}H_{18}O_5$: 302.1154; Found 302.1144

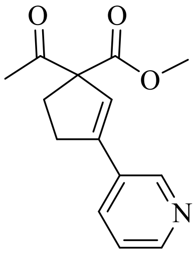

(19) methyl 1-acetyl-3-(pyridin-3-yl)cyclopent-2-ene-1-carboxylate Prepared in reaction of methyl 2-acetylhex-5-ynoate (100.9 mg, 0.6 mmol) with 3-bromopirydine (63.2 mg, 0.4 mmol) under **conditions A**. The title compound was isolated as yellow oil (78%, 76.4 mg, 0.31 mmol) after chromatography on silica gel (15g column, Hexane:Etyl Acetate 1:1) ^{1}H NMR (400 MHz, $CDCl_3$) 8.68 (d, J = 1.8 Hz, 1H), 8.48 (dd, J = 4.8, 1.5 Hz, 1H), 7.71 (dt, J = 8.0, 1.9 Hz, 1H), 7.26 – 7.20 (m, 1H), 6.31 (t, J = 1.9 Hz, 1H), 3.73 (s, 3H), 2.89 – 2.76 (m, 2H), 2.63 – 2.48 (m,

2H), 2.20 (s, 3H); ^{13}C NMR (101 MHz, $CDCl_3$) 202.8, 171.7, 149.2, 147.4, 144.4, 133.1, 130.5, 124.4, 123.2, 73.7, 52.7, 32.1, 30.1, 26.5; IR (CH_2Cl_2): 1739 (C=O), 1713 (C=O), 1244 (C-O) cm^{-1}; HRMS (EI-EBE) m/z: $[M]^+$ Calcd for $C_{14}H_{15}NO_3$: 245.1052; Found 245.1060

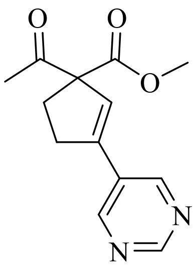

(20) methyl 1-acetyl-3-(pyrimidin-5-yl)cyclopent-2-ene-1-carboxylate Prepared in reaction of methyl 2-acetylhex-5-ynoate (100.9 mg, 0.6 mmol) with 5-bromopyrimidine (63.6 mg, 0.4 mmol) under **conditions A**. The title compound was isolated as orange solid (74%, 73.2 mg, 0.30 mmol) after chromatography on silica gel (15g column, Hexane:Dioxane 7:3) ^1H NMR (400 MHz, $CDCl_3$) 9.06 (s, 1H), 8.77 (s, 2H), 6.41 (t, J = 1.8 Hz, 1H), 3.73 (s, 3H), 2.90 – 2.75 (m, 2H), 2.65 – 2.48 (m, 2H), 2.20 (s, 3H); ^{13}C NMR (101 MHz, $CDCl_3$) δ 202.3, 171.4, 157.8, 154.0, 141.1, 128.6, 126.4, 73.9, 52.9, 31.8, 30.1, 26.6; IR (CH_2Cl_2): 1739 (C=O), 1713 (C=O), 1244 (C-O) cm^{-1}; HRMS (EI-EBE) m/z: $[M]^+$ Calcd for $C_{13}H_{14}N_2O_3$: 246.1004; Found 246.1004

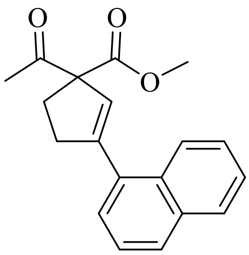

(21) methyl 1-acetyl-3-(naphthalen-1-yl)cyclopent-2-ene-1-carboxylate Prepared in reaction of methyl 2-acetylhex-5-ynoate (100.9 mg, 0.6 mmol) with 1-bromonaphthalene (82.8 mg, 0.4 mmol) under **conditions A**. The title compound was isolated as off-white solid (67%, 79.2 mg, 0.27 mmol) after chromatography on silica gel (15g column, Hexane:Etyl Acetate 9:1) ^{1}HNMR (400 MHz, $CDCl_3$) 8.15 – 8.10 (m, 1H), 7.87-7.84 (m, 1H), 7.78 (d, J=8,2 Hz, 1H), 7.53-7.41 (m, 3H), 7.37 (dd, J=7.2 Hz, 1.2 Hz, 1H), 6.07 (t, J=1.8 Hz, 1H), 3.81 (s, 3H), 2.99-2.92 (m, 2H), 2.66 (t, 2H), 2.30 (s, 3H); ^{13}C NMR (101 MHz, $CDCl_3$) 203.4, 172.3, 147.7, 134.8, 133.8, 131.1, 128.5, 128.1, 127.5, 126.3, 125.9, 125.3, 125.1, 125.0, 74.2, 52.7, 36.7, 30.6, 26.7; IR (CH_2Cl_2): 1741 (C=O), 1713 (C=O), 1235 (C-O) cm^{-1}; HRMS (EI-EBE) m/z: $[M]^+$ Calcd for $C_{19}H_{18}O_3$: 294.1256; Found 294.1258

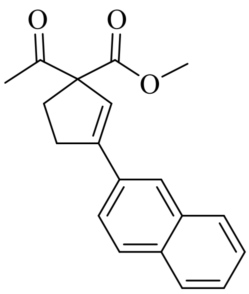

(22) methyl 1-acetyl-3-(naphthalen-2-yl)cyclopent-2-ene-1-carboxylate Prepared in reaction of methyl 2-acetylhex-5-ynoate (100.9 mg, 0.6 mmol) with 2-bromonaphthalene (82.8 mg, 0.4 mmol) under **conditions A**. The title compound was isolated as off-white solid (76%, 89.7 mg, 0.30 mmol) after chromatography on silica gel (15g column, Hexane:Etyl Acetate 9:1) ^1H NMR (400 MHz, $CDCl_3$) 7.85 – 7.77 (m, 4H), 7.72 – 7.68 (m, 1H), 7.50 – 7.44 (m, 2H), 6.41 (t, J = 1.6 Hz, 1H), 3.78 (s, 3H), 3.07 – 2.92 (m, 2H), 2.71 – 2.55 (m, 2H), 2.27 (s, 3H); ^{13}C NMR (101 MHz, $CDCl_3$) 203.5, 172.2, 147.4, 133.3, 133.2, 132.3, 128.3, 128.0, 127.6, 126.4, 126.3, 125.3, 124.0, 123.2, 73.9, 52.7, 32.5, 30.3, 26.6; IR (CH_2Cl_2): 1739 (C=O), 1712 (C=O), 1244 (C-O) cm^{-1}; HRMS (EI-EBE) m/z: $[M]^+$ Calcd for $C_{19}H_{18}O_3$: 294.1256; Found 294.1264

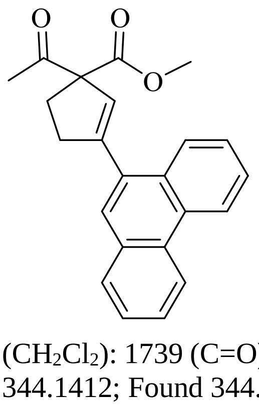

(23) methyl 1-acetyl-3-(phenanthren-9-yl)cyclopent-2-ene-1-carboxylate Prepared in reaction of methyl 2-acetylhex-5-ynoate (100.9 mg, 0.6 mmol) with 9-bromophenanthrene (102.9 mg, 0.4 mmol) under **conditions A**. The title compound was isolated as orange oil (32%, 44.5 mg, 0.13 mmol) after chromatography on silica gel (15g column, Hexane:Etyl Acetate 95:5) ^{1}HNMR (400 MHz, $CDCl_3$) 8.70 (dd, J = 26.3, 8.0 Hz, 2H), 8.14 (d, J = 7.9 Hz, 1H), 7.86 (d, J = 7.6 Hz, 1H), 7.71 – 7.55 (m, 5H), 6.13 (t, J = 1.7 Hz, 1H), 3.84 (s, 3H), 3.05 – 2.97 (m, 2H), 2.71 (t, J = 7.1 Hz, 2H), 2.33 (s, 3H); ^{13}C NMR (101 MHz, $CDCl_3$) 203.3, 148.1, 133.5, 131.2, 130.6, 130.2, 130.0, 128.5, 127.7, 126.8, 126.8, 126.7, 126.6, 126.1, 125.7, 123.0, 122.5, 74.1, 52.7, 36.8, 30.6, 26.7; IR (CH_2Cl_2): 1739 (C=O), 1712 (C=O), 1227 (C-O) cm^{-1}; HRMS (EI-EBE) m/z: $[M]^+$ Calcd for $C_{23}H_{20}O_3$: 344.1412; Found 344.1407

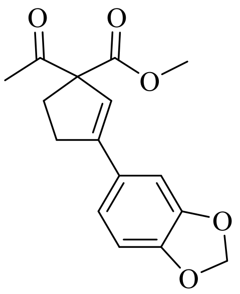

(24) methyl 1-acetyl-3-(benzo[d][1,3]dioxol-5-yl)cyclopent-2-ene-1-carboxylate Prepared in reaction of methyl 2-acetylhex-5-ynoate (100.9 mg, 0.6 mmol) with 5-bromobenzo[d][1,3]dioxole (80.4 mg, 0.4 mmol) under **conditions A**. The title compound was isolated as yellowish oil (55%, 64.0 mg, 0.22 mmol) after chromatography on silica gel (15g column, Hexane:Etyl Acetate 9:1) ^{1}HNMR (400 MHz, $CDCl_3$) 6.98 (d, *J* = 1.7 Hz, 1H), 6.91 (dd, *J* = 8.1, 1.7 Hz, 1H), 6.76 (d, *J* = 8.1 Hz, 1H), 6.09 (t, *J* = 1.7 Hz, 1H), 5.94 (s, 2H), 3.74 (s, 3H), 2.86 – 2.71 (m, 2H), 2.60 – 2.45 (m, 2H), 2.21 (s, 3H); ^{13}C NMR (101 MHz, $CDCl_3$) 203.6, 172.2, 147.8, 147.7, 146.9, 129.3, 121.1, 120.1, 108.0, 106.3, 101.1, 73.6, 52.5, 32.6, 30.1, 26.4; IR (CH_2Cl_2): 1739 (C=O), 1713 (C=O), 1227 (C-O) cm^{-1}; HRMS (EI-EBE) m/z: [M]$^+$ Calcd for $C_{16}H_{16}O_5$: 288.0998; Found 288.0990

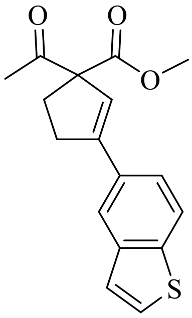

(25) methyl 1-acetyl-3-(benzo[b]thiophen-5-yl)cyclopent-2-ene-1-carboxylate Prepared in reaction of methyl 2-acetylhex-5-ynoate (100.9 mg, 0.6 mmol) with 5-bromobenzo[b]thiophene (85.2 mg, 0.4 mmol) under **conditions A**. The title compound was isolated as off-white solid (78%, 93.2 mg, 0.31 mmol) after chromatography on silica gel (15g column, Hexane:Etyl Acetate 95:5) ^{1}HNMR (400 MHz, $CDCl_3$) 7.86 – 7.80 (m, 2H), 7.52 (dd, *J* = 8.5, 1.6 Hz, 1H), 7.44 (d, *J* = 5.4 Hz, 1H), 7.32 (d, *J* = 5.4 Hz, 1H), 6.32 (t, *J* = 1.7 Hz, 1H), 3.77 (s, 3H), 3.01 – 2.87 (m, 2H), 2.69 – 2.53 (m, 2H), 2.25 (s, 3H); ^{13}C NMR (101 MHz, $CDCl_3$) 203.6, 172.2, 147.5, 139.8, 139.6, 131.4, 127.1, 124.0, 122.5, 122.4, 121.1, 73.8, 52.6, 32.7, 30.3, 26.6; IR (CH_2Cl_2): 1739 (C=O), 1712 (C=O), 1232 (C-O) cm^{-1}; HRMS (EI-EBE) m/z: [M]$^+$ Calcd for $C_{17}H_{16}O_3S$: 300.0820; Found 300.0820

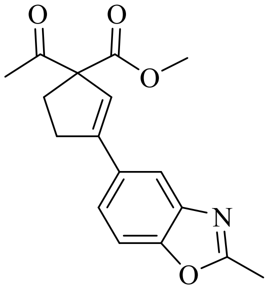

(26) methyl 1-acetyl-3-(2-methylbenzo[d]oxazol-5-yl)cyclopent-2-ene-1-carboxylate Prepared in reaction of methyl 2-acetylhex-5-ynoate (100.9 mg, 0.6 mmol) with 5-bromo-2-methylbenzo[d]oxazole (84.8 mg, 0.4 mmol) under **conditions A**. The title compound was isolated as transparent oil (62%, 74.1 mg, 0.25 mmol) after chromatography on silica gel (15g column, Hexane:Etyl Acetate 9:1) ^{1}HNMR (400 MHz, $CDCl_3$) 7.67 (d, *J* = 1.4 Hz, 1H), 7.44 (dd, *J* = 8.5, 1.7 Hz, 1H), 7.38 (d, *J* = 8.5 Hz, 1H), 6.23 (t, *J* = 1.7 Hz, 1H), 3.74 (s, 3H), 2.96 – 2.80 (m, 2H), 2.64 – 2.49 (m, 2H), 2.59 (s, 3H), 2.21 (s, 3H); ^{13}C NMR (101 MHz, $CDCl_3$) 203.4, 172.1, 164.4, 150.8, 147.1, 141.8, 131.5, 122.8, 122.2, 116.9, 109.9, 73.7, 52.5, 32.8, 30.1, 26.5, 14.4; IR (CH_2Cl_2): 1739 (C=O), 1713 (C=O), 1238 (C-O) cm^{-1}; HRMS (EI-EBE) m/z: [M]$^+$ Calcd for $C_{17}H_{17}NO_4$: 299.1158; Found 299.1162

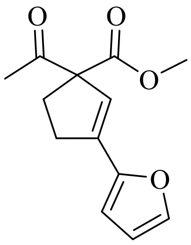

(27) methyl 1-acetyl-3-(furan-2-yl)cyclopent-2-ene-1-carboxylate Prepared in reaction of methyl 2-acetylhex-5-ynoate (100.9 mg, 0.6 mmol) with 2-bromofuran (58.8 mg, 0.4 mmol) under **conditions A**. The title compound was isolated as orange oil (45%, 42.0 mg, 0.18 mmol) after chromatography on silica gel (15g column, Hexane:Etyl Acetate 95:5) ^{1}HNMR (400 MHz, $CDCl_3$) 7.39 (d, *J* = 1.5 Hz, 1H), 6.38 (dd, *J* = 3.3, 1.8 Hz, 1H), 6.32 (d, *J* = 3.3 Hz, 1H), 6.15 (t, *J* = 1.9 Hz, 1H), 3.74 (s, 3H), 2.82 – 2.67 (m, 2H), 2.59 – 2.47 (m, 2H), 2.22 (s, 3H); ^{13}C NMR (101 MHz, $CDCl_3$) 203.3, 172.0, 150.6, 142.7, 137.3, 121.0, 111.2, 108.6, 73.7, 52.6, 31.4, 30.1, 26.6; IR (CH_2Cl_2): 1739 (C=O), 1713 (C=O), 1238 (C-O) cm^{-1}; HRMS (EI-EBE) m/z: [M]$^+$ Calcd for $C_{13}H_{14}O_4$: 234.0892; Found 234.0893

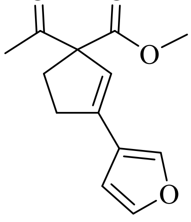

(28) methyl 1-acetyl-3-(furan-3-yl)cyclopent-2-ene-1-carboxylate Prepared in reaction of methyl 2-acetylhex-5-ynoate (100.9 mg, 0.6 mmol) with 2-bromofuran (58.8 mg, 0.4 mmol) under **conditions A**. The title compound was isolated as orange oil (63%, 59.5 mg, 0.25 mmol) after chromatography on silica gel (15g column, Hexane:Etyl Acetate 95:5) ^{1}HNMR (400 MHz, $CDCl_3$) 7.43 (s, 1H), 7.38 (t, *J* = 1.6 Hz, 1H), 6.56 – 6.52 (m, 1H), 5.96 (t, *J* = 1.6 Hz, 1H), 3.74 (s, 3H), 2.77 – 2.62 (m, 2H), 2.59 – 2.43 (m, 2H), 2.20 (s, 3H); ^{13}C NMR (101 MHz, $CDCl_3$) 203.6, 172.2, 143.6, 140.3, 139.4, 121.8, 108.4, 73.4, 52.6, 32.6, 30.3, 26.5; IR (CH_2Cl_2): 1739 (C=O),

1713 (C=O), 1245 (C-O) cm^{-1}; HRMS (EI-EBE) m/z: $[M]^+$ Calcd for $C_{13}H_{14}O_4$: 234.0892; Found 234.0890

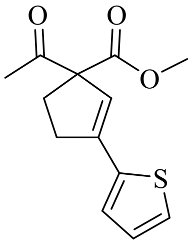

(29) methyl 1-acetyl-3-(thiophen-2-yl)cyclopent-2-ene-1-carboxylate Prepared in reaction of methyl 2-acetylhex-5-ynoate (100.9 mg, 0.6 mmol) with 2-bromotiophene (65.2 mg, 0.4 mmol) under **conditions A**. The title compound was isolated as orange oil (59%, 59.0 mg, 0.24 mmol) after chromatography on silica gel (15g column, Hexane:Dioxane 95:5) ^{1}HNMR (400 MHz, $CDCl_3$) 7.24 (dd, J = 5.1, 1.0 Hz, 1H), 7.04 (dd, J = 3.6, 1.0 Hz, 1H), 6.98 (dd, J = 5.0, 3.6 Hz, 1H), 6.08 (t, J = 1.8 Hz, 1H), 3.75 (s, 3H), 2.92 – 2.77 (m, 2H), 2.62 – 2.48 (m, 2H), 2.22 (s, 3H); ^{13}C NMR (101 MHz, $CDCl_3$) 203.3, 171.9, 141.4, 139.0, 127.4, 125.6, 125.4, 121.6, 73.7, 52.6, 33.3, 30.2, 26.5; IR (CH_2Cl_2): 1740 (C=O), 1713 (C=O), 1237 (C-O) cm^{-1}; HRMS (EI-EBE) m/z: $[M]^+$ Calcd for $C_{13}H_{14}O_3S$: 250.0659; Found 250.0659

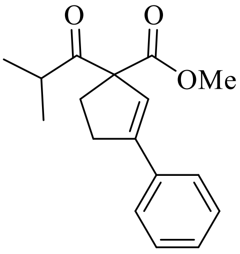

(30) methyl 1-isobutyryl-3-phenylcyclopent-2-ene-1-carboxylate Prepared in reaction of methyl 2-isobutyrylhex-5-ynoate (134.5 mg, 0.6 mmol) with bromobenzene (62.8 mg, 0.4 mmol) under **conditions A**. The title compound was isolated as yellowish oil (66%, 66.8 mg, 0.24 mmol) after chromatography on silica gel (15g column, Hexane:Ethyl Acetate 95:5) ^{1}H NMR (400 MHz, $CDCl_3$) 7.50 – 7.44 (m, 2H), 7.37 – 7.26 (m, 3H), 6.30 (t, J = 1.8 Hz, 1H), 3.75 (s, 3H), 2.97 (hept, J = 6.8 Hz, 1H), 2.92 – 2.78 (m, 2H), 2.61 – 2.55 (m, 2H), 1.10 (dd, J = 12.1, 6.7 Hz, 6H); ^{13}C NMR (101 MHz, $CDCl_3$) 210.1, 172.2, 147.4, 135.0, 128.4, 128.2, 126.0, 122.4, 73.7, 52.4, 37.4, 32.4, 30.2, 19.9, 19.7; IR (CH_2Cl_2): 1742 (C=O), 1712 (C=O), 1232 (C-O) cm^{-1}; HRMS (EI-EBE) m/z: $[M]^+$ Calcd for $C_{17}H_{20}O_3$: 272.1412; Found 272.1411

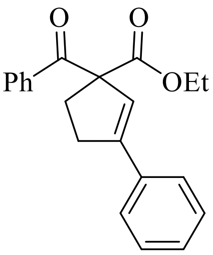

(31) ethyl 1-benzoyl-3-phenylcyclopent-2-ene-1-carboxylate Prepared in reaction of ethyl 2-benzoylhex-5-ynoate (146.6 mg, 0.6 mmol) with bromobenzene (62.8 mg, 0.4 mmol) under **conditions A**. The title compound was isolated as off-white solid (90%, 66.8 mg, 0.24 mmol) after chromatography on silica gel (15g column, Hexane:Ethyl Acetate 95:5) ^{1}H NMR (400 MHz, $CDCl_3$) 7.95 (d, J = 8.2 Hz, 1H), 7.59 – 7.41 (m, 4H), 7.36 – 7.22 (m, 2H), 6.36 (s, 1H), 4.19 – 4.07 (m, 2H), 3.06 – 2.95 (m, 1H), 2.94 – 2.82 (m, 2H), 2.72 – 2.63 (m, 1H), 1.08 (t, J = 7.1 Hz, 2H); ^{13}C NMR (101 MHz, $CDCl_3$) 195.4, 172.5, 146.8, 135.3, 135.1, 132.9, 128.8, 128.6, 128.3, 128.1, 126.2, 123.8, 71.2, 61.6, 32.7, 32.1, 13.8; IR (CH_2Cl_2): 1733 (C=O), 1711 (C=O), 1239 (C-O) cm^{-1}; HRMS (EI-EBE) m/z: $[M]^+$ Calcd for $C_{21}H_{20}O_3$: 320.1412; Found 320.1411

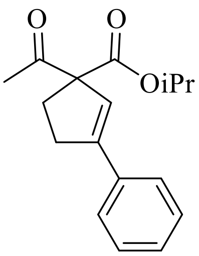

(32) isopropyl 1-acetyl-3-phenylcyclopent-2-ene-1-carboxylate Prepared in reaction of isopropyl 2-acetylpent-4-ynoate (117.7 mg, 0.6 mmol) with bromobenzene (62.8 mg, 0.4 mmol) under **conditions A**. The title compound was isolated as colorless oil (83%, 66.8 mg, 0.24 mmol) after chromatography on silica gel (15g column, Hexane:dioxane 98:2) ^{1}H NMR (400 MHz, $CDCl_3$) 7.49 – 7.45 (m, 2H), 7.37 – 7.31 (m, 2H), 7.31 – 7.27 (m, 1H), 6.25 (t, J = 1.8 Hz, 1H), 5.07 (hept, J = 6.3 Hz, 1H), 2.93 – 2.78 (m, 2H), 2.58 – 2.52 (m, 2H), 2.23 (s, 3H), 1.26 (dd, J = 6.3, 4.3 Hz, 6H); ^{13}C NMR (101 MHz, $CDCl_3$) 203.4, 171.1, 147.4, 135.1, 128.2, 126.0, 122.7, 73.9, 69.0, 32.4, 30.0, 26.5, 21.6; IR (CH_2Cl_2): 1742 (C=O), 1713 (C=O), 1236 (C-O) cm^{-1}; HRMS (ESI-TOF) m/z: $[M+Na]^+$ Calcd for $C_{17}H_{20}O_3Na$: 295.1310; Found 295.1311

Additionally prepared in reaction of isopropyl 2-acetylpent-4-ynoate (294.4 mg, 1.5 mmol) with bromobenzene (157.0 mg, 1.0 mmol) under **procedure for reaction run at 1 mmol scale**. The title compound was isolated as yellowish oil (87%, 237.0 mg, 0.87 mmol) after chromatography on silica gel (30g column, Hexane:Etyl Acetate 98:2→95:5)

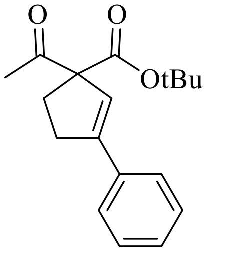

(33) tert-butyl 1-acetyl-3-phenylcyclopent-2-ene-1-carboxylate Prepared in reaction of tert-butyl 2-acetylpent-4-ynoate (126.1 mg, 0.6 mmol) with bromobenzene (62.8 mg, 0.4 mmol) under **conditions A**. The title compound was isolated as colorless oil (89%, 66.8 mg, 0.24 mmol) after chromatography on silica gel (15g column, Hexane:dioxane 98:2) ^{1}H NMR (400 MHz, $CDCl_3$) 7.49 – 7.45 (m, 2H), 7.36 – 7.30 (m, 2H), 7.30 – 7.26 (m, 1H), 6.23 (t, J = 1.8 Hz, 1H), 2.91 – 2.75 (m, 2H), 2.51 (td, J = 7.1, 6.4, 1.4 Hz, 2H), 2.22 (s, 3H), 1.47 (s, 9H); ^{13}C NMR (101 MHz, $CDCl_3$) 203.6, 170.7, 147.0, 135.2, 128.3, 128.1, 126.0, 123.0, 81.9, 74.6, 32.4, 30.0, 27.8, 26.5; IR (CH_2Cl_2): 1735 (C=O), 1712 (C=O), 1254 (C-O) cm^{-1}; HRMS (ESI-TOF) m/z: $[M+Na]^+$ Calcd for $C_{18}H_{22}O_3Na$: 309.1467; Found 309.1475

Additionally prepared in reaction of tert-butyl 2-acetylpent-4-ynoate (315.4 mg, 1.5 mmol) with bromobenzene (157.0 mg, 1.0 mmol) under **procedure for reaction run at 1 mmol scale**. The title compound was isolated as yellowish oil (91%, 261.5 mg, 0.91 mmol) after chromatography on silica gel (30g column, Hexane:Etyl Acetate 98:2→95:5)

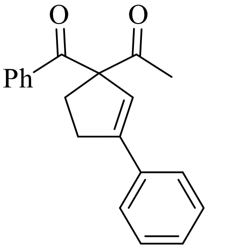

(34) 1-(1-benzoyl-3-phenylcyclopent-2-en-1-yl)ethan-1-one Prepared in reaction of 2-(but-3-yn-1-yl)-1-phenylbutane-1,3-dione (128.6 mg, 0.6 mmol) with bromobenzene (62.8 mg, 0.4 mmol) under **conditions A**. The title compound was isolated as beige solid (64%, 74.2 mg, 0.26 mmol) after chromatography on silica gel (15g column, Hexane:Ethyl Acetate 9:1) ^{1}H NMR (400 MHz, $CDCl_3$) 7.94 – 7.87 (m, 2H), 7.58 – 7.52 (m, 1H), 7.51 – 7.41 (m, 4H), 7.37 – 7.23 (m, 3H), 6.50 – 6.45 (m, 1H), 2.98 – 2.81 (m, 3H), 2.65 – 2.54 (m, 1H), 2.20 (s, 3H); ^{13}C NMR (101 MHz, $CDCl_3$) 204.4, 197.3, 147.0, 135.5, 135.0, 133.2, 129.2, 128.7, 128.4, 128.2, 126.1, 123.5, 79.7, 32.6, 30.7, 27.1; IR (CH_2Cl_2): 1712 (C=O), 1672 (C=O), 1235 (C-O) cm^{-1}; HRMS (EI-EBE) m/z: $[M]^+$ calc'd for $C_{20}H_{18}O_2$: 290.1307; found 290.1300

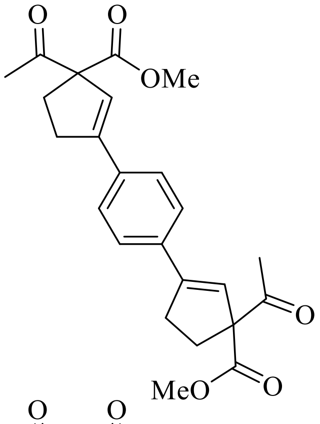

(35) dimethyl 3,3'-(1,4-phenylene)bis(1-acetylcyclopent-2-ene-1-carboxylate) Prepared in reaction of methyl 2-acetylhex-5-ynoate (100.9 mg, 0.6 mmol) with 4-bromophenyl trifluoromethanesulfonate (122.0 mg, 0.4 mmol) under **conditions B**. The title compound was isolated as off-white solid (67%, 83.0 mg, 0.20 mmol) after chromatography on silica gel (15g column, Hexane:Ethyl Acetate 7:3) ^{1}H NMR (400 MHz, $CDCl_3$) 7.42 (s, 4H), 6.26 (t, J = 1.7 Hz, 2H), 3.75 (s, 6H), 2.93 – 2.75 (m, 4H), 2.64 – 2.46 (m, 5H), 2.22 (s, 6H); ^{13}C NMR (101 MHz, $CDCl_3$) 203.4, 172.0, 146.8, 134.7, 126.1, 122.8, 73.7, 52.6, 32.3, 30.1, 26.5; IR (CH_2Cl_2): 1739 (C=O), 1713 (C=O), 1233 (C-O) cm^{-1}; HRMS (EI-EBE) m/z: $[M]^+$ Calcd for $C_{24}H_{26}O_6$: 410.1729; Found 410.1741

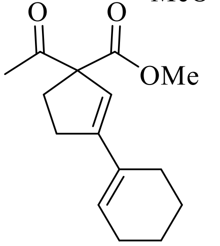

(36) methyl 1-acetyl-3-(cyclohex-1-en-1-yl)cyclopent-2-ene-1-carboxylate Prepared in reaction of methyl 2-acetylhex-5-ynoate (100.9 mg, 0.6 mmol) with cyclohex-1-en-1-yl trifluoromethanesulfonate (92.1 mg, 0.4 mmol) under **conditions B**. The title compound was isolated as colorless oil (52%, 52.0 mg, 0.21 mmol) after chromatography on silica gel (15g column, Hexane:Ethyl Acetate 95:5) ^{1}H NMR (400 MHz, $CDCl_3$) 5.83 (t, J = 4.0 Hz, 1H), 5.69 (s, 1H), 3.71 (s, 3H), 2.66 – 2.50 (m, 2H), 2.46 – 2.35 (m, 2H), 2.22 (ddq, J = 6.1, 4.0, 2.0 Hz, 2H), 2.16 (s, 3H), 2.15 – 2.09 (m, 2H), 1.67 (dtd, J = 10.3, 5.0, 4.1, 2.6 Hz, 2H), 1.58 (dtt, J = 9.0, 5.8, 2.6 Hz, 2H); ^{13}C NMR (101 MHz, $CDCl_3$) 204.0, 172.5, 149.4, 132.8, 128.0, 120.2, 73.4, 52.4, 31.3, 30.0, 26.4, 25.9, 25.8, 22.5, 22.1; IR (CH_2Cl_2): 1741 (C=O), 1713 (C=O), 1234 (C-O) cm^{-1}; HRMS (ESI-TOF) m/z: $[M+Na]^+$ Calcd for $C_{15}H_{20}O_3Na$: 271.1304; Found 271.1307

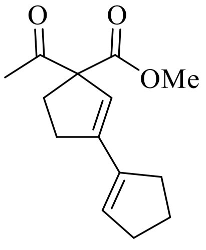

(37) methyl 3-acetyl-[1,1'-bi(cyclopentane)]-1,1'-diene-3-carboxylate Prepared in reaction of methyl 2-acetylhex-5-ynoate (100.9 mg, 0.6 mmol) with cyclopent-1-en-1-yl trifluoromethanesulfonate (86.5 mg, 0.4 mmol) under **conditions B**. The title compound was isolated as colorless oil (64%, 60.0 mg, 0.26 mmol) after chromatography on silica gel (15g column, Hexane:Ethyl Acetate 95:5) ^{1}H NMR (400 MHz, $CDCl_3$) 75.78 – 5.73 (m, 1H), 5.63 (s, 1H), 3.72 (s, 3H), 2.69 – 2.54 (m, 2H), 2.53 – 2.46 (m, 3H), 2.46 – 2.38 (m, 4H), 2.17 (s, 3H), 1.92 (p, *J* = 7.5 Hz, 2H); ^{13}C NMR (101 MHz, $CDCl_3$) 203.8, 172.4, 145.1, 139.3, 130.7, 122.8, 73.6, 52.5, 33.2, 32.7, 32.1, 30.4, 26.4, 23.3; IR (CH_2Cl_2): 1741 (C=O), 1713 (C=O), 1230 (C-O) cm^{-1}; HRMS (ESI-TOF) m/z: [M+Na]$^+$ Calcd for $C_{14}H_{18}O_3Na$: 257.1148; Found 257.1147

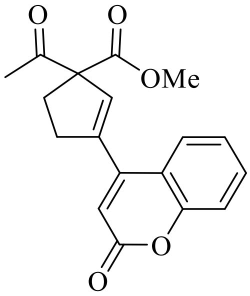

(38) methyl 1-acetyl-3-(2-oxo-2H-chromen-4-yl)cyclopent-2-ene-1-carboxylate Prepared in reaction of methyl 2-acetylhex-5-ynoate (100.9 mg, 0.6 mmol) with 2-oxo-2H-chromen-4-yl trifluoromethanesulfonate (117.7 mg, 0.4 mmol) under **conditions B**. The title compound was isolated as colorless oil (75%, 94.1 mg, 0.30 mmol) after chromatography on silica gel (15g column, Hexane:dioxane 9:1) ^{1}H NMR (400 MHz, $CDCl_3$) 7.73 (dd, *J* = 8.0, 1.4 Hz, 1H), 7.55 – 7.49 (m, 1H), 7.34 – 7.24 (m, 2H), 6.27 – 6.25 (m, 2H), 3.78 (s, 3H), 2.92 – 2.76 (m, 2H), 2.69 – 2.51 (m, 2H), 2.25 (s, 3H); ^{13}C NMR (101 MHz, $CDCl_3$) 202.0, 171.2, 160.5, 154.0, 150.2, 142.1, 132.0, 132.0, 126.1, 124.4, 117.9, 117.4, 113.6, 74.3, 53.0, 35.2, 30.2, 26.6; IR (CH_2Cl_2): 1740 (C=O), 1713 (C=O), 1256 (C-O), 1225 (C-O) cm^{-1}; HRMS (EI-EBE) m/z: [M]$^+$ Calcd for $C_{18}H_{16}O_5$: 312.0998; Found 312.1005

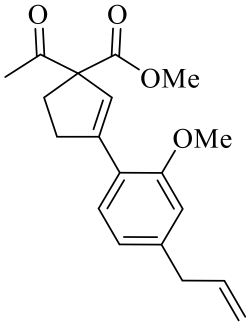

(39) methyl 1-acetyl-3-(4-allyl-2-methoxyphenyl)cyclopent-2-ene-1-carboxylate Prepared in reaction of methyl 2-acetylhex-5-ynoate (100.9 mg, 0.6 mmol) with 4-allyl-2-methoxyphenyl trifluoromethanesulfonate (118.5 mg, 0.4 mmol) under **conditions B**. The title compound was isolated as colorless oil (76%, 95.0 mg, 0.30 mmol) after chromatography on silica gel (15g column, Hexane:Ethyl Acetate 9:1) ^{1}H NMR (400 MHz, $CDCl_3$) 7.21 (d, *J* = 7.8 Hz, 1H), 6.77 (dd, *J* = 7.8, 1.5 Hz, 1H), 6.75 – 6.72 (m, 1H), 6.51 (t, *J* = 1.8 Hz, 1H), 6.01 – 5.90 (m, 1H), 5.11 (dq, *J* = 8.3, 1.5 Hz, 1H), 5.07 (t, *J* = 1.4 Hz, 1H), 3.87 (s, 3H), 3.75 (s, 3H), 3.40 – 3.36 (m, 2H), 2.96 – 2.81 (m, 2H), 2.54 – 2.42 (m, 2H), 2.23 (s, 3H); ^{13}C NMR (101 MHz, $CDCl_3$) 204.2, 172.5, 157.9, 143.9, 141.4, 136.9, 129.0, 126.0, 122.1, 120.5, 116.0, 111.3, 74.2, 55.2, 52.5, 40.1, 34.3, 29.6, 26.5; IR (CH_2Cl_2): 1740 (C=O), 1713 (C=O), 1256 (C-O), 1225 (C-O) cm^{-1}; HRMS (ESI-TOF) m/z: [M+Na]$^+$ Calcd for $C_{19}H_{22}O_4Na$: 337.1410; Found 337.1415

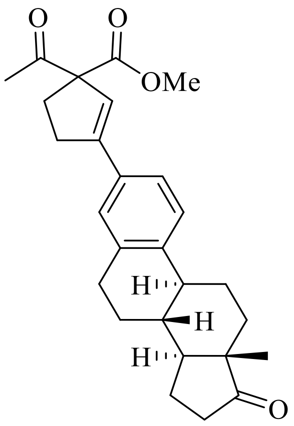

(40) methyl 1-acetyl-3-((8R,9S,13S,14S)-13-methyl-17-oxo-7,8,9,11,12,13,14,15,16,17-decahydro-6H-cyclopenta[a]phenanthren-3-yl)cyclopent-2-ene-1-carboxylate Prepared in reaction of methyl 2-acetylhex-5-ynoate (100.9 mg, 0.6 mmol) with (8R,9S,13S,14S)-13-methyl-17-oxo-7,8,9,11,12,13,14,15,16,17-decahydro-6H-cyclopenta[a]phenanthren-3-yl trifluoromethanesulfonate (160.9 mg, 0.4 mmol) under conditions B. The title compound was isolated as rubbery, white solid (72%, 121.1 mg, 0.29 mmol) after chromatography on silica gel (15g column, Hexane:Ethyl Acetate 95:5) ^{1}H NMR (400 MHz, $CDCl_3$) 7.26 (br s, 2H), 7.20 (br s, 1H), 6.21 – 6.18 (m, 1H), 3.74 (s, 3H), 2.91 (dd, *J* = 8.7, 4.1 Hz, 2H), 2.87 – 2.79 (m, 2H), 2.62 – 2.45 (m, 3H), 2.46 – 2.38 (m, 1H), 2.33 – 2.24 (m, 1H), 2.21 (s, 3H), 2.19 – 1.99 (m, 3H), 1.99 – 1.92 (m, 1H), 1.68 – 1.39 (m, 6H), 0.90 (s, 3H); ^{13}C NMR (101 MHz, $CDCl_3$): 220.6, 203.7,, 172.2, 147.3, 140.2, 136.5, 132.6, 126.7, 125.5, 123.6, 122.0, 73.7, 52.6, 50.5, 47.9, 44.4, 38.1, 35.8, 32.4, 31.6, 30.2, 29.4, 26.5, 26.4, 25.7, 21.6, 13.8; IR (CH_2Cl_2): 1739 (C=O), 1713 (C=O), 1256 (C-O) cm^{-1}; HRMS (EI-EBE) m/z: [M]$^+$ Calcd for $C_{27}H_{32}O_4$: 420.2301; Found 420.2295

methyl 2-acetylhex-5-ynoate Prepared in reaction of methyl 3-oxobutanoate (3.49 g, 30.0 mmol) with 4-iodobut-1-yne (5.51 g, 30.6 mmol) under **conditions C** (2.37 g, 14.1 mmol 47%). The title compound was isolated as yellowish oil after chromatography on silica gel (250 g column, Hexane:Ethyl Acetate 9:1) ^{1}H NMR (400 MHz, $CDCl_3$): 3.74 – 3.69 (m, 1H), 3.72 (s, 3H), 2.26 (s, 3H), 2.25 – 2.22 (m, 2H), 2.08 -2.02 (m, 2H), 1.98 (t, *J* = 2.6 Hz, 1H); ^{13}C NMR (101 MHz, $CDCl_3$): 202.4, 169.7, 82.5, 69.8, 57.6, 52.5, 29.4, 26.4, 16.3; Compound previously reported in literature.[4,5]

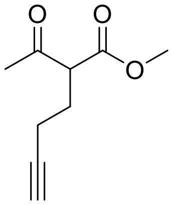

isopropyl 2-acetylhex-5-ynoate Prepared in reaction of isopropyl 3-oxobutanoate (2.88 g, 20.0 mmol) with 4-iodobut-1-yne (3.67 g, 20.4 mmol) under **conditions C** (1.02 g, 5.20 mmol 25%). The title compound was isolated as yellowish oil after chromatography on silica gel (200 g column, Hexane:Ethyl Acetate 95:5) ^{1}H NMR (400 MHz, $CDCl_3$): 5.04 (m, 1H), 3,64 (t, *J* = 7.1 Hz, 1H), 2.25-2.21 (m, 5H), 2.05-1.99 (m, 2H), 1.97 (t, *J* = 2.6 Hz, 1H),1.25 – 1.22 (m, 6H); ^{13}C NMR (101 MHz, $CDCl_3$): 202.5, 168.8, 82.6, 69.7, 69.1, 58.1, 29.2, 26.3, 21.6, 21.5, 16.2; Compound previously reported in literature.[5]

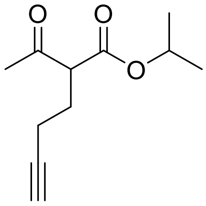

tert-butyl 2-acetylhex-5-ynoate Prepared in reaction of tert-butyl 3-oxobutanoate (3.16 g, 20.0 mmol) with 4-iodobut-1-yne (3.67 g, 20.4 mmol) under **conditions C** (1.27 g, 6.02 mmol 30%). The title compound was isolated as yellowish oil after chromatography on silica gel (200 g column, Hexane:Ethyl Acetate 95:5) ^{1}H NMR (400 MHz, $CDCl_3$): 3.58 (t, *J* = 7.2 Hz, 1H), 2.25 – 2.18 (m, 5H), 2.03 – 1.95 (m, 3H), 1.44 (s, 9H); ^{13}C NMR (101 MHz, $CDCl_3$): 202.8, 168.3, 82.7, 82.1, 69.6, 58.9, 29.2, 27.8, 26.3, 16.2. Compound previously reported in literature.[5,6]

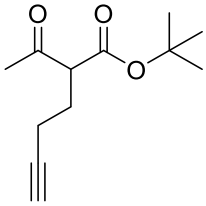

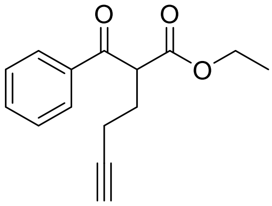

ethyl 2-benzoylhex-5-ynoate Prepared in reaction of ethyl 3-oxo-3-phenylpropanoate (4.89 g, 20.0 mmol) with 4-iodobut-1-yne (3.67 g, 20.4 mmol) under **conditions C** (1.88 g, 7.70 mmol 38%). The title compound was isolated as yellowish oil after chromatography on silica gel (250 g column, Hexane:Dioxane 95:5) ^{1}H NMR (400 MHz, $CDCl_3$): 8.05 – 8.00 (m, 2H), 7.62-7.56 (m, 1H), 7.52-7.45 (m, 2H), 4.60 (t, J = 7.1 Hz, 1H), 4.16 (q, *J* = 7.1, Hz, 2H), 2.39-2.26 (m, 2H), 2.25-2.18 (m, 2H), 2.02 (t, *J* = 2.6 Hz, 1H), 1.18 (t, J = 7.1 Hz, 3H); ^{13}C NMR (101 MHz, $CDCl_3$): 194.9, 169.5, 136.1, 133.6, 128.7, 82.8, 69.9, 67.1, 61.5, 52.4, 27.4, 16.5, 13.9; Compound previously reported in literature.[5]

methyl 2-isobutyrylhex-5-ynoate Prepared in reaction of ethyl methyl 2-isobutyrylhex-5-ynoate (2.88 g, 20.00 mmol) with 4-iodobut-1-yne (3.67 g, 20.4 mmol) under **conditions C** (2.08 g, 10.60 mmol 53%). The title compound was isolated as colorless oil after chromatography on silica gel (200 g column, Hexane:Ethyl Acetate 9:1) ^{1}H NMR (400 MHz, $CDCl_3$): 3.94 (t, *J* = 7.1 Hz, 1H), 3.72 (s, 3H), 2.83 (sept, *J* = 6.9 Hz, 1H), 2.31-2.15 (m, 2H), 2.07-2.02 (m, 2H), 1.99 (t, *J* = 2.6 Hz, 1H), 1.13 (d, *J* = 1.4 Hz, 3H), 1.11 (d, *J* = 1.2 Hz, 3H); ^{13}C NMR (101 MHz, $CDCl_3$): 208.6, 169.8, 82.7, 69.8, 54.7, 52.4, 40.9, 26.7, 18.3, 17.9, 16.4; Compound previously reported in literature.[5,7]

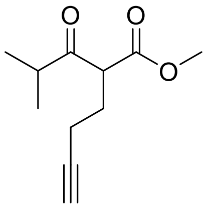

2-(but-3-yn-1-yl)-1-phenylbutane-1,3-dione Prepared in reaction of 1-phenylbutane-1,3-dione (1.62 g, 10.0 mmol) with 4-iodobut-1-yne (1.71 g, 9.5 mmol) under **conditions D** (0.29 g, 1.37 mmol 15%). The title compound was isolated as yellowish oil after chromatography on silica gel (250 g column, Hexane:Ethyl Acetate 95:5) ^{1}H NMR (400 MHz, $CDCl_3$): 8.06 – 7.99 (m, 2H), 7.64 – 7.57 (m, 1H), 7.52 – 7.45 (m, 2H), 4.75 (t, *J* = 6.7 Hz, 1H), 2.36 – 2.23 (m, 2H), 2.23 – 2.12 (m, 2H), 2.16 (s, 3H), 2.03 (t, *J* = 2.6 Hz, 1H).; ^{13}C NMR (101 MHz, $CDCl_3$): 203.3, 196.2, 136.2, 133.8, 128.9, 128.8, 82.8, 70.0, 60.9, 28.6, 27.1, 16.6. HRMS (EI-EBE) m/z: $[M]^+$ Calcd for $C_{14}H_{14}O_2$: 214.0994; Found 214.0990

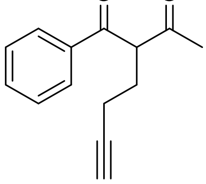

Computational Studies

General.

All calculations were performed using Gaussian 09 package.[8] Structures of minima and transition states were optimized employing B3LYP functional with the D3 version of Grimme's empirical dispersion correction[9] Def2-SVP bases set.[10] Frequency calculations were then performed at the same level of theory to confirm the nature of stationary points and provide corrections to thermodynamic functions. Single point energies were calculated at M06 level of theory using Def2-TZVPP basis set and SMD solvation model.[11] Structure of XPhos ligand was slightly trimmed – cyclohexyl groups at phosphorus were replaced with *i*-propyl. Various conformers of intermediates and transition states were investigated and only the lowest energy conformers are shown in the work. Molecular structures were visualized in CYLview.[12]

Optimized geometries, energies and corrections to thermodynamic functions.

PdLPhBr

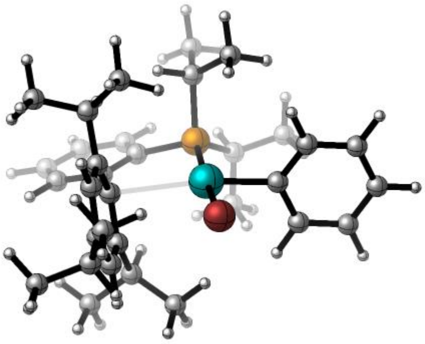

E (B3LYP-D3/Def2-SVP)) = -4327.58700213
E (SMD(DMF)/M06/Def2-TZVPP//B3LYP-D3/Def2-SVP) = -4328.15585297

Zero-point correction=	0.706994 (Hartree/Particle)
Thermal correction to Energy=	0.748542
Thermal correction to Enthalpy=	0.749486
Thermal correction to Gibbs Free Energy=	0.633319

Charge = 0 Multiplicity = 1

Pd	-0.46339300	-0.77389900	-0.11413600
P	-1.75137900	1.15210400	-0.24866100
C	-3.31628400	1.36024700	0.76249500
H	-3.63168700	2.40159000	0.58660200
C	-2.18728300	1.60509200	-2.03459500
H	-1.20532900	1.92924300	-2.41586800
C	-0.73930000	2.61664100	0.26181400
C	0.66302300	2.48333800	0.35199500
C	-1.32440100	3.87118600	0.52066600
C	1.43346900	3.62549000	0.64305900
C	-0.54716800	4.98786100	0.82669800
H	-2.40854000	3.98678500	0.49423400
C	0.84379000	4.86730100	0.87350700
H	2.51907200	3.51766000	0.70072900
H	-1.02841300	5.94856800	1.02530500
H	1.46748600	5.73488600	1.10280800
C	1.43586300	1.19678900	0.21325500

C	2.05523900	0.86883400	-1.02227100
C	1.83900000	0.52032400	1.40347300
C	3.06341800	-0.10166600	-1.04388400
C	2.86119400	-0.42442900	1.32496900
C	3.48758900	-0.75408700	0.11566400
H	3.51699400	-0.36165900	-2.00053000
H	3.16409400	-0.95602800	2.23005200
C	-2.04421100	-1.97534300	0.11237200
C	-2.42872200	-2.32291000	1.41508700
C	-2.75648500	-2.49941900	-0.97243900
C	-3.55171200	-3.13149500	1.62885600
H	-1.85429600	-1.97632600	2.27590500
C	-3.88082700	-3.30561400	-0.75402200
H	-2.43807700	-2.29724800	-1.99424400
C	-4.29246200	-3.61384700	0.54577500
H	-3.84244900	-3.38679600	2.65185100
H	-4.43160200	-3.70106100	-1.61234500
H	-5.17126500	-4.24171300	0.71309000
C	1.67459200	1.58410700	-2.31262800
H	0.81916100	2.23900000	-2.08796800
C	4.60037100	-1.79091200	0.10677500
H	4.36448800	-2.50296700	0.91619600
C	1.19171100	0.83380500	2.74928200
H	0.30086100	1.44899700	2.55266600
C	2.81219100	2.49437900	-2.80339200
H	3.71504300	1.90798100	-3.03863600
H	2.51359400	3.03642000	-3.71572200
H	3.08399400	3.23746600	-2.03728700
C	1.22729100	0.59759900	-3.40279800
H	0.83640100	1.13855800	-4.28010300
H	2.06248700	-0.03303400	-3.74554000
H	0.44329500	-0.07734700	-3.02754500
C	0.70974400	-0.43336400	3.47263800
H	0.12810200	-0.16456100	4.36931700
H	0.07860400	-1.04657600	2.81305400
H	1.55104100	-1.06408100	3.80035900
C	2.13427500	1.66006000	3.64024900
H	2.42218000	2.60387800	3.15249700
H	1.65015900	1.90532800	4.59982200
H	3.05696900	1.09833600	3.85976300
C	5.94894200	-1.12703100	0.44262300
H	6.75386400	-1.87818800	0.50024300
H	6.22288500	-0.39313600	-0.33385200
H	5.90653800	-0.59413300	1.40576000
C	4.68484100	-2.59478200	-1.19571000
H	3.70319900	-3.01728200	-1.45745800
H	5.03193000	-1.97230400	-2.03735300
H	5.40592700	-3.42052400	-1.08703100
C	-3.16585100	2.77517100	-2.18794600
H	-4.16940100	2.52495300	-1.81064700
H	-2.81947300	3.68489400	-1.67852200
H	-3.27381100	3.02289900	-3.25691500
C	-2.61909600	0.39063400	-2.86449900
H	-1.85367700	-0.39652600	-2.84960500

H	-3.55962000	-0.05002900	-2.50337500
H	-2.77009600	0.69658300	-3.91282300
C	-2.98609700	1.19956100	2.25272800
H	-2.21626800	1.91307000	2.58429200
H	-2.62799700	0.18343700	2.46901000
C	-4.47458800	0.45199100	0.32770000
H	-4.25391100	-0.60944800	0.49083200
H	-5.36797800	0.70490100	0.92247100
H	-4.73736400	0.58599700	-0.73107900
H	-3.89080600	1.37424200	2.85772000
Br	0.86594700	-2.85314200	-0.25328300

Compound 1 (enol form)

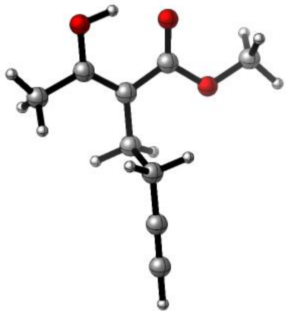

E (B3LYP-D3/Def2-SVP)) = -575.398596939
E (SMD(DMF)/M06/Def2-TZVPP//B3LYP-D3/Def2-SVP) = -575.667738469

Zero-point correction=	0.194763 (Hartree/Particle)
Thermal correction to Energy=	0.208084
Thermal correction to Enthalpy=	0.209028
Thermal correction to Gibbs Free Energy=	0.154115

Charge = 0 Multiplicity = 1

C	-1.04074900	-0.28005900	0.60697300
C	0.41564100	-0.45130000	0.24527000
C	-4.50354400	0.37341500	0.03273700
C	-3.33844500	0.24368300	-0.27435900
C	-1.92616700	0.07618000	-0.61252600
H	-1.14173900	0.52339500	1.35177800
H	-5.53717900	0.49205600	0.29760800
H	-1.54227800	1.00580700	-1.06521100
H	-1.82267800	-0.70942100	-1.38203300
H	-1.43431000	-1.19047200	1.08176300
C	1.00031300	-1.68349000	0.03452700
C	1.25635400	0.72269500	0.05806200
O	2.27823700	-1.83052400	-0.28152500
O	2.45854000	0.69680600	-0.22868000
O	0.61019900	1.89094300	0.21682200
C	0.26439300	-2.98857900	0.15670500
H	0.86191900	-3.78578600	-0.30475700
H	0.10725800	-3.24424600	1.21798400
H	-0.72303100	-2.94969600	-0.32440300
C	1.38363400	3.07921000	0.04888200
H	0.69406500	3.91628800	0.21426100
H	2.21054700	3.11437600	0.77471100

H	1.81149800	3.12873100	-0.96422600
H	2.67155500	-0.90935800	-0.32803600

TS, M=H, 5-endo carbocyclizaton

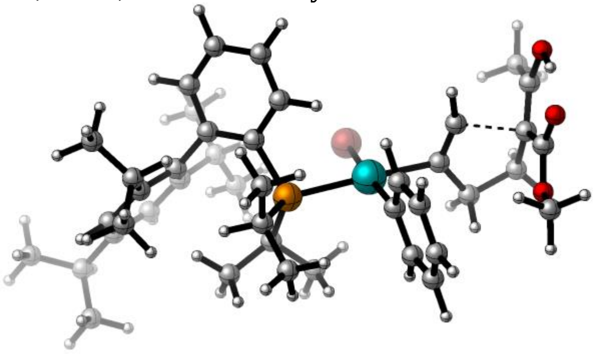

E (B3LYP-D3/Def2-SVP)) = -4902.97338070
E (SMD(DMF)/M06/Def2-TZVPP//B3LYP-D3/Def2-SVP) = -4903.80142553

Zero-point correction=	0.901799 (Hartree/Particle)
Thermal correction to Energy=	0.957307
Thermal correction to Enthalpy=	0.958251
Thermal correction to Gibbs Free Energy=	0.809029

Charge = 0 Multiplicity = 1

Pd	-1.53836100	0.01548700	-0.22435000
P	0.71559600	0.73228900	-0.02409600
C	1.21376800	2.50437400	0.35903200
H	2.31037400	2.48554600	0.26597200
C	1.59237900	0.31763900	-1.64556200
H	1.88742800	-0.72547300	-1.48204100
C	1.42102400	-0.19004700	1.43752200
C	2.74211100	-0.63955700	1.66943700
C	0.46116300	-0.39041000	2.45232500
C	3.03636400	-1.26693300	2.89654400
C	0.77505300	-0.99598200	3.66590200
H	-0.56789400	-0.07119400	2.27134800
C	2.07750200	-1.44371000	3.89108100
H	4.05615900	-1.62312700	3.05866500
H	-0.00089900	-1.13061300	4.42330500
H	2.34531300	-1.93466500	4.83013200
C	3.88717700	-0.51777600	0.70874900
C	4.80077200	0.55622300	0.83728100
C	4.10526700	-1.52702000	-0.25885400
C	5.90710400	0.61076100	-0.01980000
C	5.21776700	-1.41499800	-1.10392800
C	6.12793900	-0.35785400	-1.00539000
H	6.60939800	1.44153500	0.07476700
H	5.38634300	-2.17923900	-1.86603700
C	-2.36157700	1.85732300	-0.20307500
C	-2.75920900	2.49510400	0.98615700
C	-2.63538600	2.51706500	-1.41680000
C	-3.37613200	3.75343500	0.96805100

H	-2.58302300	2.00936400	1.95048400
C	-3.25445300	3.77332400	-1.44096200
H	-2.34374500	2.05498700	-2.36499300
C	-3.62185400	4.40302300	-0.24678100
H	-3.66213000	4.23007300	1.91073500
H	-3.44167300	4.26680400	-2.39977400
H	-4.09584900	5.38816600	-0.26217400
C	4.61529100	1.64072100	1.89662900
H	3.57000300	1.59475600	2.23627100
C	7.30442500	-0.24869900	-1.96435500
H	7.29464200	-1.15580800	-2.59353600
C	3.18493100	-2.74189200	-0.35610500
H	2.19648400	-2.45589400	0.03361200
C	5.50508000	1.36868000	3.12237000
H	6.57021000	1.37221700	2.83717400
H	5.35600500	2.14099100	3.89510700
H	5.28001500	0.39051000	3.57150900
C	4.85942300	3.06079900	1.36073300
H	4.55081400	3.80897600	2.10846100
H	5.92426400	3.23985300	1.14181100
H	4.29482400	3.25080000	0.43489900
C	2.96185600	-3.24455900	-1.78990200
H	2.13678600	-3.97249700	-1.80039300
H	2.68606400	-2.42650600	-2.47306400
H	3.85676500	-3.74082300	-2.20059800
C	3.70769800	-3.88559500	0.53249100
H	3.79215400	-3.57372100	1.58351900
H	3.02253100	-4.74772400	0.49015000
H	4.70373500	-4.21899900	0.19540800
C	8.65361800	-0.21167600	-1.22913500
H	9.49015600	-0.19902500	-1.94698000
H	8.74329200	0.68986300	-0.60104400
H	8.77511400	-1.08882900	-0.57446000
C	7.14491600	0.96326000	-2.89856900
H	6.19143400	0.91843200	-3.44798100
H	7.15535000	1.90584600	-2.32658600
H	7.96556900	1.00672200	-3.63361900
C	2.83069500	1.14503000	-2.01000500
H	2.56222400	2.17166900	-2.30081700
H	3.57199100	1.19687900	-1.20752100
H	3.32782800	0.67961900	-2.87624100
C	0.58617400	0.32879000	-2.81047200
H	-0.22720500	-0.39207700	-2.64927900
H	0.14390300	1.32653900	-2.96374700
H	1.10908600	0.05061400	-3.74093600
C	0.86118500	2.92980600	1.78919600
H	1.32965600	2.28915900	2.54942500
H	-0.22748100	2.91487200	1.94830900
C	0.65007200	3.51095500	-0.65422300
H	-0.42095200	3.68272800	-0.48752000
H	1.17110300	4.47619500	-0.53836500
H	0.77573300	3.19043900	-1.69761200
H	1.20792500	3.96306800	1.95820300
Br	-0.69112300	-2.41685900	-0.46438900

C	-3.45503400	-0.68792000	-0.52607500
C	-4.14857300	-0.84713000	0.54552600
C	-4.03629300	-0.89491400	-1.88139800
H	-4.25624300	-0.75344800	1.61868500
C	-5.42571100	-1.58481500	-1.69462900
H	-3.36438700	-1.49017600	-2.51757400
H	-4.14657600	0.09524400	-2.35261500
C	-5.97439500	-1.29303600	-0.31544300
H	-6.13309500	-1.23745800	-2.46195000
H	-5.30923400	-2.66805800	-1.82572200
C	-6.24175100	-2.30590900	0.63111700
C	-6.53282600	0.04759800	-0.03335800
O	-6.81526400	-2.02851000	1.77093200
C	-5.82963700	-3.73364100	0.45990600
O	-7.06866600	0.34952000	1.03732600
O	-6.35525900	0.91448300	-1.01594500
H	-6.00841700	-4.27772700	1.39568300
H	-6.40842000	-4.20838700	-0.34847500
H	-4.76359900	-3.79708400	0.19455400
C	-6.61283300	2.29962400	-0.73261600
H	-6.46089600	2.82960800	-1.67911000
H	-7.63901400	2.43508900	-0.36251900
H	-5.89163200	2.66361000	0.01353400
H	-7.05538400	-1.03957100	1.75257400

TS, M=H, 6-exo oxocyclizaton

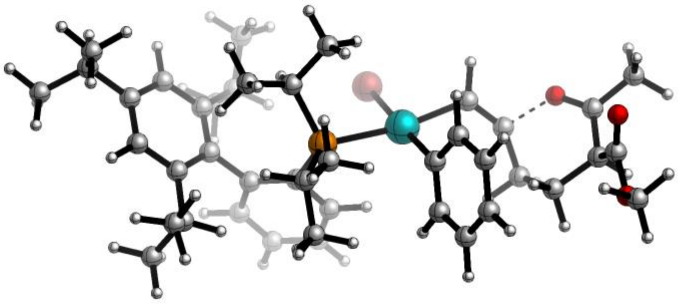

E (B3LYP-D3/Def2-SVP)) = -4902.95906440
E (SMD(DMF)/M06/Def2-TZVPP//B3LYP-D3/Def2-SVP) = -4903.79568276

Zero-point correction=	0.902286 (Hartree/Particle)
Thermal correction to Energy=	0.957601
Thermal correction to Enthalpy=	0.958545
Thermal correction to Gibbs Free Energy=	0.809128

Charge = 0 Multiplicity = 1

P	0.79263200	0.44488900	-0.15173700
C	1.03201600	2.31265800	-0.19856100
H	2.10801800	2.42520300	-0.39953700
C	1.70556000	-0.16615200	-1.69542100
H	2.15653800	-1.09769300	-1.33391500
C	1.66331100	-0.02180400	1.43311600
C	3.04447800	-0.17719400	1.69881000
C	0.75914200	-0.16836600	2.50639700
C	3.45094200	-0.46384000	3.01742700

C	1.18058100	-0.43511300	3.80632400
H	-0.31114000	-0.08838600	2.30294200
C	2.54317900	-0.58602300	4.06678000
H	4.51834700	-0.59661500	3.20768900
H	0.44366700	-0.54188800	4.60607900
H	2.89835700	-0.80878700	5.07618700
C	4.14513600	-0.08219800	0.68419100
C	4.87211500	1.12450700	0.54306800
C	4.51627300	-1.22815700	-0.05905500
C	5.94397600	1.16916600	-0.35739600
C	5.58552100	-1.12477700	-0.95921000
C	6.30956000	0.05964300	-1.12764700
H	6.50065500	2.10164600	-0.47177800
H	5.86904700	-1.99798200	-1.55112900
C	-2.44672200	1.05537300	-0.18881600
C	-2.64501700	1.84708900	0.96065800
C	-3.15243300	1.43449000	-1.34983600
C	-3.49183400	2.96444700	0.95324500
H	-2.12958600	1.59198700	1.89149700
C	-3.99994400	2.54962800	-1.36683300
H	-3.04301400	0.84447300	-2.26410200
C	-4.17496600	3.32426900	-0.21319800
H	-3.61607500	3.55639400	1.86535900
H	-4.52877500	2.81219600	-2.28846900
H	-4.83013600	4.19986900	-0.22463200
C	4.52569000	2.36678800	1.36084500
H	3.51003300	2.22681500	1.75808300
C	7.43851200	0.14598600	-2.14434200
H	7.57435400	-0.86605200	-2.56403800
C	3.81018900	-2.56671400	0.14439100
H	2.78992800	-2.36568700	0.50354300
C	5.47145300	2.51146200	2.56614000
H	6.51506700	2.62696400	2.22930000
H	5.20741700	3.39635700	3.16880800
H	5.42487100	1.62853900	3.22001900
C	4.51538600	3.65919500	0.52865100
H	4.10516200	4.49180500	1.12253700
H	5.52930800	3.95305700	0.21305000
H	3.90274200	3.55436900	-0.37982800
C	3.66554900	-3.39945400	-1.13750400
H	2.97103700	-4.23363500	-0.95765400
H	3.25425100	-2.80510700	-1.96774600
H	4.62778500	-3.82437000	-1.46850500
C	4.52114200	-3.38888800	1.23509100
H	4.55910600	-2.84510300	2.19009600
H	3.98825500	-4.33795100	1.40778600
H	5.55669600	-3.62364500	0.93577800
C	8.77153600	0.56187200	-1.50240400
H	9.58426200	0.55286700	-2.24722900
H	8.71554400	1.58133700	-1.08661100
H	9.04864300	-0.11897800	-0.68236800
C	7.06343600	1.08155500	-3.30678700
H	6.12401100	0.76307700	-3.78530700
H	6.92125800	2.11520500	-2.95018700

H	7.85491400	1.09607500	-4.07436200
C	2.79893400	0.73919900	-2.27514400
H	2.37318600	1.62881200	-2.76383500
H	3.53252300	1.07056900	-1.53511200
H	3.35228200	0.17858600	-3.04570700
C	0.70330700	-0.54550800	-2.80076200
H	0.01731500	-1.33717500	-2.47194100
H	0.10670700	0.31938800	-3.13307700
H	1.26078800	-0.92023400	-3.67585400
C	0.72902600	3.01957300	1.12738700
H	1.28616000	2.59182900	1.97263900
H	-0.34244200	2.97780900	1.36615500
C	0.24518900	2.95564000	-1.34916100
H	-0.83526500	2.92672500	-1.15388200
H	0.54491800	4.01180900	-1.45726600
H	0.42186700	2.46151800	-2.31476200
H	1.00907800	4.08327800	1.04318800
Br	-0.04421300	-2.86832000	0.04534100
Pd	-1.32920300	-0.63031200	-0.13320600
C	-3.07842400	-1.70048200	-0.42032200
H	-3.10895000	-2.28988100	-1.34049000
C	-4.10248900	-1.60168700	0.36675800
C	-4.62261000	-0.94499800	1.57822600
C	-5.67140700	0.10685300	1.20621000
H	-5.02612800	-1.68967900	2.28796300
H	-3.76678200	-0.45580400	2.06269900
C	-6.88495000	-0.49080600	0.45087900
H	-5.18270300	0.85443200	0.56863200
H	-6.04051100	0.64088000	2.09284600
C	-6.62637300	-1.77996200	-0.30736900
C	-7.44184300	0.58626600	-0.48682800
H	-7.69029500	-0.73985700	1.16715700
O	-5.52632300	-2.34775200	-0.30475500
C	-7.73690400	-2.40586500	-1.08678100
O	-7.82975000	1.64520400	0.22468100
O	-7.47312700	0.52343800	-1.69095300
H	-7.82245400	-1.84008400	-2.02995700
H	-7.50694100	-3.45682900	-1.30249300
H	-8.69922200	-2.30815100	-0.56256300
C	-8.17862500	2.82146600	-0.51942800
H	-8.98129600	2.60374000	-1.23906700
H	-8.50993400	3.55934800	0.22053200
H	-7.29711600	3.19291600	-1.06309400

Enol of compound 1 ('naked')

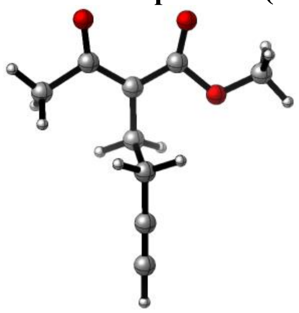

E (B3LYP-D3/Def2-SVP)) = -574.822369811
E (SMD(DMF)/M06/Def2-TZVPP//B3LYP-D3/Def2-SVP) = -575.169472432

Zero-point correction=	0.179753 (Hartree/Particle)
Thermal correction to Energy=	0.193124
Thermal correction to Enthalpy=	0.194068
Thermal correction to Gibbs Free Energy=	0.139400

Charge = -1 Multiplicity = 1

C	-3.23620800	-0.30792700	0.27411200
C	-4.40504900	-0.44480400	-0.02848400
C	-1.82376500	-0.11656800	0.60114400
H	-5.43526500	-0.57148000	-0.29768100
C	-0.92017900	0.25698000	-0.61286500
H	-1.41662800	-1.04207900	1.04007200
H	-1.73201500	0.66830700	1.37263700
C	0.53032000	0.47385800	-0.23861200
H	-1.01663100	-0.55977100	-1.34590700
H	-1.35180100	1.14844100	-1.09263000
C	1.05945800	1.79114700	-0.00940300
C	1.36119800	-0.67765700	-0.04722800
O	2.22088200	2.08638300	0.29986000
C	0.07004000	2.98074100	-0.16225000
O	2.54709100	-0.79250400	0.22068100
O	0.60348300	-1.87951900	-0.19956700
H	0.60647600	3.88864000	0.14872600
H	-0.26151500	3.10777600	-1.20837000
H	-0.84016500	2.87002400	0.45275000
C	1.34162500	-3.06244100	-0.04632500
H	0.63896500	-3.90082400	-0.18743300
H	2.16253200	-3.14429600	-0.78323200
H	1.80975000	-3.13959400	0.95274200

TS, M= - , 5-endo carbocyclizaton

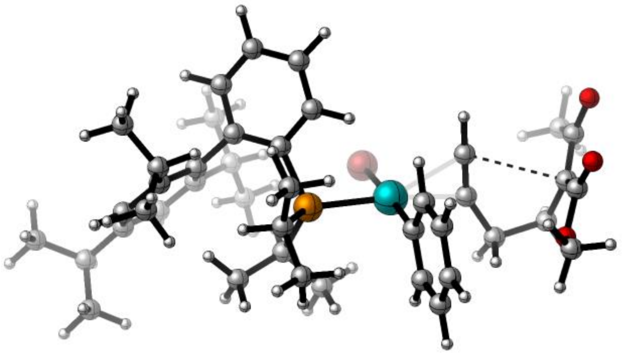

E (B3LYP-D3/Def2-SVP)) = -4902.42156292
E (SMD(DMF)/M06/Def2-TZVPP//B3LYP-D3/Def2-SVP) = -4903.32227483

Zero-point correction=	0.886873 (Hartree/Particle)
Thermal correction to Energy=	0.942907
Thermal correction to Enthalpy=	0.943851
Thermal correction to Gibbs Free Energy=	0.792912

Charge = -1 Multiplicity = 1

Pd	-1.54687800	-0.11127400	-0.19559100
P	0.68759000	0.64293300	-0.03582600
C	1.18178700	2.42024200	0.34469500
H	2.27833400	2.39487800	0.25259000
C	1.56760500	0.23885800	-1.65979100
H	1.87856700	-0.79883500	-1.49199600
C	1.43433900	-0.24776800	1.43042600
C	2.77308800	-0.63827400	1.66756400
C	0.48513800	-0.47989000	2.44851500
C	3.09458100	-1.24173000	2.90032500
C	0.82408800	-1.06083200	3.66714900
H	-0.55681500	-0.20811600	2.26447700
C	2.14428600	-1.45081900	3.89665300
H	4.12843700	-1.55503800	3.06385500
H	0.05228600	-1.22335000	4.42322200
H	2.43204700	-1.92362500	4.83934600
C	3.91403100	-0.48373500	0.70592000
C	4.79317500	0.61978900	0.82609100
C	4.16548100	-1.49169400	-0.25540900
C	5.89510600	0.70699100	-0.03433600
C	5.27224900	-1.34789000	-1.10335800
C	6.14566400	-0.25966900	-1.01450400
H	6.56825800	1.56261300	0.05292000
H	5.46283600	-2.11071700	-1.86186300
C	-2.33248900	1.74293800	-0.20442500
C	-2.74245700	2.39109300	0.97129400
C	-2.54105900	2.39981100	-1.42936900
C	-3.31378300	3.66908100	0.92874800
H	-2.63211300	1.89223300	1.93807700
C	-3.12028700	3.67379000	-1.47564000
H	-2.24743100	1.91716900	-2.36586000
C	-3.49920800	4.31961600	-0.29500100
H	-3.62913600	4.15122100	1.85887800
H	-3.27695200	4.16236400	-2.44233900
H	-3.95564400	5.31258300	-0.32932300
C	4.57599700	1.70277300	1.88103500
H	3.53424700	1.62390800	2.22397000
C	7.31267700	-0.11526000	-1.98065500
H	7.33176300	-1.02652800	-2.60379800
C	3.28528500	-2.73551400	-0.34445700
H	2.28565100	-2.47802000	0.03625600
C	5.47795300	1.46538100	3.10505900
H	6.54192400	1.50216300	2.81659100
H	5.30571300	2.23599000	3.87501900
H	5.28497400	0.48265500	3.55896500
C	4.77197000	3.12800400	1.33926100
H	4.44243200	3.86803800	2.08632900
H	5.82967900	3.34051600	1.11387700
H	4.19503700	3.29607600	0.41708500
C	3.08345000	-3.25914400	-1.77362900
H	2.26892800	-3.99871700	-1.78022100
H	2.79738500	-2.45367900	-2.46728000
H	3.99037600	-3.74463000	-2.17238100
C	3.83780400	-3.85337700	0.55854200

H	3.90675900	-3.52781900	1.60658200
H	3.17640400	-4.73408200	0.52232200
H	4.84501900	-4.16226900	0.22961300
C	8.66414100	-0.02548500	-1.25413800
H	9.49656300	0.01427500	-1.97642600
H	8.72355800	0.88154600	-0.63026100
H	8.82035000	-0.89428900	-0.59552100
C	7.10571100	1.08364600	-2.92232500
H	6.15056800	1.00119700	-3.46415200
H	7.08511500	2.02957400	-2.35611700
H	7.91994400	1.15173500	-3.66327600
C	2.78734200	1.08620600	-2.04086500
H	2.49687800	2.10590000	-2.33511800
H	3.53606700	1.15850800	-1.24690900
H	3.28406000	0.62479100	-2.91007400
C	0.55160700	0.22508800	-2.81570600
H	-0.25434400	-0.49993200	-2.63634700
H	0.10005600	1.21698600	-2.97859900
H	1.06820700	-0.06282200	-3.74720600
C	0.82941600	2.84843100	1.77444900
H	1.29477100	2.20850800	2.53725300
H	-0.25957300	2.83546000	1.93009600
C	0.62790700	3.43689500	-0.66420600
H	-0.43142900	3.64799900	-0.47235800
H	1.18697500	4.38380500	-0.57055100
H	0.71197500	3.10315600	-1.70748000
H	1.17765800	3.88199700	1.94150700
Br	-0.53356300	-2.50633600	-0.41843500
C	-3.60687900	-0.79481700	-0.56891900
C	-3.62313200	-1.01651700	0.64893000
C	-4.34265200	-0.63866400	-1.82641600
H	-3.64778000	-1.24168800	1.69933700
C	-5.78363700	-1.26300500	-1.67118800
H	-3.79408600	-1.09048900	-2.66999600
H	-4.43166400	0.44173600	-2.01502200
C	-6.41894400	-1.12069400	-0.31226300
H	-6.39020100	-0.76904700	-2.44989800
H	-5.71060200	-2.32164700	-1.95138300
C	-6.61663300	-2.25190300	0.56462700
C	-6.77694100	0.20353300	0.12890900
O	-7.11423700	-2.21955500	1.69542300
C	-6.15916400	-3.64160000	0.05122000
O	-7.26719900	0.60121200	1.17156700
O	-6.45707000	1.15936900	-0.86451500
H	-6.36090300	-4.36335400	0.85507300
H	-6.71028100	-3.95538400	-0.85271200
H	-5.08400700	-3.66597200	-0.19284500
C	-6.68638400	2.49846100	-0.49686300
H	-6.35116400	3.12156400	-1.34051200
H	-7.75365200	2.69338200	-0.28575700
H	-6.11609300	2.77958300	0.40463700

TS, M= - , 6-exo oxocyclizaton

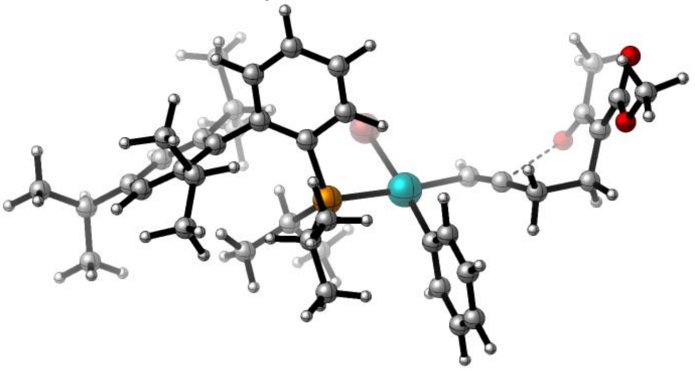

E (B3LYP-D3/Def2-SVP)) = -4902.43772937
E (SMD(DMF)/M06/Def2-TZVPP//B3LYP-D3/Def2-SVP) = -4903.32709950

Zero-point correction=	0.887720 (Hartree/Particle)
Thermal correction to Energy=	0.943399
Thermal correction to Enthalpy=	0.944343
Thermal correction to Gibbs Free Energy=	0.793210

Charge = -1 Multiplicity = 1

Pd	-1.34842500	0.09725700	-0.89875400
P	0.84556800	0.68608200	-0.28355800
C	1.40535200	2.40449300	0.25820600
H	2.49850900	2.28754400	0.30776400
C	1.97398200	0.29157400	-1.75482800
H	2.18669600	-0.77128400	-1.59115600
C	1.27260700	-0.31198600	1.24052900
C	2.52961500	-0.77159000	1.70055000
C	0.14198100	-0.54682100	2.05142500
C	2.59430900	-1.43565900	2.94221800
C	0.22743600	-1.19127600	3.28255800
H	-0.83845200	-0.22882900	1.68893000
C	1.46790400	-1.64285000	3.73494800
H	3.56732900	-1.80182100	3.27834700
H	-0.67806900	-1.35475600	3.87156700
H	1.55836700	-2.16439700	4.69145100
C	3.83203500	-0.64279200	0.96637300
C	4.73126200	0.40088800	1.29433600
C	4.20117100	-1.62486600	0.01568500
C	5.97015300	0.46045000	0.64305200
C	5.44579600	-1.51036400	-0.61851400
C	6.34154600	-0.47737400	-0.32647800
H	6.65911300	1.27122800	0.88985900
H	5.73017300	-2.25244700	-1.36813200
C	-1.98403300	2.00692700	-0.87553500
C	-2.36586000	2.65955900	0.30919500
C	-2.08561100	2.72280800	-2.08158900
C	-2.81080000	3.98733200	0.29615600
H	-2.33475400	2.12207000	1.26108700
C	-2.53191800	4.04987300	-2.09954900
H	-1.81665000	2.23942600	-3.02487900
C	-2.88934100	4.69272000	-0.90892100
H	-3.10356700	4.46953600	1.23395100

H	-2.60273200	4.58445800	-3.05207000
H	-3.23838100	5.72898400	-0.92202900
C	4.39160900	1.44551400	2.35526300
H	3.30530100	1.40832800	2.51739600
C	7.66539500	-0.35982900	-1.06799400
H	7.74959800	-1.24511300	-1.72243800
C	3.29487300	-2.81609900	-0.28251000
H	2.25671900	-2.51940900	-0.07248000
C	5.06471600	1.10294600	3.69608900
H	6.16250600	1.09508300	3.58926200
H	4.80174900	1.84516700	4.46832700
H	4.75386800	0.11210300	4.05757800
C	4.74146700	2.88118900	1.93176800
H	4.33202400	3.60253300	2.65727400
H	5.83073800	3.04372800	1.88910000
H	4.32923000	3.12515200	0.94091800
C	3.32707000	-3.27778300	-1.74636300
H	2.49509900	-3.97422900	-1.92855900
H	3.20412400	-2.43473500	-2.44332200
H	4.26801700	-3.79481000	-2.00045100
C	3.62687300	-3.99228300	0.65394700
H	3.52354500	-3.70712500	1.71088500
H	2.94302100	-4.83536000	0.46414900
H	4.66133200	-4.34216100	0.49382000
C	8.87155200	-0.37466700	-0.11545700
H	9.81853800	-0.35229800	-0.68023900
H	8.86412300	0.50182500	0.55326000
H	8.86830800	-1.27659400	0.51662200
C	7.68416200	0.88543800	-1.97124700
H	6.83591000	0.87785600	-2.67343400
H	7.60964100	1.80755400	-1.37127400
H	8.61800300	0.93791400	-2.55595400
C	3.29172900	1.06694500	-1.87389200
H	3.12170000	2.11426800	-2.16584400
H	3.89168100	1.05864700	-0.95965000
H	3.90518700	0.60393000	-2.66408600
C	1.18466000	0.39694700	-3.07198400
H	0.32703200	-0.28904700	-3.08418500
H	0.81565500	1.41961700	-3.25224200
H	1.84890700	0.12878100	-3.91144000
C	0.91564300	2.81264400	1.65246300
H	1.17529200	2.07766600	2.42694300
H	-0.17372700	2.95231600	1.66388700
C	1.07789600	3.49981100	-0.76724100
H	0.01865300	3.78227400	-0.72290400
H	1.67964100	4.39828900	-0.54602600
H	1.29518000	3.20084100	-1.80178000
H	1.37776700	3.77528700	1.93106500
Br	-0.40034600	-2.34031800	-1.05858900
C	-3.22234300	-0.74246300	-1.64557900
C	-4.10973800	-0.05204500	-1.11032700
H	-3.06002200	-1.63686900	-2.23322400
C	-5.00558400	0.88268700	-0.46226100
C	-6.52976900	0.54404700	-0.62367400

H	-4.78150900	0.92249800	0.61597100
H	-4.80440000	1.88453800	-0.87888100
C	-6.96085700	-0.73236600	0.03321400
H	-6.73619700	0.49364500	-1.70441800
H	-7.06412300	1.40503500	-0.20009400
C	-6.65643000	-1.92090100	-0.69811500
C	-7.49016500	-0.74670000	1.35811500
O	-6.04376800	-1.86096000	-1.78896500
C	-7.04778900	-3.29406500	-0.16188000
O	-7.51055800	0.53700500	1.93213700
O	-7.90096300	-1.69009900	2.03193600
H	-6.54312500	-3.49648200	0.79567100
H	-8.12405500	-3.34752300	0.06119100
H	-6.76290400	-4.04854400	-0.91008300
C	-8.03331900	0.59897900	3.23663300
H	-7.45941400	-0.02528700	3.94497800
H	-7.98174800	1.65406400	3.54998700
H	-9.08142600	0.25148600	3.28313800

K-enolate of compound 1

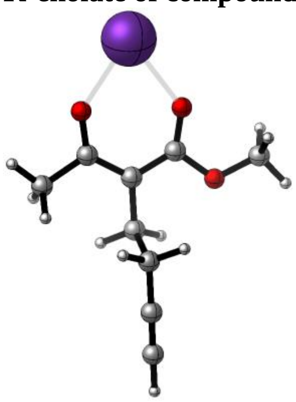

E (B3LYP-D3/Def2-SVP)) = -1174.72115505
E (SMD(DMF)/M06/Def2-TZVPP//B3LYP-D3/Def2-SVP) = -1175.04023039

Zero-point correction=	0.182516 (Hartree/Particle)
Thermal correction to Energy=	0.197611
Thermal correction to Enthalpy=	0.198555
Thermal correction to Gibbs Free Energy=	0.139127

Charge = 0 Multiplicity = 1

C	-1.71673100	-0.33559700	0.60984700
C	-0.22196200	-0.33882500	0.34914000
C	-5.19772000	-0.17192000	-0.21893900
C	-4.00587300	-0.15609900	-0.44257300
C	-2.56239000	-0.14676600	-0.67647500
H	-1.97843500	0.47701800	1.30355900
H	-6.25337800	-0.18313100	-0.02613700
H	-2.27192200	0.80961000	-1.14226800
H	-2.30520200	-0.93813500	-1.40293200
H	-2.03557700	-1.26514800	1.10209500
C	0.49752700	-1.55598500	0.19417900
C	0.43034100	0.92592500	0.19378800
O	1.73872300	-1.65713400	-0.02140700

O	1.63527300	1.17658400	-0.00379000
O	-0.43910900	1.98629200	0.27319600
C	-0.26950300	-2.87688900	0.29661800
H	0.41321100	-3.69178500	0.02114200
H	-0.62683300	-3.04982800	1.32576200
H	-1.15292500	-2.90619200	-0.36054500
C	0.11696100	3.28109000	0.14503300
H	-0.71990600	3.98696200	0.23946000
H	0.86445100	3.48290000	0.93054000
H	0.61034400	3.41748000	-0.83195600
K	3.66069300	-0.20712000	-0.30770600

K-enolate of compound 1 (open form)

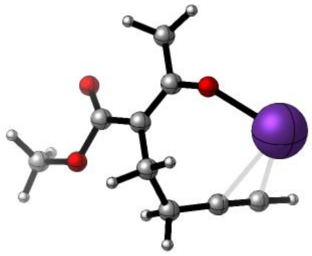

E (B3LYP-D3/Def2-SVP)) = -1174.70532075
E (SMD(DMF)/M06/Def2-TZVPP//B3LYP-D3/Def2-SVP) = -1175.03452721

Zero-point correction=	0.182037 (Hartree/Particle)
Thermal correction to Energy=	0.197149
Thermal correction to Enthalpy=	0.198093
Thermal correction to Gibbs Free Energy=	0.138551

Charge = 0 Multiplicity = 1

C	0.14368900	-0.80587500	-1.16758700
C	0.66904200	0.38297000	-0.39987200
C	-2.09432700	-1.99921000	1.48680900
C	-1.19985900	-2.01382800	0.66082100
C	-0.12713600	-2.08694600	-0.32945500
H	0.87374000	-1.11134000	-1.93350100
H	-2.80159600	-2.04589700	2.29480700
H	-0.34704300	-2.93698800	-1.00219100
H	0.80781400	-2.33936000	0.19785500
H	-0.77539300	-0.51553400	-1.70244400
C	-0.18760500	1.46400000	-0.12085800
C	2.05021000	0.37967100	0.02601300
O	-1.42806200	1.44633800	-0.43612500
O	2.67932800	1.24161100	0.62249700
O	2.68208600	-0.81074100	-0.31216500
C	0.31372300	2.71478800	0.58128300
H	-0.51411800	3.43622100	0.64008500
H	0.68688800	2.47861100	1.58936600
H	1.17266700	3.15648900	0.05593100
C	4.04602200	-0.90149700	0.05816700
H	4.39546300	-1.88966200	-0.27426200
H	4.64883100	-0.11093400	-0.41896400
H	4.17808400	-0.80320400	1.14865600
K	-3.44603700	0.25576300	-0.22952200

TS, M=K, 5-endo carbocyclizaton

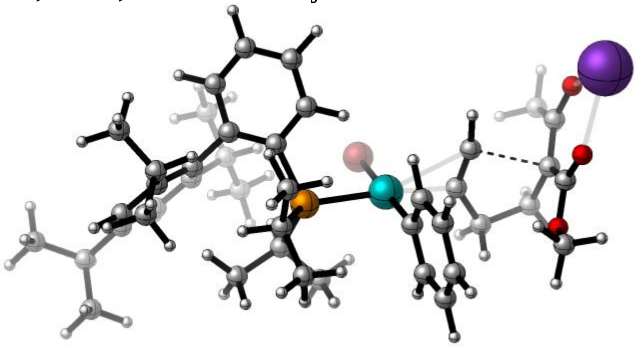

E (B3LYP-D3/Def2-SVP)) = -5502.30818339
E (SMD(DMF)/M06/Def2-TZVPP//B3LYP-D3/Def2-SVP) = -5503.18580005

Zero-point correction=	0.889774 (Hartree/Particle)
Thermal correction to Energy=	0.947362
Thermal correction to Enthalpy=	0.948306
Thermal correction to Gibbs Free Energy=	0.793706

Charge = 0 Multiplicity = 1

Pd	-1.20912300	0.05095400	-0.36634800
P	1.03622400	0.72099800	-0.04539000
C	1.55720200	2.47210500	0.40252900
H	2.65545900	2.42143800	0.35123900
C	1.97278700	0.32957500	-1.63978200
H	2.24296000	-0.72196700	-1.48749600
C	1.66870800	-0.24337800	1.42378100
C	2.97605000	-0.70566000	1.70390900
C	0.66404000	-0.45975100	2.39053600
C	3.21212000	-1.36194400	2.92867400
C	0.92068100	-1.09364600	3.60291000
H	-0.35416700	-0.13106100	2.16991500
C	2.20938400	-1.55467700	3.87562600
H	4.22146100	-1.72923300	3.12726200
H	0.11095000	-1.24131300	4.32162800
H	2.43260100	-2.06879500	4.81397400
C	4.16360300	-0.57173800	0.79752700
C	5.08016900	0.48998200	0.99120500
C	4.41538500	-1.56040000	-0.18322400
C	6.22158700	0.55576500	0.18218000
C	5.56325000	-1.43783000	-0.97800100
C	6.47582600	-0.39054800	-0.81691600
H	6.92571400	1.37789300	0.32592100
H	5.75766900	-2.18546700	-1.75033600
C	-1.96719700	1.91862600	-0.34272600
C	-2.39030300	2.54657600	0.84197400
C	-2.15818100	2.61182400	-1.55243500
C	-2.95476300	3.82913500	0.82433900
H	-2.27597400	2.03347400	1.80160400
C	-2.72453600	3.89231900	-1.57563700
H	-1.84634300	2.15558400	-2.49656400
C	-3.11895300	4.51228500	-0.38532400
H	-3.26366200	4.29875000	1.76357500

H	-2.85203700	4.41088400	-2.53086400
H	-3.55402100	5.51520100	-0.40052000
C	4.86160900	1.54847100	2.07039300
H	3.80347600	1.50353700	2.36727000
C	7.69074800	-0.26727700	-1.72493700
H	7.70093300	-1.15944100	-2.37509700
C	3.49226800	-2.76574100	-0.34651500
H	2.48982300	-2.48107300	0.00647500
C	5.69978800	1.23608600	3.32265100
H	6.77543100	1.23742700	3.08004500
H	5.52635900	1.98906100	4.10933800
H	5.44922300	0.24841900	3.73606300
C	5.13891800	2.97983800	1.58284800
H	4.80811400	3.71083800	2.33809400
H	6.21301700	3.15406100	1.41006200
H	4.61225000	3.20010000	0.64155500
C	3.32739800	-3.23559500	-1.79912900
H	2.49658200	-3.95428700	-1.86104700
H	3.08887300	-2.40044900	-2.47553200
H	4.23473300	-3.73210300	-2.18143200
C	3.96925100	-3.93216600	0.53829300
H	4.01094200	-3.64370100	1.59858400
H	3.28047100	-4.78766700	0.44806000
H	4.97647800	-4.26607200	0.23660600
C	9.00958200	-0.25634500	-0.93581100
H	9.87431500	-0.23269800	-1.61917800
H	9.07924500	0.62979400	-0.28370400
H	9.09965800	-1.14923100	-0.29751000
C	7.57579800	0.96706600	-2.63612900
H	6.64475400	0.94139700	-3.22385900
H	7.56875100	1.89599900	-2.04214800
H	8.42540800	1.02207500	-3.33673800
C	3.23759700	1.14483400	-1.93343500
H	2.99710500	2.18161700	-2.21239800
H	3.94548200	1.16777800	-1.10025200
H	3.76311100	0.69008000	-2.78846400
C	1.01793400	0.38369400	-2.84576500
H	0.18162000	-0.31986900	-2.73282100
H	0.60748900	1.39443000	-3.00192800
H	1.57420700	0.10708700	-3.75710400
C	1.16391400	2.87354900	1.82894200
H	1.58069600	2.19888600	2.58972600
H	0.07017900	2.89273400	1.94575000
C	1.06611200	3.52266400	-0.60400900
H	0.00086300	3.74259100	-0.45944100
H	1.62959800	4.45944000	-0.45664300
H	1.20079600	3.21630800	-1.65038000
H	1.53861800	3.88985000	2.03712900
Br	-0.35077600	-2.37750400	-0.59920200
C	-3.14993600	-0.60144900	-0.88100300
C	-3.69425900	-0.76146300	0.24158000
C	-3.68049100	-0.68920700	-2.25746100
H	-3.87862200	-0.81919200	1.30043700
C	-5.10912900	-1.34494700	-2.20042600

H	-3.00072400	-1.26454600	-2.90478600
H	-3.73447300	0.33410600	-2.66085200
C	-5.81815500	-1.16480500	-0.87659600
H	-5.70569800	-0.90662700	-3.01523900
H	-5.00124300	-2.41192800	-2.42654400
C	-6.09256500	-2.27010600	-0.00035400
C	-6.25772400	0.17696900	-0.54464400
O	-6.71069400	-2.17609200	1.09094800
C	-5.57474900	-3.65459700	-0.37671200
O	-6.83423700	0.57313800	0.47969900
O	-5.92417800	1.07474800	-1.50356800
H	-5.81787100	-4.34433200	0.44201500
H	-6.04257300	-4.02405700	-1.30368300
H	-4.48480500	-3.64738300	-0.53382500
C	-6.08564600	2.45270200	-1.18936200
H	-5.74820400	3.00753800	-2.07349700
H	-7.13706500	2.69229100	-0.96438800
H	-5.45877600	2.73287600	-0.32896000
K	-7.72545000	-0.48823100	2.53672800

TS, M=K, 6-exo oxocyclizaton

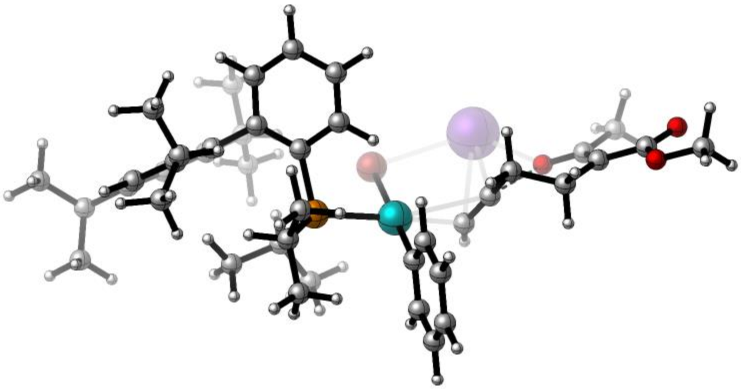

E (B3LYP-D3/Def2-SVP)) = -5502.31584168
E (SMD(DMF)/M06/Def2-TZVPP//B3LYP-D3/Def2-SVP) = -5503.18971969

Zero-point correction=	0.889521 (Hartree/Particle)
Thermal correction to Energy=	0.947272
Thermal correction to Enthalpy=	0.948216
Thermal correction to Gibbs Free Energy=	0.791674

Charge = 0 Multiplicity = 1

P	1.15686500	0.88172300	-0.30693800
C	1.80838800	2.60303000	0.07717200
H	2.89444200	2.43123300	0.12516400
C	2.22397200	0.27865100	-1.74829200
H	2.37319800	-0.77755600	-1.49287600
C	1.50911000	-0.03213700	1.28265700
C	2.72610900	-0.57983900	1.75501500
C	0.37700000	-0.09713000	2.12273500
C	2.74603400	-1.17166300	3.03372000
C	0.42186800	-0.66930600	3.39142100
H	-0.57096000	0.30712800	1.75875300
C	1.61951900	-1.21826700	3.85181500

H	3.68621900	-1.60334500	3.38387400
H	-0.47858800	-0.69339400	4.00998900
H	1.67795900	-1.68301600	4.83925300
C	4.02684500	-0.59534900	1.00692100
C	4.99182700	0.41205300	1.25236800
C	4.32547700	-1.66855400	0.13432300
C	6.22574200	0.34209100	0.59361300
C	5.56911500	-1.68224900	-0.51161800
C	6.53031700	-0.68850900	-0.30285100
H	6.96788100	1.12159200	0.77700100
H	5.80279900	-2.49708400	-1.20056600
C	-1.64131400	2.28630700	-0.95264200
C	-1.98939500	3.01537600	0.19517300
C	-1.74141900	2.91958600	-2.20291800
C	-2.41940300	4.34623300	0.09927800
H	-1.91665100	2.55532600	1.18460100
C	-2.16784200	4.24928800	-2.29873200
H	-1.48096200	2.37731500	-3.11574000
C	-2.50509500	4.97023500	-1.14789500
H	-2.68289800	4.89582900	1.00763700
H	-2.23739600	4.72439600	-3.28159900
H	-2.83682100	6.00881700	-1.22401900
C	4.73046600	1.54907500	2.23774900
H	3.64428700	1.60446400	2.39835000
C	7.85587800	-0.71429600	-1.04943300
H	7.87591600	-1.64571400	-1.64170900
C	3.34729300	-2.82413100	-0.06350600
H	2.33511300	-2.45489900	0.15699400
C	5.37779300	1.24815300	3.60136600
H	6.47146300	1.15322700	3.49915800
H	5.17104400	2.05754200	4.32080600
H	4.99721700	0.30879200	4.02802300
C	5.18615300	2.92296900	1.72042300
H	4.82838700	3.72012800	2.39144100
H	6.28408700	3.00266100	1.67811200
H	4.80020900	3.12920700	0.71027600
C	3.31498600	-3.37857400	-1.49521800
H	2.45912600	-4.06102200	-1.60873000
H	3.19926600	-2.57905700	-2.24290700
H	4.22940400	-3.94226700	-1.74243900
C	3.64483100	-3.95546900	0.93796000
H	3.58706600	-3.60045700	1.97708700
H	2.91822400	-4.77587700	0.81954600
H	4.65565300	-4.36547000	0.77543700
C	9.05985300	-0.74843100	-0.09457600
H	10.00327200	-0.83294600	-0.65827500
H	9.11765500	0.17103200	0.51080200
H	8.99549500	-1.60193000	0.59834000
C	7.95811400	0.46259200	-2.03496900
H	7.11039400	0.46737500	-2.73808200
H	7.95294400	1.42677100	-1.50019700
H	8.89108500	0.40779800	-2.61963500
C	3.58887000	0.95015500	-1.94257800
H	3.48468600	1.97867200	-2.31878000

H	4.19797900	0.97424200	-1.03484200
H	4.15812400	0.38286900	-2.69607300
C	1.42578800	0.32788600	-3.06371200
H	0.51384700	-0.28275100	-3.01528700
H	1.13919800	1.35704300	-3.33290300
H	2.05373400	-0.06635800	-3.87994600
C	1.36116100	3.15406400	1.43592200
H	1.58941700	2.47366500	2.26794400
H	0.28264200	3.36215500	1.44560500
C	1.52052000	3.61571700	-1.04051900
H	0.47297700	3.94233200	-1.02164900
H	2.15639200	4.50512900	-0.89648300
H	1.72710400	3.22057900	-2.04470300
H	1.88440100	4.10572700	1.62682000
Br	-0.36731000	-2.22279000	-0.88091600
Pd	-1.08363200	0.36981400	-0.86406400
C	-3.01580200	-0.27525700	-1.55872500
C	-3.77603300	-0.18830800	-0.55921500
H	-3.00467300	-0.75263900	-2.53349500
C	-4.39615100	0.35345200	0.64134900
C	-5.88315300	0.73195800	0.43757600
H	-4.29766200	-0.34871800	1.48692200
H	-3.82799900	1.26563000	0.88878700
C	-6.84730000	-0.43652600	0.41699200
H	-5.95353000	1.34124300	-0.48317500
H	-6.15655700	1.40508900	1.26047900
C	-6.46975100	-1.65200400	-0.15005200
C	-8.18634900	-0.25300900	0.95384800
O	-5.29507700	-1.83888800	-0.66201200
C	-7.39267700	-2.85904600	-0.19107500
O	-8.39605100	1.03286600	1.39605100
O	-9.08495300	-1.07753900	1.04130000
H	-7.70425700	-3.16302100	0.81908300
H	-8.32787800	-2.62759900	-0.72062500
H	-6.87659600	-3.68846000	-0.69958300
C	-9.67947800	1.29333700	1.93785100
H	-9.88624100	0.65549000	2.81336600
H	-9.68262300	2.35147100	2.23494600
H	-10.47499700	1.10752400	1.19755400
K	-3.30556000	-3.35220900	-0.55367400

Na-enolate of compound 1

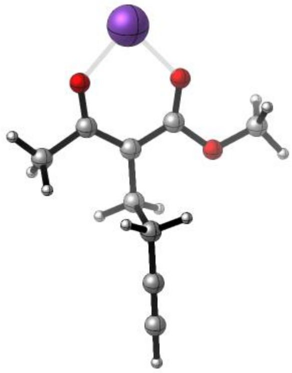

E (B3LYP-D3/Def2-SVP)) = -737.107085632

E (SMD(DMF)/M06/Def2-TZVPP//B3LYP-D3/Def2-SVP) = -737.418633580

Zero-point correction=	0.183264 (Hartree/Particle)
Thermal correction to Energy=	0.198012
Thermal correction to Enthalpy=	0.198956
Thermal correction to Gibbs Free Energy=	0.140946

Charge = 0 Multiplicity = 1

C	-1.36765100	-0.30940700	0.61259600
C	0.11905200	-0.35909000	0.31033400
C	-4.86200200	-0.03962900	-0.11613500
C	-3.67729400	-0.05924800	-0.37389400
C	-2.24161800	-0.09292800	-0.64951300
H	-1.58311200	0.50975400	1.31411600
H	-5.91174800	-0.01956100	0.10639900
H	-1.93626500	0.85480800	-1.12343800
H	-2.02936000	-0.89045700	-1.38366500
H	-1.70060000	-1.22878700	1.11384200
C	0.79018000	-1.60025000	0.13349700
C	0.80144600	0.88831000	0.14249200
O	2.02350800	-1.75265100	-0.11526300
O	2.01105500	1.10782300	-0.08875000
O	-0.02687300	1.96991300	0.25056900
C	-0.01723200	-2.89256200	0.25239500
H	0.62774600	-3.72918000	-0.04676200
H	-0.34830400	-3.05741300	1.29144700
H	-0.92053400	-2.88688900	-0.37731600
C	0.56212700	3.25160000	0.11265600
H	-0.25288600	3.97787700	0.23506000
H	1.33621900	3.42595900	0.87791100
H	1.02965500	3.37786800	-0.87766300
Na	3.54414700	-0.33231300	-0.36808600

Na-enolate of compound 1 (open form)

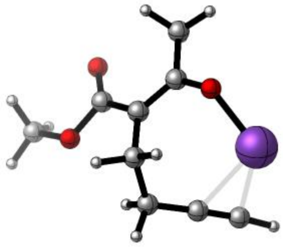

E (B3LYP-D3/Def2-SVP)) = -737.087407954
E (SMD(DMF)/M06/Def2-TZVPP//B3LYP-D3/Def2-SVP) = -737.408658519

Zero-point correction=	0.182466 (Hartree/Particle)
Thermal correction to Energy=	0.197300
Thermal correction to Enthalpy=	0.198244
Thermal correction to Gibbs Free Energy=	0.139959

Charge = 0 Multiplicity = 1

C	-0.19698800	-0.76334500	-1.20305100
C	0.38565400	0.39105300	-0.42195400
C	-2.71646300	-1.79824100	1.25468800

C	-1.74643300	-1.87830300	0.51989500
C	-0.59900700	-2.01853100	-0.37400900
H	0.54242800	-1.12952500	-1.93175300
H	-3.50168200	-1.83859000	1.98941900
H	-0.81255300	-2.86467200	-1.05278500
H	0.27494500	-2.31024600	0.23136400
H	-1.06462300	-0.41303000	-1.78651600
C	-0.37901900	1.55208400	-0.20621600
C	1.74626300	0.27993500	0.06070300
O	-1.61073000	1.63819900	-0.55720400
O	2.42139200	1.09938400	0.66419200
O	2.28257400	-0.96784900	-0.22439200
C	0.21035000	2.78086600	0.46300300
H	-0.54890500	3.57567400	0.45922800
H	0.52292800	2.55578000	1.49396700
H	1.12517200	3.11829300	-0.04469100
C	3.61996800	-1.16768900	0.20101100
H	3.89244200	-2.19064100	-0.09540200
H	4.30574000	-0.44492500	-0.27091900
H	3.71878700	-1.05255700	1.29311400
Na	-3.19513800	0.41541800	-0.10092800

TS, M=Na, 5-endo carbocyclizaton

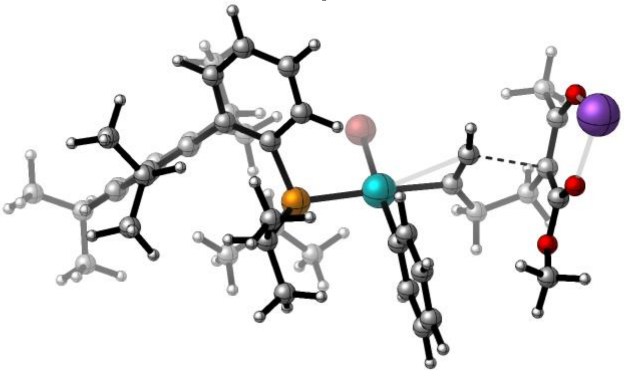

E (B3LYP-D3/Def2-SVP)) = -5064.69232581
E (SMD(DMF)/M06/Def2-TZVPP//B3LYP-D3/Def2-SVP) = -5065.56318827

Zero-point correction= 0.890460 (Hartree/Particle)
Thermal correction to Energy= 0.947696
Thermal correction to Enthalpy= 0.948640
Thermal correction to Gibbs Free Energy= 0.795492

Charge = 0 Multiplicity = 1

Pd	-1.36210900	0.04390500	-0.27603500
P	0.89002400	0.72316000	-0.02523500
C	1.41588700	2.48088700	0.38856600
H	2.51273800	2.43434700	0.31004200
C	1.78358400	0.31580100	-1.63984200
H	2.06036000	-0.73343000	-1.48344900
C	1.56367700	-0.22337600	1.43677400
C	2.87897000	-0.68058600	1.68615300
C	0.58604600	-0.43046800	2.43286100
C	3.14967300	-1.32241100	2.91135300

C	0.87690100	-1.05010600	3.64490800
H	-0.43845900	-0.10595300	2.23649100
C	2.17350900	-1.50574700	3.88752900
H	4.16479300	-1.68570600	3.08644600
H	0.08743800	-1.19074800	4.38716400
H	2.42325400	-2.00851800	4.82532700
C	4.04067500	-0.55535000	0.74581500
C	4.96029800	0.50991500	0.90184000
C	4.26694300	-1.55476900	-0.23023700
C	6.07898100	0.56789900	0.06109400
C	5.39233200	-1.43978100	-1.05762800
C	6.30734200	-0.38957700	-0.93361300
H	6.78540800	1.39256000	0.17620800
H	5.56682500	-2.19596900	-1.82638400
C	-2.12887700	1.90831700	-0.26027100
C	-2.53020500	2.55057900	0.92454000
C	-2.35161600	2.58329800	-1.47512700
C	-3.10427600	3.82882000	0.90137700
H	-2.39089500	2.05235000	1.88865200
C	-2.92777900	3.85941000	-1.50385200
H	-2.05616700	2.11668700	-2.41957400
C	-3.30050500	4.49347100	-0.31391900
H	-3.39553800	4.30956000	1.84055700
H	-3.07921500	4.36383000	-2.46318500
H	-3.74261800	5.49322500	-0.33344500
C	4.76950700	1.58055700	1.97429300
H	3.72007100	1.53717600	2.30090800
C	7.49695000	-0.27541700	-1.87566700
H	7.49098200	-1.17520000	-2.51530400
C	3.34160800	-2.76312100	-0.35455000
H	2.34869100	-2.47582200	0.02247100
C	5.64269700	1.28427000	3.20638300
H	6.71117500	1.28438400	2.93395500
H	5.48978200	2.04621900	3.98865500
H	5.40556300	0.30119200	3.63824900
C	5.03030200	3.00657700	1.46254200
H	4.71892800	3.74585600	2.21797600
H	6.09886400	3.18063500	1.25819400
H	4.47750300	3.21483900	0.53356300
C	3.13806200	-3.24979200	-1.79669400
H	2.30779400	-3.97113200	-1.82763900
H	2.87901500	-2.42294200	-2.47576400
H	4.03581800	-3.74857700	-2.19815300
C	3.84439100	-3.91878200	0.53012700
H	3.91438500	-3.61826000	1.58555600
H	3.15491100	-4.77624400	0.46834600
H	4.84360600	-4.25464900	0.20495000
C	8.83667000	-0.25342300	-1.12277900
H	9.68248700	-0.23688100	-1.82961100
H	8.92264600	0.64053900	-0.48342200
H	8.94542100	-1.13850600	-0.47657600
C	7.35531200	0.94790800	-2.79789300
H	6.40884700	0.91394400	-3.36002800
H	7.36260100	1.88378100	-2.21491700

H	8.18565200	0.99586700	-3.52172400
C	3.03804800	1.13028700	-1.97715800
H	2.78737500	2.16311600	-2.26174500
H	3.76831400	1.16468500	-1.16384000
H	3.54135800	0.66659900	-2.84070200
C	0.79560400	0.35464500	-2.81948600
H	-0.03474500	-0.35049700	-2.67627000
H	0.37710900	1.36236600	-2.97381000
H	1.32732500	0.07103200	-3.74323700
C	1.05638900	2.89680600	1.81976900
H	1.49631000	2.23345300	2.57748400
H	-0.03422300	2.91091500	1.96400100
C	0.89456100	3.51755900	-0.61714400
H	-0.16806800	3.73337700	-0.44886100
H	1.45610300	4.45893500	-0.49380100
H	1.00562300	3.20038200	-1.66306700
H	1.42994300	3.91761100	2.00676800
Br	-0.51253900	-2.38723500	-0.50608700
C	-3.31040900	-0.62101200	-0.70802300
C	-3.85967700	-0.76995300	0.41770100
C	-3.87270000	-0.74639400	-2.07055700
H	-4.00935300	-0.80258400	1.48399300
C	-5.29329700	-1.40985300	-1.96175000
H	-3.20402900	-1.33434800	-2.71788100
H	-3.94166200	0.26559300	-2.49952800
C	-5.95579600	-1.21326000	-0.61519300
H	-5.92355800	-0.98863000	-2.75966800
H	-5.18721600	-2.47942100	-2.17463300
C	-6.20109500	-2.31248600	0.27866700
C	-6.40476700	0.13185400	-0.30060000
O	-6.79493500	-2.21650400	1.38625900
C	-5.67931800	-3.69400700	-0.09162700
O	-6.96174600	0.54288200	0.73332900
O	-6.10966200	1.01137600	-1.27800500
H	-5.88526500	-4.37362200	0.74519200
H	-6.17488000	-4.08261600	-0.99592700
H	-4.59590200	-3.67446700	-0.28693500
C	-6.26827100	2.39741900	-0.98628400
H	-5.96459200	2.93308500	-1.89367100
H	-7.31244400	2.63396900	-0.72967400
H	-5.61121800	2.69534800	-0.15534400
Na	-7.61152100	-0.57347700	2.42443000

TS, M=Na, 6-exo oxocyclizaton

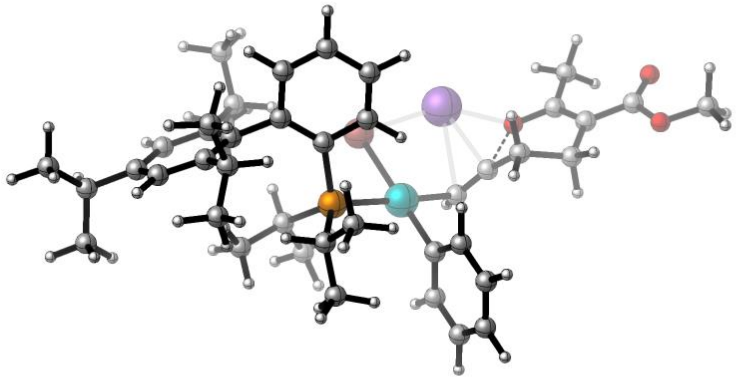

E (B3LYP-D3/Def2-SVP)) = -5064.70132012
E (SMD(DMF)/M06/Def2-TZVPP//B3LYP-D3/Def2-SVP) = -5065.56259186

Zero-point correction=	0.890416 (Hartree/Particle)
Thermal correction to Energy=	0.947720
Thermal correction to Enthalpy=	0.948664
Thermal correction to Gibbs Free Energy=	0.794604

Charge = 0 Multiplicity = 1

P	1.09334600	0.91529700	-0.27014200
C	1.78436400	2.57464100	0.27790900
H	2.86314200	2.36691600	0.34346600
C	2.18291400	0.39564900	-1.72702000
H	2.29530000	-0.68176500	-1.55466000
C	1.36348000	-0.14786400	1.24052200
C	2.54478000	-0.78390400	1.69260800
C	0.20364200	-0.24844900	2.03840900
C	2.50289200	-1.49480300	2.90845800
C	0.18752400	-0.94052800	3.24660900
H	-0.71762700	0.22453900	1.68914100
C	1.34958200	-1.57651600	3.68547900
H	3.41556300	-1.99315800	3.24232100
H	-0.73276700	-0.99002200	3.83354400
H	1.35947000	-2.13611600	4.62413900
C	3.86767400	-0.77591400	0.98449000
C	4.85370400	0.17394900	1.34753200
C	4.16224700	-1.77702300	0.02890200
C	6.10547100	0.12269000	0.72161500
C	5.42528900	-1.77429800	-0.57835000
C	6.40819900	-0.83432200	-0.25364100
H	6.86412700	0.85850900	0.99539800
H	5.65694100	-2.53272700	-1.32958200
C	-1.65869800	2.45992800	-0.85513400
C	-2.03145600	3.08217700	0.34639800
C	-1.69869500	3.21237300	-2.04077800
C	-2.42477400	4.42764200	0.36609000
H	-2.00610900	2.52633300	1.28775800
C	-2.08931600	4.55635100	-2.02024200
H	-1.41799400	2.75375100	-2.99253100
C	-2.45009200	5.17154500	-0.81640100
H	-2.70716800	4.89401800	1.31442700

H	-2.11164800	5.12607100	-2.95376900
H	-2.75306300	6.22152400	-0.80173500
C	4.59476800	1.22514800	2.42453100
H	3.50594800	1.30363800	2.55520600
C	7.75497300	-0.83590300	-0.96171900
H	7.77026400	-1.71700700	-1.62663900
C	3.15667700	-2.87786700	-0.30119700
H	2.15007000	-2.49836300	-0.07252100
C	5.18428300	0.77831400	3.77435600
H	6.27731600	0.65549700	3.69860600
H	4.97805200	1.52499200	4.55881000
H	4.75978700	-0.18252100	4.09969200
C	5.11210100	2.62361500	2.05127200
H	4.75662700	3.36827800	2.78121000
H	6.21282000	2.66823500	2.05195900
H	4.76900800	2.93463700	1.05252400
C	3.14953400	-3.29664500	-1.77878700
H	2.27941000	-3.94053400	-1.97738800
H	3.07787100	-2.42874200	-2.45215300
H	4.05475600	-3.86231700	-2.05343300
C	3.39140200	-4.10595700	0.59775200
H	3.31447800	-3.84682900	1.66358900
H	2.64459800	-4.88804800	0.38466100
H	4.39395900	-4.53109800	0.42370300
C	8.92894500	-0.97790600	0.02002500
H	9.88599300	-1.04353000	-0.52275700
H	8.99238200	-0.11084800	0.69779700
H	8.82303200	-1.88122900	0.64090800
C	7.91446800	0.41323300	-1.84566800
H	7.08899500	0.49596900	-2.57009100
H	7.91504700	1.33112000	-1.23477500
H	8.86334300	0.38178500	-2.40593800
C	3.57207700	1.03735100	-1.82827400
H	3.50884100	2.09655800	-2.11868000
H	4.15464700	0.96628300	-0.90560300
H	4.14634100	0.51731800	-2.61142500
C	1.42518100	0.57590300	-3.05484000
H	0.49500300	-0.00822800	-3.08254100
H	1.17534300	1.63179100	-3.24561100
H	2.06501700	0.23081400	-3.88393200
C	1.30541400	3.02083800	1.66397900
H	1.48670000	2.26533000	2.44094600
H	0.23277100	3.25850100	1.65614800
C	1.55977000	3.68621800	-0.75724200
H	0.52100600	4.03987700	-0.74186400
H	2.21335600	4.54165900	-0.51836200
H	1.78852600	3.37232800	-1.78507400
H	1.84634100	3.93711500	1.95318000
Br	-0.53132100	-2.10496700	-1.14902000
Pd	-1.14832800	0.53302200	-0.92964900
C	-3.07686900	-0.00914100	-1.67658000
C	-3.84174200	-0.09518900	-0.67090800
H	-3.08183600	-0.40872900	-2.68812700
C	-4.43806500	0.32084200	0.59377700

C	-5.96693000	0.53090800	0.48893500
H	-4.21512200	-0.40916800	1.39106500
H	-3.95611100	1.27622400	0.85903200
C	-6.77241500	-0.74859200	0.39546200
H	-6.16292600	1.20169800	-0.36823600
H	-6.27952900	1.08665100	1.38226900
C	-6.27488400	-1.83970400	-0.30177900
C	-8.09179400	-0.80575700	1.01373900
O	-5.11676200	-1.79820600	-0.90105100
C	-7.00666100	-3.16239500	-0.41779800
O	-8.43825200	0.38487700	1.60091600
O	-8.86307400	-1.75175000	1.04824400
H	-7.23114600	-3.58649900	0.57147900
H	-7.98459800	-3.02983500	-0.90261500
H	-6.39594900	-3.86392700	-1.00642700
C	-9.71246100	0.41531600	2.22409900
H	-9.78279700	-0.32977700	3.03363000
H	-9.83380400	1.42800700	2.63268300
H	-10.51738500	0.19996800	1.50257800
Na	-3.18998600	-2.74678600	-1.03528300

Li-enolate of compound 1

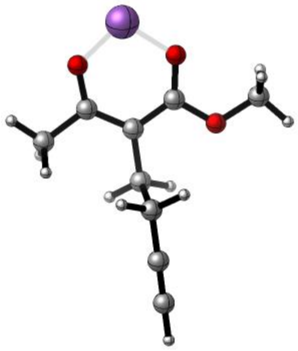

E (B3LYP-D3/Def2-SVP)) = -582.406667202
E (SMD(DMF)/M06/Def2-TZVPP//B3LYP-D3/Def2-SVP) = -582.678052942

Zero-point correction=	0.185313 (Hartree/Particle)
Thermal correction to Energy=	0.199272
Thermal correction to Enthalpy=	0.200216
Thermal correction to Gibbs Free Energy=	0.144576

Charge = 0 Multiplicity = 1

C	-1.07272000	-0.25824600	0.60988300
C	0.39313000	-0.42396800	0.26094500
C	-4.54994900	0.30794400	-0.00644100
C	-3.37984000	0.19230700	-0.30162000
C	-1.96144400	0.04245300	-0.62350400
H	-1.19680700	0.56783800	1.32549600
H	-5.58722600	0.41311200	0.24881900
H	-1.59348700	0.96597600	-1.10114700
H	-1.83869900	-0.76294300	-1.36937500
H	-1.46156000	-1.15458500	1.11285100
C	0.97096400	-1.70286300	0.05571300
C	1.17709800	0.75720500	0.07834000

O	2.19204800	-1.91516200	-0.23839400
O	2.40017000	0.84128300	-0.20190900
O	0.48126700	1.91043800	0.22630400
C	0.10230600	-2.94469700	0.19416700
H	0.68669300	-3.81543700	-0.13004000
H	-0.20366700	-3.09513000	1.24290600
H	-0.81887100	-2.87939400	-0.40524900
C	1.19585200	3.12908000	0.07073500
H	0.46475300	3.93241500	0.23056000
H	2.01255000	3.21120800	0.80549700
H	1.63211800	3.21162000	-0.93748200
Li	3.35464600	-0.62748100	-0.44671800

Li-enolate of compound 1 (open form)

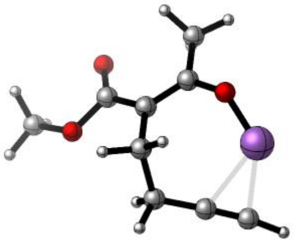

E (B3LYP-D3/Def2-SVP)) = -582.378723845
E (SMD(DMF)/M06/Def2-TZVPP//B3LYP-D3/Def2-SVP) = -582.659641307

Zero-point correction=	0.184020 (Hartree/Particle)
Thermal correction to Energy=	0.198280
Thermal correction to Enthalpy=	0.199224
Thermal correction to Gibbs Free Energy=	0.142989

Charge = 0 Multiplicity = 1

C	-0.48609900	-0.80680700	-1.11676000
C	0.21041800	0.34704200	-0.43790100
C	-3.48047900	-1.17979200	0.95628500
C	-2.40237000	-1.46496300	0.46079100
C	-1.14331100	-1.85710300	-0.17041500
H	0.24280800	-1.37020600	-1.71495800
H	-4.41248000	-1.07307800	1.48525600
H	-1.35112800	-2.78667900	-0.73109300
H	-0.41337100	-2.11488200	0.61424800
H	-1.24947600	-0.42418500	-1.81412200
C	-0.47826400	1.54308800	-0.22562500
C	1.57695600	0.16942700	0.02214300
O	-1.73969200	1.64357900	-0.49381800
O	2.32630300	0.99262700	0.52082200
O	1.99347800	-1.13828100	-0.13314800
C	0.18375300	2.78128600	0.33965200
H	-0.54289000	3.60557100	0.32665500
H	0.53373200	2.60135700	1.36782900
H	1.08603900	3.05052300	-0.22699300
C	3.32076900	-1.41162700	0.28773000
H	3.49425800	-2.47995200	0.09728600
H	4.05267000	-0.80577800	-0.27102600
H	3.45647400	-1.19184600	1.35940700

Li	-3.11519300	0.76081800	-0.11291000

TS, M=Li, 5-endo carbocyclizaton

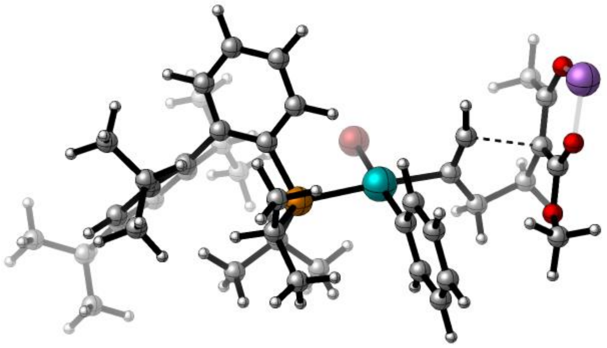

E (B3LYP-D3/Def2-SVP)) = -4909.98861745
E (SMD(DMF)/M06/Def2-TZVPP//B3LYP-D3/Def2-SVP) = -4910.82023332

Zero-point correction=	0.892377 (Hartree/Particle)
Thermal correction to Energy=	0.948798
Thermal correction to Enthalpy=	0.949743
Thermal correction to Gibbs Free Energy=	0.799038

Charge = 0 Multiplicity = 1

Pd	-1.50688600	0.02624000	-0.20480600
P	0.74740800	0.72273800	-0.01059000
C	1.26691800	2.48902600	0.37293600
H	2.36250000	2.45261200	0.27356100
C	1.60987300	0.30336600	-1.63865100
H	1.89660200	-0.74245300	-1.47743400
C	1.45611700	-0.20397500	1.44708000
C	2.77830400	-0.65312400	1.67335000
C	0.50057000	-0.40440400	2.46576500
C	3.07771600	-1.28035000	2.89936900
C	0.81965500	-1.00981600	3.67794600
H	-0.52929900	-0.08606200	2.28818100
C	2.12314600	-1.45721500	3.89789300
H	4.09803800	-1.63705200	3.05690000
H	0.04672500	-1.14551100	4.43826900
H	2.39487200	-1.94852300	4.83564000
C	3.91913100	-0.53320700	0.70725700
C	4.83671600	0.53790300	0.83212400
C	4.12956300	-1.54241300	-0.26221100
C	5.93786300	0.59089700	-0.03175800
C	5.23740600	-1.43218300	-1.11359800
C	6.15035300	-0.37705100	-1.01982400
H	6.64288500	1.41972100	0.05968600
H	5.39974500	-2.19626400	-1.87720300
C	-2.29337500	1.88297000	-0.19694900
C	-2.67850100	2.53766000	0.98678900
C	-2.54954500	2.53876400	-1.41615500
C	-3.26795900	3.80884600	0.95787900
H	-2.51342900	2.05526200	1.95483000
C	-3.14109400	3.80780800	-1.45076000
H	-2.26695600	2.06301000	-2.36010700
C	-3.49696800	4.45426700	-0.26223000

H	-3.54507300	4.29936000	1.89622400
H	-3.31675200	4.29752400	-2.41362700
H	-3.95006400	5.44899900	-0.28622200
C	4.66235800	1.62036000	1.89546200
H	3.61981400	1.57619000	2.24346400
C	7.32084200	-0.26937700	-1.98625400
H	7.30552200	-1.17619500	-2.61572900
C	3.20679600	-2.75567100	-0.35445300
H	2.22071900	-2.46812100	0.03997700
C	5.56133900	1.34286400	3.11328100
H	6.62412200	1.34429600	2.81945800
H	5.42062000	2.11360600	3.88918000
H	5.33740300	0.36412800	3.56174100
C	4.90608900	3.04125200	1.36149200
H	4.60620200	3.78821200	2.11400000
H	5.96953300	3.21773200	1.13387800
H	4.33407600	3.23569000	0.44116500
C	2.97560800	-3.25863300	-1.78682000
H	2.14844100	-3.98419300	-1.79283300
H	2.69868600	-2.44030300	-2.46918300
H	3.86717500	-3.75761700	-2.20154400
C	3.73231100	-3.89983600	0.53195300
H	3.82272200	-3.58773000	1.58243900
H	3.04530400	-4.76064600	0.49351200
H	4.72600600	-4.23521700	0.18987200
C	8.67487900	-0.23480700	-1.25989200
H	9.50670700	-0.22325700	-1.98321700
H	8.77017800	0.66635800	-0.63207800
H	8.79923400	-1.11241600	-0.60636000
C	7.15710900	0.94321600	-2.91891600
H	6.19998900	0.90004900	-3.46208600
H	7.17268000	1.88552300	-2.34657300
H	7.97300400	0.98575000	-3.65931000
C	2.85215200	1.12155900	-2.01045700
H	2.58940800	2.14993500	-2.30027000
H	3.59893000	1.16846900	-1.21284500
H	3.34025900	0.65193800	-2.87951200
C	0.59695200	0.32395300	-2.79748200
H	-0.22388100	-0.38718200	-2.63079000
H	0.16606100	1.32664400	-2.95048500
H	1.11139800	0.03768900	-3.73015500
C	0.93063700	2.91712200	1.80622300
H	1.39286800	2.26732000	2.56241400
H	-0.15706300	2.92036900	1.97186100
C	0.71522300	3.50865000	-0.63396800
H	-0.34759900	3.71115700	-0.45073200
H	1.26514100	4.45898200	-0.52848000
H	0.81455600	3.18296600	-1.67847900
H	1.29587200	3.94411200	1.97470200
Br	-0.65787800	-2.40459000	-0.43098600
C	-3.45195500	-0.65680800	-0.55479800
C	-4.03802800	-0.79401300	0.56147600
C	-4.03627900	-0.83339800	-1.90592800
H	-4.16166600	-0.78327700	1.63319600

C	-5.44066000	-1.51418500	-1.74598100
H	-3.37037000	-1.43172700	-2.54615100
H	-4.12828300	0.16315200	-2.36610000
C	-6.05879500	-1.27609000	-0.38523200
H	-6.10606600	-1.13324200	-2.53551200
H	-5.32506800	-2.59020700	-1.91911100
C	-6.30602800	-2.34169100	0.54688800
C	-6.53133500	0.06887000	-0.10083400
O	-6.88389500	-2.17844300	1.66085600
C	-5.81301100	-3.74019300	0.23423900
O	-7.08055200	0.47234500	0.94731400
O	-6.28204000	0.93408100	-1.08643900
H	-6.01044300	-4.37978600	1.10361900
H	-6.33137900	-4.15861200	-0.64343500
H	-4.73386500	-3.74047500	0.01608400
C	-6.45310300	2.32698000	-0.81183900
H	-6.19475200	2.84817600	-1.74068200
H	-7.48992500	2.54650200	-0.51673200
H	-5.76503500	2.64472200	-0.01462000
Li	-7.49591200	-0.64293300	2.26595100

TS, M=Li, 6-exo oxocyclizaton

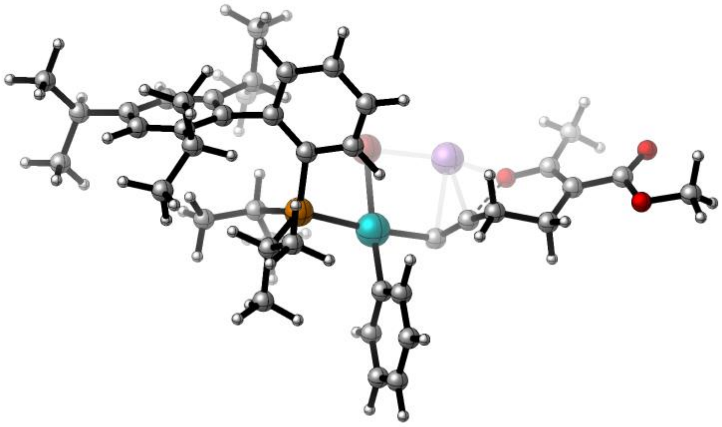

E (B3LYP-D3/Def2-SVP)) = -4909.98441009
E (SMD(DMF)/M06/Def2-TZVPP//B3LYP-D3/Def2-SVP) = -4910.80766578

Zero-point correction=	0.891651 (Hartree/Particle)
Thermal correction to Energy=	0.948377
Thermal correction to Enthalpy=	0.949321
Thermal correction to Gibbs Free Energy=	0.796769

Charge = 0 Multiplicity = 1

P	1.03274000	0.99479800	-0.22809900
C	1.78506400	2.55311600	0.50167100
H	2.85169200	2.29233400	0.57676500
C	2.13807600	0.55985600	-1.69975200
H	2.18966800	-0.53371900	-1.63152900
C	1.19395700	-0.23044700	1.17101500
C	2.32258400	-0.97836500	1.58487300
C	0.00487400	-0.34507700	1.92279600
C	2.20180100	-1.80698000	2.71803800
C	-0.08937200	-1.15671500	3.05028900

H	-0.87687600	0.21426100	1.60056100
C	1.02082700	-1.90118900	3.45092700
H	3.07390800	-2.38998900	3.02191100
H	-1.03022700	-1.21406300	3.60283500
H	0.96873700	-2.55506100	4.32499800
C	3.66534900	-0.97706900	0.91528500
C	4.69081900	-0.12755200	1.39764300
C	3.93328900	-1.89375400	-0.12877900
C	5.95746000	-0.19117900	0.80359000
C	5.21331100	-1.90559800	-0.69926900
C	6.23698200	-1.06320000	-0.25480000
H	6.74733200	0.46726900	1.17036600
H	5.42571900	-2.59779000	-1.51717300
C	-1.66690600	2.71914700	-0.67268000
C	-2.05849200	3.19704000	0.58746800
C	-1.65393200	3.61327400	-1.75593600
C	-2.41643400	4.54077300	0.76519800
H	-2.07545100	2.52814100	1.45238600
C	-2.00951000	4.95533900	-1.57702100
H	-1.35842600	3.26796500	-2.75006200
C	-2.38826900	5.42650900	-0.31512900
H	-2.71349300	4.89380600	1.75705800
H	-1.98991100	5.63710200	-2.43224400
H	-2.66349600	6.47512200	-0.17676300
C	4.45514400	0.82700000	2.56609100
H	3.36856300	0.95832900	2.67155400
C	7.60464100	-1.07666700	-0.92150100
H	7.58920900	-1.88245800	-1.67591700
C	2.87802800	-2.89415200	-0.59559500
H	1.88808300	-2.48095900	-0.35294200
C	4.96942400	0.21540300	3.88162000
H	6.05548200	0.03378500	3.82641100
H	4.77919300	0.89321600	4.73004800
H	4.47844900	-0.74470600	4.09694300
C	5.06491600	2.22115500	2.34919100
H	4.72580500	2.91184300	3.13764900
H	6.16559800	2.19884600	2.38861100
H	4.77735300	2.64740600	1.37572600
C	2.89287000	-3.15941800	-2.10830600
H	1.99554900	-3.72895700	-2.39386500
H	2.89012200	-2.22499300	-2.69036500
H	3.77370000	-3.74559500	-2.41697200
C	3.01511500	-4.21911600	0.17712100
H	2.92067800	-4.06565900	1.26180400
H	2.23187500	-4.92935700	-0.13383900
H	3.99680100	-4.68312600	-0.01552300
C	8.73263200	-1.39642500	0.07236400
H	9.70250400	-1.46550500	-0.44673400
H	8.82437000	-0.61172200	0.84118800
H	8.55029200	-2.35167000	0.58904900
C	7.87113600	0.24515000	-1.66224200
H	7.07812600	0.45643800	-2.39678300
H	7.90689100	1.09225600	-0.95738700
H	8.83524200	0.21108400	-2.19570000

C	3.56142300	1.13033700	-1.70028900
H	3.56297400	2.21516700	-1.88324100
H	4.11000800	0.93473000	-0.77466300
H	4.13165600	0.66018700	-2.51719800
C	1.43204400	0.90854600	-3.02250000
H	0.47232700	0.38426800	-3.12968700
H	1.24672700	1.99048600	-3.11693000
H	2.07643600	0.60721800	-3.86501100
C	1.27736000	2.88157800	1.91013800
H	1.40648200	2.04760700	2.61395300
H	0.21423600	3.15888800	1.89387900
C	1.63757200	3.76719000	-0.42636700
H	0.60785500	4.14727000	-0.42243300
H	2.30015000	4.57585700	-0.07567500
H	1.90723200	3.54805200	-1.46892000
H	1.84023700	3.74416300	2.30369000
Br	-0.74748600	-1.85463700	-1.47100600
Pd	-1.20825400	0.80226800	-0.97031000
C	-3.13787600	0.37523800	-1.75182100
C	-3.86934300	0.05580300	-0.75760700
H	-3.16852100	0.09161200	-2.80337400
C	-4.39043400	0.25442600	0.59533400
C	-5.93334200	0.27337600	0.62776400
H	-4.01239000	-0.53079300	1.27332400
H	-3.99857000	1.22473500	0.94028300
C	-6.57656200	-1.06577000	0.32868600
H	-6.28924700	1.05489700	-0.06868200
H	-6.23841200	0.60289000	1.62951500
C	-5.98642700	-1.95118700	-0.54946800
C	-7.85984300	-1.39419700	0.95129200
O	-4.86508700	-1.65011600	-1.17195200
C	-6.53628600	-3.31997200	-0.88270200
O	-8.31931800	-0.37578600	1.73961700
O	-8.50577100	-2.42062000	0.82755800
Li	-3.14738100	-2.05992900	-1.50138900
H	-6.68677300	-3.92270700	0.02387200
H	-7.53056900	-3.23748600	-1.34540700
H	-5.84996400	-3.83205400	-1.57278800
C	-9.56670400	-0.60506400	2.37953400
H	-9.51898100	-1.48035900	3.04756600
H	-9.78813100	0.30121300	2.95920900
H	-10.36547300	-0.78871800	1.64299800

PhBr

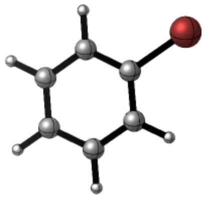

E (B3LYP-D3/Def2-SVP)) = -2805.36003379
E (SMD(DMF)/M06/Def2-TZVPP//B3LYP-D3/Def2-SVP) = -2805.61723417

Zero-point correction= 0.090520 (Hartree/Particle)

Thermal correction to Energy= 0.096204
Thermal correction to Enthalpy= 0.097148
Thermal correction to Gibbs Free Energy= 0.059647

Charge = 0 Multiplicity = 1

C	-2.18391600	-1.20936200	-0.00000200
C	-0.78556800	-1.21755000	-0.00002300
C	-0.09755200	-0.00006300	0.00003500
C	-0.78555000	1.21754000	0.00000500
C	-2.18381100	1.20942000	-0.00003200
C	-2.88602300	0.00001500	0.00002200
H	-2.72492600	-2.15887900	0.00000100
H	-0.23336600	-2.15871800	-0.00002900
H	-0.23317700	2.15860500	0.00000500
H	-2.72487100	2.15890900	-0.00002600
H	-3.97847900	0.00010200	0.00003500
Br	1.81226700	0.00000000	-0.00000100

Li-enolate of compound 1, complex with 2xDMF

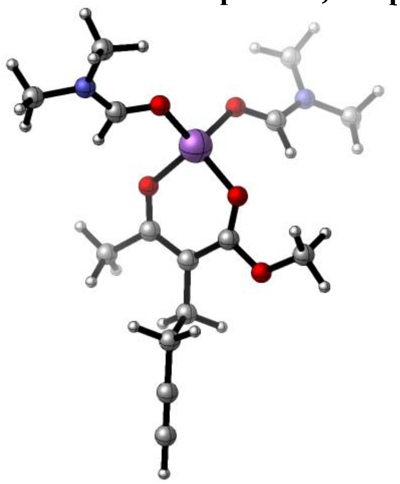

E (B3LYP-D3/Def2-SVP)) = -1079.14872864
E (SMD(DMF)/M06/Def2-TZVPP//B3LYP-D3/Def2-SVP) = -1079.62975743

Zero-point correction= 0.392129 (Hartree/Particle)
Thermal correction to Energy= 0.421718
Thermal correction to Enthalpy= 0.422662
Thermal correction to Gibbs Free Energy= 0.326226

Charge = 0 Multiplicity = 1

C	5.70984000	-0.05528800	-0.08638300
C	6.88908900	-0.29371400	0.06617100
C	4.28001900	0.20928600	-0.24113400
H	7.93386700	-0.50214000	0.19561200
C	3.37721900	-0.67016200	0.66278300
H	3.98577900	0.04025600	-1.29038600
H	4.08072900	1.27502100	-0.02992700
C	1.90070000	-0.37116400	0.50024300
H	3.58055500	-1.72276400	0.41497400
H	3.69595500	-0.53874200	1.70662400
C	1.21260000	0.51004300	1.37652700
C	1.21384900	-0.93944400	-0.61588200

O	-0.00992600	0.82154700	1.29262700
C	1.98778800	1.15631100	2.52442900
O	0.00961800	-0.84242200	-0.92487800
O	2.02730800	-1.68151000	-1.43248700
H	1.32385000	1.86704600	3.03440900
H	2.32037500	0.40075500	3.25545500
H	2.88936100	1.68488400	2.17542200
C	1.42358100	-2.28001500	-2.56467400
H	2.23447900	-2.76359100	-3.12684600
H	0.67567000	-3.03800500	-2.27386300
H	0.91683500	-1.53416600	-3.19799100
Li	-1.24855500	0.20522600	0.03047800
O	-2.64289400	-1.09901700	0.45069100
C	-2.47502600	-2.27118000	0.12607600
N	-3.32384000	-3.28145100	0.42429500
H	-1.58108300	-2.58243200	-0.45446400
C	-3.05434100	-4.64403200	0.01260500
C	-4.53636600	-3.03568800	1.18459100
H	-3.86236600	-5.02892200	-0.63423700
H	-2.10971200	-4.68723400	-0.54820900
H	-2.97036500	-5.31215600	0.88779700
H	-5.42609000	-3.33036800	0.60166800
H	-4.52952900	-3.61579500	2.12355900
H	-4.59336600	-1.96575300	1.41959900
O	-2.04071100	1.86472000	-0.63368100
C	-1.66571600	2.90745200	-0.10148300
N	-2.09323600	4.14283400	-0.44987800
H	-0.92557600	2.89040900	0.72596500
C	-1.60212200	5.32979100	0.22004300
C	-3.05875700	4.32438000	-1.51877300
H	-2.42958000	5.88835500	0.69216100
H	-0.88201100	5.04858000	1.00159600
H	-1.09740900	6.00581300	-0.49260600
H	-3.96659700	4.82590200	-1.14094500
H	-2.63202400	4.94404500	-2.32647400
H	-3.32785200	3.33964000	-1.92025900

TS1

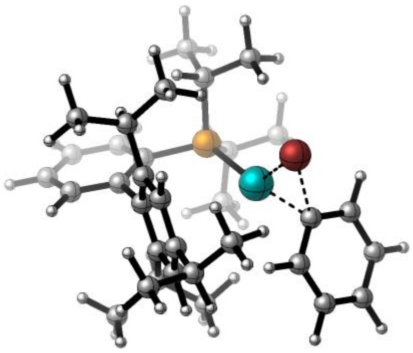

E (B3LYP-D3/Def2-SVP)) = -4327.54552756
E (SMD(DMF)/M06/Def2-TZVPP//B3LYP-D3/Def2-SVP) = -4328.11884401

Zero-point correction=	0.704607 (Hartree/Particle)
Thermal correction to Energy=	0.746047
Thermal correction to Enthalpy=	0.746991

Thermal correction to Gibbs Free Energy= 0.629977

Charge = 0 Multiplicity = 1

Pd	0.41662500	-1.22569300	-0.09915000
P	-1.93515400	-1.15140700	-0.21830800
C	-2.69012800	-2.56896400	0.76185000
H	-3.78133500	-2.55847300	0.60762600
C	-2.59741000	-1.30841400	-1.98933700
H	-2.50855300	-0.26765800	-2.34266900
C	-2.88413200	0.31912200	0.40523600
C	-2.24618400	1.57052400	0.58290500
C	-4.25790900	0.21714500	0.70066700
C	-3.01611700	2.67264600	1.00030000
C	-5.00327200	1.31631800	1.12747600
H	-4.76439100	-0.74427900	0.60784300
C	-4.37977500	2.55800300	1.26679100
H	-2.51520700	3.63550500	1.12629300
H	-6.06726500	1.20058000	1.34878600
H	-4.94963400	3.43159300	1.59340300
C	-0.77539800	1.81857100	0.40618100
C	-0.26423400	2.25860700	-0.83495600
C	0.07625300	1.72835500	1.53201000
C	1.08296800	2.62692100	-0.92230000
C	1.41210600	2.12692800	1.39760000
C	1.93834600	2.57933600	0.18331500
H	1.47511700	2.96191400	-1.88431100
H	2.07211800	2.07148000	2.26744100
C	2.36455800	-1.74084700	0.11875500
C	3.08908200	-0.91375500	0.99786700
C	2.35739700	-3.14187200	0.27859600
C	3.72318100	-1.49238700	2.09767900
H	3.12097700	0.16055700	0.82826900
C	2.99922700	-3.69708500	1.39309500
H	1.87472900	-3.77848300	-0.46522000
C	3.67780000	-2.87908400	2.30187900
H	4.25714000	-0.85157800	2.80479700
H	2.97860200	-4.78071400	1.53813000
H	4.18557900	-3.32151000	3.16229900
C	-1.14987600	2.35088500	-2.07224000
H	-2.10835600	1.86663100	-1.83052600
C	3.39048000	3.02681100	0.08608900
H	3.89667100	2.67671000	1.00382400
C	-0.43393900	1.23349900	2.88285900
H	-1.42600900	0.78754400	2.71965000
C	-1.46621200	3.81477700	-2.42023800
H	-0.54389100	4.37146300	-2.65450000
H	-2.13128000	3.87615600	-3.29759900
H	-1.96121000	4.32459300	-1.57865100
C	-0.54823100	1.60188400	-3.27202300
H	-1.27129300	1.55888800	-4.10310900
H	0.35813800	2.10121900	-3.64998500
H	-0.26310800	0.57576500	-2.99773900
C	0.45255300	0.13238200	3.48436300
H	-0.00657600	-0.27054900	4.40169200

H	0.59425400	-0.69709300	2.77452900
H	1.45323300	0.50676500	3.75338700
C	-0.61624500	2.40405400	3.86364500
H	-1.31117300	3.15641200	3.45941600
H	-1.01725000	2.05109600	4.82819300
H	0.34579700	2.90604500	4.05916700
C	3.48448800	4.56297000	0.06706900
H	4.53574000	4.89510400	0.04735900
H	2.98367800	4.97006100	-0.82707500
H	2.99896800	5.00425400	0.95166500
C	4.13037100	2.41262100	-1.11217100
H	4.04152000	1.31626600	-1.12787900
H	3.72726000	2.78587000	-2.06753800
H	5.19949300	2.67847900	-1.08189500
C	-4.06246100	-1.73587800	-2.12381900
H	-4.22447500	-2.76052000	-1.75204700
H	-4.74910300	-1.06224500	-1.59251800
H	-4.35629100	-1.72821500	-3.18694300
C	-1.67840300	-2.16995900	-2.86766900
H	-0.62643400	-1.85778100	-2.79644300
H	-1.72922900	-3.23488000	-2.59296600
H	-1.98982800	-2.08755700	-3.92240300
C	-2.39570800	-2.36316400	2.25434400
H	-2.81235400	-1.41784000	2.63389900
H	-1.30925200	-2.35143700	2.44000400
C	-2.14741000	-3.91940500	0.27660900
H	-1.04687900	-3.94850300	0.33984300
H	-2.54459500	-4.73286100	0.90644100
H	-2.43184400	-4.13662300	-0.76305200
H	-2.83108000	-3.18594100	2.84550800
Br	2.21848100	-0.99578600	-1.99467000

IM2

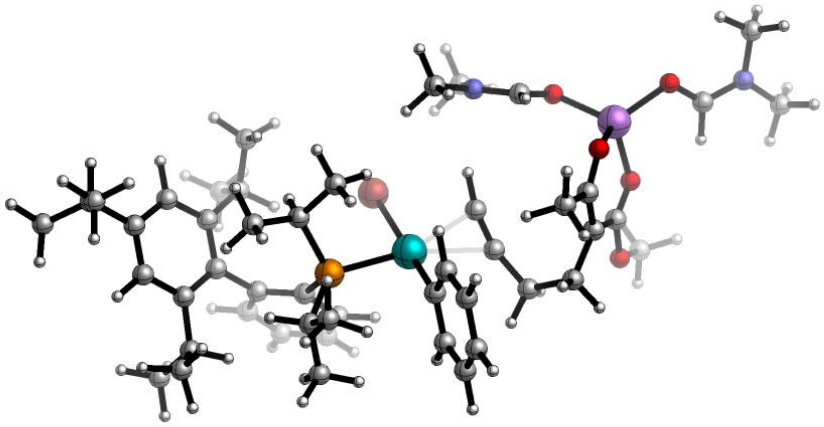

E (B3LYP-D3/Def2-SVP)) = -5406.75930458
E (SMD(DMF)/M06/Def2-TZVPP//B3LYP-D3/Def2-SVP) = -5407.79235405

Zero-point correction=	1.101322 (Hartree/Particle)
Thermal correction to Energy=	1.173464
Thermal correction to Enthalpy=	1.174408
Thermal correction to Gibbs Free Energy=	0.986687

Charge = 0 Multiplicity = 1

Pd	-0.13064600	0.71750200	0.12942000
P	2.19912900	0.84959000	-0.15797300
C	3.14605300	2.39105300	-0.67200200
H	4.15784400	1.98035000	-0.81024100
C	2.64842100	-0.44016500	-1.46851500
H	2.73787000	-1.34802400	-0.86006300
C	2.95243400	0.49163200	1.51064300
C	4.19933500	-0.10322600	1.81916400
C	2.15643100	0.96755100	2.57362300
C	4.58159300	-0.19831900	3.17205000
C	2.56030100	0.87932700	3.90290700
H	1.18113200	1.40539000	2.34799200
C	3.78598100	0.28603500	4.20757200
H	5.53777100	-0.67340300	3.40201100
H	1.90971400	1.25830100	4.69443400
H	4.11888400	0.19264300	5.24430600
C	5.17195300	-0.66692300	0.82516700
C	6.26781100	0.11694400	0.38775200
C	5.04865900	-2.01313600	0.40552500
C	7.19870000	-0.45106000	-0.49130800
C	6.00042700	-2.52820800	-0.48490200
C	7.07677800	-1.76716400	-0.95111400
H	8.03934600	0.15493100	-0.83521300
H	5.90443500	-3.56087600	-0.82789800
C	-0.39983000	2.51826900	-0.72871400
C	-0.31321500	3.72758300	-0.02108200
C	-0.74779900	2.56618000	-2.08817100
C	-0.56568400	4.95175700	-0.65408900
H	-0.04819300	3.72470500	1.03974500
C	-1.00197000	3.78798400	-2.72291700
H	-0.82457000	1.64256600	-2.66764300
C	-0.90873100	4.98698400	-2.00924200
H	-0.49170200	5.88162200	-0.08271900
H	-1.27790100	3.79972900	-3.78115400
H	-1.10585000	5.94129400	-2.50415700
C	6.47594000	1.54880700	0.87646200
H	5.52120600	1.89808100	1.29484000
C	8.06815100	-2.34574300	-1.94999900
H	7.79379500	-3.40415200	-2.10246800
C	3.94171700	-2.91310100	0.94859700
H	3.10170300	-2.27347200	1.25580800
C	7.51270500	1.59037900	2.01334600
H	8.49081200	1.22376800	1.66044900
H	7.64739200	2.61991800	2.38412600
H	7.20385500	0.96152200	2.86089600
C	6.87008700	2.52581400	-0.24304800
H	6.85035600	3.56184900	0.13121100
H	7.88907500	2.33444400	-0.61545300
H	6.18605900	2.46134200	-1.10321200
C	3.38401400	-3.90956900	-0.07826900
H	2.46492200	-4.36903100	0.31564700
H	3.12523900	-3.41790600	-1.02876300
H	4.09734200	-4.72004500	-0.30122700

C	4.42544400	-3.66000400	2.20525500
H	4.74897400	-2.96108700	2.99005300
H	3.61434900	-4.28058000	2.61940500
H	5.27655900	-4.31933200	1.96536600
C	9.51145600	-2.31446300	-1.42255000
H	10.19877700	-2.79802900	-2.13585500
H	9.86228100	-1.27983400	-1.27511900
H	9.59454400	-2.83686600	-0.45668500
C	7.95621600	-1.63843800	-3.31167700
H	6.92793100	-1.68954400	-3.70294900
H	8.22749900	-0.57317900	-3.22613600
H	8.63065700	-2.09860200	-4.05249000
C	3.94452500	-0.21992100	-2.25825200
H	3.84621200	0.60658600	-2.97731000
H	4.81607500	-0.02659400	-1.62747900
H	4.16355400	-1.13196400	-2.83598200
C	1.47801200	-0.65479700	-2.44397500
H	0.55963400	-0.95358700	-1.92132300
H	1.26341200	0.24956700	-3.03486500
H	1.74378900	-1.45834800	-3.15089900
C	3.22480200	3.46862600	0.41530900
H	3.61363300	3.08474800	1.36833700
H	2.24028100	3.91896300	0.60096600
C	2.68248200	2.99769200	-2.00442200
H	1.77918500	3.60601400	-1.87372400
H	3.47767700	3.65318300	-2.39732800
H	2.46496400	2.24380000	-2.77269200
H	3.89882500	4.27250500	0.07560500
Br	0.29296200	-1.73679000	0.98334800
C	-2.47457400	1.08235800	0.69078200
C	-2.26310800	-0.00665600	0.15885000
C	-3.10992700	2.28476800	1.22880600
H	-2.33683000	-1.01939700	-0.19089700
C	-4.27661400	2.77150800	0.31551200
H	-2.37239900	3.08636800	1.37920500
H	-3.51777300	2.01934800	2.21788700
C	-5.21613900	1.65706100	-0.09646400
H	-4.80719500	3.55465700	0.88074900
H	-3.83396900	3.26035100	-0.56165100
C	-5.20569100	1.10879300	-1.41163800
C	-6.04141900	1.06882000	0.90844600
O	-5.83941600	0.08004800	-1.77674400
C	-4.35911300	1.78280600	-2.48836600
O	-6.78086300	0.06965000	0.82835600
O	-5.97582000	1.72441000	2.11433500
H	-4.55790400	1.28019700	-3.44433000
H	-4.58538000	2.85607400	-2.59018800
H	-3.28719900	1.70434200	-2.25275300
C	-6.74733400	1.18641800	3.17323700
H	-6.50943500	1.78478000	4.06391100
H	-7.82830000	1.25533400	2.96278500
H	-6.50512600	0.12680200	3.35483700
Li	-6.66320100	-1.14966200	-0.63433700
O	-8.48977200	-1.80157900	-0.77237900

C	-9.33583000	-1.32039800	-0.02305300
N	-10.64763900	-1.65065500	-0.03419800
H	-9.06153000	-0.55758400	0.73549200
C	-11.59054800	-1.03784300	0.87882700
C	-11.16093600	-2.63294200	-0.97246100
H	-12.07315800	-1.79796600	1.51826300
H	-11.07144600	-0.31728300	1.52687800
H	-12.38372600	-0.50242900	0.32772200
H	-11.61840300	-3.48115900	-0.43439300
H	-11.92842900	-2.18188300	-1.62496500
H	-10.33025700	-2.99871200	-1.58842900
O	-5.28841200	-2.54869700	-0.51470700
C	-4.29244400	-2.47865500	-1.23218600
N	-3.12344300	-3.11689200	-0.99739700
H	-4.28037900	-1.83029600	-2.13396900
C	-2.01340900	-3.05339000	-1.93513200
C	-2.95126300	-3.96349800	0.17546100
H	-1.85992600	-4.02855900	-2.43086300
H	-2.21690100	-2.29646100	-2.70666000
H	-1.08699700	-2.78265500	-1.40198500
H	-2.82051500	-5.01764000	-0.12564500
H	-2.06036600	-3.63840100	0.73690700
H	-3.84438200	-3.87649400	0.80583400

IM3

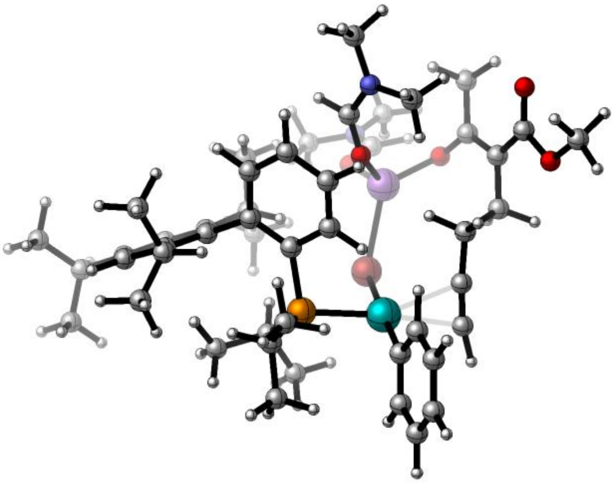

E (B3LYP-D3/Def2-SVP)) = -5406.75807377
E (SMD(DMF)/M06/Def2-TZVPP//B3LYP-D3/Def2-SVP) = -5407.78896176

Zero-point correction=	1.101479 (Hartree/Particle)
Thermal correction to Energy=	1.173592
Thermal correction to Enthalpy=	1.174536
Thermal correction to Gibbs Free Energy=	0.989652

Charge = 0 Multiplicity = 1

P	-1.56706300	1.72047900	0.47704300
C	-2.74845700	2.99737700	-0.24489100
H	-3.62362000	2.37589200	-0.49105800
C	-2.59043900	0.83985100	1.80004500
H	-2.19041400	-0.18176700	1.75872000

C	-1.18890600	0.69427900	-1.04024900
C	-1.92952800	-0.35667100	-1.62995700
C	-0.05018900	1.16921200	-1.72523200
C	-1.49131300	-0.87958800	-2.86404000
C	0.33753400	0.67296100	-2.96503800
H	0.54259000	1.96483400	-1.27291300
C	-0.38841000	-0.36771000	-3.54279700
H	-2.06065800	-1.70397700	-3.29890400
H	1.21433500	1.08971200	-3.46293700
H	-0.09349600	-0.78673400	-4.50823100
C	-3.17718300	-0.97143400	-1.07170500
C	-4.43687700	-0.56774200	-1.57870100
C	-3.09152000	-2.01333500	-0.11876000
C	-5.59104400	-1.20794500	-1.11076300
C	-4.27959400	-2.60438900	0.33274900
C	-5.53605400	-2.22006300	-0.14609100
H	-6.56321800	-0.89487500	-1.49721800
H	-4.22763000	-3.39786600	1.08130300
C	0.46224300	4.15905900	0.86640900
C	1.02042700	4.65362600	-0.32258300
C	-0.04863800	5.07976700	1.79624400
C	1.04689000	6.02917000	-0.58646100
H	1.45226000	3.97016500	-1.05897000
C	-0.01593500	6.45626700	1.53693200
H	-0.48371100	4.72763700	2.73608100
C	0.52473800	6.93636600	0.34028900
H	1.48357000	6.39131200	-1.52176300
H	-0.41967700	7.15508400	2.27548800
H	0.54652400	8.00971400	0.13513200
C	-4.56831000	0.54526100	-2.61722200
H	-3.61406700	1.09235100	-2.64087600
C	-6.80638400	-2.86389200	0.38982600
H	-6.49591000	-3.66246200	1.08598000
C	-1.74101700	-2.53385100	0.36424300
H	-1.01109500	-1.71469600	0.29113700
C	-4.79814900	-0.03392900	-4.02398100
H	-5.73206700	-0.61910100	-4.05558700
H	-4.87553500	0.77187500	-4.77245500
H	-3.97507500	-0.69864900	-4.32415000
C	-5.66732600	1.56375600	-2.27040900
H	-5.61388500	2.42941400	-2.94999800
H	-6.67516800	1.13087000	-2.37258400
H	-5.57054700	1.93365700	-1.23810500
C	-1.73553100	-3.00282500	1.82545300
H	-0.69835000	-3.17213400	2.14453300
H	-2.17435100	-2.25041700	2.49840800
H	-2.29545200	-3.94423700	1.95775700
C	-1.23834000	-3.66096900	-0.55762300
H	-1.16910000	-3.32212900	-1.60198700
H	-0.24173700	-3.99195100	-0.22885300
H	-1.92638600	-4.52312500	-0.52985000
C	-7.63902400	-3.51975900	-0.72331300
H	-8.51834300	-4.03424700	-0.30239100
H	-8.00751300	-2.77030000	-1.44295400

H	-7.04430500	-4.25779100	-1.28405100
C	-7.64420100	-1.85244600	1.19094400
H	-7.05531600	-1.40753300	2.00841100
H	-7.99281100	-1.02957800	0.54519100
H	-8.53312300	-2.33524100	1.62928000
C	-4.11125100	0.80444500	1.60420600
H	-4.56820700	1.79058600	1.77446100
H	-4.41773400	0.44788700	0.61808800
H	-4.54418500	0.11312300	2.34463400
C	-2.26180500	1.41167100	3.19242300
H	-1.19600200	1.32587500	3.43824400
H	-2.56047600	2.46776900	3.28565300
H	-2.82533200	0.84277300	3.95016200
C	-2.25927900	3.65414800	-1.54080000
H	-2.04581900	2.92253600	-2.33204800
H	-1.35367100	4.25358400	-1.37008400
C	-3.17854000	4.06474300	0.77004100
H	-2.36225100	4.77210400	0.96438700
H	-4.02824000	4.63429900	0.35830300
H	-3.50063700	3.64387100	1.73151700
H	-3.04451100	4.33174600	-1.91528000
Br	0.76097700	-0.27833600	2.16786000
Pd	0.56340600	2.20900900	1.34847600
C	2.55343200	2.82563700	2.34927700
C	2.94468200	2.17167400	1.38445100
H	2.42471200	3.44358100	3.22035000
C	3.56306600	1.35318000	0.34129300
C	4.79678500	0.56286900	0.85990200
H	2.81462600	0.64158900	-0.03997700
H	3.86717100	1.99818200	-0.50029100
C	5.31762100	-0.41967000	-0.16141700
H	4.49110900	0.01198700	1.76005300
H	5.57444100	1.28658100	1.14420700
C	4.88327500	-1.75428800	-0.05414100
C	6.13219200	0.06321000	-1.24987400
O	4.00123700	-2.10994200	0.79774400
C	5.42554400	-2.87519200	-0.92661100
O	6.38691500	1.41657900	-1.13170200
O	6.55679300	-0.53535000	-2.23683000
Li	2.15157000	-1.90855900	0.80135600
H	4.64670700	-3.16861000	-1.65261500
H	6.32953000	-2.60929900	-1.48273500
H	5.60093400	-3.74969900	-0.27954200
C	7.16239000	1.99128600	-2.16753000
H	6.67522000	1.87849200	-3.15093600
H	7.26787300	3.05759000	-1.92214700
H	8.15759800	1.52202100	-2.23735900
O	1.60447400	-3.70466400	1.38160600
C	2.49378800	-4.44867000	1.79963800
N	2.27723800	-5.66704200	2.33891600
H	3.55612600	-4.13418500	1.74337200
C	3.37562600	-6.48718400	2.81134700
C	0.93335200	-6.20036500	2.48060900
H	3.40567500	-7.45213200	2.27548700

H	4.32955200	-5.96664100	2.64693900
H	3.27374200	-6.69843200	3.89018300
H	0.82891600	-7.14244700	1.91499300
H	0.71072500	-6.40747000	3.54140100
H	0.21288100	-5.46685000	2.09923900
O	1.71921000	-1.57793200	-1.04024800
C	1.81887400	-2.27735900	-2.04412600
N	2.58883500	-2.00547100	-3.11480700
H	1.25161400	-3.22819000	-2.14269500
C	2.64960600	-2.90452100	-4.24934200
C	3.43694400	-0.82298400	-3.16628600
H	3.68039100	-3.26716900	-4.40115900
H	1.99236600	-3.77056300	-4.08446700
H	2.32784200	-2.39129900	-5.17251900
H	4.50275100	-1.09920200	-3.19565300
H	3.19442200	-0.22899500	-4.06337300
H	3.26768700	-0.22301800	-2.26670200

TS2

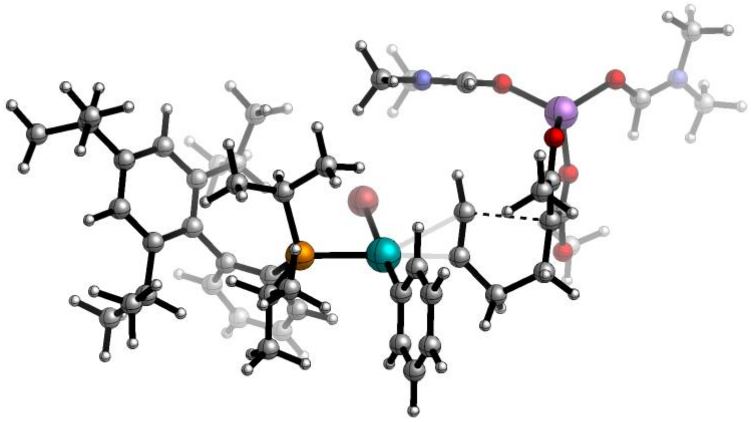

E (B3LYP-D3/Def2-SVP)) = -5406.74741826
E (SMD(DMF)/M06/Def2-TZVPP//B3LYP-D3/Def2-SVP) = -5407.77679160

Zero-point correction=	1.100654 (Hartree/Particle)
Thermal correction to Energy=	1.172077
Thermal correction to Enthalpy=	1.173021
Thermal correction to Gibbs Free Energy=	0.986988

Charge = 0 Multiplicity = 1

Pd	-0.21569500	1.37071400	0.26615900
P	2.11001700	1.08655800	-0.09604600
C	3.28311500	2.44822300	-0.65390400
H	4.21865200	1.89507200	-0.82900900
C	2.28554000	-0.25700700	-1.41737000
H	2.21934900	-1.17035200	-0.81361100
C	2.86126700	0.59609100	1.54054400
C	3.96264900	-0.24810600	1.81570700
C	2.22718700	1.24054000	2.62387000
C	4.36926400	-0.41066200	3.15533200
C	2.65593900	1.08290500	3.93868200
H	1.35739000	1.87119100	2.42505500
C	3.73725300	0.24343300	4.21003700

H	5.21134000	-1.07615500	3.35835500
H	2.13300300	1.60052600	4.74625200
H	4.08306800	0.09140900	5.23562900
C	4.75909900	-1.01007200	0.79803000
C	5.97538700	-0.47092700	0.31247200
C	4.34694000	-2.30456800	0.40128000
C	6.74410700	-1.22837700	-0.58032400
C	5.14416500	-3.01330400	-0.50779200
C	6.34105700	-2.49646500	-1.01351900
H	7.67893900	-0.81062600	-0.95964600
H	4.82718600	-4.00693500	-0.83290700
C	-0.26346000	3.16887600	-0.63061200
C	0.02440200	4.36258100	0.05291000
C	-0.65466900	3.25802500	-1.97796400
C	-0.05294700	5.60280800	-0.59448300
H	0.30973300	4.33270500	1.10811500
C	-0.73400800	4.49611000	-2.62754600
H	-0.89710800	2.34985200	-2.53639300
C	-0.42704600	5.67508700	-1.94026600
H	0.18144700	6.51750000	-0.04165700
H	-1.03692800	4.53751000	-3.67811000
H	-0.48498400	6.64200500	-2.44686900
C	6.47466900	0.90307900	0.75465300
H	5.61915400	1.44178500	1.18755200
C	7.16296500	-3.27865900	-2.02752800
H	6.67350000	-4.26029900	-2.15307500
C	3.09251400	-2.94837400	0.98690700
H	2.41159100	-2.14533400	1.30458800
C	7.53624000	0.76992600	1.86082500
H	8.41178500	0.20936100	1.49335900
H	7.88178400	1.76236100	2.19447200
H	7.13793800	0.23670000	2.73616900
C	7.01235100	1.75659700	-0.40543100
H	7.20230600	2.78686800	-0.06419900
H	7.96363000	1.36363500	-0.79852400
H	6.30070500	1.80135500	-1.24422600
C	2.31089000	-3.81702800	-0.00970200
H	1.33236000	-4.08039400	0.42001400
H	2.12567000	-3.28753700	-0.95720700
H	2.83683900	-4.75650000	-0.24673800
C	3.44310100	-3.76696900	2.24282000
H	3.92742500	-3.14219800	3.00693900
H	2.53127600	-4.19716000	2.68807700
H	4.12976500	-4.59354900	1.99354200
C	8.59712200	-3.53734700	-1.53908200
H	9.14973200	-4.16078600	-2.26097300
H	9.15621500	-2.59472100	-1.42033400
H	8.60007300	-4.05285200	-0.56595600
C	7.15820200	-2.58102400	-3.39875900
H	6.13013600	-2.42465000	-3.76163500
H	7.64413900	-1.59306700	-3.33996500
H	7.70198400	-3.17910000	-4.14855100
C	3.57633100	-0.28200200	-2.24409300
H	3.62049300	0.55966100	-2.95105700

H	4.48472500	-0.26896900	-1.63638300
H	3.60098100	-1.20953000	-2.83870300
C	1.06821200	-0.23531900	-2.35840000
H	0.12362800	-0.32122900	-1.80526000
H	1.02885700	0.68871600	-2.95621600
H	1.13776400	-1.08311800	-3.06072600
C	3.55428200	3.50134200	0.42684800
H	3.95003000	3.06525700	1.35456400
H	2.64147600	4.06297800	0.67177700
C	2.86052800	3.12071800	-1.96847400
H	2.03605700	3.82676200	-1.80927400
H	3.71586800	3.68473100	-2.37706700
H	2.53925800	2.40502100	-2.73714000
H	4.29737100	4.22386100	0.04984600
Br	-0.21471200	-1.05923000	1.25288500
C	-2.31768700	1.75831900	0.51896700
C	-2.70770600	0.71074800	-0.06568500
C	-3.10029400	2.93562100	0.96698200
H	-2.74860600	-0.30469200	-0.41930200
C	-4.43469300	2.97487500	0.14333700
H	-2.53947500	3.87464200	0.86081400
H	-3.32136400	2.79780400	2.03794600
C	-4.95169800	1.60741300	-0.24082500
H	-5.18223000	3.51768100	0.74217100
H	-4.25089600	3.57491900	-0.75591100
C	-4.97533900	1.14425200	-1.60483900
C	-5.54858400	0.79637400	0.79521400
O	-5.38135700	0.01298800	-1.96236400
C	-4.44071800	2.05746100	-2.70109100
O	-6.05072400	-0.33180600	0.69500300
O	-5.51377300	1.39382900	2.01683000
H	-4.47740400	1.50875500	-3.65115900
H	-5.04942000	2.97179400	-2.79215300
H	-3.40615500	2.37259400	-2.49634700
C	-5.98972500	0.63200200	3.11605900
H	-5.80463900	1.23884900	4.01241600
H	-7.06912400	0.42504700	3.02439700
H	-5.45965800	-0.33017300	3.19401900
Li	-6.01900400	-1.39643400	-0.89899200
O	-7.81248200	-2.13292300	-0.92578400
C	-8.57114000	-1.92620900	0.01789800
N	-9.82869500	-2.41264000	0.11370800
H	-8.25698500	-1.30204100	0.88089600
C	-10.66851100	-2.12408700	1.25863100
C	-10.38701600	-3.24644000	-0.93671600
H	-10.96499200	-3.05383500	1.77517100
H	-10.12566400	-1.48813200	1.97246500
H	-11.58823500	-1.59637500	0.95072900
H	-10.66579100	-4.23679200	-0.53749500
H	-11.29013300	-2.77766300	-1.36395900
H	-9.63468800	-3.37102100	-1.72511200
O	-4.63328600	-2.72295500	-0.88750600
C	-3.55068100	-2.61946700	-1.46672800
N	-2.40929300	-3.21771900	-1.08506300

H	-3.44027300	-1.97925100	-2.36924900
C	-1.16386800	-3.03564600	-1.80970800
C	-2.35540000	-4.03106900	0.12089900
H	-0.79654500	-4.00124800	-2.19899900
H	-1.31711700	-2.35039200	-2.65512300
H	-0.40473300	-2.60436500	-1.13936900
H	-1.98652400	-5.04221700	-0.12132400
H	-1.67657600	-3.56023900	0.84993100
H	-3.36334500	-4.10048300	0.54674300

TS3

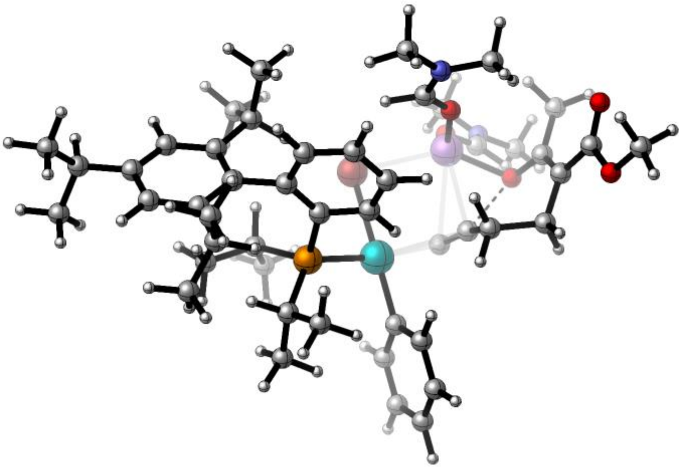

E (B3LYP-D3/Def2-SVP)) = -5406.73880962
E (SMD(DMF)/M06/Def2-TZVPP//B3LYP-D3/Def2-SVP) = -5407.77060600

Zero-point correction=	1.099699 (Hartree/Particle)
Thermal correction to Energy=	1.171534
Thermal correction to Enthalpy=	1.172478
Thermal correction to Gibbs Free Energy=	0.985702

Charge = 0 Multiplicity = 1

P	-1.73667000	1.42111500	0.09177400
C	-2.81585800	2.63011200	-0.86480000
H	-3.81383100	2.16995500	-0.80227700
C	-2.69991500	1.10098100	1.68813500
H	-2.49529700	0.03950200	1.87611000
C	-1.69531300	-0.04840000	-1.07072700
C	-2.67425200	-1.04248000	-1.30693500
C	-0.53152700	-0.06195400	-1.87042300
C	-2.45512300	-1.97491600	-2.34159800
C	-0.33300900	-0.98838300	-2.89093500
H	0.24428300	0.68116700	-1.67589800
C	-1.31328500	-1.95056500	-3.13873700
H	-3.21681700	-2.73906100	-2.51163000
H	0.58485800	-0.95466500	-3.48298600
H	-1.18902800	-2.68364500	-3.93950100
C	-3.94184200	-1.22202700	-0.52675100
C	-5.16542000	-0.73059700	-1.04130300
C	-3.92203600	-1.97094500	0.67442500
C	-6.34676000	-0.98128000	-0.33181700
C	-5.12965600	-2.17696700	1.35441500

C	-6.35018300	-1.69125800	0.87377200
H	-7.29058500	-0.59707700	-0.72419200
H	-5.12492700	-2.73729300	2.29178200
C	0.63101200	3.61766300	-0.14833100
C	0.81886100	3.89868800	-1.51039400
C	0.54575500	4.70145100	0.74462700
C	0.91100800	5.21904100	-1.97191600
H	0.88211000	3.08552300	-2.23858000
C	0.63612000	6.02097400	0.28599400
H	0.39947800	4.52089300	1.81344700
C	0.81554400	6.28690300	-1.07602500
H	1.05347900	5.40943500	-3.03984800
H	0.56428300	6.84705800	0.99980700
H	0.88355400	7.31734500	-1.43436600
C	-5.22818600	0.05274300	-2.35064900
H	-4.20589300	0.38064800	-2.58897000
C	-7.63708900	-1.90175000	1.65792900
H	-7.38727000	-2.53612000	2.52619200
C	-2.62642900	-2.58592700	1.19951100
H	-1.79180000	-1.95289800	0.86559200
C	-5.70243800	-0.84463500	-3.50731600
H	-6.71776900	-1.22721000	-3.31136100
H	-5.72723000	-0.28234200	-4.45529200
H	-5.03822300	-1.71057900	-3.64340000
C	-6.10146300	1.31480100	-2.25864200
H	-5.98397400	1.92683800	-3.16720300
H	-7.17127700	1.06781800	-2.16820800
H	-5.83073500	1.93638600	-1.39153600
C	-2.53734200	-2.64964200	2.73008600
H	-1.50896100	-2.90092800	3.02956600
H	-2.78485400	-1.68233500	3.19320100
H	-3.21123000	-3.41225300	3.15408000
C	-2.40197100	-3.98454400	0.59468700
H	-2.38188100	-3.95160100	-0.50460400
H	-1.44307400	-4.40353600	0.94263800
H	-3.20578300	-4.67679300	0.89578000
C	-8.70425700	-2.64269000	0.83670300
H	-9.60020000	-2.83989800	1.44774800
H	-9.02323600	-2.04864500	-0.03543700
H	-8.32318600	-3.60637900	0.46401800
C	-8.17712100	-0.56723900	2.20052900
H	-7.42118700	-0.05261700	2.81425100
H	-8.45436100	0.11039600	1.37613400
H	-9.07459400	-0.72730400	2.82043200
C	-4.21556300	1.33073300	1.65719300
H	-4.46312100	2.40074200	1.58747100
H	-4.72091400	0.80581900	0.84201200
H	-4.64982100	0.95760600	2.59866800
C	-2.07229300	1.90300300	2.84331900
H	-1.01479400	1.64883300	2.99325100
H	-2.15044200	2.98968000	2.67976500
H	-2.61137800	1.66897700	3.77642400
C	-2.45184200	2.76112800	-2.34805200
H	-2.43944500	1.79460600	-2.87086900

H	-1.46970800	3.23610600	-2.47575300
C	-2.86581200	4.01060400	-0.19524600
H	-1.91005100	4.53999700	-0.30601600
H	-3.65079000	4.62037100	-0.67303300
H	-3.09680700	3.95796900	0.87775300
H	-3.19846400	3.40233700	-2.84571300
Br	0.51086000	-0.56638100	1.86110500
Pd	0.54163000	1.76489900	0.60827200
C	2.64778000	1.75873500	1.01268700
C	3.10427100	1.29303200	-0.06855100
H	2.97141300	1.85027600	2.04450700
C	3.17821400	1.04100600	-1.50606500
C	4.61818400	1.02840500	-2.08073100
H	2.68712700	0.08085400	-1.73880400
H	2.59649900	1.84553600	-1.98393200
C	5.35839100	-0.25278400	-1.77601100
H	5.15088900	1.90782900	-1.67814000
H	4.54492600	1.16741900	-3.16535100
C	5.43769600	-0.62973800	-0.43803600
C	5.86356000	-1.08927400	-2.84814300
O	4.82958900	0.06260500	0.47386400
C	6.19585300	-1.83598600	0.07367600
O	5.82401900	-0.46275200	-4.07087800
O	6.26704800	-2.24513100	-2.77754800
Li	3.26930600	-0.86944500	1.36369400
H	5.48295700	-2.66265000	0.22645900
H	6.96973200	-2.18332400	-0.61785000
H	6.63059300	-1.58649700	1.05411600
C	6.26318000	-1.23608400	-5.17420300
H	5.65652800	-2.14933600	-5.29626400
H	6.16165300	-0.59657900	-6.06213000
H	7.31276000	-1.55071300	-5.05457300
O	4.00849000	-1.04846900	3.12483500
C	5.08863400	-0.53546000	3.41907300
N	5.68166000	-0.64445500	4.62611100
H	5.65029700	0.06553600	2.67498600
C	6.96175300	-0.02057300	4.89817400
C	5.06169200	-1.40418900	5.69838300
H	7.71718200	-0.77697200	5.17388100
H	7.31559300	0.51538200	4.00608700
H	6.87953800	0.69999600	5.73052300
H	5.71103000	-2.24264800	6.00388400
H	4.89015000	-0.75981700	6.57746900
H	4.10139200	-1.79767900	5.34322100
O	2.94479900	-2.19677600	0.05360500
C	1.99476400	-2.79749100	-0.44785200
N	2.08291100	-3.57024500	-1.54659800
H	0.97534400	-2.72080100	-0.01678400
C	0.94297700	-4.32797800	-2.02336000
C	3.34467600	-3.73045900	-2.25741900
H	1.12898200	-5.41309100	-1.93624400
H	0.04685200	-4.07269100	-1.44124400
H	0.74169100	-4.09871200	-3.08231600
H	3.70607400	-4.76998800	-2.17981300

H	3.20686100	-3.49206100	-3.32480800
H	4.10465600	-3.05817400	-1.84495800

IM4

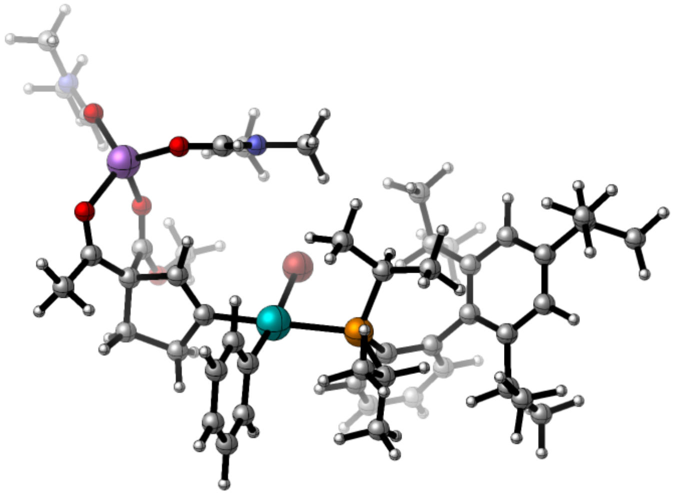

E (B3LYP-D3/Def2-SVP)) = -5406.78821095
E (SMD(DMF)/M06/Def2-TZVPP//B3LYP-D3/Def2-SVP) = -5407.81891179

Zero-point correction=	1.103743 (Hartree/Particle)
Thermal correction to Energy=	1.174778
Thermal correction to Enthalpy=	1.175723
Thermal correction to Gibbs Free Energy=	0.990390

Charge = 0 Multiplicity = 1

Pd	-0.30869900	1.57837800	0.12705600
P	2.04532800	1.10851000	-0.21167600
C	3.29838300	2.40241900	-0.76459100
H	4.22655400	1.83627600	-0.93691800
C	2.18574900	-0.26608800	-1.50456200
H	2.06043100	-1.17122400	-0.89690600
C	2.73962500	0.59620600	1.44687400
C	3.78521700	-0.29971500	1.77115400
C	2.11194600	1.29186700	2.50280100
C	4.15398300	-0.45269400	3.12281000
C	2.50068900	1.14159700	3.83143700
H	1.27993200	1.95983200	2.26581800
C	3.53299200	0.25745200	4.14779600
H	4.95551400	-1.15603400	3.36052100
H	1.98576900	1.70306300	4.61486100
H	3.84857300	0.11331500	5.18440700
C	4.55366800	-1.13337800	0.78966200
C	5.80900100	-0.68295800	0.31458800
C	4.06781200	-2.40849400	0.41486800
C	6.54783300	-1.51083800	-0.54019900
C	4.83772400	-3.19015800	-0.45728600
C	6.07566500	-2.76313600	-0.94803300
H	7.51322900	-1.16098900	-0.91167100
H	4.46576700	-4.16974900	-0.76625500
C	-0.32761600	3.42567900	-0.67292800
C	0.01548200	4.56803500	0.07490500

C	-0.68232400	3.62060500	-2.02252100
C	0.04603300	5.84250600	-0.50762100
H	0.27534000	4.46705600	1.13311900
C	-0.65271800	4.89094500	-2.60964400
H	-0.97841300	2.76229700	-2.63279300
C	-0.28078300	6.01068100	-1.85663100
H	0.33022400	6.70865700	0.09847900
H	-0.92107400	5.00714900	-3.66463800
H	-0.25305700	7.00327300	-2.31464400
C	6.37675300	0.67424200	0.72409700
H	5.54217700	1.27624800	1.11231600
C	6.86674200	-3.62204500	-1.92391300
H	6.32029100	-4.57528600	-2.03315700
C	2.75761300	-2.95171900	0.98151100
H	2.11162700	-2.09784800	1.23594800
C	7.39904700	0.52071700	1.86397100
H	8.24932100	-0.10318900	1.54158200
H	7.79392500	1.50318400	2.17162100
H	6.94684700	0.04492600	2.74615900
C	6.99378400	1.45238000	-0.44931700
H	7.22966400	2.48335800	-0.14034300
H	7.93325100	0.99406800	-0.79804500
H	6.30911800	1.50297200	-1.30975800
C	1.96644300	-3.82210200	-0.00516200
H	0.95775000	-4.00572200	0.39494900
H	1.85561200	-3.33024200	-0.98396600
H	2.44399100	-4.80157300	-0.17337100
C	3.01150500	-3.73071800	2.28503100
H	3.50053600	-3.10039900	3.04105200
H	2.05949800	-4.08861900	2.71009300
H	3.65831500	-4.60493900	2.09928200
C	8.27308200	-3.95431100	-1.40043200
H	8.80034800	-4.62893500	-2.09493300
H	8.88615300	-3.04436700	-1.29390100
H	8.22628600	-4.44292600	-0.41458300
C	6.93006100	-2.96411300	-3.31321200
H	5.92019200	-2.75485300	-3.69969500
H	7.47442700	-2.00627000	-3.27088100
H	7.44953000	-3.61476700	-4.03619800
C	3.48582700	-0.37315200	-2.30911400
H	3.59481400	0.46463200	-3.01418100
H	4.38102000	-0.41106700	-1.68234700
H	3.46937000	-1.30064600	-2.90524400
C	0.98164300	-0.18089800	-2.46088500
H	0.02806200	-0.24289900	-1.91790400
H	0.98019300	0.75976200	-3.03400700
H	1.03153500	-1.01107100	-3.18655900
C	3.57099800	3.45604700	0.31594500
H	3.95669900	3.01774000	1.24734400
H	2.65751900	4.02160600	0.55351900
C	2.87991500	3.08220100	-2.07659600
H	2.01402000	3.73994500	-1.92486300
H	3.71413400	3.69830500	-2.45313500
H	2.61718200	2.36555900	-2.86720700

H	4.32069400	4.17550900	-0.05461500
Br	-0.54868600	-0.86824600	1.13486500
C	-2.26264100	1.92642900	0.44324600
C	-3.28257400	1.41596800	-0.26883000
C	-2.77674500	2.67159900	1.65214900
H	-3.23591700	0.77328900	-1.14774600
C	-4.30853200	2.77223100	1.43900300
H	-2.30162900	3.66081600	1.75249700
H	-2.52554400	2.09832600	2.56040900
C	-4.65926600	1.63579100	0.44400400
H	-4.88948300	2.70199200	2.36932500
H	-4.53652300	3.74029900	0.97742700
C	-5.66072900	1.93987800	-0.66063000
C	-4.86543800	0.28930100	1.12628900
O	-6.22633600	1.04971700	-1.28689100
C	-5.85238900	3.37530400	-1.08833200
O	-5.30511300	-0.72429100	0.59402700
O	-4.39829400	0.28121800	2.36913000
H	-6.44705200	3.39241100	-2.01025500
H	-6.37020800	3.95011700	-0.30387400
H	-4.87582300	3.85431800	-1.25844800
C	-4.07988600	-0.98046500	2.97118700
H	-3.89642600	-0.76889500	4.03149700
H	-4.90822300	-1.69489500	2.85843000
H	-3.16097300	-1.37098200	2.50535500
Li	-5.86516400	-0.84731100	-1.28486900
O	-7.32892100	-2.07494100	-1.13457900
C	-7.70887800	-2.55158500	-0.06635900
N	-8.74407800	-3.40515100	0.06879700
H	-7.20210800	-2.29867000	0.88885500
C	-9.13492600	-3.92784700	1.36398400
C	-9.51356800	-3.83440700	-1.08789200
H	-9.06590600	-5.02928200	1.37997600
H	-8.47663000	-3.52632600	2.14749700
H	-10.17492000	-3.64631900	1.60332300
H	-9.46061500	-4.93062400	-1.19999900
H	-10.57250000	-3.54712800	-0.97340300
H	-9.10192300	-3.35641600	-1.98496900
O	-4.37386400	-1.36099800	-2.27375200
C	-3.21689300	-1.75716800	-2.44010700
N	-2.54368300	-2.59251100	-1.63985400
H	-2.62631200	-1.42590600	-3.32080500
C	-1.17074500	-2.98109500	-1.92279700
C	-3.11629800	-3.10622900	-0.40455500
H	-1.08753200	-4.08038400	-1.96145100
H	-0.85334900	-2.56848400	-2.88936000
H	-0.50853200	-2.59342300	-1.13383800
H	-3.26674400	-4.19739600	-0.48100000
H	-2.42604700	-2.88352100	0.42304700
H	-4.07130000	-2.61111600	-0.20302800

IM5

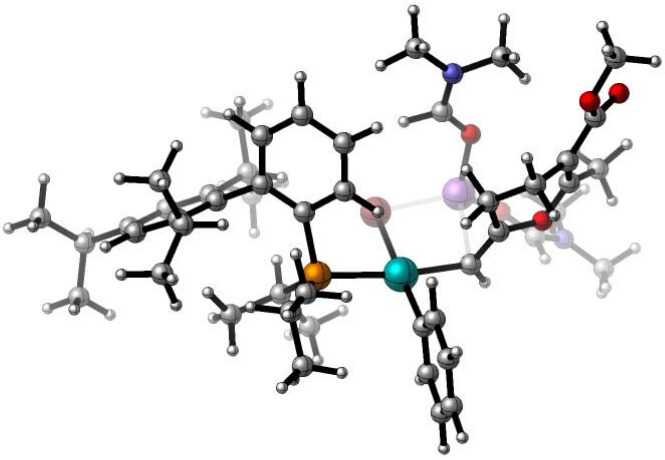

E (B3LYP-D3/Def2-SVP)) = -5406.77383678
E (SMD(DMF)/M06/Def2-TZVPP//B3LYP-D3/Def2-SVP) = -5407.80156925

Zero-point correction=	1.103235 (Hartree/Particle)
Thermal correction to Energy=	1.174825
Thermal correction to Enthalpy=	1.175769
Thermal correction to Gibbs Free Energy=	0.987560

Charge = 0 Multiplicity = 1

P	-1.84406600	1.26278800	-0.33377400
C	-2.80639500	2.22650600	-1.63303300
H	-3.85031000	1.90245500	-1.50443000
C	-2.85441300	1.46189500	1.25140500
H	-2.76717800	0.46974900	1.71092800
C	-1.92285900	-0.49096400	-0.99473800
C	-2.93587300	-1.47309200	-0.88390700
C	-0.76577900	-0.81608600	-1.73650000
C	-2.73754600	-2.72552800	-1.50088800
C	-0.59607300	-2.04985100	-2.35908200
H	0.03241500	-0.07323600	-1.80662400
C	-1.59064100	-3.02217300	-2.23456400
H	-3.52322200	-3.47760900	-1.39937100
H	0.31472400	-2.24744300	-2.93006800
H	-1.47994100	-4.00228600	-2.70655200
C	-4.23993700	-1.30160100	-0.16183600
C	-5.38844100	-0.88911400	-0.88009700
C	-4.34134300	-1.64121300	1.20737500
C	-6.61351600	-0.80426900	-0.20716100
C	-5.58710000	-1.52147500	1.83833400
C	-6.73285100	-1.10334300	1.15483300
H	-7.49845300	-0.48005700	-0.75834900
H	-5.67174400	-1.76339000	2.90030200
C	0.78786800	3.20586700	-1.03532200
C	0.99365800	3.15219800	-2.42543300
C	0.73025800	4.48068400	-0.43801200
C	1.10261900	4.31840400	-3.19436500
H	1.05593100	2.18627300	-2.93435300

C	0.83744600	5.64836000	-1.20262300
H	0.58355600	4.56835600	0.64226200
C	1.01871800	5.57499600	-2.58810500
H	1.25055000	4.24066700	-4.27603900
H	0.77713200	6.62411500	-0.71045100
H	1.09963100	6.48640200	-3.18658900
C	-5.32158100	-0.56001900	-2.36967000
H	-4.26834200	-0.36296400	-2.61612800
C	-8.06115800	-0.94585100	1.88032600
H	-7.90949400	-1.30127900	2.91452700
C	-3.13781700	-2.17366500	1.98113100
H	-2.22740800	-1.81521700	1.47915300
C	-5.77111600	-1.76403600	-3.21628000
H	-6.81725400	-2.02870500	-2.98943200
H	-5.70246400	-1.53458900	-4.29258500
H	-5.14904100	-2.64880600	-3.01694800
C	-6.12069200	0.69532300	-2.75358600
H	-5.89892600	0.98426100	-3.79339500
H	-7.20762800	0.52737600	-2.68736400
H	-5.87582500	1.54890500	-2.10323100
C	-3.06244100	-1.68392800	3.43456800
H	-2.07934000	-1.93690100	3.85949600
H	-3.17850900	-0.59177200	3.50438000
H	-3.83501000	-2.14843300	4.06930900
C	-3.10400800	-3.71236700	1.93618000
H	-3.06825900	-4.08581400	0.90230600
H	-2.21555800	-4.09326400	2.46634100
H	-3.99995100	-4.13792400	2.41860500
C	-9.17187900	-1.80498000	1.25546400
H	-10.10432700	-1.72267400	1.83770900
H	-9.39605500	-1.48304000	0.22523100
H	-8.88186100	-2.86676800	1.21848400
C	-8.47633700	0.53363000	1.95640800
H	-7.69153600	1.14236500	2.43216500
H	-8.65217900	0.94591100	0.94904800
H	-9.40642600	0.65387000	2.53617400
C	-4.33772100	1.82262700	1.11404200
H	-4.47231000	2.85305200	0.75150500
H	-4.88839700	1.14990100	0.44998500
H	-4.81662900	1.76141400	2.10497400
C	-2.14879900	2.46353300	2.18367900
H	-1.12221800	2.15067900	2.41788000
H	-2.10770500	3.47315200	1.74401900
H	-2.70912000	2.53564700	3.13133200
C	-2.37740800	1.89991100	-3.06818800
H	-2.48066900	0.83225500	-3.30913900
H	-1.33316700	2.19441500	-3.24556300
C	-2.71886000	3.73816900	-1.37541900
H	-1.70627400	4.11628900	-1.57098700
H	-3.41632500	4.26741000	-2.04648700
H	-2.98274000	4.01111300	-0.34376500
H	-3.00689300	2.46650900	-3.77490300
Br	0.26599800	-0.43916100	1.91840500
Pd	0.51295900	1.58696200	0.11263800

C	2.52788300	1.61534500	0.41245800
C	3.39505800	0.89241700	-0.30880700
H	2.92918500	2.12578700	1.30069400
C	3.11928100	0.13610700	-1.58076300
C	4.38316000	-0.04164200	-2.42730800
H	2.70341800	-0.85749700	-1.34498700
H	2.34338500	0.67750700	-2.13639300
C	5.49668500	-0.61047900	-1.57412300
H	4.68715000	0.93661400	-2.84259000
H	4.18041600	-0.69542800	-3.28415500
C	5.60538100	-0.13524700	-0.29696500
C	6.39861600	-1.64641100	-2.08745800
O	4.72510800	0.76037500	0.19138500
C	6.62683200	-0.47973800	0.74248700
O	6.13900500	-1.93594500	-3.39477700
O	7.26690700	-2.25151100	-1.48261200
Li	2.71828500	-0.56320800	1.99488700
H	6.15483200	-1.14328100	1.48754200
H	7.49487100	-0.98976300	0.31754500
H	6.93062700	0.44016300	1.26604000
C	6.96092100	-2.93677600	-3.98333400
H	6.84954500	-3.90061600	-3.46038700
H	6.63222900	-3.03079200	-5.02664200
H	8.02409100	-2.65253600	-3.94266400
O	3.96353000	0.03315600	3.25621300
C	4.82278300	0.91541600	3.27445200
N	5.75329200	1.05334400	4.23755600
H	4.89662800	1.66589500	2.46427100
C	6.74945600	2.10661400	4.18037500
C	5.81603400	0.12943700	5.35799100
H	7.76635800	1.68044800	4.13203400
H	6.58924100	2.72809600	3.28807200
H	6.68834300	2.75227000	5.07303600
H	6.78405300	-0.39988900	5.36538800
H	5.70902800	0.67405600	6.31110400
H	5.00319200	-0.60045600	5.26242500
O	3.25587900	-2.09211300	1.01656900
C	2.49059300	-2.78744100	0.34085100
N	2.88492300	-3.68233200	-0.58271900
H	1.38989000	-2.69529800	0.44938900
C	1.91771400	-4.46219100	-1.33265000
C	4.29214800	-3.89327900	-0.88688500
H	2.05803100	-5.54124900	-1.14814000
H	0.89650300	-4.17929000	-1.04337200
H	2.03260600	-4.28152500	-2.41520400
H	4.56857200	-4.94666800	-0.71300400
H	4.49189300	-3.64680100	-1.94249800
H	4.90665500	-3.24479500	-0.25403800

TS4

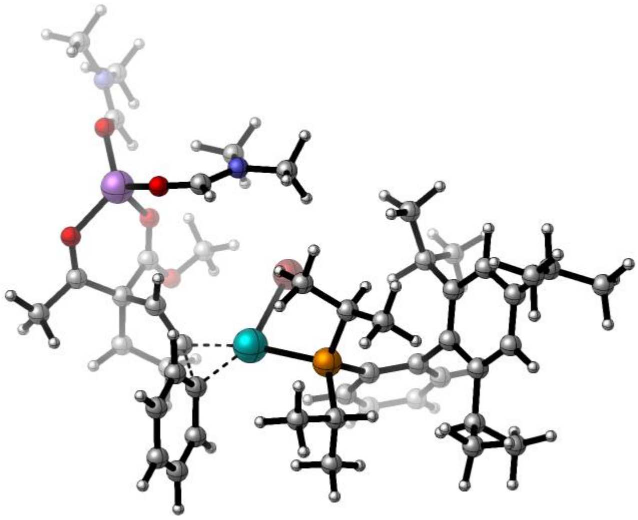

E (B3LYP-D3/Def2-SVP)) = -5406.76562824
E (SMD(DMF)/M06/Def2-TZVPP//B3LYP-D3/Def2-SVP) = -5407.80187775

Zero-point correction=	1.102851 (Hartree/Particle)
Thermal correction to Energy=	1.173264
Thermal correction to Enthalpy=	1.174208
Thermal correction to Gibbs Free Energy=	0.991272

Charge = 0 Multiplicity = 1

Pd	-0.38758600	1.52341000	0.14458800
P	1.95633000	1.11873300	-0.19357400
C	3.07026800	2.53899100	-0.73830000
H	4.05909400	2.10758100	-0.95928700
C	2.21938500	-0.22317300	-1.50196500
H	2.19295700	-1.14553200	-0.90893500
C	2.69560100	0.63763500	1.45588800
C	3.77430700	-0.21980000	1.77433000
C	2.04455000	1.30010100	2.51940400
C	4.15889900	-0.36096700	3.12291700
C	2.44676300	1.16274900	3.84543100
H	1.18336400	1.93235800	2.28767600
C	3.51728900	0.32208700	4.15361200
H	4.98877800	-1.03328500	3.35347600
H	1.91256800	1.69884400	4.63385100
H	3.84483800	0.18859100	5.18796900
C	4.56188800	-1.02722000	0.78719100
C	5.79625900	-0.53452700	0.30177100
C	4.11537000	-2.31812800	0.41787300
C	6.56201700	-1.34170500	-0.54895300
C	4.90790700	-3.07666200	-0.45455800
C	6.13118600	-2.61142200	-0.94835500
H	7.51311700	-0.96029900	-0.92653800
H	4.56728700	-4.06856200	-0.76041300
C	-1.00777200	3.34140800	-0.62479500
C	-0.57646200	4.52312500	0.02365300
C	-1.24843600	3.43021500	-2.02031600
C	-0.35248800	5.70796900	-0.68602100
H	-0.38346400	4.50471000	1.09923000

C	-1.02292600	4.60745800	-2.72734000
H	-1.62230600	2.54988800	-2.55007100
C	-0.56872100	5.75974200	-2.06554300
H	0.00432100	6.59524100	-0.15435300
H	-1.20185400	4.63310100	-3.80649600
H	-0.39387800	6.68480600	-2.62079900
C	6.30584700	0.85192000	0.68942000
H	5.44721300	1.41967400	1.07818700
C	6.94947700	-3.44922800	-1.92029600
H	6.43353900	-4.41978300	-2.02558700
C	2.82151200	-2.89858300	0.98789400
H	2.14633800	-2.06444300	1.23454500
C	7.34545300	0.76142700	1.81996300
H	8.22015100	0.17236700	1.49714900
H	7.69829400	1.76499200	2.11033600
H	6.92377300	0.27779300	2.71305100
C	6.87160800	1.64152800	-0.50195700
H	7.06619300	2.68585900	-0.20926900
H	7.82526900	1.22036800	-0.85918200
H	6.17267100	1.65041200	-1.35223200
C	2.06001000	-3.80201900	0.00788300
H	1.06188000	-4.02273800	0.41542100
H	1.92458900	-3.31733600	-0.97147500
H	2.57426200	-4.76287100	-0.15975200
C	3.09763100	-3.65888200	2.29795800
H	3.56716900	-3.00832300	3.04904200
H	2.15611200	-4.04097200	2.72513400
H	3.77020900	-4.51496100	2.11913200
C	8.36522200	-3.73450200	-1.39435500
H	8.91463900	-4.39476700	-2.08555800
H	8.94858900	-2.80487300	-1.29087800
H	8.33296300	-4.22023600	-0.40650000
C	6.99348800	-2.79614600	-3.31262300
H	5.97790000	-2.61979000	-3.70055100
H	7.50796300	-1.82175100	-3.27446000
H	7.53329300	-3.43373300	-4.03241500
C	3.51514800	-0.19388900	-2.31871600
H	3.54555600	0.67018300	-2.99993300
H	4.41324000	-0.17052000	-1.69465300
H	3.57835500	-1.10217700	-2.94122900
C	0.99855400	-0.24440000	-2.43906700
H	0.06678500	-0.39311500	-1.87531000
H	0.90131200	0.69497800	-3.00616600
H	1.10930600	-1.06389200	-3.17083600
C	3.23078600	3.59002500	0.36659800
H	3.72092500	3.18623500	1.26428900
H	2.25145200	3.99788700	0.66443400
C	2.50213700	3.18806300	-2.00840000
H	1.52569800	3.65184600	-1.81044400
H	3.18569700	3.97708900	-2.36540600
H	2.36920100	2.47089300	-2.83092400
H	3.84192300	4.43007700	-0.00483500
Br	-0.68766300	-0.96976900	1.17318700
C	-2.27363700	2.27724000	0.31690200

C	-3.29696100	1.66083000	-0.33907900
C	-2.78396800	2.83669800	1.63600900
H	-3.24045800	1.09342400	-1.26789500
C	-4.32050100	2.82122300	1.48735800
H	-2.40122600	3.84332700	1.85651400
H	-2.45567700	2.17074000	2.45005700
C	-4.61030600	1.68235800	0.47586500
H	-4.85404200	2.68448900	2.43779100
H	-4.64019200	3.77787500	1.05632600
C	-5.70597500	1.91714100	-0.56105100
C	-4.67899700	0.30379500	1.13801900
O	-6.19618100	0.99168200	-1.20154900
C	-6.08829100	3.33674100	-0.90924700
O	-5.01860400	-0.73236200	0.58132400
O	-4.24949000	0.31932300	2.39133600
H	-6.73633800	3.31865700	-1.79455000
H	-6.62162400	3.81403900	-0.07123700
H	-5.18932600	3.93785600	-1.11417900
C	-3.96316200	-0.93487700	3.02912300
H	-3.77789500	-0.69460200	4.08294000
H	-4.81552500	-1.62405000	2.93551500
H	-3.05634500	-1.36588900	2.57638200
Li	-5.60091900	-0.84591500	-1.28385100
O	-6.85525500	-2.29706900	-1.12828700
C	-7.14314200	-2.84566700	-0.06504800
N	-8.05467200	-3.82957200	0.06934600
H	-6.64574200	-2.55392700	0.88361300
C	-8.33641400	-4.43354200	1.35777800
C	-8.79308100	-4.32680500	-1.08048700
H	-8.12239200	-5.51607600	1.33874700
H	-7.71445300	-3.96928100	2.13624800
H	-9.39717900	-4.29857200	1.63075900
H	-8.60102800	-5.40359200	-1.22414900
H	-9.87679700	-4.18336100	-0.93268700
H	-8.47171500	-3.77742900	-1.97355200
O	-4.08978200	-1.13637600	-2.34285500
C	-2.96860800	-1.64140400	-2.46329400
N	-2.46903100	-2.64055000	-1.72646000
H	-2.26843600	-1.28218800	-3.24656600
C	-1.09116000	-3.08295100	-1.87819900
C	-3.20103900	-3.18482000	-0.59210600
H	-1.05485800	-4.18142300	-1.96474800
H	-0.65262000	-2.64343800	-2.78289100
H	-0.50200600	-2.76082800	-1.00561100
H	-3.05766800	-4.27646000	-0.55931500
H	-2.82014600	-2.73449100	0.33845900
H	-4.26732000	-2.96042900	-0.69841500

TS5

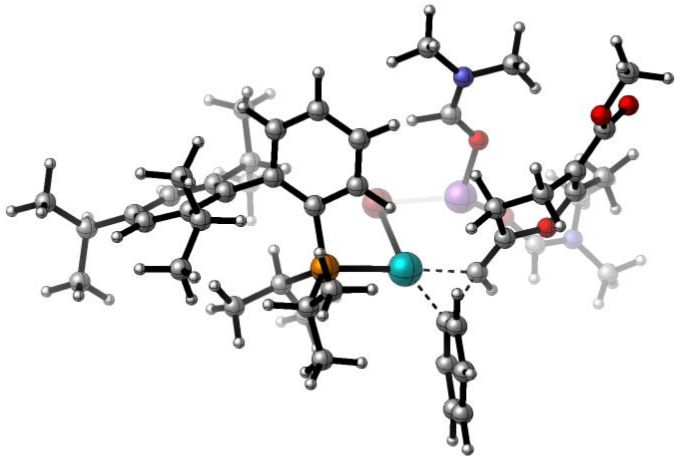

E (B3LYP-D3/Def2-SVP)) = -5406.75418125
E (SMD(DMF)/M06/Def2-TZVPP//B3LYP-D3/Def2-SVP) = -5407.78918724

Zero-point correction=	1.102429 (Hartree/Particle)
Thermal correction to Energy=	1.173476
Thermal correction to Enthalpy=	1.174420
Thermal correction to Gibbs Free Energy=	0.989628

Charge = 0 Multiplicity = 1

P	-1.78804800	1.14474800	-0.51390200
C	-2.49139100	1.93411600	-2.07215000
H	-3.57779500	1.75529500	-2.06626700
C	-2.94921000	1.68800400	0.87411000
H	-3.01455800	0.78860400	1.49722000
C	-1.94169600	-0.69483100	-0.85047000
C	-3.00838100	-1.59475600	-0.61855900
C	-0.76156700	-1.21117100	-1.43020700
C	-2.84375000	-2.95172100	-0.96646800
C	-0.62241900	-2.54862000	-1.79036200
H	0.07773400	-0.52875000	-1.58219500
C	-1.67436000	-3.43534400	-1.54965000
H	-3.67399200	-3.63503500	-0.77349500
H	0.30936400	-2.89255300	-2.24686700
H	-1.58920100	-4.49312100	-1.81329000
C	-4.33878500	-1.23239700	-0.02869700
C	-5.41837700	-0.91110100	-0.88541400
C	-4.53482900	-1.30284200	1.36999300
C	-6.67700700	-0.66292100	-0.32437700
C	-5.80912100	-1.02439200	1.88295400
C	-6.89194100	-0.70416200	1.05799200
H	-7.51034500	-0.41147700	-0.98358800
H	-5.96754400	-1.05875600	2.96339300
C	1.44498400	2.91750800	-1.16129400
C	1.52056800	2.82255100	-2.57419800
C	1.41018800	4.22689900	-0.61575500
C	1.51822700	3.95713500	-3.38439500
H	1.56018100	1.84238500	-3.05222600
C	1.39295800	5.36153800	-1.43016800
H	1.37932000	4.35033500	0.47051400

C	1.44458500	5.23885600	-2.82251000
H	1.55868000	3.83888500	-4.47142800
H	1.33900800	6.35268800	-0.96977600
H	1.43218500	6.12569100	-3.46095100
C	-5.23629600	-0.84273400	-2.40021400
H	-4.16014600	-0.72537100	-2.59653400
C	-8.25408900	-0.37421500	1.65096000
H	-8.18075400	-0.53554200	2.74069300
C	-3.39942300	-1.70819800	2.30800600
H	-2.44703000	-1.48138900	1.80603500
C	-5.68256000	-2.15572400	-3.06689700
H	-6.75276900	-2.34254400	-2.87771500
H	-5.53081800	-2.11397600	-4.15831200
H	-5.11641100	-3.01444200	-2.67729700
C	-5.94647200	0.35874300	-3.04355500
H	-5.64494800	0.45917800	-4.09856200
H	-7.04248900	0.24605500	-3.02781100
H	-5.70125900	1.29966800	-2.52770200
C	-3.38622100	-0.94215400	3.63901400
H	-2.43953800	-1.13481300	4.16584400
H	-3.45869700	0.14511700	3.48465200
H	-4.21281100	-1.24753200	4.30175500
C	-3.42469200	-3.22557300	2.56707000
H	-3.34593100	-3.79682800	1.63046200
H	-2.58313500	-3.51839200	3.21621600
H	-4.36260300	-3.52195900	3.06621000
C	-9.35947000	-1.30129400	1.12082900
H	-10.32183700	-1.08448000	1.61297900
H	-9.50585800	-1.17054300	0.03603100
H	-9.11212400	-2.35947900	1.29961500
C	-8.61138500	1.10519600	1.42596900
H	-7.83213300	1.76820400	1.83346100
H	-8.70849700	1.32812400	0.35054000
H	-9.56912600	1.35876700	1.90990800
C	-4.36563600	2.12411500	0.48545200
H	-4.35971500	3.07629100	-0.06718900
H	-4.89383600	1.37903500	-0.11766900
H	-4.96244200	2.28140100	1.39906100
C	-2.26204000	2.77791700	1.71581300
H	-1.30534000	2.42819000	2.12739600
H	-2.07002600	3.68980300	1.12762400
H	-2.91736000	3.05639700	2.55899300
C	-1.90331100	1.31656700	-3.34565900
H	-2.14966100	0.24959900	-3.44842400
H	-0.80733100	1.41900500	-3.36236100
C	-2.22432700	3.44688700	-2.03211400
H	-1.14485800	3.65793900	-2.04790000
H	-2.67861900	3.93221600	-2.91248300
H	-2.64555400	3.92630900	-1.13673000
H	-2.29898800	1.84203700	-4.23112900
Br	0.14229000	-0.15007600	2.25116500
Pd	0.52918700	1.48964200	0.00085900
C	2.55840700	1.78586000	-0.06034000
C	3.35957600	0.81180800	-0.56220700

H	3.03392600	2.45425100	0.66796100
C	3.01812700	-0.20670900	-1.60691700
C	4.25104500	-0.59386700	-2.43016600
H	2.61032600	-1.11210700	-1.12912700
H	2.21559700	0.19299600	-2.23975100
C	5.36759500	-0.99926300	-1.49273200
H	4.56371000	0.26334600	-3.05420300
H	4.01016400	-1.41229600	-3.11925300
C	5.50816100	-0.28180600	-0.33753000
C	6.23137100	-2.14683600	-1.79340800
O	4.65839700	0.72307400	-0.03136300
C	6.52909000	-0.44021100	0.74409700
O	5.95334400	-2.67610700	-3.01772300
O	7.08317200	-2.64866600	-1.08060400
Li	2.54840200	-0.11487000	2.05001100
H	6.04329300	-0.92106700	1.61036300
H	7.37496600	-1.05462900	0.42634400
H	6.86340900	0.55715200	1.06813300
C	6.73316700	-3.80381100	-3.39850400
H	6.59234900	-4.63976400	-2.69429600
H	6.39077200	-4.09015400	-4.40146500
H	7.80601300	-3.55613900	-3.41788900
O	3.90935700	0.72985200	3.00623100
C	4.72539200	1.63940000	2.87808800
N	5.93498600	1.67231500	3.46854000
H	4.51999000	2.52477900	2.24329600
C	6.86358900	2.76231300	3.23667700
C	6.39031600	0.57019900	4.29954000
H	7.78012100	2.39455100	2.74339500
H	6.40186800	3.52070200	2.58883200
H	7.15155900	3.24014200	4.18828000
H	7.26725400	0.08242400	3.84107200
H	6.67490400	0.93711200	5.29954100
H	5.57949000	-0.16160500	4.39604000
O	3.10675400	-1.79155900	1.34338200
C	2.32196300	-2.66103700	0.95005800
N	2.68352000	-3.75803300	0.25938600
H	1.23200700	-2.57099100	1.13785300
C	1.69889000	-4.73608700	-0.16555500
C	4.07231500	-4.01118200	-0.09274700
H	1.89170500	-5.71595100	0.30459300
H	0.68914600	-4.39945900	0.10562600
H	1.73458600	-4.86787500	-1.26037400
H	4.39043100	-4.99532000	0.28948800
H	4.19768500	-4.00392100	-1.18841000
H	4.70885200	-3.23057200	0.33618400

Br^-

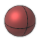

E (B3LYP-D3/Def2-SVP)) = -2573.92677842
E (SMD(DMF)/M06/Def2-TZVPP//B3LYP-D3/Def2-SVP) = -2574.22345699

Zero-point correction=	0.000000 (Hartree/Particle)
Thermal correction to Energy=	0.001416
Thermal correction to Enthalpy=	0.002360
Thermal correction to Gibbs Free Energy=	-0.016176

Charge = -1 Multiplicity = 1

Br	-0.20714286	0.84285713	0.00000000

$FeCl_3(DMF)_2$

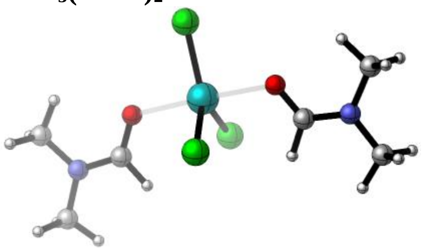

E (B3LYP-D3/Def2-SVP)) = -3140.68120569
E (SMD(DMF)/M06/Def2-TZVPP//B3LYP-D3/Def2-SVP) = -3141.40624735

Zero-point correction=	0.212023 (Hartree/Particle)
Thermal correction to Energy=	0.232959
Thermal correction to Enthalpy=	0.233903
Thermal correction to Gibbs Free Energy=	0.156030

Charge = 0 Multiplicity = 6

Fe	-0.00006400	0.57987800	-0.00152500
Cl	0.00024200	-0.72676100	-1.85686400
Cl	-0.00019600	-0.72552800	1.85439500
Cl	0.00000600	2.78359500	-0.00094300
O	-2.13857700	0.53099500	-0.00197300
C	-2.72912700	-0.55718100	-0.00162400
N	-4.06212400	-0.67904000	0.00153200
H	-2.16529600	-1.50751500	-0.00419900
C	-4.70328900	-1.98116600	0.00162400
C	-4.92111100	0.49617800	0.00524500
H	-5.34078600	-2.10054100	-0.89084200
H	-3.94360200	-2.77499100	-0.00160000
H	-5.33569300	-2.10292200	0.89738900
H	-5.56681400	0.49826500	-0.88867600
H	-5.56278400	0.49547300	0.90206100
H	-4.29420300	1.39591700	0.00522800
O	2.13855300	0.53091000	-0.00139400
C	2.72914900	-0.55721500	-0.00140500
N	4.06216300	-0.67902100	0.00183900
H	2.16536800	-1.50758600	-0.00416900
C	4.70337600	-1.98111500	0.00107000
C	4.92108200	0.49623800	0.00564200

H	5.33659700	-2.10300800	0.89623300
H	3.94371800	-2.77497100	-0.00159200
H	5.34007000	-2.10031900	-0.89200100
H	5.56352900	0.49491600	0.90189700
H	5.56602100	0.49904500	-0.88883800
H	4.29412100	1.39593900	0.00679900

$FeCl_3Br^-$

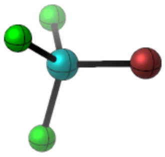

E (B3LYP-D3/Def2-SVP)) = -5217.96639688
E (SMD(DMF)/M06/Def2-TZVPP//B3LYP-D3/Def2-SVP) = -5218.72640642

Zero-point correction=	0.004334 (Hartree/Particle)
Thermal correction to Energy=	0.012273
Thermal correction to Enthalpy=	0.013217
Thermal correction to Gibbs Free Energy=	-0.033463

Charge = -1 Multiplicity = 6

Fe	-0.41947900	-0.00008500	-0.00007000
Cl	-1.14666700	0.70332500	1.98752300
Cl	-1.14544800	-2.07357200	-0.38414100
Cl	-1.14778300	1.36905000	-1.60287800
Br	1.98242100	0.00064400	-0.00019300

IM6

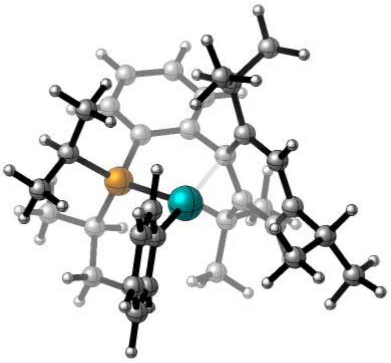

E (B3LYP-D3/Def2-SVP)) = -1753.46403196
E (SMD(DMF)/M06/Def2-TZVPP//B3LYP-D3/Def2-SVP) = -1753.89336381

Zero-point correction=	0.706327 (Hartree/Particle)
Thermal correction to Energy=	0.745967
Thermal correction to Enthalpy=	0.746911
Thermal correction to Gibbs Free Energy=	0.634076

Charge = 1 Multiplicity = 1

Pd	-0.67767700	0.07582300	-0.03605900
P	0.90016200	1.66753100	-0.24409600
C	0.85536900	3.14974000	0.88092100
H	1.82502300	3.64666900	0.70614700
C	1.19197200	2.24096200	-2.01118000
H	1.65018900	1.34336000	-2.45914200

C	2.45538300	0.75998400	0.19444400
C	2.44827400	-0.65413300	0.27254100
C	3.65987000	1.45343700	0.40769000
C	3.65385700	-1.33109300	0.50561200
C	4.84844700	0.76417000	0.65677600
H	3.68250900	2.54401300	0.39119900
C	4.84839500	-0.63213600	0.69088200
H	3.64817100	-2.42200000	0.55573400
H	5.77352400	1.32070500	0.82222000
H	5.77616000	-1.17822200	0.87574500
C	1.17101200	-1.44193300	0.17053300
C	0.69695200	-1.90244500	-1.09642400
C	0.45917100	-1.78135900	1.36965100
C	-0.52288700	-2.60438100	-1.15453900
C	-0.76091600	-2.46008800	1.24572100
C	-1.28733400	-2.85621500	-0.00493800
H	-0.88912300	-2.93760000	-2.12392200
H	-1.33627600	-2.68768900	2.14473200
C	-2.27580200	1.25452200	0.09983000
C	-2.78459400	1.50865200	1.38436500
C	-3.04609100	1.57645700	-1.02697000
C	-4.06954800	2.04474600	1.53673200
H	-2.19923300	1.28036800	2.27750700
C	-4.33110300	2.11161700	-0.86568300
H	-2.67164000	1.39898400	-2.03571200
C	-4.84327800	2.34925100	0.41285000
H	-4.46145800	2.22754900	2.54065300
H	-4.93087800	2.34478500	-1.74937700
H	-5.84220200	2.77477700	0.53345000
C	1.51401400	-1.68578500	-2.36307100
H	2.22528100	-0.87073000	-2.15986000
C	-2.67403700	-3.47414300	-0.05918600
H	-2.75782100	-4.13470400	0.82205700
C	1.00536100	-1.42139500	2.74613000
H	1.74446800	-0.61866700	2.60601400
C	2.34481400	-2.94808300	-2.66187200
H	1.68840000	-3.81242900	-2.85171100
H	2.97148700	-2.79347700	-3.55390300
H	3.00651400	-3.20348500	-1.82054200
C	0.66710900	-1.27506100	-3.57530900
H	1.32022400	-0.98970900	-4.41400300
H	0.02802900	-2.09793900	-3.93124600
H	0.01452100	-0.42054500	-3.34061200
C	-0.06624000	-0.89193500	3.70875600
H	0.40574200	-0.52422200	4.63231300
H	-0.63106600	-0.06164600	3.25967000
H	-0.78525800	-1.67247500	4.00236300
C	1.74221800	-2.63270400	3.34796400
H	2.55617000	-2.97904300	2.69409900
H	2.17842100	-2.37079700	4.32437600
H	1.04872100	-3.47551000	3.49948200
C	-2.93822400	-4.31896100	-1.30929500
H	-3.90729700	-4.83177500	-1.21867900
H	-2.98703400	-3.69667800	-2.21767500

H	-2.16195600	-5.08547000	-1.45779700
C	-3.73320600	-2.36135500	0.09317800
H	-3.59075400	-1.78425700	1.01975800
H	-3.67555800	-1.65320200	-0.74987500
H	-4.74634900	-2.79110200	0.11037800
C	2.19301900	3.39934000	-2.12179600
H	1.80215100	4.32657600	-1.67657600
H	3.16284400	3.16904000	-1.66024900
H	2.38348500	3.60539200	-3.18695100
C	-0.11137900	2.52974300	-2.76468000
H	-0.75410800	1.64112500	-2.80783700
H	-0.69268500	3.34344600	-2.30798800
H	0.12455800	2.82411000	-3.79928000
C	0.79012700	2.69715500	2.34492000
H	1.60419200	2.00454100	2.60606300
H	-0.16704300	2.20178100	2.56001100
C	-0.26484700	4.13659400	0.52822600
H	-1.25687500	3.67386400	0.62504000
H	-0.22091300	4.99065800	1.22223700
H	-0.16881800	4.53738700	-0.49043400
H	0.86900200	3.57440100	3.00563200

IM7

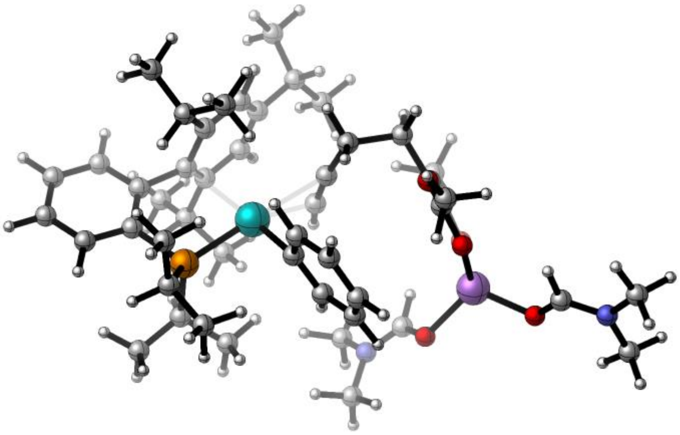

E (B3LYP-D3/Def2-SVP)) = -2832.67126731
E (SMD(DMF)/M06/Def2-TZVPP//B3LYP-D3/Def2-SVP) = -2833.55133874

Zero-point correction= 1.101661 (Hartree/Particle)
Thermal correction to Energy= 1.171357
Thermal correction to Enthalpy= 1.172301
Thermal correction to Gibbs Free Energy= 0.993421

Charge = 1 Multiplicity = 1

P	2.65762800	-2.12863700	-0.23340600
C	2.56158800	-3.48043100	-1.52041200
H	3.41235700	-4.13602800	-1.27239200
C	2.61238100	-2.95602200	1.46488100
H	3.12961400	-2.20410000	2.08279400
C	4.39945300	-1.53183000	-0.33513200
C	4.67659900	-0.16604400	-0.11015500
C	5.46525800	-2.41941600	-0.57821900
C	6.01849300	0.25611900	-0.08569000

C	6.78856400	-1.97949400	-0.56655500
H	5.27088500	-3.47173200	-0.78869600
C	7.06761300	-0.63598200	-0.30469800
H	6.23133900	1.31200300	0.09656600
H	7.59842800	-2.68651100	-0.75957900
H	8.10020800	-0.27995300	-0.28448800
C	3.64611700	0.92172400	0.06120600
C	3.33269700	1.40550600	1.36261300
C	3.27749400	1.69734500	-1.08335200
C	2.69263100	2.64578600	1.49616800
C	2.64830100	2.92863100	-0.88614600
C	2.35153500	3.42890400	0.39028900
H	2.45972300	3.00467200	2.49882100
H	2.37855300	3.52959000	-1.75789800
C	-0.30250200	-1.31181300	-0.88850700
C	-0.47584200	-1.39555600	-2.27551200
C	-1.28029600	-1.81901000	-0.02994000
C	-1.61330300	-2.02500100	-2.79730500
H	0.26067500	-0.97988400	-2.96512100
C	-2.41705900	-2.44252500	-0.55878200
H	-1.19016400	-1.71321900	1.04974800
C	-2.57643500	-2.56231500	-1.94080300
H	-1.74094900	-2.08657600	-3.88099600
H	-3.19214800	-2.79492500	0.12358400
H	-3.46508300	-3.05000700	-2.34701800
C	3.72934900	0.62907700	2.61311800
H	4.17627000	-0.32176600	2.28537800
C	1.67911400	4.78876400	0.51447000
H	0.90897200	4.82393100	-0.27783000
C	3.62607200	1.23853200	-2.49661600
H	3.92284200	0.18081700	-2.43843300
C	4.80607900	1.37631000	3.41717300
H	4.43042200	2.34329500	3.78784300
H	5.12295100	0.78136400	4.28833500
H	5.69465200	1.57512200	2.79877000
C	2.50992700	0.28690700	3.48517300
H	2.80903600	-0.33003300	4.34712400
H	2.02545900	1.19348900	3.88212700
H	1.75750900	-0.27505900	2.91028100
C	2.42997600	1.31842100	-3.45720800
H	2.68821100	0.86320400	-4.42589600
H	1.55369700	0.79057100	-3.05217000
H	2.12857500	2.35865100	-3.65708900
C	4.82933300	2.02628100	-3.04358200
H	5.70995600	1.91733800	-2.39336800
H	5.10197300	1.66864200	-4.04906500
H	4.59381000	3.10024200	-3.11750100
C	2.69249000	5.91343200	0.22610800
H	2.19762500	6.89711200	0.24484600
H	3.49039500	5.91782800	0.98650300
H	3.16816800	5.78949500	-0.75890500
C	0.97224900	5.01977300	1.85335100
H	0.27127600	4.20446500	2.08914100
H	1.69188100	5.10546500	2.68370500

H	0.39893900	5.95851400	1.82370400
C	3.40805800	-4.26452100	1.54684500
H	2.94906800	-5.06583700	0.94840800
H	4.45244400	-4.14372300	1.22817100
H	3.42712300	-4.61049900	2.59284600
C	1.19158100	-3.11280000	2.01863100
H	0.67559800	-2.14587900	2.08114100
H	0.56897600	-3.77810600	1.40355800
H	1.23943200	-3.53788600	3.03405300
C	2.79969800	-2.89796800	-2.92028900
H	3.75292900	-2.35193400	-2.98498500
H	1.99091500	-2.21510500	-3.21352600
C	1.28922500	-4.33553100	-1.44373600
H	0.38396500	-3.75767000	-1.66532000
H	1.36233100	-5.14487800	-2.18773000
H	1.16251100	-4.80866100	-0.46015000
H	2.83053100	-3.71408000	-3.65931000
Pd	1.27019400	-0.25994800	-0.22868900
C	-0.31535900	1.61567900	-0.33396700
C	-0.06639900	1.25309400	0.81438800
C	-0.81765600	2.12209000	-1.60422800
H	-0.01442500	1.13548900	1.88229100
C	-2.15171800	2.90790200	-1.40677600
H	-0.04590500	2.74362900	-2.08827900
H	-1.01697000	1.26499100	-2.26717000
C	-3.20928000	2.09111200	-0.70222700
H	-2.47945200	3.20158500	-2.41536800
H	-1.93076000	3.83656600	-0.86382400
C	-3.52195500	2.28900300	0.67043800
C	-3.86403100	1.05522700	-1.44173300
O	-4.28728800	1.55454900	1.35261800
C	-2.90692900	3.47635900	1.41307100
O	-4.70615500	0.23269600	-1.04371500
O	-3.48539400	1.01925500	-2.75959800
Li	-5.11877000	-0.04311400	0.80364900
H	-3.31113200	3.48932600	2.43367000
H	-3.14855600	4.43413300	0.92486800
H	-1.80900300	3.40663500	1.46202100
C	-4.23553900	0.17934300	-3.62362100
O	-7.00598900	-0.37461300	0.79051900
O	-4.10421700	-1.34472700	1.93289900
H	-3.71687000	0.18948800	-4.59190800
H	-5.26137800	0.56385000	-3.75439200
H	-4.29721500	-0.84714900	-3.23761100
C	-7.68017900	-0.20207800	-0.22470200
C	-3.27527500	-0.87190000	2.70661700
N	-9.02243600	-0.31047100	-0.28355400
H	-7.20095900	0.06317600	-1.19103800
N	-2.40823300	-1.60233400	3.44839100
H	-3.17845500	0.22594500	2.83406500
C	-9.75077200	-0.08779300	-1.51832700
C	-9.79823600	-0.64606700	0.89991900
C	-1.45714700	-0.96480800	4.33803800
C	-2.43758700	-3.05541100	3.42344700

H	-10.31811200	-0.98918500	-1.80740300
H	-9.05139300	0.15954200	-2.32954100
H	-10.46564800	0.74552900	-1.40720900
H	-10.36447900	-1.57868100	0.73718400
H	-10.51515500	0.15983500	1.13076900
H	-9.11416200	-0.77777800	1.74678100
H	-1.63125900	-1.26191600	5.38649500
H	-1.55470300	0.12831700	4.26879600
H	-0.42185700	-1.24303500	4.07377900
H	-2.58494400	-3.45389300	4.44102800
H	-1.49186100	-3.45949100	3.02468000
H	-3.26748200	-3.38185100	2.78515200

IM8

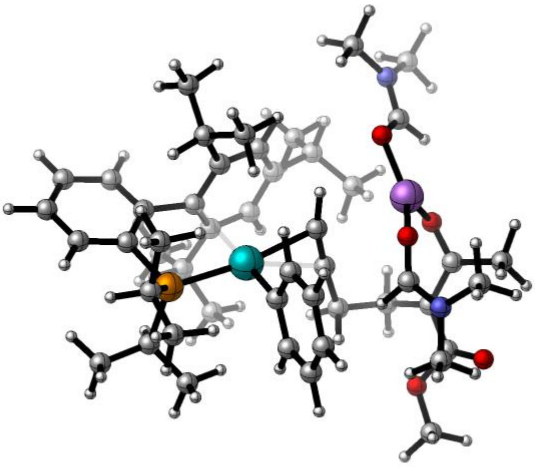

E (B3LYP-D3/Def2-SVP)) = -2832.66486516
E (SMD(DMF)/M06/Def2-TZVPP//B3LYP-D3/Def2-SVP) = -2833.54302314

Zero-point correction=	1.103273 (Hartree/Particle)
Thermal correction to Energy=	1.172399
Thermal correction to Enthalpy=	1.173344
Thermal correction to Gibbs Free Energy=	0.998748

Charge = 1 Multiplicity = 1

P	2.91899500	-1.98159000	-0.14365600
C	3.22372500	-3.13352500	-1.58529900
H	4.26961800	-3.45233800	-1.44814100
C	3.28796400	-2.93013500	1.44875600
H	3.55714800	-2.10654500	2.12942500
C	4.32935800	-0.79411600	-0.23561000
C	4.12333600	0.56620000	0.07757200
C	5.62648300	-1.23278100	-0.56672400
C	5.23420300	1.43062400	0.10103700
C	6.71218700	-0.35822000	-0.55609700
H	5.80265900	-2.27107400	-0.84912600
C	6.51710100	0.97989000	-0.20555000
H	5.07268500	2.48134100	0.35269900
H	7.70729800	-0.72346300	-0.81943400
H	7.36011200	1.67437400	-0.18372500
C	2.79259600	1.22348400	0.34730800
C	2.33421700	1.39085000	1.68763300

C	2.21970000	2.01699200	-0.69307100
C	1.35086500	2.35154400	1.95710000
C	1.26859200	2.98417200	-0.35612600
C	0.83663300	3.18738300	0.96021800
H	1.01546600	2.48549400	2.98751500
H	0.84629300	3.60804900	-1.14619300
C	-0.14670500	-2.20664300	-0.80278400
C	-0.45735100	-2.15954600	-2.16823500
C	-0.66738100	-3.23606700	-0.01126500
C	-1.23561200	-3.17003500	-2.74600600
H	-0.11310300	-1.33743300	-2.79758000
C	-1.44073300	-4.24815000	-0.59688800
H	-0.47627900	-3.27359300	1.05967700
C	-1.71528200	-4.22624300	-1.96631600
H	-1.46896900	-3.12179700	-3.81254100
H	-1.82895500	-5.05537000	0.02970300
H	-2.31412800	-5.01860900	-2.42085700
C	2.98161600	0.64020000	2.84520600
H	3.68416700	-0.08855000	2.41556600
C	-0.13186500	4.31044600	1.29207500
H	-0.38063300	4.80246500	0.33819100
C	2.65842400	1.87212400	-2.14739700
H	3.38794800	1.05035800	-2.19831300
C	3.81480800	1.59457800	3.71791000
H	3.17928800	2.35950100	4.19181400
H	4.32794800	1.03851800	4.51836700
H	4.57900600	2.11392000	3.11940100
C	1.95686200	-0.14101900	3.68009000
H	2.46185800	-0.73513400	4.45769400
H	1.24699600	0.53019800	4.18862700
H	1.37390400	-0.82872500	3.04888100
C	1.48230900	1.48773800	-3.06101600
H	1.82144500	1.38215300	-4.10315800
H	1.04164200	0.52791500	-2.75199600
H	0.67990300	2.24304500	-3.04545700
C	3.36529200	3.14253200	-2.64848700
H	4.22770800	3.39598100	-2.01343000
H	3.72974900	3.00194100	-3.67822600
H	2.68202800	4.00717200	-2.64991600
C	0.53641100	5.36368400	2.19319700
H	-0.14479700	6.21206100	2.36719000
H	0.79406600	4.94014900	3.17743500
H	1.46248700	5.75244200	1.74157500
C	-1.44302000	3.79751900	1.90494600
H	-1.96330700	3.08292600	1.25115700
H	-1.26042400	3.29864700	2.87061900
H	-2.12867000	4.63764300	2.10178900
C	4.49003400	-3.87818900	1.35158800
H	4.29786000	-4.72161300	0.67142500
H	5.40729700	-3.36845500	1.02800600
H	4.69208200	-4.30411400	2.34746900
C	2.06028900	-3.63216600	2.03887700
H	1.23764500	-2.92690300	2.21856800
H	1.68590700	-4.43684600	1.38999100

H	2.32968100	-4.08221500	3.00743700
C	3.12464500	-2.35536700	-2.90422300
H	3.80300700	-1.48914900	-2.92770200
H	2.10007500	-1.99732300	-3.07641100
C	2.35664900	-4.39953200	-1.59113100
H	1.29963000	-4.17819400	-1.77939100
H	2.70752500	-5.06290000	-2.39784500
H	2.42630000	-4.96389600	-0.65094600
H	3.39332500	-3.01500400	-3.74441300
Pd	0.95786800	-0.71208900	-0.04735100
C	-0.79671300	0.66647000	-0.25574700
C	-1.15687400	0.16141100	0.80915100
H	-0.75190000	1.30358800	-1.12383800
C	-1.68940800	-0.35194500	2.06060300
C	-3.01643200	0.35570800	2.48122600
H	-1.87945500	-1.43408000	1.97588400
H	-0.92728000	-0.21170900	2.84538300
C	-4.25228600	0.02278100	1.68713400
H	-2.85304200	1.43996400	2.45481100
H	-3.16967900	0.06516300	3.53077700
C	-4.69159100	0.90796600	0.68812800
C	-4.95608600	-1.21012900	1.96248500
O	-3.93461100	1.84336900	0.23571700
C	-6.06776800	0.78436400	0.05981700
O	-4.25558200	-2.00180000	2.85354000
O	-6.00276500	-1.62154200	1.47869700
Li	-3.17065900	1.45296300	-1.39137300
H	-6.10885900	-0.08396600	-0.61381600
H	-6.85134300	0.60961400	0.80832600
H	-6.27299500	1.70095300	-0.51155700
C	-4.87838300	-3.22483800	3.21981600
O	-2.19219600	2.98700100	-1.94841100
O	-3.41439200	-0.27527800	-1.99441200
H	-4.96510800	-3.90845500	2.35861900
H	-4.24197800	-3.67925000	3.99144900
H	-5.89181500	-3.05741100	3.61632500
C	-2.54168200	4.04026400	-1.39249300
C	-3.63789500	-1.33051600	-1.38039800
N	-2.01185400	5.24643600	-1.64401300
H	-3.33870700	4.03089800	-0.62259800
N	-4.63729100	-2.17623200	-1.64999000
H	-2.99747000	-1.64988300	-0.53773700
C	-2.46080600	6.43250300	-0.93115400
C	-0.95096400	5.41874300	-2.62472700
C	-4.87975100	-3.35373400	-0.83023200
C	-5.58528400	-1.91377100	-2.72181100
H	-2.84824000	7.18558100	-1.63651100
H	-3.25859200	6.16635600	-0.22445000
H	-1.62642000	6.88122300	-0.36622900
H	-1.25625700	6.14667400	-3.39359900
H	-0.03572200	5.79347000	-2.13603900
H	-0.74260600	4.45409700	-3.10188300
H	-4.86840500	-4.26143600	-1.45570600
H	-4.09584100	-3.43753600	-0.06786000

H	-5.84428600	-3.25789000	-0.31132800
H	-5.57656300	-2.74575100	-3.44539300
H	-6.60365600	-1.81879200	-2.31189900
H	-5.30731400	-0.98383700	-3.23102300

TS6

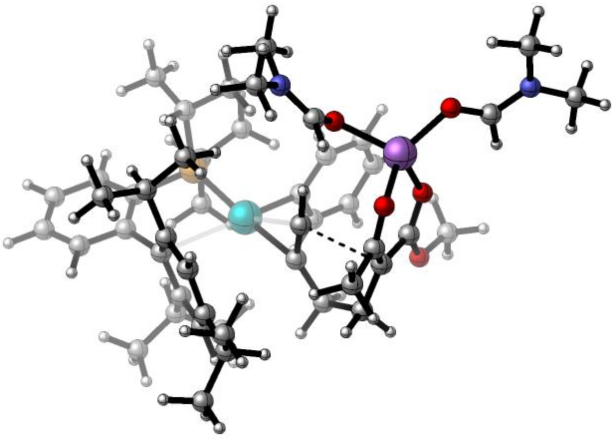

E (B3LYP-D3/Def2-SVP)) = -2832.66060168
E (SMD(DMF)/M06/Def2-TZVPP//B3LYP-D3/Def2-SVP) = -2833.53149102

Zero-point correction=	1.101219 (Hartree/Particle)
Thermal correction to Energy=	1.170343
Thermal correction to Enthalpy=	1.171288
Thermal correction to Gibbs Free Energy=	0.993523

Charge = 1 Multiplicity = 1

P	2.45389200	-2.14459300	0.46656800
C	2.82519000	-3.77250900	-0.37498700
H	3.58336800	-4.24816700	0.26803800
C	1.89767700	-2.53319300	2.23141100
H	2.16591800	-1.59864700	2.75041000
C	4.11978000	-1.40238100	0.74682400
C	4.26621200	-0.00032100	0.67594700
C	5.23506500	-2.19287500	1.08389600
C	5.51302200	0.56689800	0.99917900
C	6.46854300	-1.61479300	1.38262900
H	5.15236900	-3.28008500	1.10946800
C	6.60442900	-0.22475200	1.35469700
H	5.62072400	1.65308400	0.95292200
H	7.31940700	-2.24993900	1.63914300
H	7.56236800	0.24206200	1.59547200
C	3.20686000	0.97655600	0.23001100
C	2.47639900	1.72149300	1.19560200
C	3.21026000	1.41480300	-1.13054900
C	1.83867700	2.90653200	0.80839500
C	2.55811500	2.60859500	-1.45583900
C	1.89749200	3.39543800	-0.50119600
H	1.30488700	3.47364200	1.56966900
H	2.58946900	2.94872700	-2.49195000
C	-0.15976500	-1.87531100	-1.31247300
C	0.08803600	-2.32129300	-2.61780500
C	-1.27336300	-2.35740000	-0.61470200

C	-0.73063000	-3.30340100	-3.19156600
H	0.92292500	-1.92453900	-3.19905700
C	-2.07800700	-3.34916100	-1.18935900
H	-1.54045700	-1.96538700	0.36639200
C	-1.80005800	-3.83897500	-2.46900700
H	-0.51885800	-3.65380300	-4.20521300
H	-2.93817400	-3.72632700	-0.63006000
H	-2.42860000	-4.61565600	-2.91100000
C	2.42798000	1.29394300	2.65743000
H	2.95674600	0.33243400	2.73887400
C	1.27492400	4.72847700	-0.91127200
H	0.44946200	4.48242000	-1.60626200
C	3.99690900	0.66566800	-2.20364100
H	4.26613800	-0.31772100	-1.79030300
C	3.17123400	2.28879600	3.56366900
H	2.69756100	3.28327400	3.54115700
H	3.17121400	1.93864200	4.60839600
H	4.21804800	2.40778300	3.24507500
C	0.98325200	1.06765600	3.13182600
H	0.97298800	0.68206900	4.16391800
H	0.40121800	2.00283000	3.12005900
H	0.45994000	0.34445500	2.48848900
C	3.17145500	0.41006300	-3.47401100
H	3.73468200	-0.22887800	-4.17179500
H	2.21965600	-0.09012700	-3.23921000
H	2.93352200	1.34401000	-4.00721400
C	5.30655100	1.40249900	-2.53425200
H	5.93269800	1.52958700	-1.63880000
H	5.88989900	0.84054000	-3.28080800
H	5.09990100	2.40288000	-2.94773000
C	2.27859100	5.60833400	-1.68125400
H	1.80006100	6.54822700	-1.99709700
H	3.13905200	5.86331400	-1.04133200
H	2.66660400	5.11750300	-2.58549400
C	0.68365600	5.52537200	0.25682900
H	-0.08197700	4.96304500	0.81038300
H	1.47018000	5.82079500	0.97046400
H	0.20769400	6.44522500	-0.11451400
C	2.66642100	-3.68080800	2.89733700
H	2.45209100	-4.65093500	2.42416400
H	3.75331500	-3.52094700	2.88933600
H	2.35855200	-3.75982300	3.95267100
C	0.38021500	-2.71091800	2.35699200
H	-0.16400100	-1.82095100	2.01463200
H	0.00771900	-3.57054700	1.78217200
H	0.11925700	-2.87759300	3.41439100
C	3.45530700	-3.51157300	-1.75009100
H	4.36285300	-2.89300100	-1.67844100
H	2.74762000	-3.00793600	-2.42331500
C	1.62610400	-4.72779000	-0.45172800
H	0.81262700	-4.32927000	-1.06942900
H	1.95669600	-5.67610800	-0.90520600
H	1.21732400	-4.96650400	0.53993500
H	3.73803100	-4.46821600	-2.21740500

Pd	1.09419900	-0.50874600	-0.54503900
C	-0.26729400	0.97339400	-1.39330000
C	-0.91785900	0.92476200	-0.32562300
C	-0.52756200	1.44688700	-2.76972200
H	-1.41384700	0.71437100	0.60828600
C	-1.81993400	2.34667400	-2.74614500
H	0.33114400	1.98488900	-3.19776500
H	-0.69848500	0.55403700	-3.39262700
C	-2.84234500	1.88160600	-1.73885200
H	-2.22783100	2.33979000	-3.76830300
H	-1.50965600	3.37672100	-2.53061700
C	-3.20457400	2.64012400	-0.57876300
C	-3.47984500	0.60886100	-1.98580300
O	-3.90689000	2.20237600	0.37030900
C	-2.71358500	4.07638700	-0.45457900
O	-4.19945000	-0.04663500	-1.21683300
O	-3.21417100	0.12147100	-3.22224900
Li	-4.36287000	0.38979000	0.61737900
H	-3.14637000	4.51506200	0.45368900
H	-3.00393500	4.68665600	-1.32400300
H	-1.61798800	4.11186600	-0.38141400
C	-3.85462900	-1.09417200	-3.59588700
O	-6.08835300	-0.23698100	1.12770800
O	-2.94552700	-0.06883800	1.99816500
H	-3.41920500	-1.38469900	-4.56041500
H	-4.94100900	-0.94432400	-3.70688200
H	-3.67743500	-1.88023300	-2.85078500
C	-6.90096300	-0.59006500	0.27000600
C	-2.81806600	0.79620700	2.87355100
N	-8.18068800	-0.92614300	0.51551800
H	-6.60960600	-0.65419100	-0.79958400
N	-2.34809300	0.57270000	4.11329800
H	-3.09277000	1.85172800	2.66570900
C	-9.08035500	-1.32762800	-0.55114600
C	-8.72141200	-0.88657200	1.86537300
C	-2.20809300	1.65013200	5.07824000
C	-1.98417000	-0.76630800	4.54869900
H	-9.46396900	-2.34714000	-0.37589600
H	-8.55304900	-1.31320500	-1.51538100
H	-9.94263600	-0.64227800	-0.61358300
H	-9.08298000	-1.88499300	2.16355900
H	-9.56689100	-0.18047100	1.91918200
H	-7.93290900	-0.56281800	2.55499000
H	-2.82380400	1.45534000	5.97201200
H	-2.53134800	2.60020300	4.63058700
H	-1.15672100	1.75065000	5.39546900
H	-2.57746200	-1.05361600	5.43223800
H	-0.91582600	-0.80586600	4.81717000
H	-2.17952900	-1.47185700	3.73301600

TS7

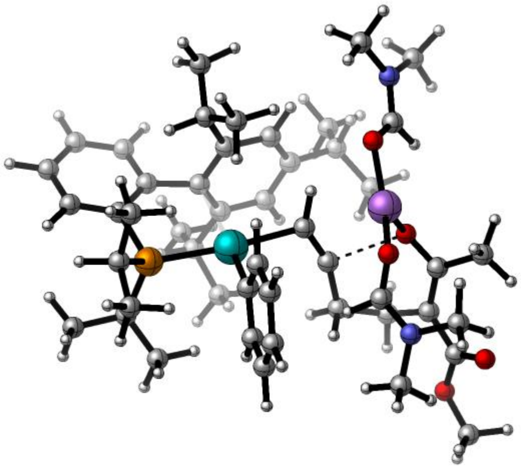

E (B3LYP-D3/Def2-SVP)) = -2832.65825513
E (SMD(DMF)/M06/Def2-TZVPP//B3LYP-D3/Def2-SVP) = -2833.52928297

Zero-point correction=	1.102634 (Hartree/Particle)
Thermal correction to Energy=	1.171026
Thermal correction to Enthalpy=	1.171970
Thermal correction to Gibbs Free Energy=	0.998979

Charge = 1 Multiplicity = 1

P	3.03329000	-2.01396400	-0.08933300
C	3.41405600	-3.03727800	-1.60836500
H	4.45502500	-3.36886000	-1.46166300
C	3.34455500	-3.10348900	1.42464100
H	3.59773700	-2.34822200	2.18642200
C	4.43559600	-0.81643300	-0.01054100
C	4.18235300	0.52002600	0.36458300
C	5.76004200	-1.21622800	-0.27318100
C	5.27228800	1.39705800	0.52158400
C	6.82617700	-0.32863900	-0.13211400
H	5.97222000	-2.23304000	-0.60537400
C	6.58195800	0.98269300	0.28359700
H	5.07418300	2.42879900	0.82145500
H	7.84427600	-0.66232800	-0.34484800
H	7.40865100	1.68589200	0.40865700
C	2.82140200	1.14308600	0.56499000
C	2.24207000	1.20762000	1.86723500
C	2.33648500	2.01736300	-0.45773500
C	1.24267900	2.15601600	2.12469300
C	1.35993000	2.96237000	-0.13175500
C	0.81601200	3.06760500	1.15483600
H	0.81875800	2.21178800	3.12899600
H	1.00521200	3.64592000	-0.90680700
C	-0.03994700	-2.15469800	-0.90164300
C	-0.31336900	-2.05935800	-2.27481200
C	-0.58880200	-3.22030100	-0.17472100
C	-1.07451100	-3.04269600	-2.91823000
H	0.05462900	-1.21527200	-2.86116700
C	-1.34435900	-4.20785600	-0.82340400
H	-0.42699700	-3.30533100	0.89905300

C	-1.58213400	-4.12866400	-2.19825300
H	-1.27520400	-2.95021000	-3.98854700
H	-1.74676000	-5.04282800	-0.24318700
H	-2.16834500	-4.89999700	-2.70297700
C	2.77067400	0.35723700	3.01571100
H	3.48541100	-0.36244900	2.59259900
C	-0.17156900	4.17299700	1.49014800
H	-0.42103900	4.66828600	0.53800700
C	2.88619000	1.96607400	-1.88018100
H	3.64466600	1.17053400	-1.91797100
C	3.55242900	1.21945700	4.02138600
H	2.90034200	1.97241200	4.49215900
H	3.97741000	0.59258900	4.82127100
H	4.38075300	1.75057800	3.52768900
C	1.65579700	-0.44550000	3.70326700
H	2.07764200	-1.12956200	4.45613400
H	0.93935700	0.21161900	4.22090600
H	1.09246600	-1.04470900	2.97088200
C	1.78991500	1.59205100	-2.89249200
H	2.20164800	1.55944300	-3.91335100
H	1.36958600	0.60121900	-2.66505700
H	0.95657400	2.31167900	-2.88575000
C	3.58455900	3.28015600	-2.26600100
H	4.38759500	3.52906000	-1.55559300
H	4.02999500	3.20067500	-3.27011600
H	2.87562500	4.12399200	-2.28122800
C	0.48925100	5.22688600	2.39691000
H	-0.20048200	6.06504900	2.58623700
H	0.76052500	4.79096900	3.37218100
H	1.40770100	5.63097100	1.94304700
C	-1.48042900	3.64814700	2.09800400
H	-1.97978300	2.92236300	1.44028200
H	-1.30260800	3.16009800	3.07034200
H	-2.17644300	4.48277400	2.28231400
C	4.53764700	-4.05844200	1.29142100
H	4.36435300	-4.83372100	0.53009400
H	5.47361300	-3.53656700	1.05205500
H	4.69383500	-4.57584400	2.25175100
C	2.08587500	-3.83509800	1.90295500
H	1.26982700	-3.13229600	2.11703900
H	1.72234500	-4.56645000	1.16680000
H	2.31039100	-4.38031200	2.83357500
C	3.35978700	-2.14815900	-2.85783500
H	4.03252600	-1.28056500	-2.77964800
H	2.33981900	-1.77898800	-3.03560500
C	2.54179200	-4.29155000	-1.75486400
H	1.48835500	-4.04361700	-1.93305300
H	2.89889700	-4.87410600	-2.61930800
H	2.59443900	-4.94768700	-0.87492100
H	3.66401800	-2.73041900	-3.74208900
Pd	1.07524700	-0.72058800	-0.04557100
C	-0.63973100	0.52947100	-0.20348200
C	-1.49225700	0.18295500	0.65427200
H	-0.55156400	1.27621400	-0.98563100

C	-2.08692300	-0.54385200	1.76673900
C	-3.33113700	0.11971800	2.42116800
H	-2.35047600	-1.56032700	1.42896000
H	-1.29264400	-0.64709600	2.52651800
C	-4.54196700	0.17656700	1.52663300
H	-3.03652200	1.13461900	2.73097000
H	-3.55795900	-0.45221000	3.32765300
C	-4.46083300	1.01168100	0.42030900
C	-5.68965900	-0.69049200	1.76417600
O	-3.31384800	1.53401500	0.09240900
C	-5.62567500	1.41184500	-0.45510600
O	-5.62589400	-1.32073400	2.97144600
O	-6.61679500	-0.92017100	0.99690100
Li	-2.78270400	1.17691000	-1.70233100
H	-5.51611700	0.96578600	-1.45597700
H	-6.59516100	1.10948100	-0.04854600
H	-5.58793800	2.50670600	-0.57800400
C	-6.69247500	-2.21257700	3.27576200
O	-1.84003200	2.71550800	-2.29226200
O	-3.50586600	-0.43832800	-2.23697400
H	-6.70605000	-3.07112100	2.58393400
H	-6.51692000	-2.56372200	4.30089600
H	-7.66747400	-1.70651700	3.20720400
C	-2.01948300	3.79280900	-1.70393400
C	-3.69450600	-1.45726600	-1.55153800
N	-1.46452500	4.95839100	-2.06531400
H	-2.67048100	3.84460300	-0.80827700
N	-4.83121900	-2.15445200	-1.50205000
H	-2.89180300	-1.86624800	-0.91093300
C	-1.73236000	6.18833000	-1.33509600
C	-0.57732800	5.04709200	-3.21707800
C	-4.98390900	-3.28993300	-0.60570000
C	-6.02150700	-1.73932900	-2.23371300
H	-2.21799100	6.92927000	-1.99107500
H	-2.39390100	5.98387300	-0.48234000
H	-0.79343500	6.62251600	-0.95457700
H	-0.93012800	5.83115700	-3.90564400
H	0.44643700	5.30164600	-2.89446600
H	-0.56562700	4.08318300	-3.73864900
H	-5.22428300	-4.20182800	-1.17707300
H	-4.04975500	-3.45832700	-0.05356400
H	-5.79571200	-3.08376000	0.10812100
H	-6.44146900	-2.60066700	-2.77695300
H	-6.76727000	-1.35546800	-1.52052300
H	-5.74934300	-0.95602700	-2.95056200

IM9

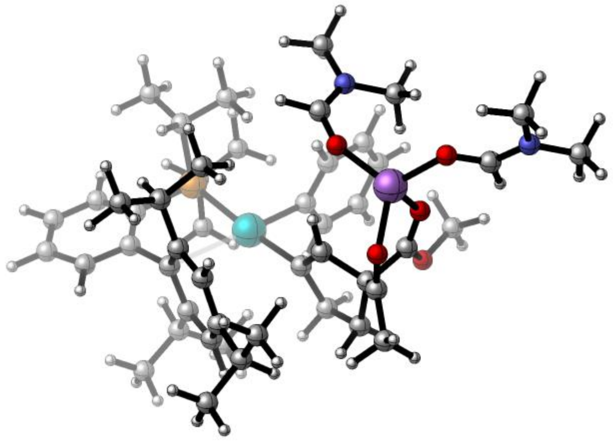

E (B3LYP-D3/Def2-SVP)) = -2832.71595338
E (SMD(DMF)/M06/Def2-TZVPP//B3LYP-D3/Def2-SVP) = -2833.58387166

Zero-point correction=	1.105073 (Hartree/Particle)
Thermal correction to Energy=	1.173485
Thermal correction to Enthalpy=	1.174430
Thermal correction to Gibbs Free Energy=	1.000088

Charge = 1 Multiplicity = 1

P	3.04912800	-1.79731400	0.21261800
C	3.79822300	-3.00357900	-1.00764700
H	4.69935900	-3.41366800	-0.52260000
C	2.70157400	-2.77183100	1.79652200
H	2.73529500	-1.97367500	2.55650700
C	4.47805300	-0.75190900	0.72704300
C	4.24437700	0.62335100	0.93651200
C	5.76361600	-1.27373500	0.96165700
C	5.28871300	1.41973300	1.44208000
C	6.79588500	-0.46589400	1.43885000
H	5.97314000	-2.32578800	0.76234400
C	6.55116400	0.88528600	1.69844100
H	5.10081300	2.48178500	1.61687200
H	7.78700500	-0.89197400	1.61105100
H	7.34716900	1.52642400	2.08460700
C	2.96770500	1.35857800	0.60260800
C	2.02133100	1.64692100	1.62521300
C	2.92111600	2.10129300	-0.61909900
C	1.12187500	2.70463900	1.44941200
C	2.00358700	3.15134400	-0.73572300
C	1.12198700	3.49563200	0.29753200
H	0.41679300	2.91850700	2.25171500
H	1.99025400	3.73737000	-1.65834200
C	0.21779100	-1.71844000	-1.48761700
C	0.44466000	-1.97512000	-2.85194800
C	-0.74681400	-2.49994600	-0.82920700
C	-0.21697300	-3.01673400	-3.51555200
H	1.15687000	-1.36653000	-3.41419800
C	-1.39675200	-3.55343800	-1.48453400
H	-0.99739400	-2.29213600	0.21078700
C	-1.12806800	-3.82597800	-2.83040100

H	-0.00642500	-3.20188800	-4.57261700
H	-2.12400500	-4.16319500	-0.94018600
H	-1.63026000	-4.65136600	-3.34103600
C	2.00673300	0.86866200	2.93457100
H	2.77317200	0.08355000	2.85907100
C	0.23229700	4.72352600	0.14775000
H	-0.24311100	4.64530400	-0.84759000
C	3.93341800	1.84415600	-1.73337900
H	4.49686300	0.93964800	-1.46305300
C	2.39031100	1.75294600	4.13141300
H	1.66275100	2.56696900	4.27912200
H	2.42243000	1.15905500	5.05900000
H	3.38152500	2.20892000	3.98424000
C	0.65225600	0.17300400	3.14899900
H	0.68673100	-0.47915200	4.03736900
H	-0.16353400	0.89857300	3.29557000
H	0.39097100	-0.43777600	2.27141100
C	3.26838200	1.56393100	-3.08874300
H	4.03143800	1.30993000	-3.84124000
H	2.56279700	0.72265800	-3.01438300
H	2.71575200	2.43980600	-3.46469700
C	4.94363900	2.99968900	-1.83560800
H	5.46347700	3.16227300	-0.87953200
H	5.70334000	2.78336400	-2.60357400
H	4.44299100	3.94168900	-2.11229000
C	1.08484000	6.00735400	0.14557400
H	0.45490200	6.89550500	-0.02299600
H	1.59523500	6.13031500	1.11466400
H	1.85679600	5.98420200	-0.63824200
C	-0.87787300	4.82431100	1.19861800
H	-1.49255600	3.91284100	1.24495700
H	-0.45987400	4.99680100	2.20327900
H	-1.54068800	5.67498000	0.97758300
C	3.75560000	-3.82053300	2.17012700
H	3.79647500	-4.64468600	1.44193500
H	4.76154100	-3.38950700	2.26192700
H	3.50366100	-4.26343300	3.14792100
C	1.28465000	-3.35664400	1.81440100
H	0.52934300	-2.57076100	1.69319500
H	1.12168400	-4.09458500	1.01557400
H	1.10577600	-3.85981200	2.77915200
C	4.22947100	-2.23520800	-2.26450400
H	4.94856900	-1.43506000	-2.03112200
H	3.36297500	-1.77830400	-2.76497100
C	2.86592500	-4.17664200	-1.34198200
H	1.91214100	-3.83743000	-1.76690900
H	3.35205900	-4.82611700	-2.08815600
H	2.64741800	-4.79931600	-0.46297400
H	4.71106600	-2.92101000	-2.97998500
Pd	1.30251500	-0.31944400	-0.52638000
C	-0.26799500	0.81300600	-1.14939100
C	-1.36819400	1.05075000	-0.41483200
C	-0.44145800	1.37104500	-2.54854600
H	-1.58111500	0.70912800	0.59813500

C	-1.70115200	2.26157300	-2.46153000
H	0.43905300	1.93424100	-2.88830400
H	-0.58329200	0.54047700	-3.25922000
C	-2.49623800	1.70411800	-1.26143500
H	-2.28603600	2.29207500	-3.39057600
H	-1.39075200	3.28746800	-2.22334800
C	-3.35007200	2.68517700	-0.45572800
C	-3.36638400	0.48966500	-1.60808900
O	-3.87416600	2.31906100	0.58767200
C	-3.56503600	4.08167000	-0.97023400
O	-4.11273400	-0.06707100	-0.81204800
O	-3.17182000	0.04356800	-2.83684400
Li	-4.27778600	0.48001500	1.13055300
H	-4.26588200	4.61293200	-0.31444900
H	-3.94893600	4.04697700	-2.00263600
H	-2.61220700	4.62882100	-1.00931900
C	-3.75968700	-1.21659600	-3.19708300
O	-6.09809500	0.05303600	1.43449700
O	-3.02372700	-0.41291100	2.21082800
H	-3.52008100	-1.36442000	-4.25589700
H	-4.84815900	-1.19346100	-3.04320300
H	-3.30990900	-2.01949200	-2.59705800
C	-6.98973200	-0.12254900	0.59907000
C	-2.49538000	-1.47078200	2.55877000
N	-8.29775900	-0.22544700	0.88171900
H	-6.75109000	-0.21249600	-0.48216900
N	-2.84110900	-2.68378500	2.10194500
H	-1.66712700	-1.48138200	3.29582900
C	-9.29124000	-0.43222900	-0.15878700
C	-8.77985200	-0.12152800	2.25251700
C	-2.14765500	-3.89124400	2.52357900
C	-3.92524800	-2.79388200	1.12765400
H	-9.84602000	-1.36952100	0.01428300
H	-8.80421000	-0.48975700	-1.14240700
H	-10.01603400	0.39877200	-0.17394500
H	-9.30940500	-1.04450800	2.54093100
H	-9.47924500	0.72537600	2.34666400
H	-7.92584900	0.03519900	2.92179400
H	-2.85313800	-4.60930700	2.97159500
H	-1.38272600	-3.64054400	3.27024000
H	-1.64656300	-4.37192000	1.66771000
H	-4.82223900	-2.27472000	1.49692800
H	-4.15864600	-3.85447000	0.97117200
H	-3.64019900	-2.33705000	0.16827400

IM10

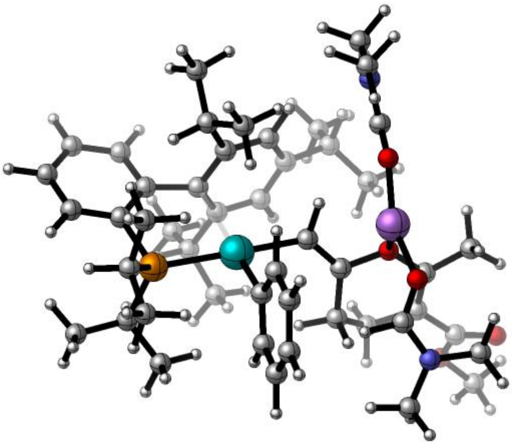

E (B3LYP-D3/Def2-SVP)) = -2832.69417396
E (SMD(DMF)/M06/Def2-TZVPP//B3LYP-D3/Def2-SVP) = -2833.56099491

Zero-point correction=	1.104765 (Hartree/Particle)
Thermal correction to Energy=	1.173621
Thermal correction to Enthalpy=	1.174565
Thermal correction to Gibbs Free Energy=	0.998389

Charge = 1 Multiplicity = 1

P	3.16257400	-1.79140500	0.15175100
C	3.86190400	-2.65830600	-1.35402200
H	4.89693300	-2.93156500	-1.09030000
C	3.35380600	-2.97323900	1.61686400
H	3.43797500	-2.26603600	2.45920400
C	4.43159400	-0.50480200	0.52952700
C	3.99544300	0.77949700	0.91885200
C	5.81235600	-0.77390500	0.48267800
C	4.95568100	1.73270000	1.30828800
C	6.75230000	0.18806100	0.85195200
H	6.16890400	-1.74697600	0.14216500
C	6.31984300	1.44453500	1.28390700
H	4.61437100	2.72254500	1.62070000
H	7.81860800	-0.04476200	0.80483600
H	7.04403200	2.20415300	1.58769500
C	2.56826300	1.27599300	0.90118300
C	1.77172000	1.23496900	2.08164400
C	2.18284800	2.14156700	-0.16733900
C	0.67344100	2.10008300	2.19547500
C	1.09978100	3.00543300	0.01761500
C	0.35143400	3.02764700	1.20087700
H	0.08451200	2.07887200	3.11453800
H	0.84006200	3.70140600	-0.78316600
C	0.16265000	-2.08713000	-1.16427000
C	0.07361600	-1.91648300	-2.55893000
C	-0.32424800	-3.28791900	-0.62032000
C	-0.41348900	-2.93720900	-3.38534800
H	0.40026800	-0.98147300	-3.02059700
C	-0.80452700	-4.31400200	-1.44612800
H	-0.31112400	-3.44982400	0.45666600
C	-0.84120700	-4.15035900	-2.83488500

H	-0.45365500	-2.78216300	-4.46697300
H	-1.14277300	-5.25223700	-0.99612000
H	-1.20621900	-4.95341100	-3.47949500
C	2.16520500	0.36906600	3.27249100
H	2.98874800	-0.28286200	2.94808100
C	-0.75123700	4.05525100	1.41152200
H	-0.77944600	4.68611000	0.50418300
C	2.96536900	2.18415500	-1.47597800
H	3.73173900	1.39741100	-1.43068600
C	2.70678100	1.23338900	4.42369500
H	1.93320300	1.91974900	4.80404500
H	3.03875900	0.60011100	5.26177200
H	3.56438500	1.83984400	4.09398000
C	1.01560700	-0.53811900	3.73581900
H	1.35580200	-1.21638400	4.53402400
H	0.17000500	0.04406100	4.13522700
H	0.63692900	-1.14695000	2.90073700
C	2.06414900	1.86566500	-2.67931100
H	2.65513200	1.83178900	-3.60825700
H	1.56676000	0.89334400	-2.54975200
H	1.27914200	2.62655500	-2.80731800
C	3.69390900	3.52507400	-1.66049900
H	4.37562800	3.72780900	-0.82095700
H	4.28831400	3.52049000	-2.58807500
H	2.97980600	4.36328300	-1.72193000
C	-0.42888100	4.98125100	2.59668500
H	-1.19053600	5.77153800	2.69192400
H	-0.41235900	4.41979700	3.54469400
H	0.55400000	5.46199200	2.47461600
C	-2.13469400	3.40656600	1.56798600
H	-2.39390800	2.79978700	0.69072600
H	-2.16500400	2.73864400	2.44400100
H	-2.91213600	4.17448900	1.70878300
C	4.61642800	-3.84289300	1.60330000
H	4.61290800	-4.56279600	0.77092500
H	5.53804200	-3.24861300	1.54379300
H	4.66863500	-4.42659900	2.53688600
C	2.09169400	-3.81295400	1.84831300
H	1.20449500	-3.17665800	1.97000000
H	1.89783200	-4.50871700	1.01876700
H	2.20710600	-4.41116100	2.76662700
C	3.89992100	-1.67362700	-2.53078700
H	4.49794600	-0.77832100	-2.30186100
H	2.88427700	-1.34991900	-2.80403600
C	3.10931200	-3.94504400	-1.71994800
H	2.07217700	-3.74295000	-2.01662800
H	3.61322700	-4.42748600	-2.57318900
H	3.09185200	-4.67311100	-0.89676500
H	4.34756700	-2.15948400	-3.41269700
Pd	1.05911200	-0.67344500	-0.04845800
C	-0.72192800	0.27063600	-0.29093600
C	-1.91182400	-0.02888700	0.23679400
H	-0.69837300	1.11474100	-0.99282300
C	-2.27997000	-1.11511600	1.19753000

C	-3.29196100	-0.61853400	2.23260500
H	-2.72086200	-1.96288000	0.64363800
H	-1.36289700	-1.48471300	1.67117200
C	-4.46701400	0.02596700	1.52949600
H	-2.81178200	0.10586500	2.91477400
H	-3.63730600	-1.44890400	2.86285000
C	-4.26633600	0.68860700	0.36121300
C	-5.81951400	-0.08013600	2.12352400
O	-3.02794400	0.74283800	-0.22382500
C	-5.23688800	1.49003500	-0.44671600
O	-5.75209200	-0.36574800	3.43799600
O	-6.88050700	0.03123700	1.53826400
Li	-2.71239300	0.64727600	-2.22559000
H	-5.33575600	1.04875100	-1.45146100
H	-6.22559700	1.52412800	0.01776300
H	-4.83936200	2.51129500	-0.56881100
C	-7.00000700	-0.52841500	4.11810900
O	-2.00856500	2.18115200	-2.95159300
O	-3.71961200	-0.84316500	-2.55389900
H	-7.58874100	-1.34069600	3.66493500
H	-6.75094200	-0.76874000	5.15878100
H	-7.59359800	0.39695800	4.06894100
C	-1.57086100	3.28107000	-2.59294000
C	-3.71465000	-1.95818800	-2.00775800
N	-0.87582200	4.11865000	-3.37223000
H	-1.72541900	3.65223800	-1.55953900
N	-4.78559900	-2.53520200	-1.45210300
H	-2.78725900	-2.55623000	-1.93855400
C	-0.37178400	5.38737000	-2.86912600
C	-0.56940500	3.78388800	-4.75763300
C	-4.67090200	-3.79988900	-0.74256600
C	-6.08945000	-1.87916300	-1.45105500
H	-0.74820400	6.22197500	-3.48197800
H	-0.69605200	5.53960200	-1.83070100
H	0.73040000	5.40089000	-2.89889700
H	-1.00050200	4.53996100	-5.43319800
H	0.52220800	3.75959300	-4.90564000
H	-0.99086900	2.79993700	-4.99273500
H	-5.35275400	-4.55056600	-1.17379400
H	-3.64016900	-4.17389800	-0.80995200
H	-4.93077800	-3.66462800	0.32078600
H	-6.87141900	-2.62260700	-1.66677700
H	-6.30052700	-1.41048400	-0.47516700
H	-6.10288400	-1.10675900	-2.22873900

TS8

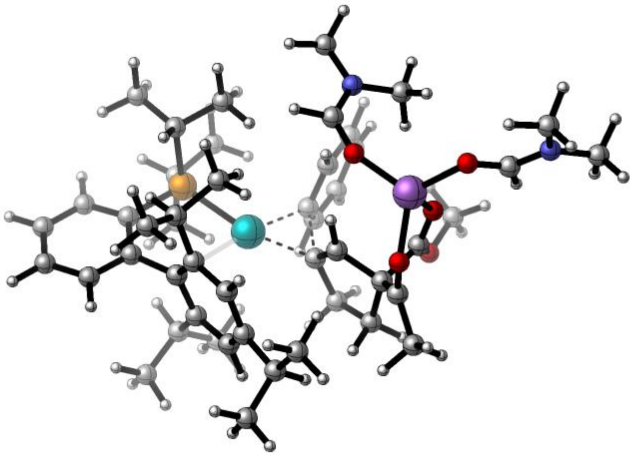

E (B3LYP-D3/Def2-SVP)) = -2832.69721533
E (SMD(DMF)/M06/Def2-TZVPP//B3LYP-D3/Def2-SVP) = -2833.57389940

Zero-point correction=	1.103316 (Hartree/Particle)
Thermal correction to Energy=	1.171566
Thermal correction to Enthalpy=	1.172510
Thermal correction to Gibbs Free Energy=	0.997776

Charge = 1 Multiplicity = 1

P	-3.06994300	-1.75226700	-0.25015800
C	-3.84782600	-2.93841100	0.97352200
H	-4.71762500	-3.40493500	0.48183800
C	-2.65558900	-2.76443400	-1.79344000
H	-2.64372400	-1.98253300	-2.57126700
C	-4.48326900	-0.71797600	-0.82595800
C	-4.24957500	0.66209900	-1.01669500
C	-5.75970200	-1.24211200	-1.09975500
C	-5.28579600	1.45908800	-1.53727100
C	-6.78393900	-0.43383800	-1.59398700
H	-5.96720300	-2.29790400	-0.91714900
C	-6.53961400	0.92174300	-1.82951000
H	-5.09836900	2.52325400	-1.69948100
H	-7.76817300	-0.86231200	-1.79752400
H	-7.32903600	1.56403900	-2.22726800
C	-2.97479600	1.39127100	-0.66307800
C	-2.01492200	1.67469900	-1.67082500
C	-2.90081000	2.08401000	0.58348300
C	-1.07887500	2.69368700	-1.46090800
C	-1.93921400	3.09224000	0.73985100
C	-1.05193100	3.44822900	-0.28342500
H	-0.37024600	2.91265300	-2.25925400
H	-1.91111300	3.64840700	1.68068300
C	0.04735600	-1.42728000	1.64933900
C	-0.30289800	-1.81404900	2.96389500
C	0.92742200	-2.28295800	0.94265200
C	0.17178400	-3.00383200	3.52612200
H	-0.96845100	-1.17941500	3.55306700
C	1.38335000	-3.47710700	1.49457700
H	1.26286600	-1.99104500	-0.05219200

C	1.01036200	-3.84991900	2.79571100
H	-0.13058900	-3.27597000	4.54096100
H	2.04806600	-4.12237000	0.91307600
H	1.37403500	-4.78326700	3.23152500
C	-1.99670700	0.90890700	-2.98782600
H	-2.80031500	0.15877500	-2.94559400
C	-0.15624500	4.67021900	-0.11252700
H	0.33811600	4.56717300	0.87139700
C	-3.93849900	1.84772700	1.67978500
H	-4.50934800	0.94964400	1.40397200
C	-2.29546700	1.81583300	-4.19148800
H	-1.52607300	2.59570900	-4.30970800
H	-2.32318900	1.22922000	-5.12394500
H	-3.26859700	2.31755200	-4.07548300
C	-0.66847200	0.15168000	-3.15915800
H	-0.69720300	-0.48675800	-4.05796800
H	0.18581000	0.83983500	-3.26086400
H	-0.47339200	-0.48342700	-2.28166100
C	-3.30984400	1.57003800	3.05243300
H	-4.09307900	1.33927600	3.79151300
H	-2.62172400	0.71234100	3.00209700
H	-2.74698500	2.43797000	3.43231800
C	-4.93217800	3.02024800	1.75080500
H	-5.42910500	3.18174600	0.78259300
H	-5.71153100	2.82485100	2.50473500
H	-4.42014900	3.95624300	2.02762700
C	-1.00750800	5.95405900	-0.05585800
H	-0.37312700	6.83779200	0.12020100
H	-1.54108800	6.10160400	-1.00886800
H	-1.76058800	5.91074300	0.74509000
C	0.93087700	4.80721000	-1.18344900
H	1.54470400	3.89967100	-1.27986300
H	0.48811700	5.01506200	-2.17057400
H	1.59947200	5.64945400	-0.94792700
C	-3.69058600	-3.81997600	-2.19739300
H	-3.77664400	-4.62087300	-1.44678400
H	-4.68773100	-3.38720000	-2.35605800
H	-3.38906700	-4.29502000	-3.14596200
C	-1.24124100	-3.35565000	-1.72065300
H	-0.48957000	-2.58123000	-1.51910300
H	-1.14414000	-4.11437200	-0.93057600
H	-0.99439800	-3.83981600	-2.68029300
C	-4.33828100	-2.14258900	2.19082800
H	-5.07189900	-1.37097900	1.91144900
H	-3.49693300	-1.64445800	2.69796200
C	-2.86705100	-4.04511400	1.38511300
H	-1.92507000	-3.62914800	1.77124100
H	-3.31346200	-4.65873400	2.18460500
H	-2.62686500	-4.72170000	0.55258500
H	-4.82086200	-2.81759900	2.91612900
Pd	-1.35475400	-0.34306300	0.58500600
C	0.37227700	0.42004800	1.43441500
C	1.36403900	0.80931300	0.58765700
C	0.51166800	1.16153300	2.76148800

H	1.56824900	0.40523700	-0.40276800
C	1.65272300	2.17627900	2.52842400
H	-0.42643300	1.65799200	3.04633700
H	0.77045000	0.46406000	3.57013700
C	2.44235800	1.62181100	1.32603100
H	2.27743300	2.33607000	3.41782200
H	1.22369200	3.14314100	2.23436800
C	3.22093000	2.61833200	0.46635600
C	3.40819700	0.47335800	1.67599600
O	3.67219900	2.26405100	-0.61328700
C	3.48812700	3.99682700	1.00132400
O	4.14743900	-0.06217800	0.85778200
O	3.27415000	0.04385900	2.91867800
Li	4.18361200	0.44110000	-1.12849700
H	4.14360300	4.54289200	0.31197200
H	3.95074200	3.92251400	1.99917500
H	2.54833500	4.55097200	1.13467400
C	3.88344900	-1.20923900	3.27645000
O	6.00284800	0.09715700	-1.50423300
O	2.92817100	-0.51024100	-2.14803500
H	3.76035900	-1.29998100	4.36143700
H	4.94790800	-1.21513200	3.00318600
H	3.35707600	-2.03071500	2.76859700
C	6.94579900	-0.03556200	-0.71822700
C	2.43712300	-1.58374600	-2.50577900
N	8.23883800	-0.08997200	-1.07234400
H	6.77092400	-0.12401500	0.37521300
N	2.84488300	-2.78558700	-2.07335300
H	1.59173500	-1.61611100	-3.22196400
C	9.29580700	-0.24848700	-0.08708600
C	8.64034200	0.01963300	-2.46861300
C	2.19500500	-4.01663300	-2.49785200
C	3.96026000	-2.85776200	-1.13069200
H	9.87636400	-1.16510000	-0.28434200
H	8.86661000	-0.31580700	0.92249100
H	9.98707600	0.61010800	-0.11836400
H	9.18779300	-0.88541900	-2.77943000
H	9.30100100	0.89116200	-2.60688900
H	7.74560000	0.13843200	-3.09078400
H	2.92341700	-4.70192000	-2.95980700
H	1.41299300	-3.78951900	-3.23407000
H	1.72209600	-4.52318200	-1.64096600
H	4.85077200	-2.36765500	-1.55308700
H	4.18812900	-3.91115600	-0.92632600
H	3.71154200	-2.35020000	-0.18652800

TS9

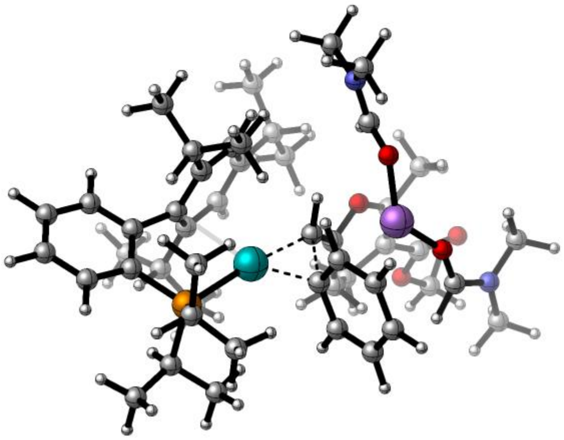

E (B3LYP-D3/Def2-SVP)) = -2832.67927327
E (SMD(DMF)/M06/Def2-TZVPP//B3LYP-D3/Def2-SVP) = -2833.55024330

Zero-point correction=	1.103771 (Hartree/Particle)
Thermal correction to Energy=	1.172100
Thermal correction to Enthalpy=	1.173044
Thermal correction to Gibbs Free Energy=	0.997752

Charge = 1 Multiplicity = 1

P	-3.41026700	-1.65223600	-0.19594300
C	-4.15557400	-2.48017400	1.30875800
H	-5.17078500	-2.81804700	1.04116900
C	-3.58308200	-2.85785300	-1.64027900
H	-3.59415500	-2.16991700	-2.50354300
C	-4.60043500	-0.30382700	-0.61166400
C	-4.08837800	0.97440600	-0.93767900
C	-5.99235000	-0.50808400	-0.62385300
C	-4.99017300	1.98602900	-1.31699600
C	-6.87317800	0.51141900	-0.98594400
H	-6.40255900	-1.47735000	-0.33510600
C	-6.36688500	1.76226300	-1.34811100
H	-4.59380000	2.96885300	-1.58326600
H	-7.95029500	0.32853000	-0.98711700
H	-7.04366300	2.56745600	-1.64396600
C	-2.63342600	1.38140100	-0.88002800
C	-1.82651200	1.33993200	-2.04779900
C	-2.16199600	2.10131900	0.26088000
C	-0.61217400	2.04748700	-2.07533100
C	-0.95213300	2.79348500	0.17311000
C	-0.16427500	2.79892800	-0.98689500
H	-0.01666400	2.01095100	-2.98751700
H	-0.60519900	3.35311700	1.04495300
C	0.03530300	-1.83242900	1.19588900
C	-0.00672100	-1.73621400	2.61601000
C	0.53164300	-3.05256900	0.65915400
C	0.42325200	-2.78561900	3.43847200
H	-0.40172600	-0.83003800	3.08170600
C	0.94998800	-4.09498400	1.48623400
H	0.54984400	-3.19998800	-0.42034100
C	0.91139300	-3.97255400	2.88215800

H	0.36021200	-2.67372000	4.52462100
H	1.29917400	-5.02741700	1.03290900
H	1.24126800	-4.79281300	3.52292700
C	-2.28321100	0.59540200	-3.29724300
H	-3.21047700	0.06070500	-3.04334100
C	1.12032200	3.62140700	-1.00293500
H	1.68865400	3.31316900	-0.10632800
C	-2.98259000	2.18388400	1.54450200
H	-3.79364200	1.44588500	1.46806200
C	-2.62556300	1.57267100	-4.43389800
H	-1.73929700	2.14794000	-4.74717500
H	-3.00314600	1.02905700	-5.31465300
H	-3.39877100	2.29032000	-4.11882800
C	-1.25804000	-0.45843300	-3.74444600
H	-1.64037000	-1.03286300	-4.60305100
H	-0.30550600	0.00219100	-4.05236600
H	-1.04311100	-1.16349400	-2.92656900
C	-2.15773200	1.81542100	2.78659700
H	-2.79369000	1.80863200	3.68566700
H	-1.70923300	0.81781100	2.67480800
H	-1.33988800	2.53223100	2.96281900
C	-3.62920500	3.57067300	1.69898000
H	-4.26852000	3.81106300	0.83669000
H	-4.25241100	3.61310300	2.60670600
H	-2.86046300	4.35749900	1.77653000
C	0.81126600	5.12471100	-0.87227800
H	1.74150600	5.71244600	-0.80880600
H	0.24569100	5.47967600	-1.74883000
H	0.20653500	5.34585900	0.02066700
C	2.02312300	3.36503800	-2.21145300
H	2.27026200	2.30020500	-2.31643200
H	1.54873600	3.70089900	-3.14755000
H	2.96892500	3.91879700	-2.10712300
C	-4.87193900	-3.68623900	-1.66877100
H	-4.93109400	-4.37956900	-0.81516200
H	-5.77470800	-3.05993300	-1.66860600
H	-4.90151000	-4.29763900	-2.58568600
C	-2.33680000	-3.74619500	-1.77339000
H	-1.41678200	-3.14660400	-1.82219900
H	-2.23203700	-4.44178600	-0.92735900
H	-2.40317300	-4.34876100	-2.69398100
C	-4.25759900	-1.46077200	2.45109700
H	-4.89942500	-0.60644500	2.18807600
H	-3.26194500	-1.07252900	2.71859100
C	-3.31968100	-3.69838100	1.72659200
H	-2.27475300	-3.41597100	1.92883000
H	-3.73539800	-4.13728100	2.64812200
H	-3.31662300	-4.48760000	0.96118900
H	-4.68558300	-1.93927900	3.34684000
Pd	-1.27647600	-0.71256000	0.07385800
C	0.62603900	-0.13650900	0.49901200
C	1.70569200	-0.09199200	-0.31500400
H	0.61623600	0.60629400	1.30040600
C	2.04147600	-1.04665200	-1.41728300

C	3.06446300	-0.48345200	-2.40419400
H	2.45333500	-1.96502300	-0.95749400
H	1.11654400	-1.34451600	-1.92756800
C	4.20864400	0.16406200	-1.65446600
H	2.58238900	0.25569000	-3.06892500
H	3.43602500	-1.28047800	-3.06052900
C	3.92992400	0.84719800	-0.51231100
C	5.58323100	0.07302500	-2.18370700
O	2.67363800	0.86202200	0.01134200
C	4.82014200	1.72647700	0.30412900
O	5.58322600	-0.22801100	-3.49622900
O	6.61818800	0.20533100	-1.55251500
Li	2.20268600	-0.70466800	2.48103500
H	4.88669800	1.33361800	1.33052600
H	5.82421300	1.80686900	-0.12053200
H	4.35904800	2.72648400	0.36412600
C	6.86155100	-0.37148600	-4.12089400
O	1.96694800	0.93113000	3.34238300
O	3.75810600	-1.44383700	1.91091500
H	7.42963400	-1.19781700	-3.66571100
H	6.66175900	-0.58420800	-5.17798900
H	7.45189400	0.55129300	-4.01836900
C	1.87195000	2.10872300	2.97144500
C	4.35128200	-2.20708700	1.13497700
N	1.35007400	3.10335600	3.70006300
H	2.21459600	2.41494400	1.96363300
N	5.65614700	-2.16133800	0.86279200
H	3.80316100	-3.00899400	0.60002100
C	1.24039100	4.45371300	3.16941600
C	0.84490600	2.87263400	5.04726500
C	6.25931600	-3.02592100	-0.14067300
C	6.53653400	-1.18047600	1.48954300
H	1.79845000	5.16561700	3.79892900
H	1.64066500	4.49334200	2.14727700
H	0.18389500	4.76815800	3.14147200
H	1.37665100	3.51696600	5.76565600
H	-0.23095700	3.10687700	5.09340500
H	1.00005800	1.82113000	5.31480900
H	7.04592300	-3.65189800	0.31103800
H	5.49786700	-3.68128600	-0.58614800
H	6.71251100	-2.40821400	-0.93218000
H	7.40467500	-1.69597500	1.92968700
H	6.88358700	-0.46473200	0.72932300
H	5.98708600	-0.65624000	2.27940200

Compound 2

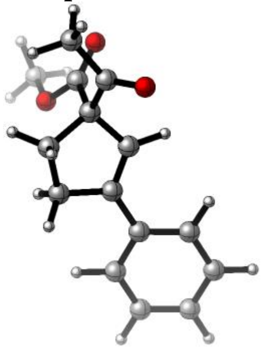

E (B3LYP-D3/Def2-SVP)) = -806.347222514
E (SMD(DMF)/M06/Def2-TZVPP//B3LYP-D3/Def2-SVP) = -806.672786189

Zero-point correction=	0.277565 (Hartree/Particle)
Thermal correction to Energy=	0.294649
Thermal correction to Enthalpy=	0.295593
Thermal correction to Gibbs Free Energy=	0.231113

Charge = 0 Multiplicity = 1

C	-2.25944700	-0.11906400	0.10086500
C	-2.81450800	-0.89541000	-0.93796500
C	-3.13083500	0.68482400	0.86139000
C	-4.18042800	-0.85744800	-1.21118800
H	-2.16848600	-1.55023600	-1.52632800
C	-4.50024500	0.72500000	0.58546600
H	-2.73438700	1.29767200	1.67341800
C	-5.03167500	-0.04463200	-0.45192300
H	-4.58751800	-1.47207800	-2.01828300
H	-5.15474600	1.36132200	1.18686100
H	-6.10309700	-0.01777000	-0.66590600
C	-0.81574500	-0.13984600	0.39171100
C	0.16452400	-0.60094500	-0.40857800
C	-0.23274400	0.42796400	1.67372300
H	0.04583500	-1.02498000	-1.40517300
C	1.21308300	-0.10599800	1.68276800
H	-0.80058200	0.12156300	2.56700500
H	-0.24832200	1.53078000	1.63930000
C	1.54435200	-0.41288900	0.19141100
H	1.93133100	0.58625700	2.14233000
H	1.24811000	-1.05429000	2.24265200
C	2.42457000	-1.67535400	0.05675700
C	2.20463500	0.75217900	-0.55113300
O	1.95988300	-2.71129300	-0.35648100
C	3.86583400	-1.55447600	0.50357600
O	2.92122600	0.62801700	-1.51551700
O	1.85040100	1.94599200	-0.04505500
H	4.29339500	-2.55554100	0.64533000
H	4.42581800	-1.02508000	-0.28336700
H	3.95523200	-0.96224300	1.42819100
C	2.33184800	3.09683700	-0.74413300
H	1.95519600	3.96825800	-0.19465500
H	3.43220600	3.10472700	-0.77168400
H	1.95861600	3.10546200	-1.77975400

Compound 3

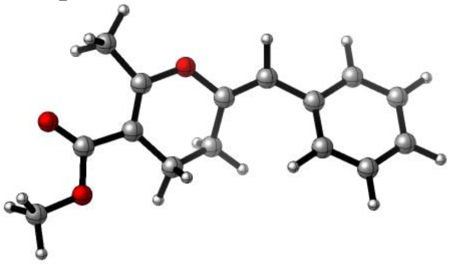

E (B3LYP-D3/Def2-SVP)) = -806.338540150
E (SMD(DMF)/M06/Def2-TZVPP//B3LYP-D3/Def2-SVP) = -806.655218626

Zero-point correction=	0.278649 (Hartree/Particle)
Thermal correction to Energy=	0.295243
Thermal correction to Enthalpy=	0.296187
Thermal correction to Gibbs Free Energy=	0.233008

Charge = 0 Multiplicity = 1

C	-3.02724900	0.31822600	-0.17939200
C	-3.05417500	-1.06895900	-0.42801900
C	-4.25233400	0.96545300	0.07935400
C	-4.25199300	-1.78517600	-0.37715600
H	-2.13030800	-1.58521900	-0.69728900
C	-5.44971700	0.25004400	0.12927100
H	-4.25716100	2.04497500	0.25354200
C	-5.45472200	-1.13126000	-0.09181100
H	-4.24735800	-2.85999500	-0.57686500
H	-6.38553500	0.77411600	0.34063300
H	-6.39191300	-1.69221900	-0.05535300
C	-1.79049700	1.11707900	-0.23420300
C	-0.57419800	0.79014800	0.23870500
H	-1.86926300	2.11432700	-0.68004400
C	-0.12665100	-0.41011300	1.01229600
C	1.12136900	-1.01601100	0.36210600
H	0.13941100	-0.06262800	2.02807200
H	-0.93802700	-1.13780600	1.11921600
C	2.13428500	0.07570300	0.09235700
H	0.85367600	-1.53979800	-0.57355200
H	1.56280500	-1.77684700	1.02139700
C	1.73370500	1.37650000	-0.00206400
C	3.55652900	-0.26397900	-0.07785600
O	0.42318800	1.73612400	0.05898100
C	2.60412100	2.57939600	-0.20965600
O	3.75475500	-1.60504600	0.00569200
O	4.47596100	0.50545700	-0.27899900
H	3.39696700	2.62760400	0.54914200
H	3.12390900	2.51850700	-1.17726900
H	1.98399400	3.48488500	-0.17509900
C	5.09863000	-2.04643700	-0.15804500
H	5.75442600	-1.61253900	0.61339200
H	5.07673300	-3.14013300	-0.06710800
H	5.49379800	-1.75318800	-1.14352400

LiBr(DMF)$_3$

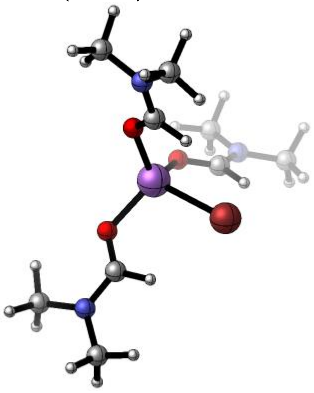

E (B3LYP-D3/Def2-SVP)) = -3326.60179429
E (SMD(DMF)/M06/Def2-TZVPP//B3LYP-D3/Def2-SVP) = -3327.13220751

Zero-point correction=	0.312752 (Hartree/Particle)
Thermal correction to Energy=	0.337724
Thermal correction to Enthalpy=	0.338668
Thermal correction to Gibbs Free Energy=	0.251619

Charge = 0 Multiplicity = 1

Li	-0.00303900	-0.00157100	-0.53672600
O	1.19236700	-1.44543100	-1.04644900
C	1.75675300	-2.12261300	-0.18712000
N	2.60439700	-3.14206900	-0.45705200
H	1.59210300	-1.92280700	0.89361700
C	3.23417300	-3.89731600	0.60699100
C	2.91430200	-3.51681300	-1.82433100
H	4.33455800	-3.82332400	0.54649900
H	2.91195700	-3.50841400	1.58331300
H	2.96117100	-4.96596300	0.54836800
H	3.99680000	-3.41996900	-2.01868200
H	2.62178800	-4.56386800	-2.01682800
H	2.36269300	-2.85483900	-2.50336900
O	0.65319700	1.75477200	-1.04491900
C	0.96312700	2.57995500	-0.18555300
N	1.42781400	3.82170000	-0.45545300
H	0.87338800	2.33706100	0.89517100
C	1.77363300	4.74224800	0.60858000
C	1.59714600	4.27752800	-1.82273900
H	1.16402100	5.66142700	0.54976700
H	1.59718700	4.26903400	1.58490400
H	2.83704600	5.03465300	0.54829100
H	0.97576200	5.16953300	-2.01552200
H	2.65107800	4.54303300	-2.01684600
H	1.29484200	3.47059800	-2.50174600
O	-1.85107700	-0.31122500	-1.04748700
C	-2.72005400	-0.45875800	-0.18798700
N	-4.02780700	-0.67710300	-0.45764500
H	-2.46413100	-0.41827800	0.89269100
C	-4.99699500	-0.84203600	0.60664100
C	-4.50828100	-0.75422900	-1.82480100
H	-5.48742200	-1.82979700	0.54512100

H	-4.49829400	-0.76082400	1.58284800
H	-5.78261900	-0.06777400	0.54933700
H	-4.96945700	-1.73809500	-2.02046500
H	-5.26598600	0.02576500	-2.01567800
H	-3.65899100	-0.60962500	-2.50404500
Br	-0.00308400	-0.00209200	2.01335000

DMF

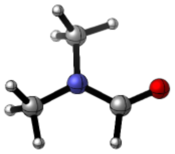

E (B3LYP-D3/Def2-SVP)) = -248.337125393
E (SMD(DMF)/M06/Def2-TZVPP//B3LYP-D3/Def2-SVP) = -248.457828454

Zero-point correction=	0.101931 (Hartree/Particle)
Thermal correction to Energy=	0.107954
Thermal correction to Enthalpy=	0.108898
Thermal correction to Gibbs Free Energy=	0.072952

Charge = 0 Multiplicity = 1

C	0.87027600	-0.64521700	0.00023500
N	-0.34624100	-0.02077400	0.00007000
C	-1.58784600	-0.76021900	0.00010700
H	-2.19569600	-0.52470500	-0.89261800
H	-1.38273100	-1.84125600	-0.00002000
H	-2.19554000	-0.52488300	0.89299000
C	-0.42397900	1.42673000	0.00007400
H	-0.95950800	1.79252700	-0.89419900
H	-0.95929100	1.79253800	0.89447800
H	0.59708400	1.82941600	-0.00003400
O	1.95082300	-0.09337500	-0.00039300
H	0.76208100	-1.75898500	-0.00044000

Copies of ^{1}H and $^{13}C\{1H\}$ NMR spectra of isolated compounds

(2) methyl 1-acetyl-3-phenylcyclopent-2-ene-1-carboxylate

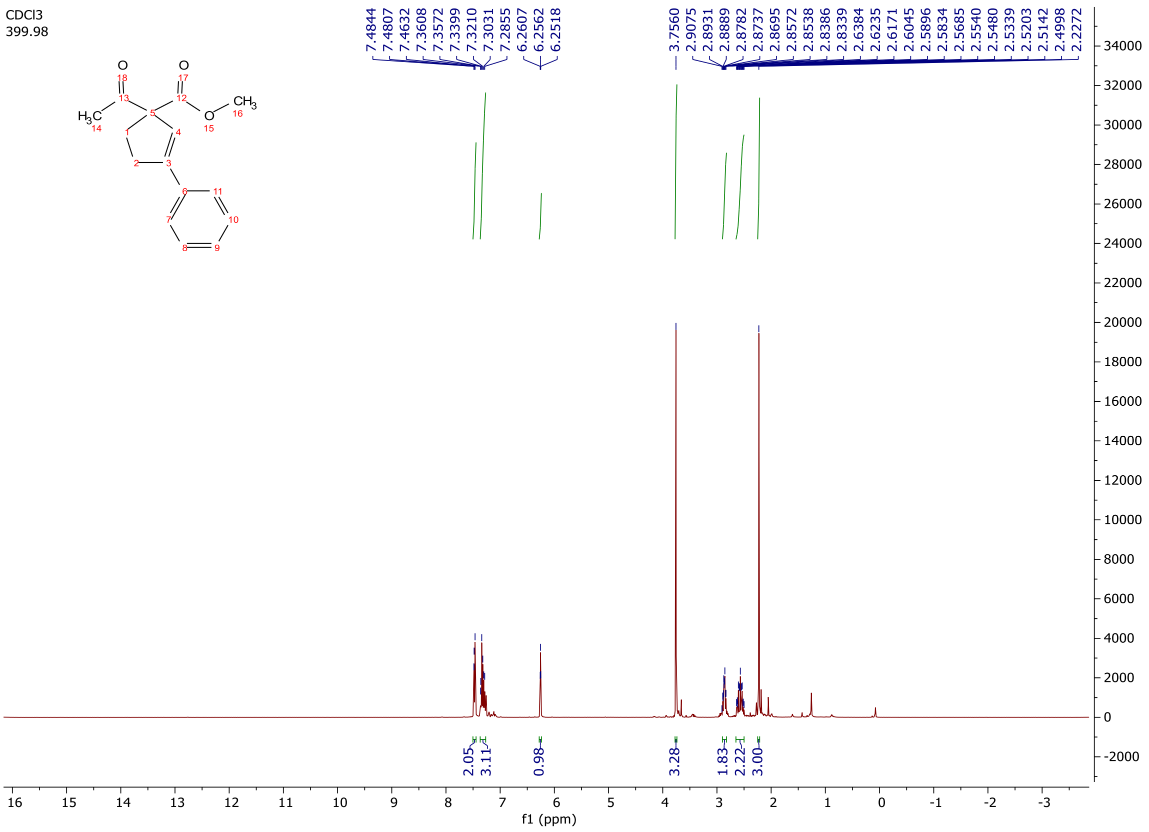

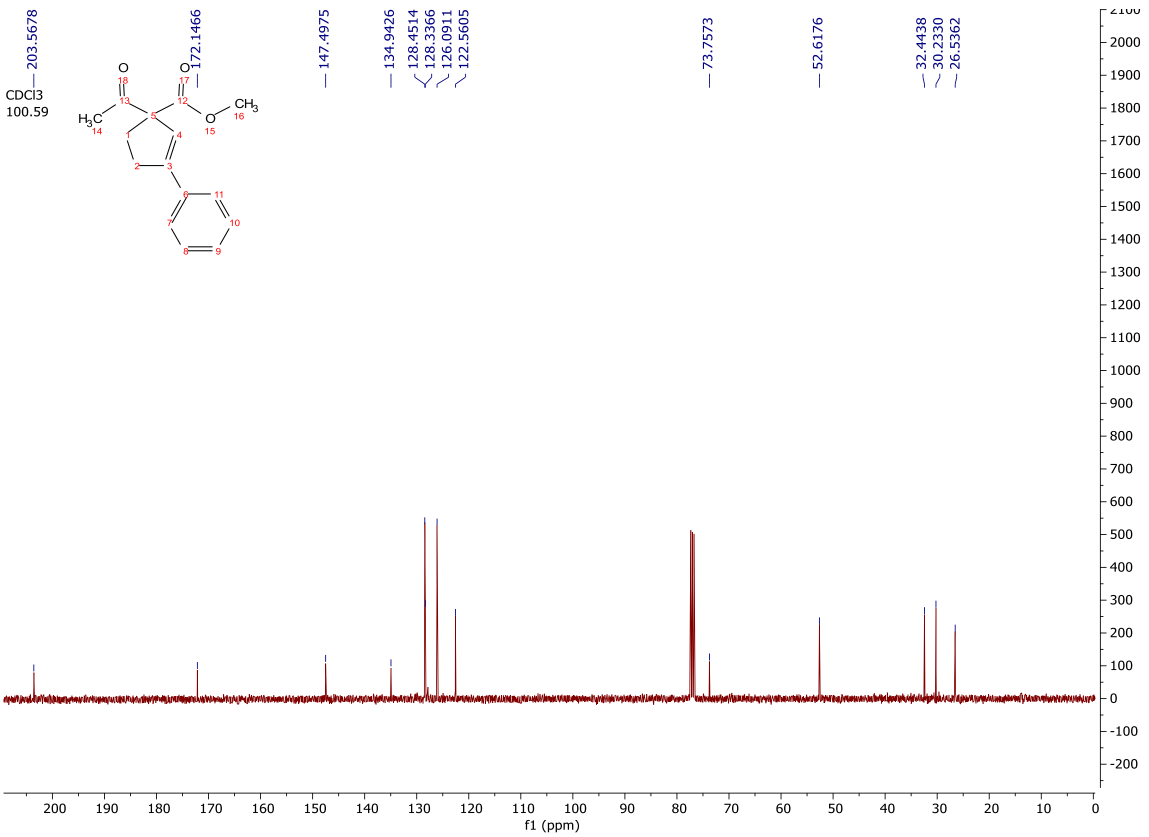

(4) methyl 1-acetyl-3-(4-methoxyphenyl)cyclopent-2-ene-1-carboxylate

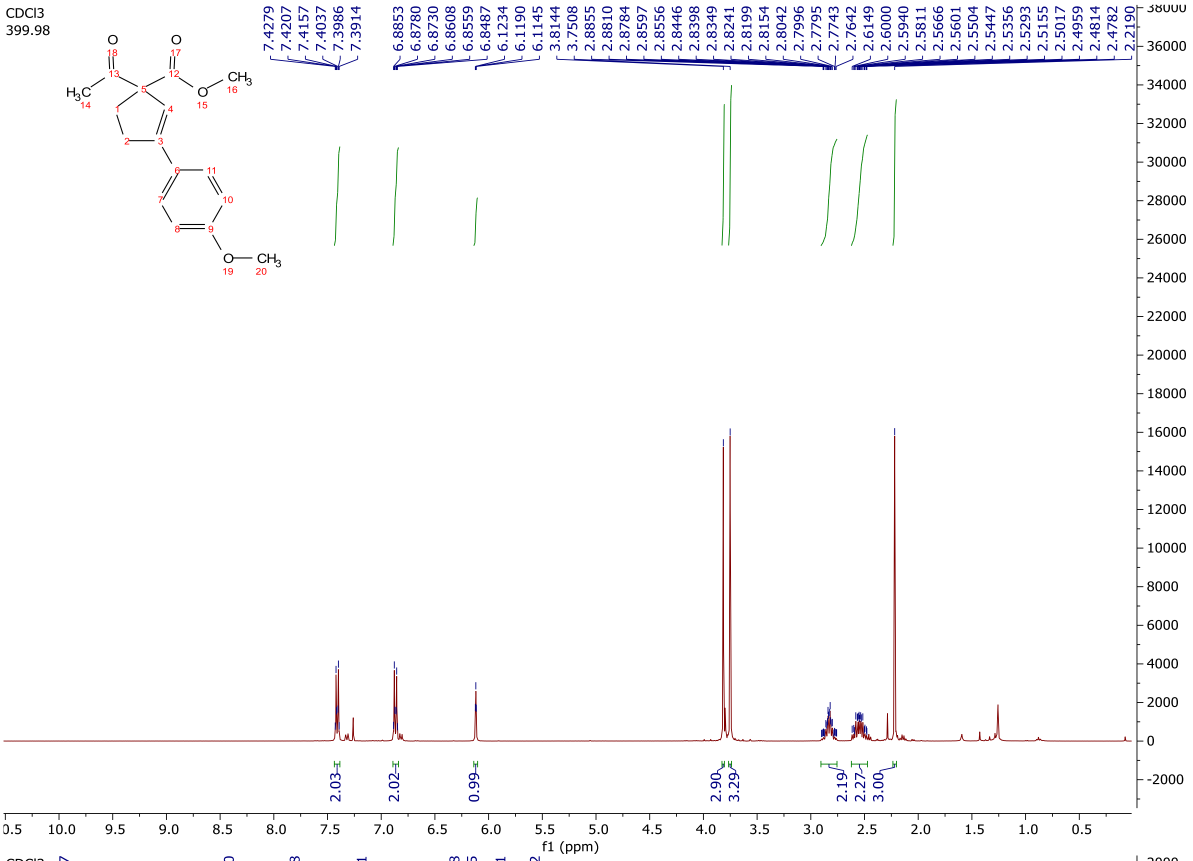

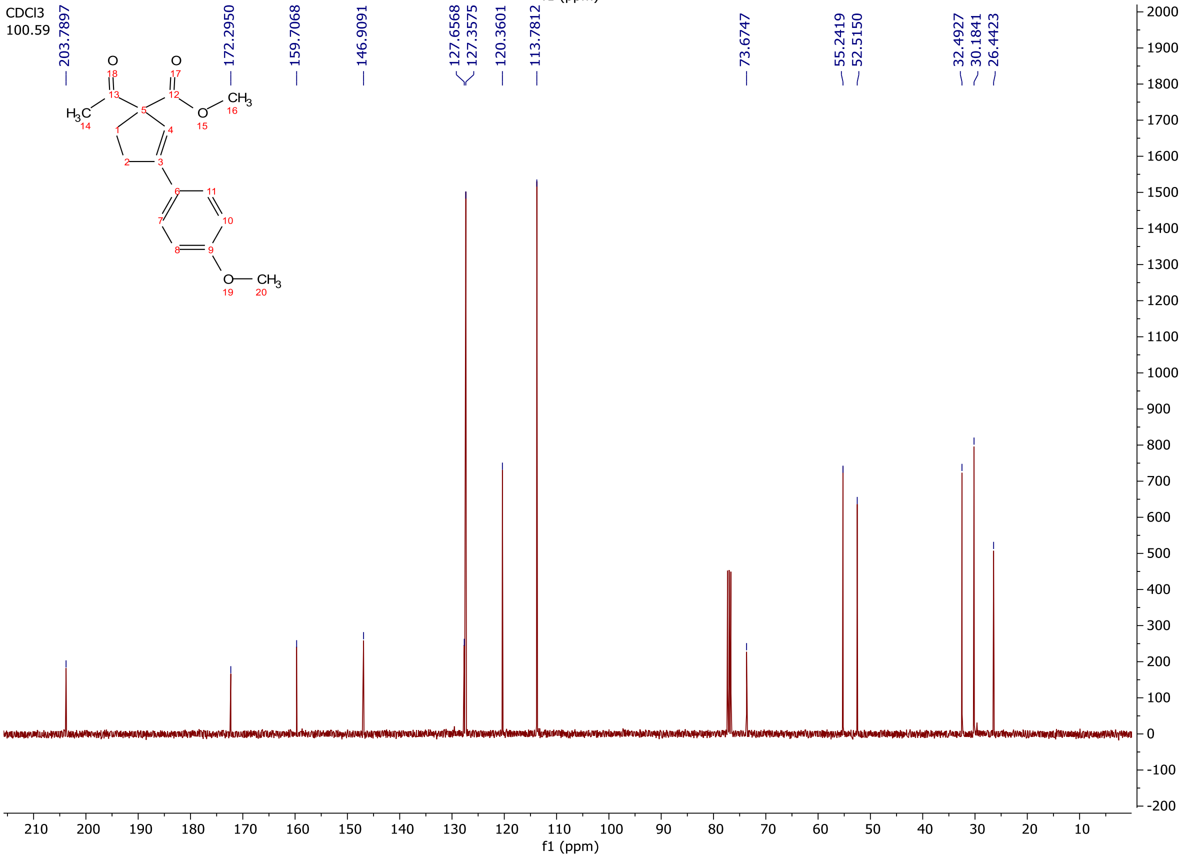

(5) methyl 1-acetyl-3-(3-methoxyphenyl)cyclopent-2-ene-1-carboxylate

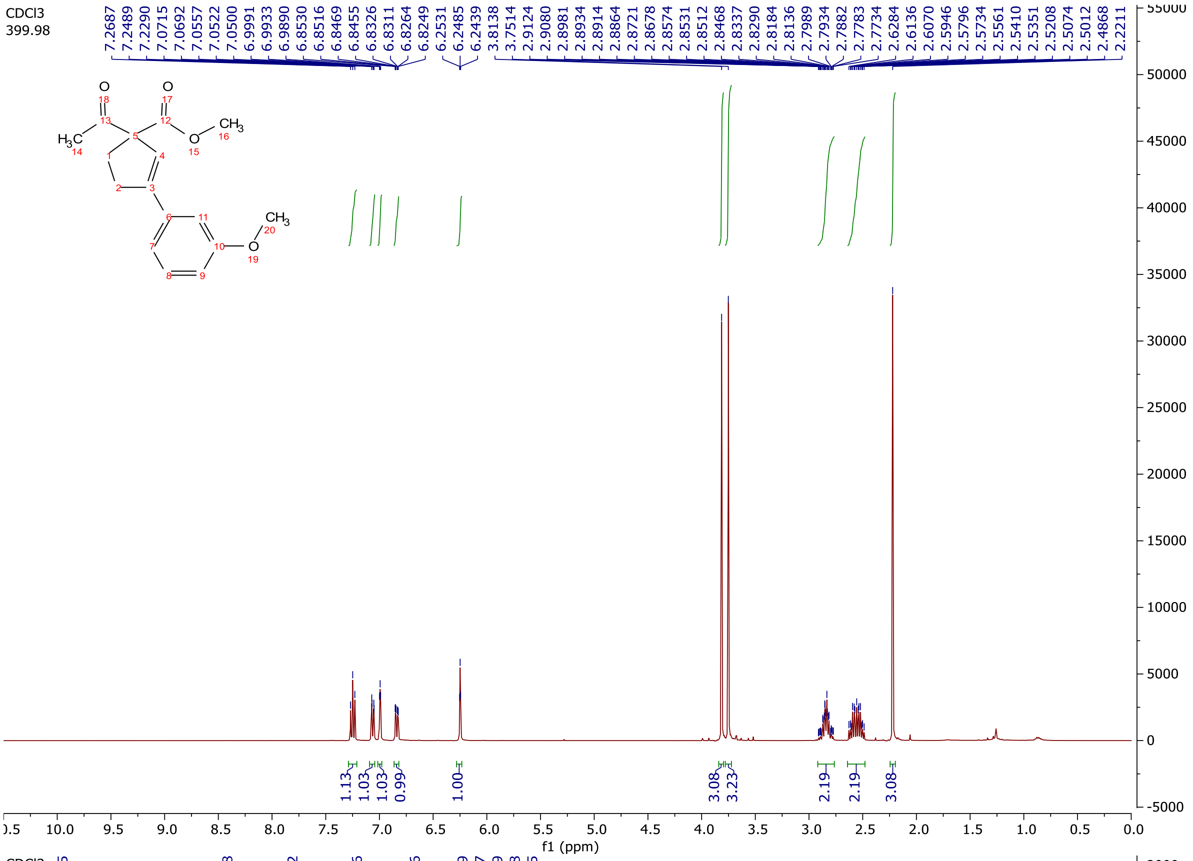

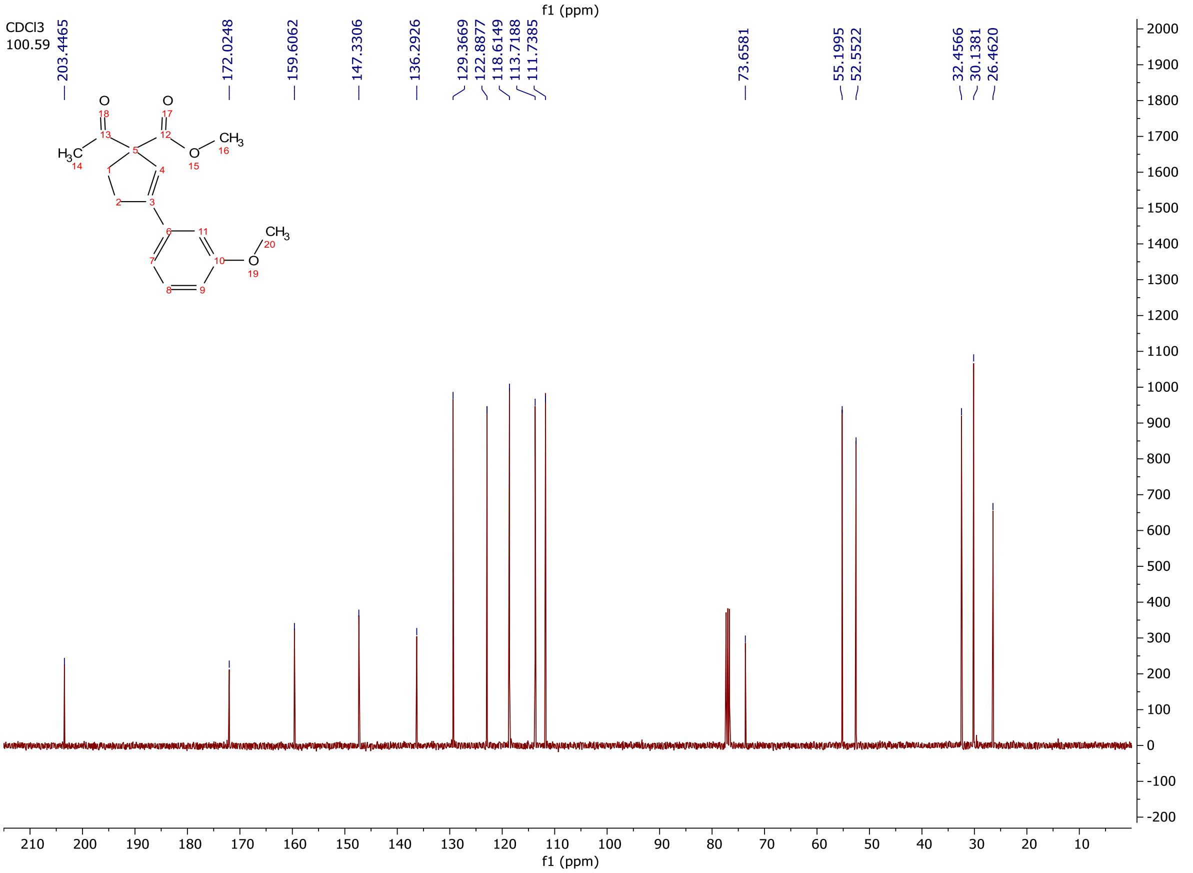

(6) methyl 1-acetyl-3-(2-methoxyphenyl)cyclopent-2-ene-1-carboxylate

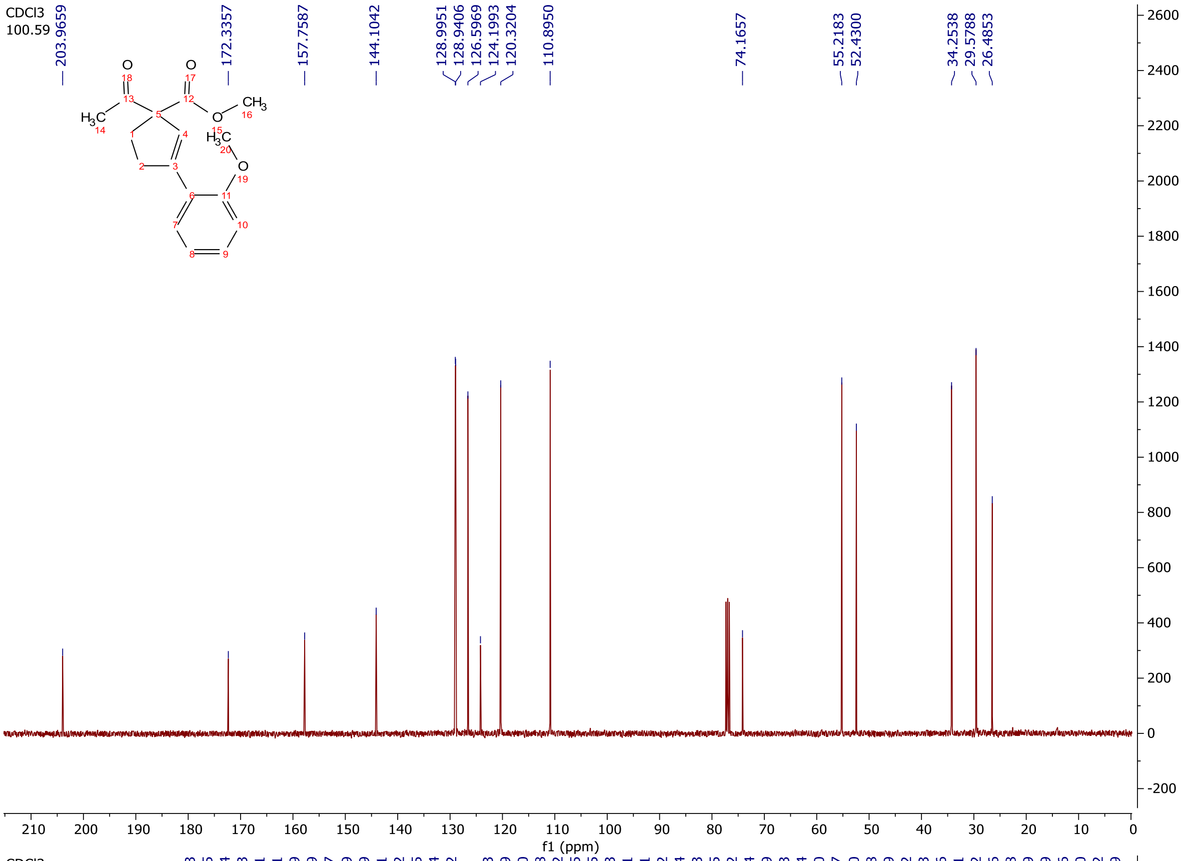

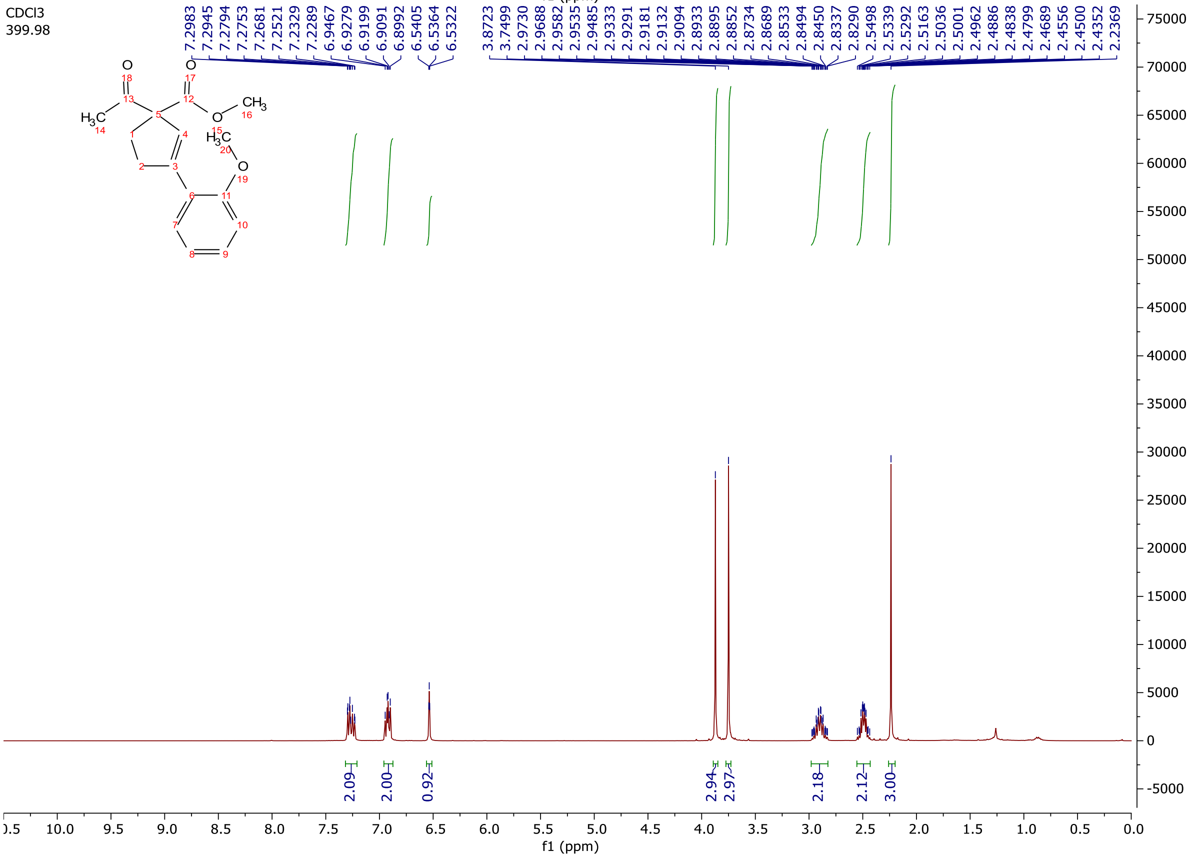

(7) methyl 3-(4-acetamidophenyl)-1-acetylcyclopent-2-ene-1-carboxylate

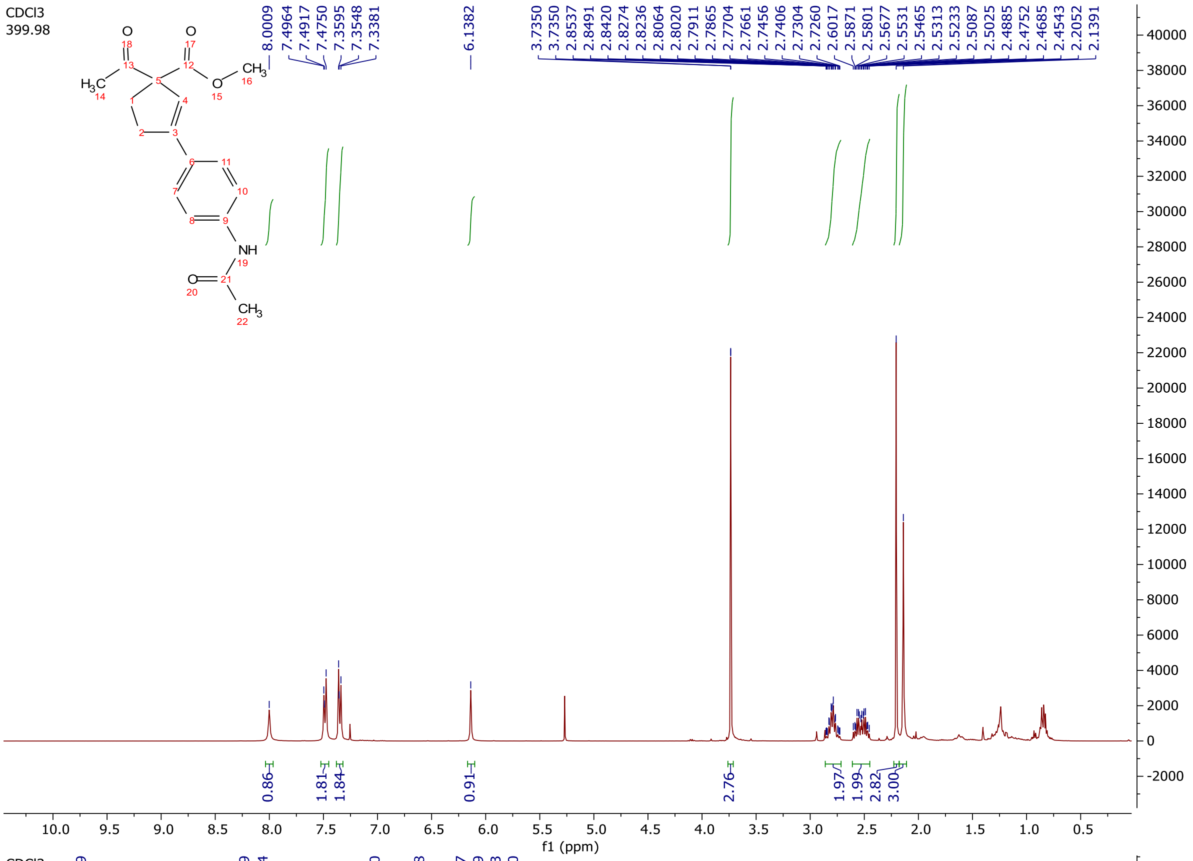

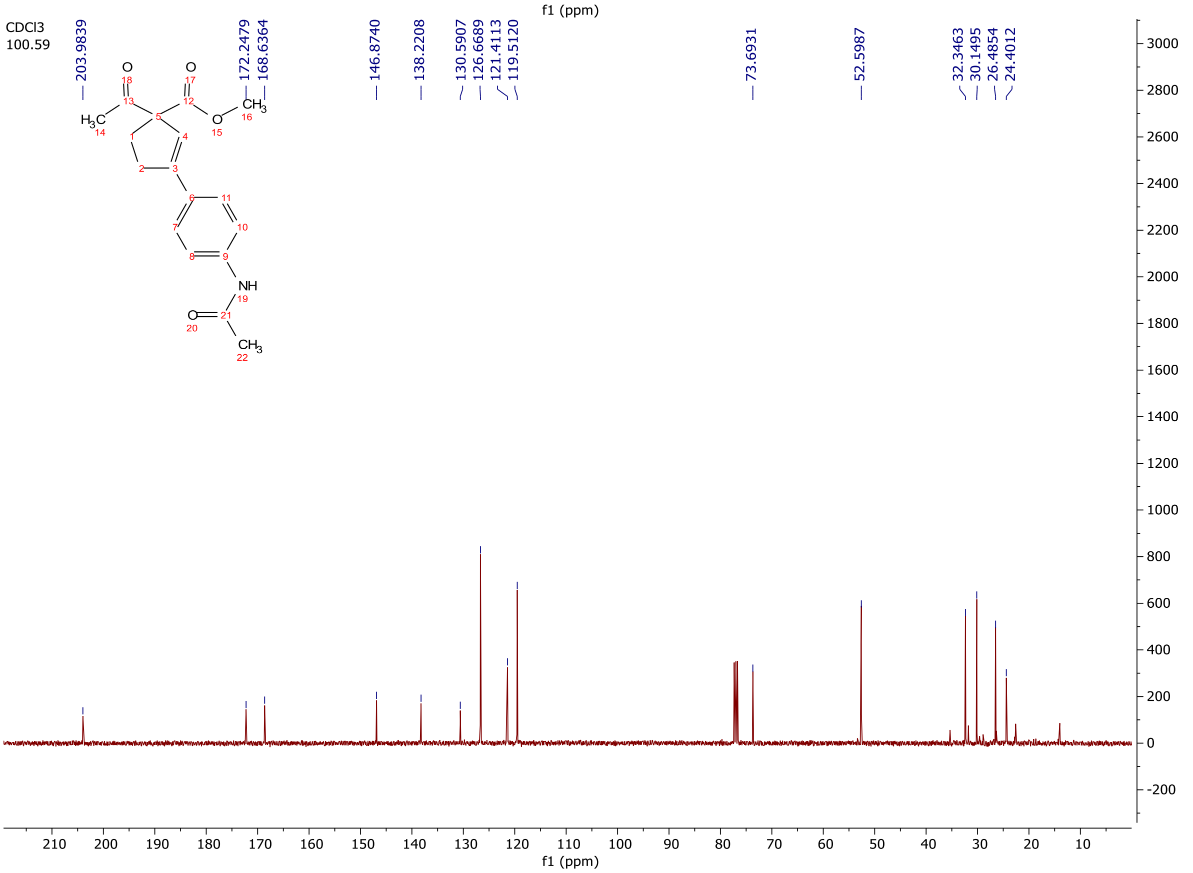

(8) methyl 1-acetyl-3-(p-tolyl)cyclopent-2-ene-1-carboxylate

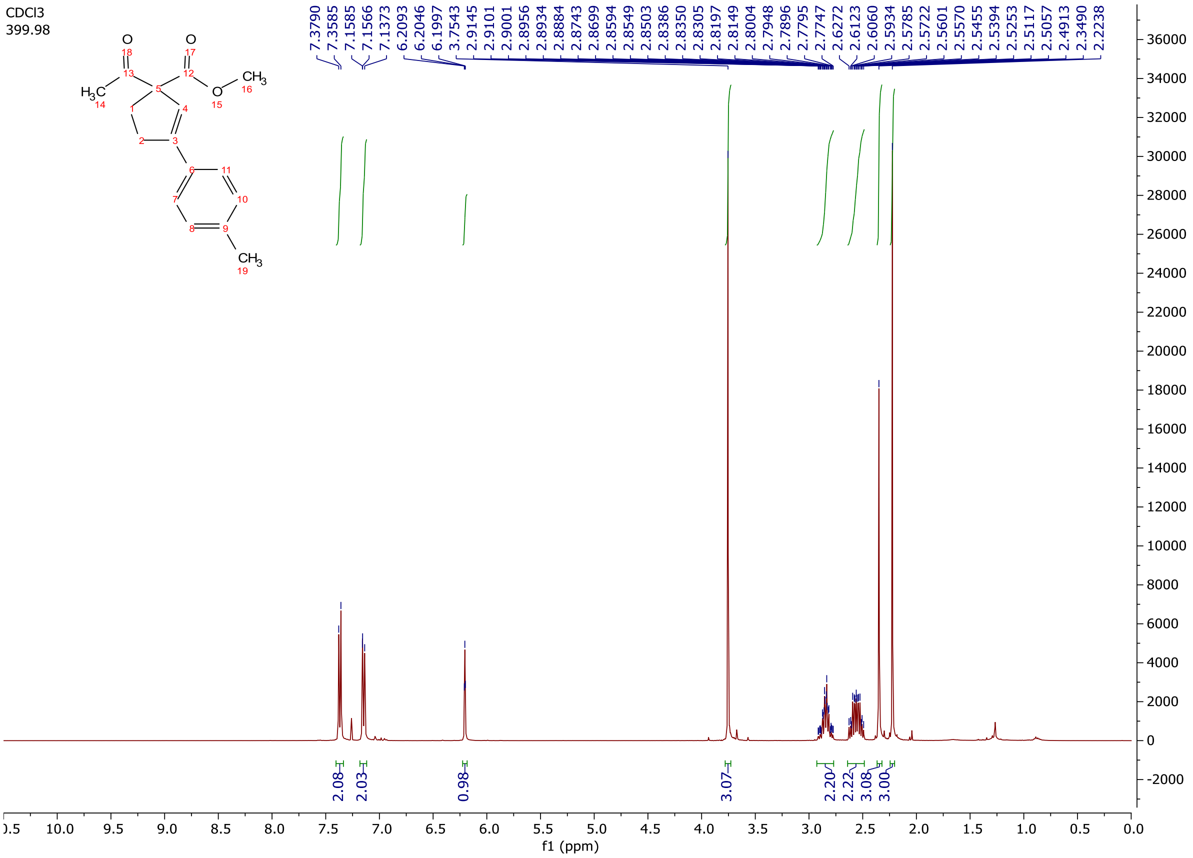

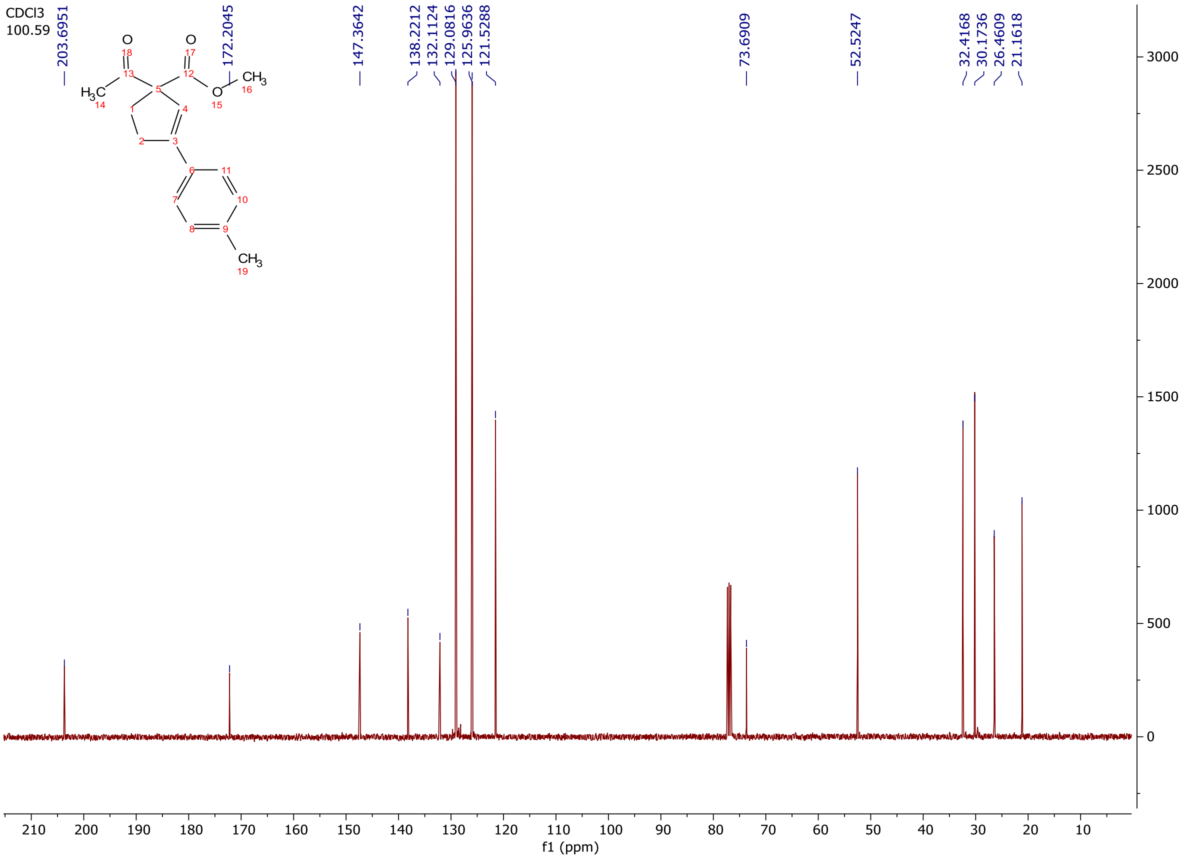

(9) methyl 1-acetyl-3-(o-tolyl)cyclopent-2-ene-1-carboxylate

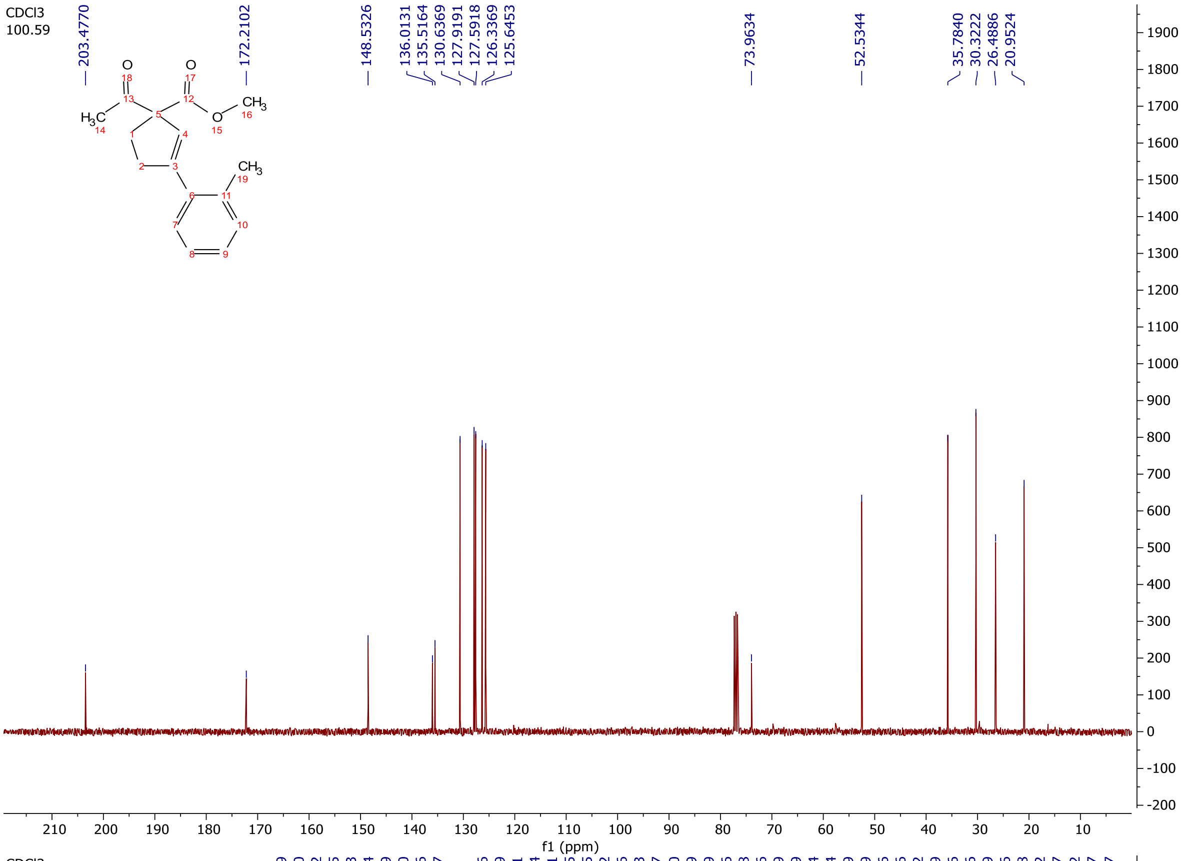

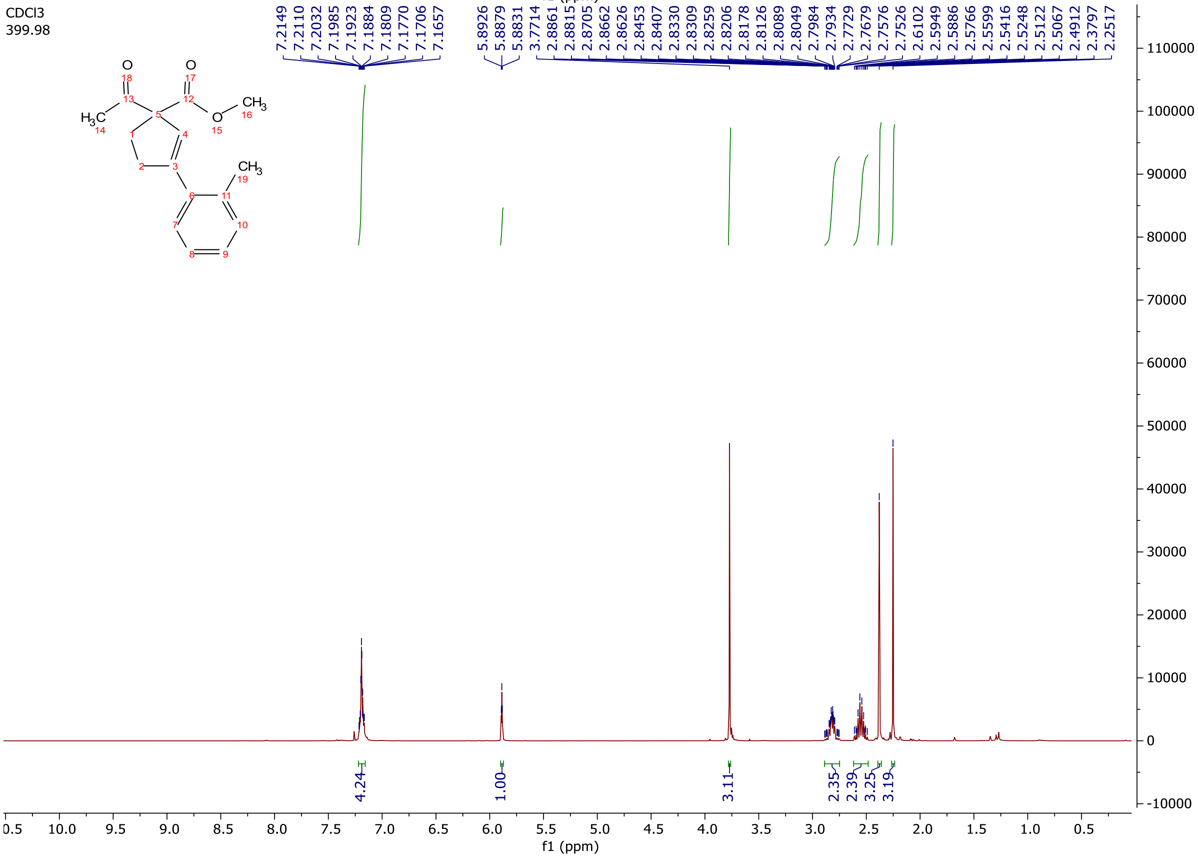

(10) methyl 1-acetyl-3-(4-chlorophenyl)cyclopent-2-ene-1-carboxylate

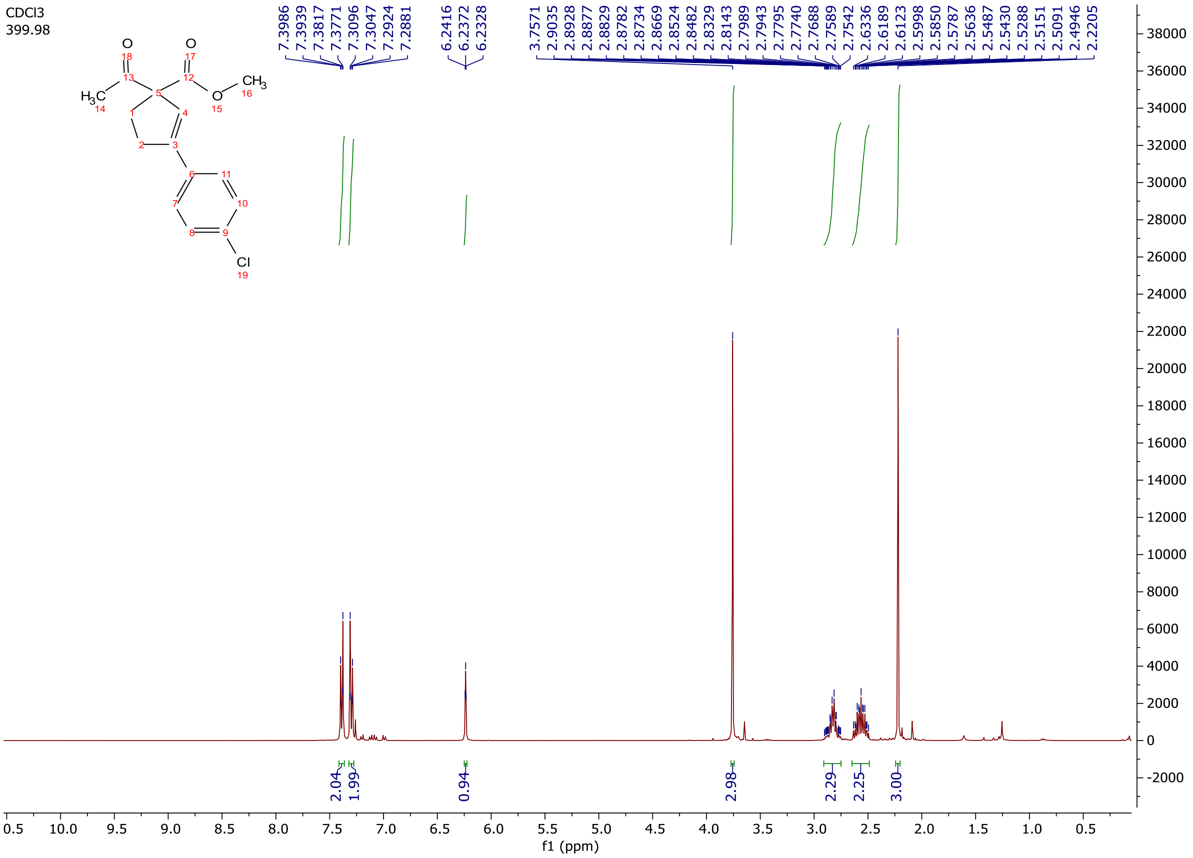

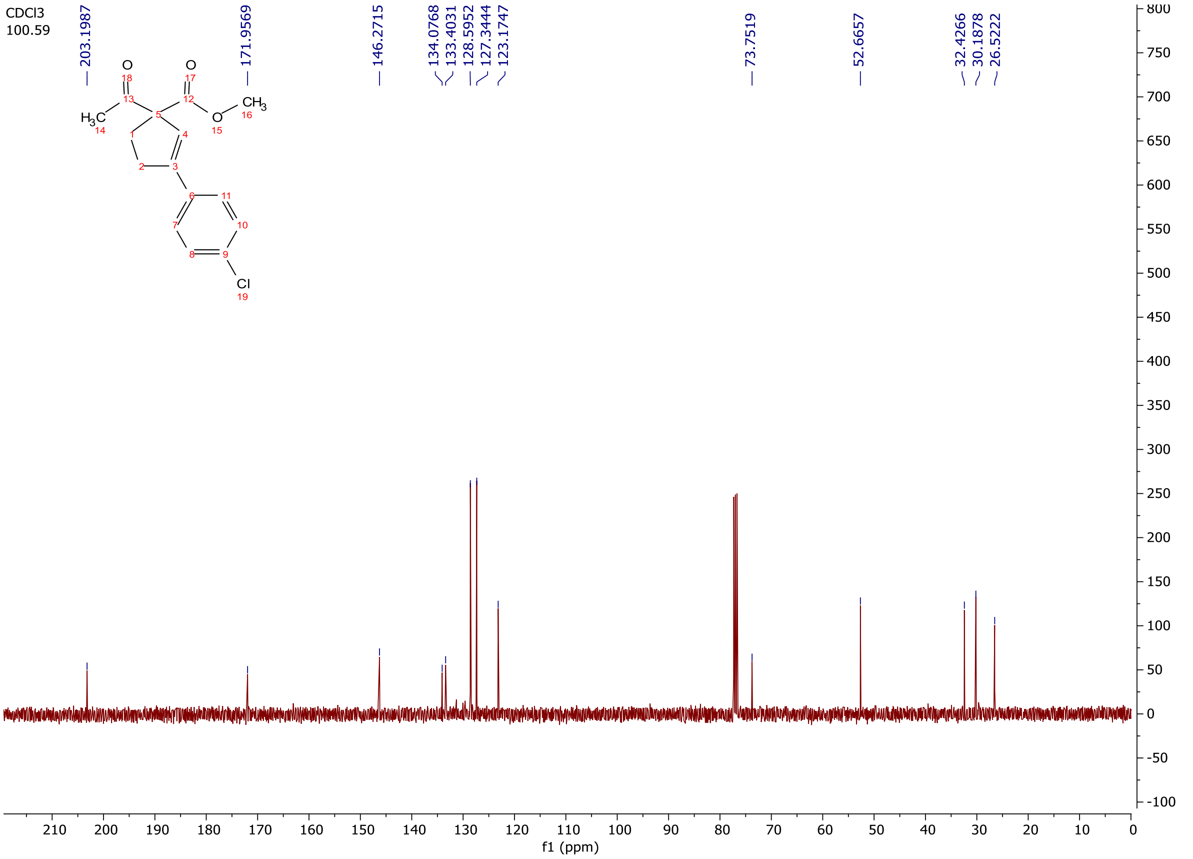

(11) methyl 1-acetyl-3-(2-chlorophenyl)cyclopent-2-ene-1-carboxylate

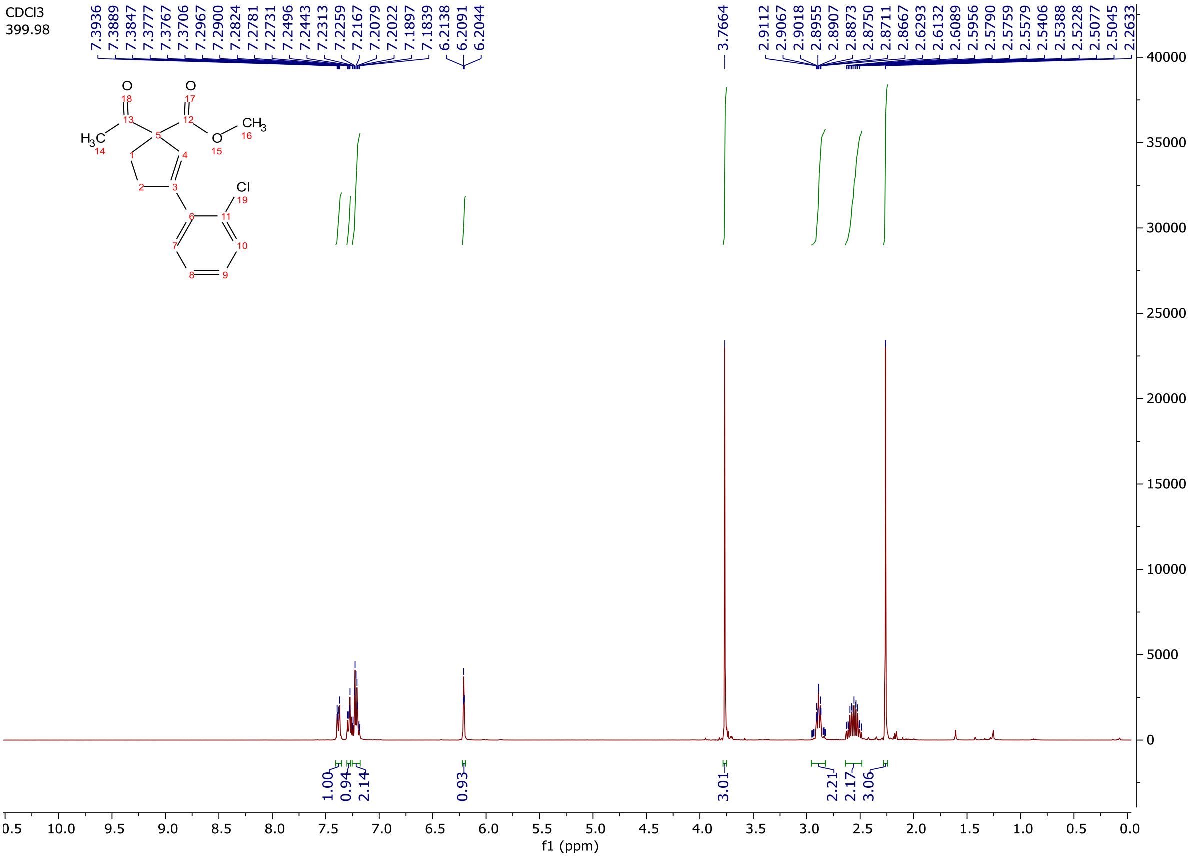

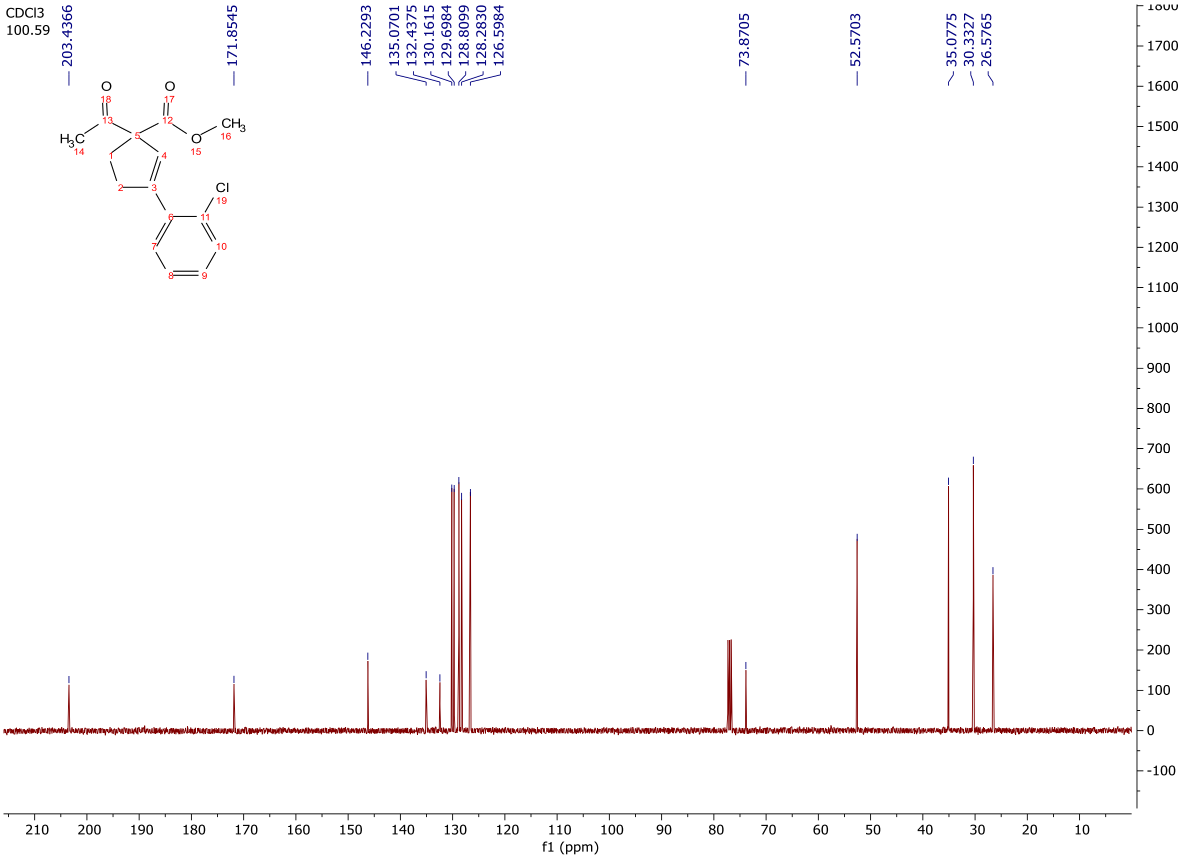

(12) methyl 1-acetyl-3-(4-fluorophenyl)cyclopent-2-ene-1-carboxylate

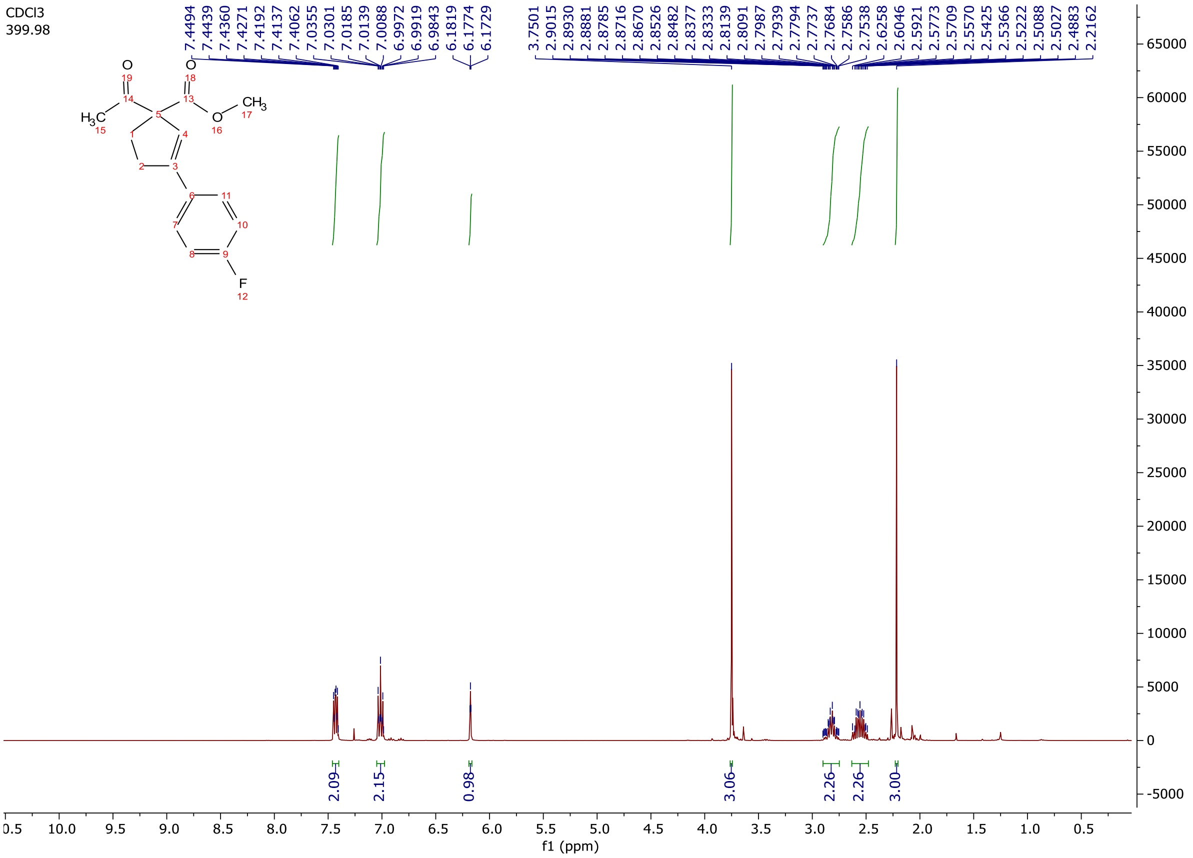

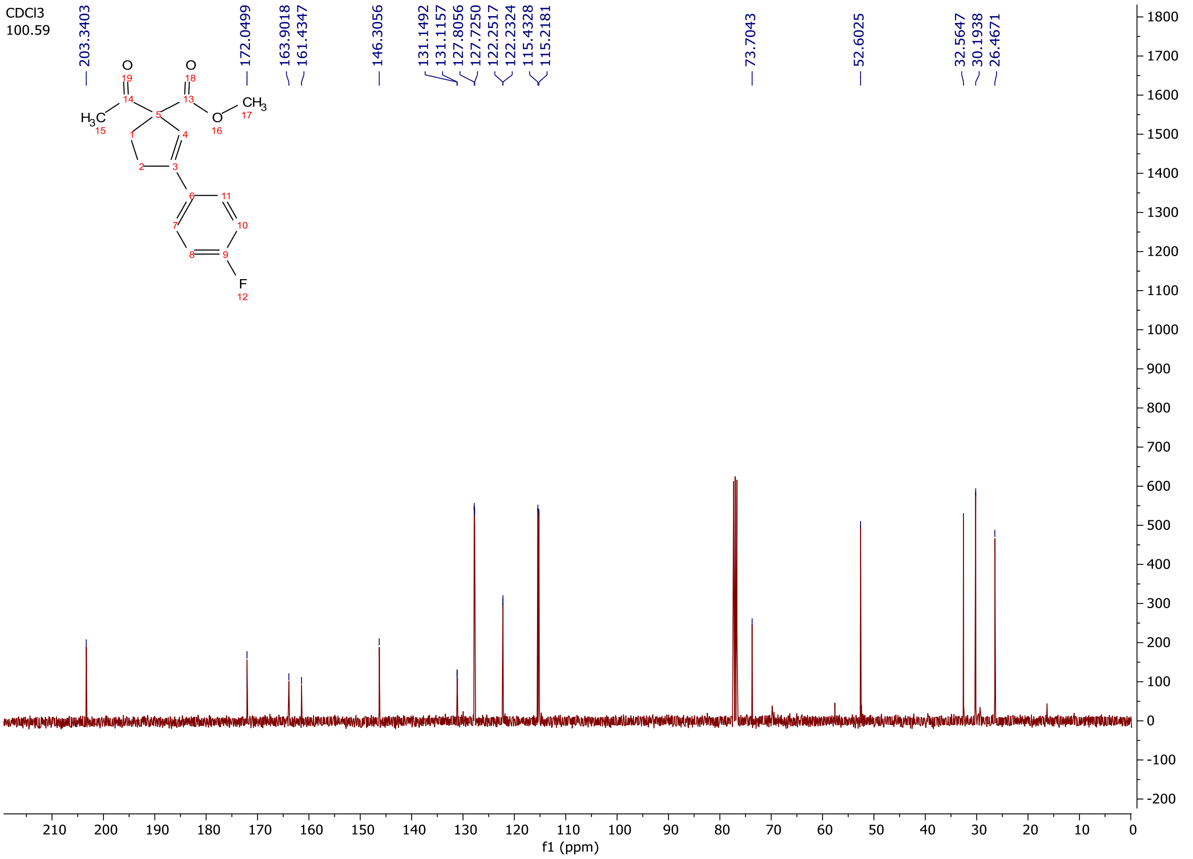

(13) methyl 1-acetyl-3-(4-(trifluoromethyl)phenyl)cyclopent-2-ene-1-carboxylate

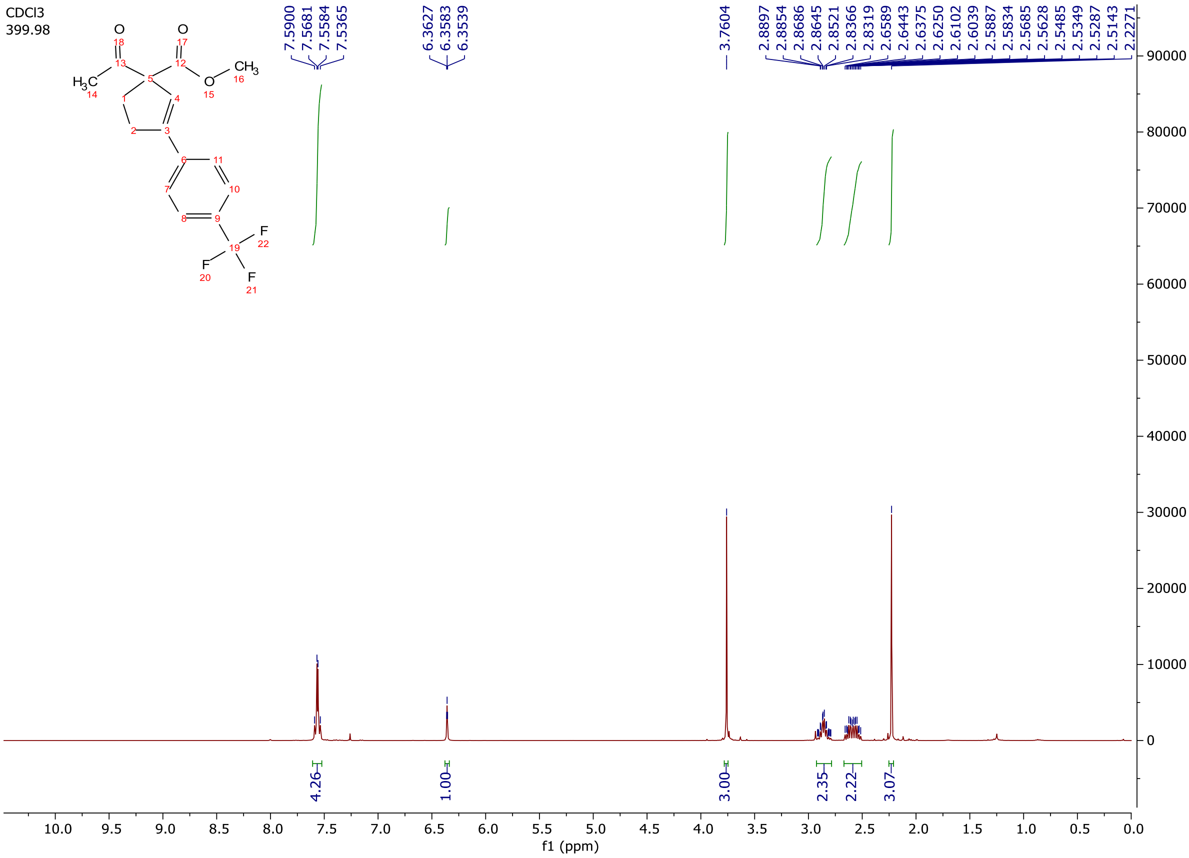

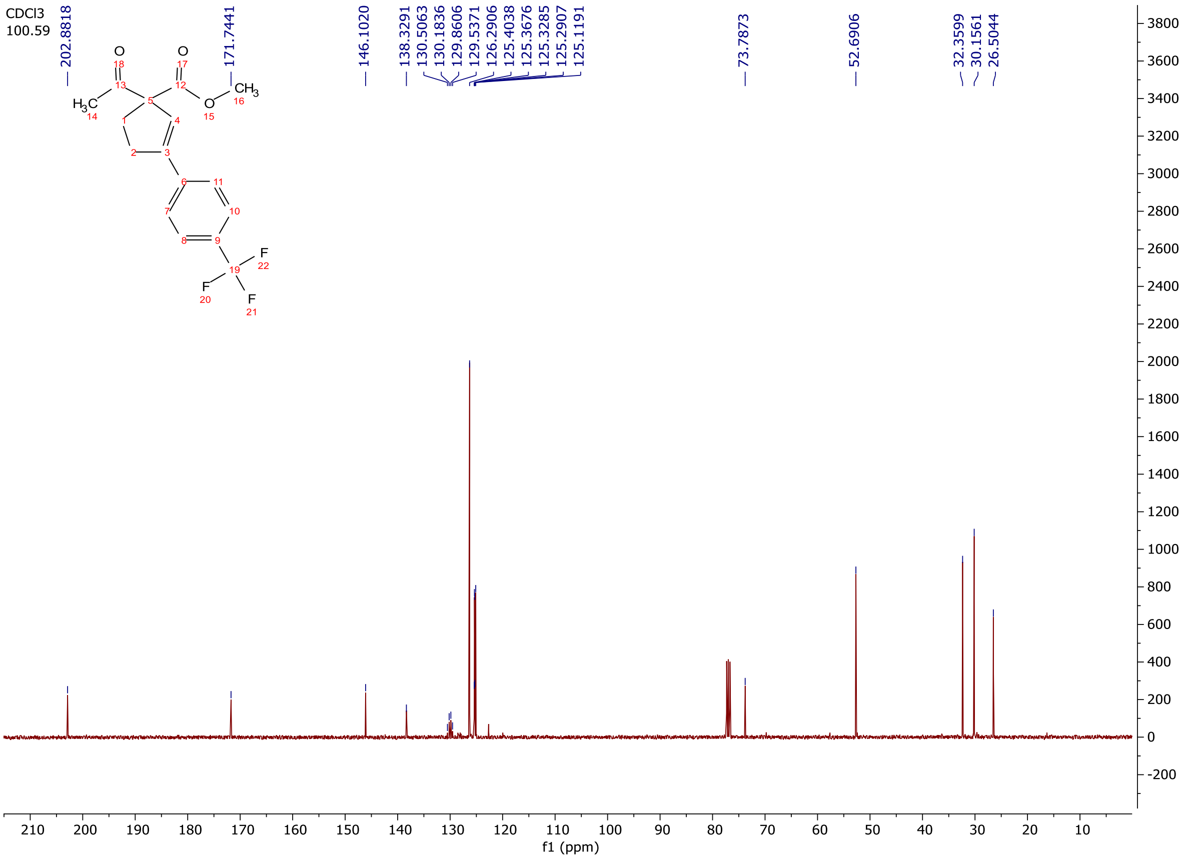

(14) methyl 1-acetyl-3-(4-cyanophenyl)cyclopent-2-ene-1-carboxylate

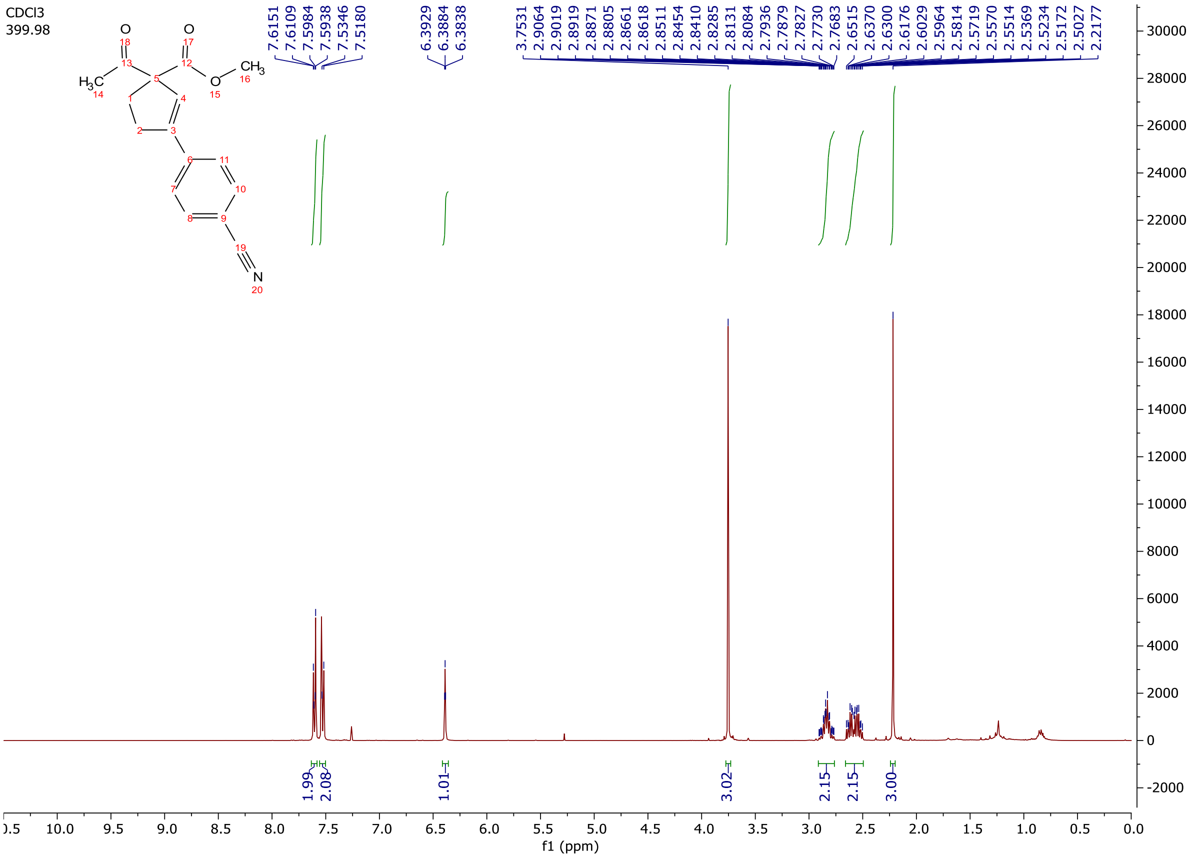

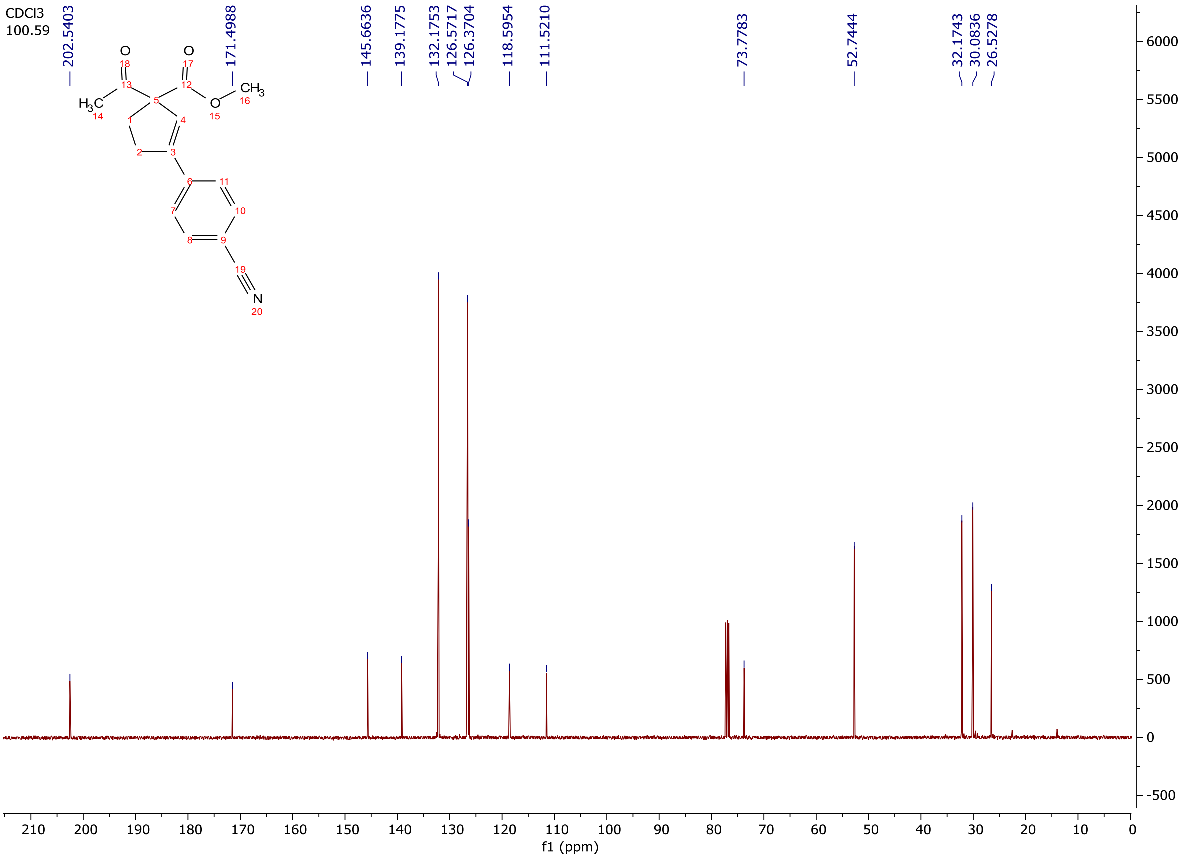

(15) methyl 1-acetyl-3-(4-acetylphenyl)cyclopent-2-ene-1-carboxylate

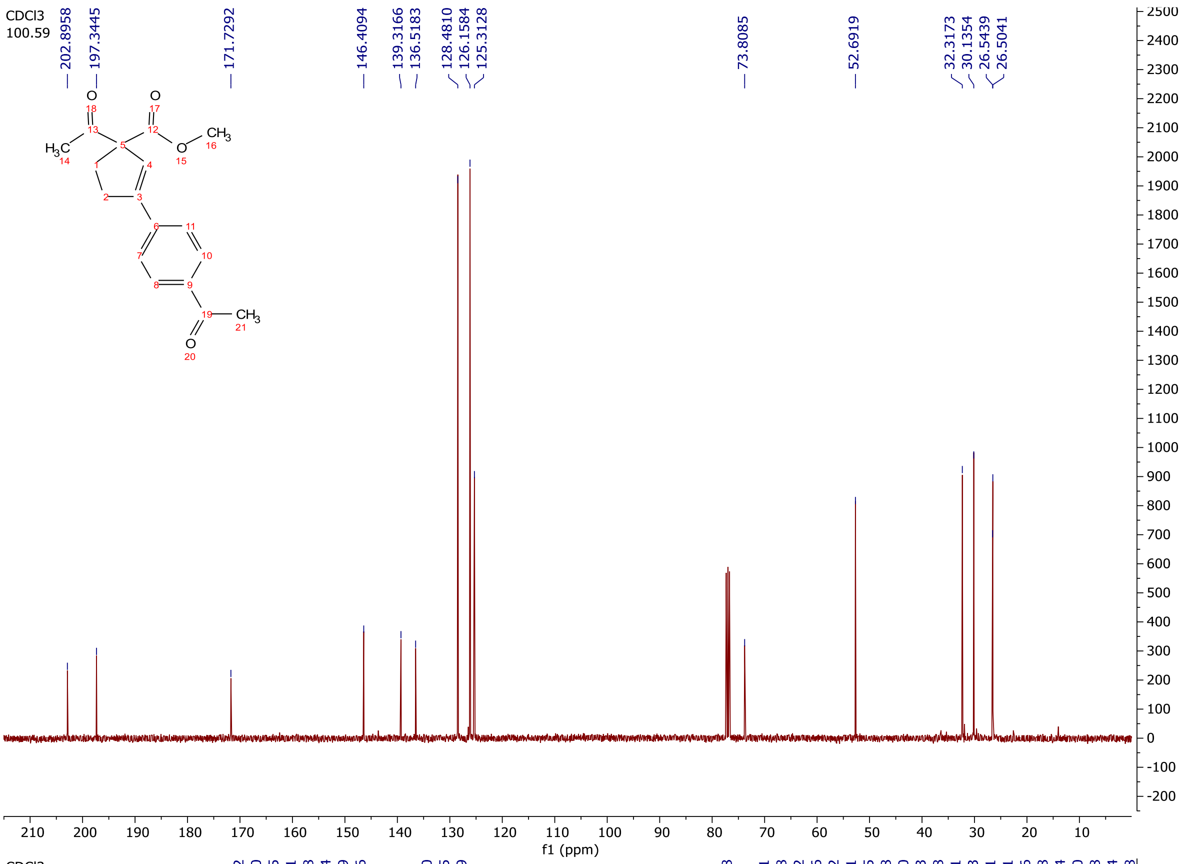

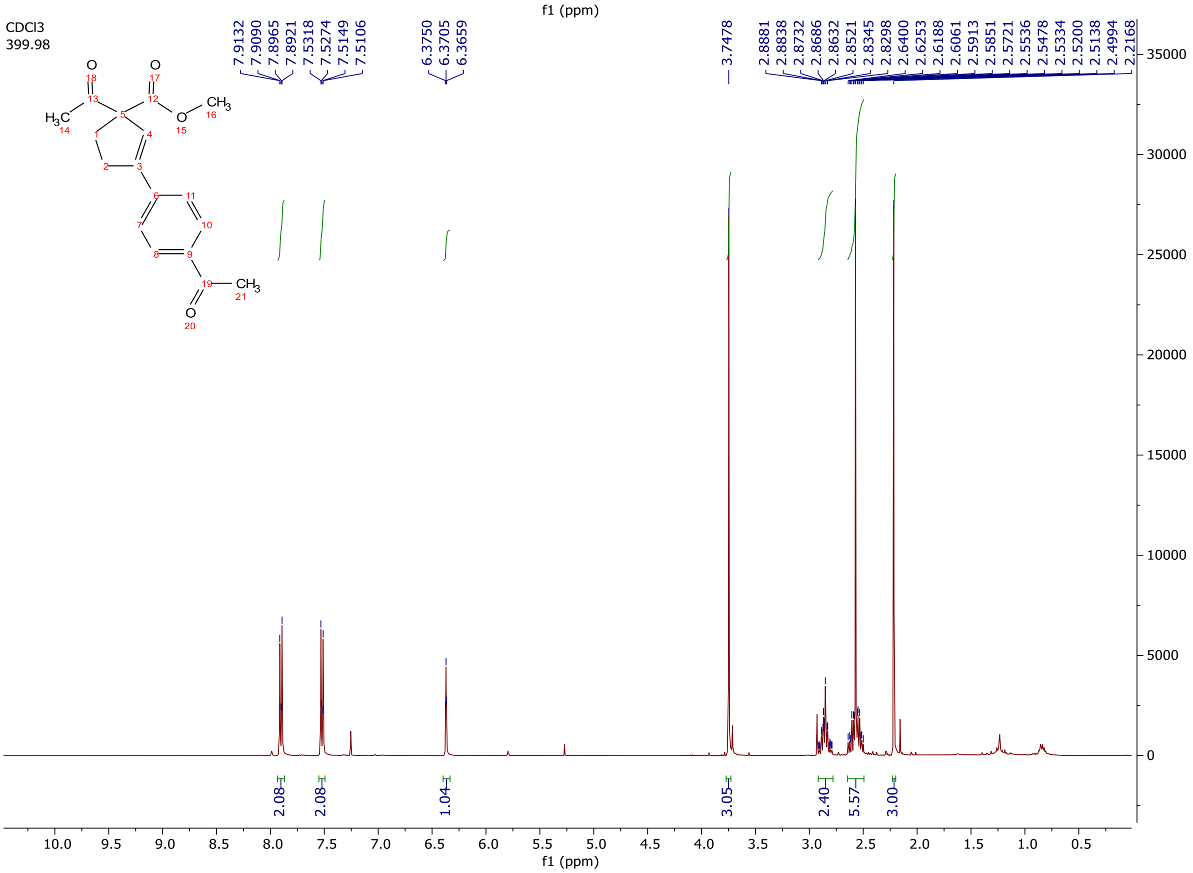

(16) methyl 1-acetyl-3-(4-formylphenyl)cyclopent-2-ene-1-carboxylate

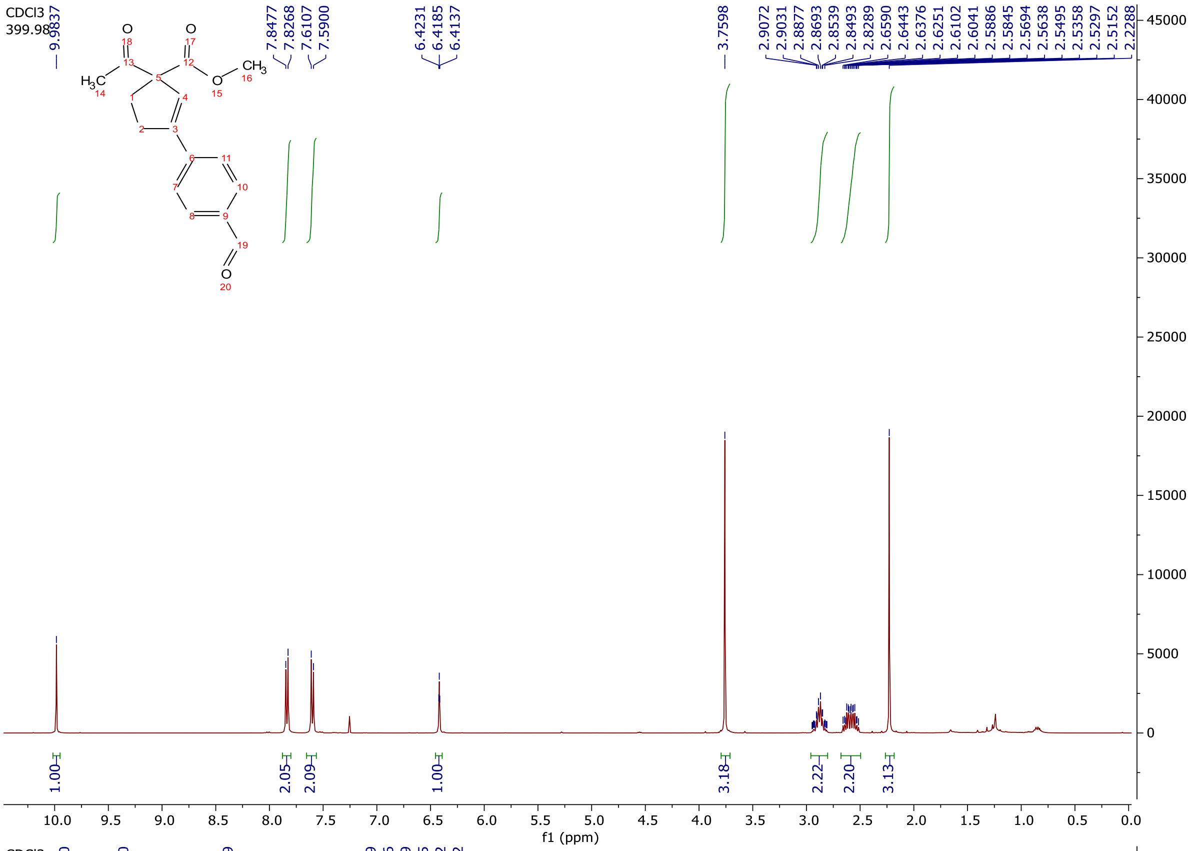

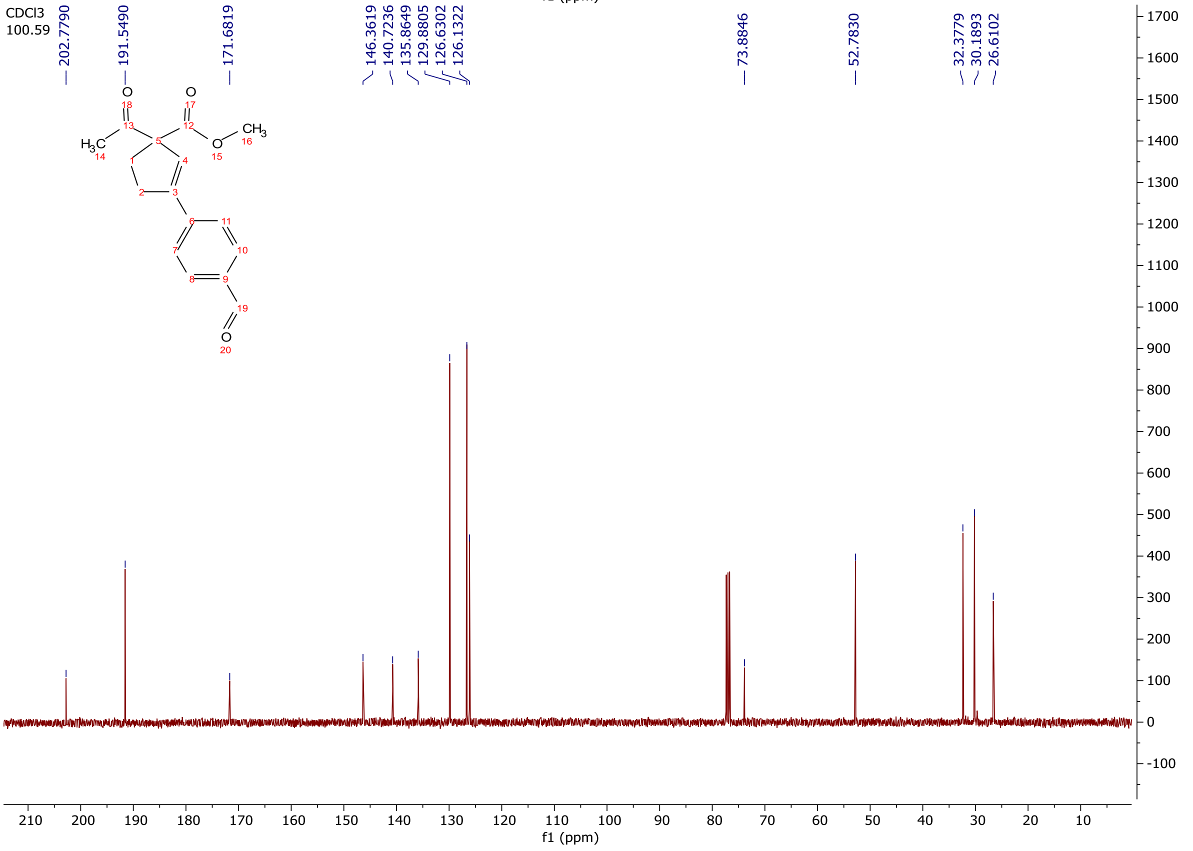

(17) methyl 1-acetyl-3-(4-nitrophenyl)cyclopent-2-ene-1-carboxylate

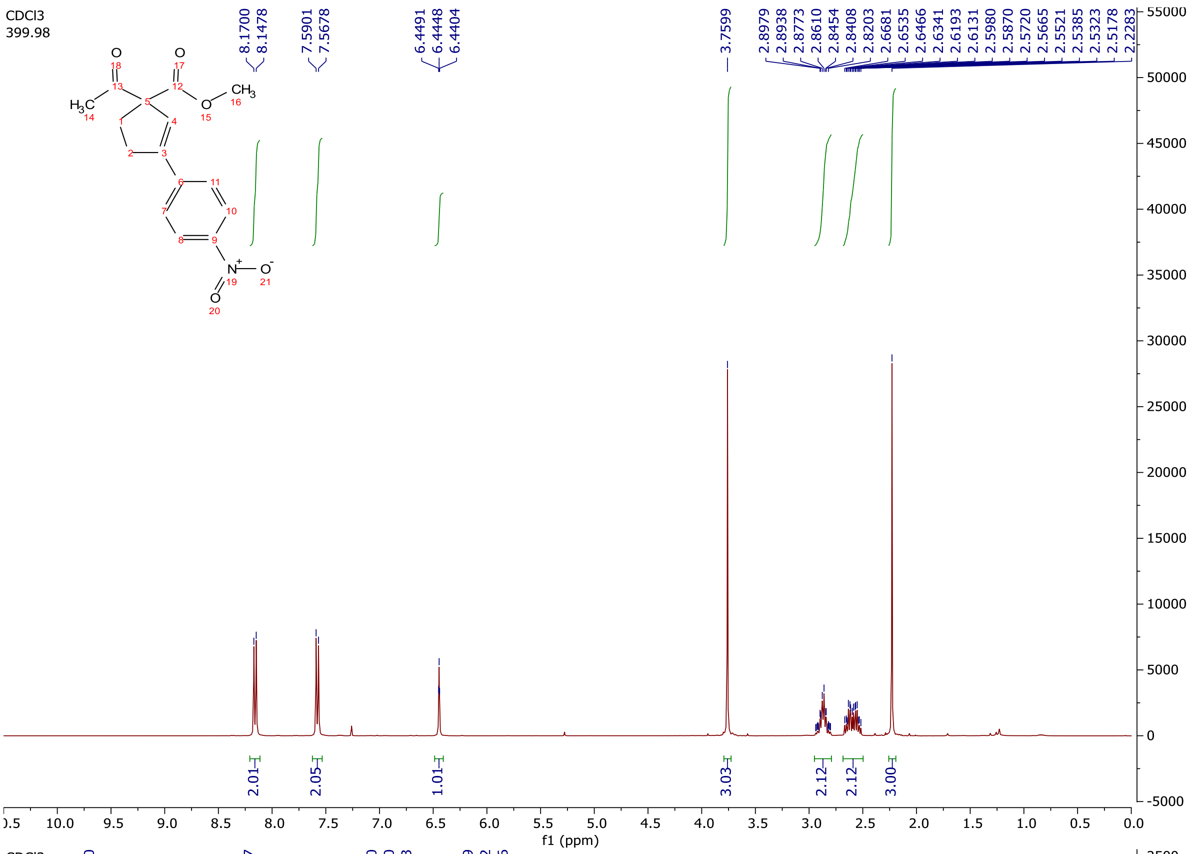

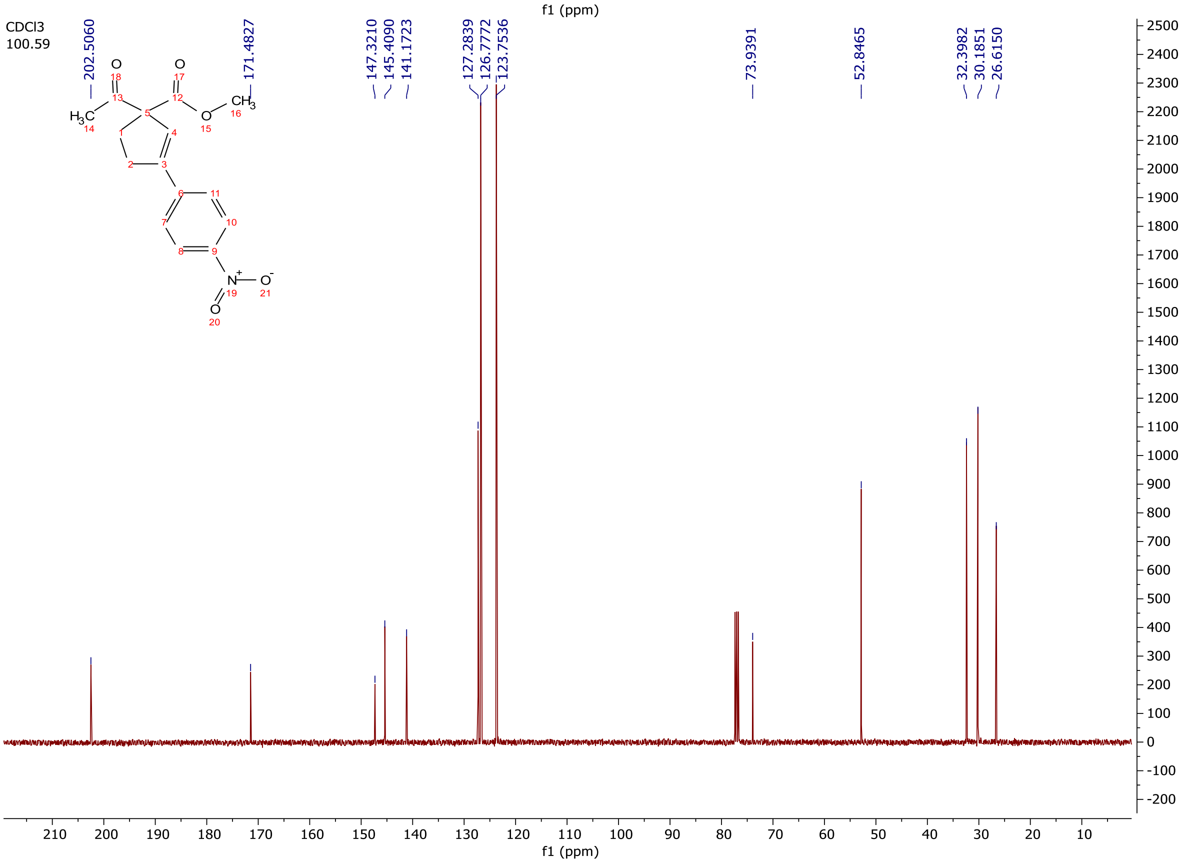

(18) methyl 2-(3-acetyl-3-(methoxycarbonyl)cyclopent-1-en-1-yl)benzoate

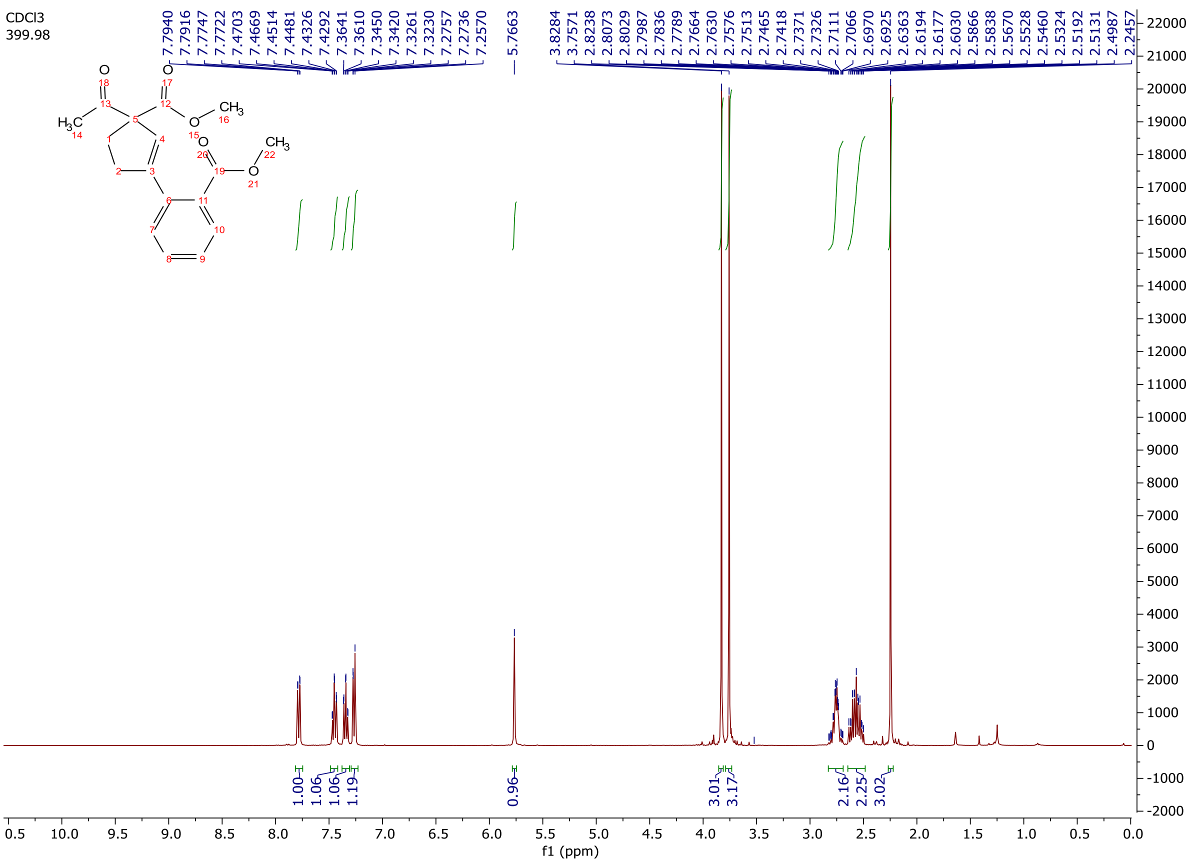

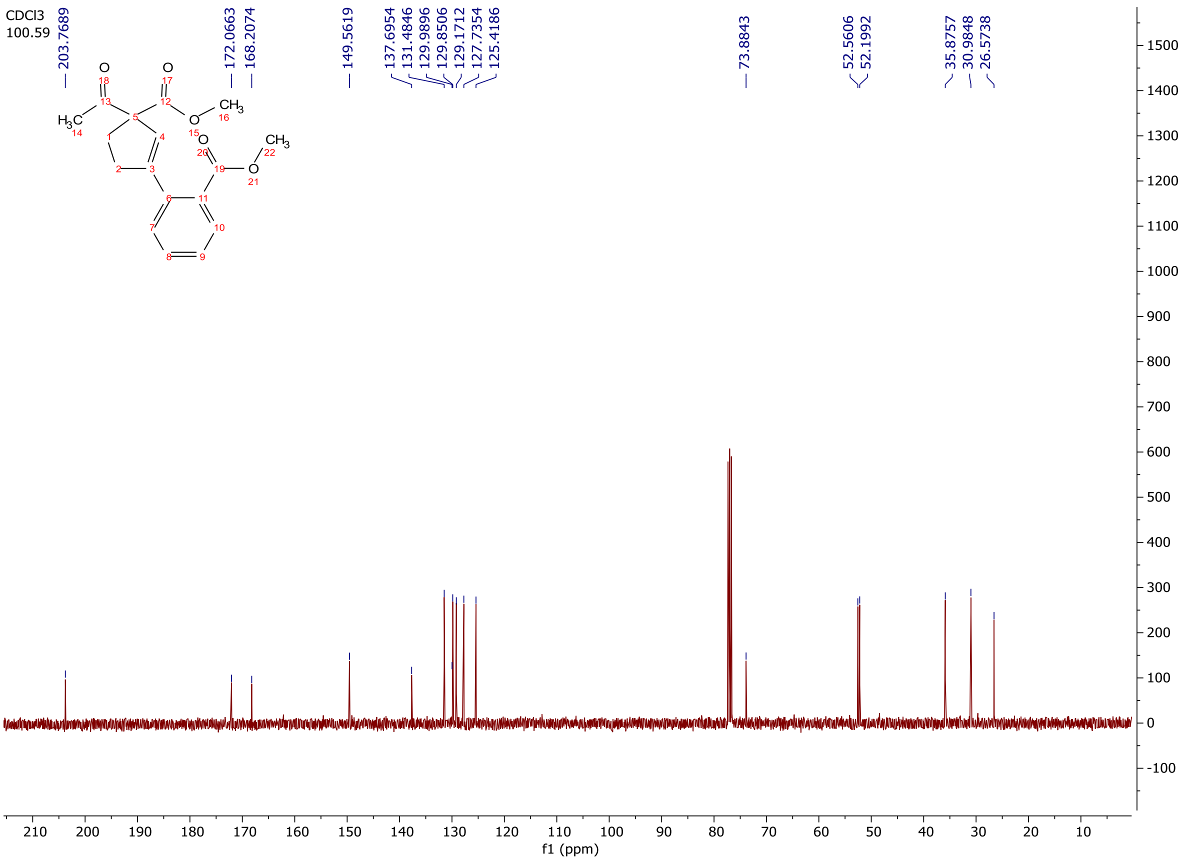

(19) methyl 1-acetyl-3-(pyridin-3-yl)cyclopent-2-ene-1-carboxylate

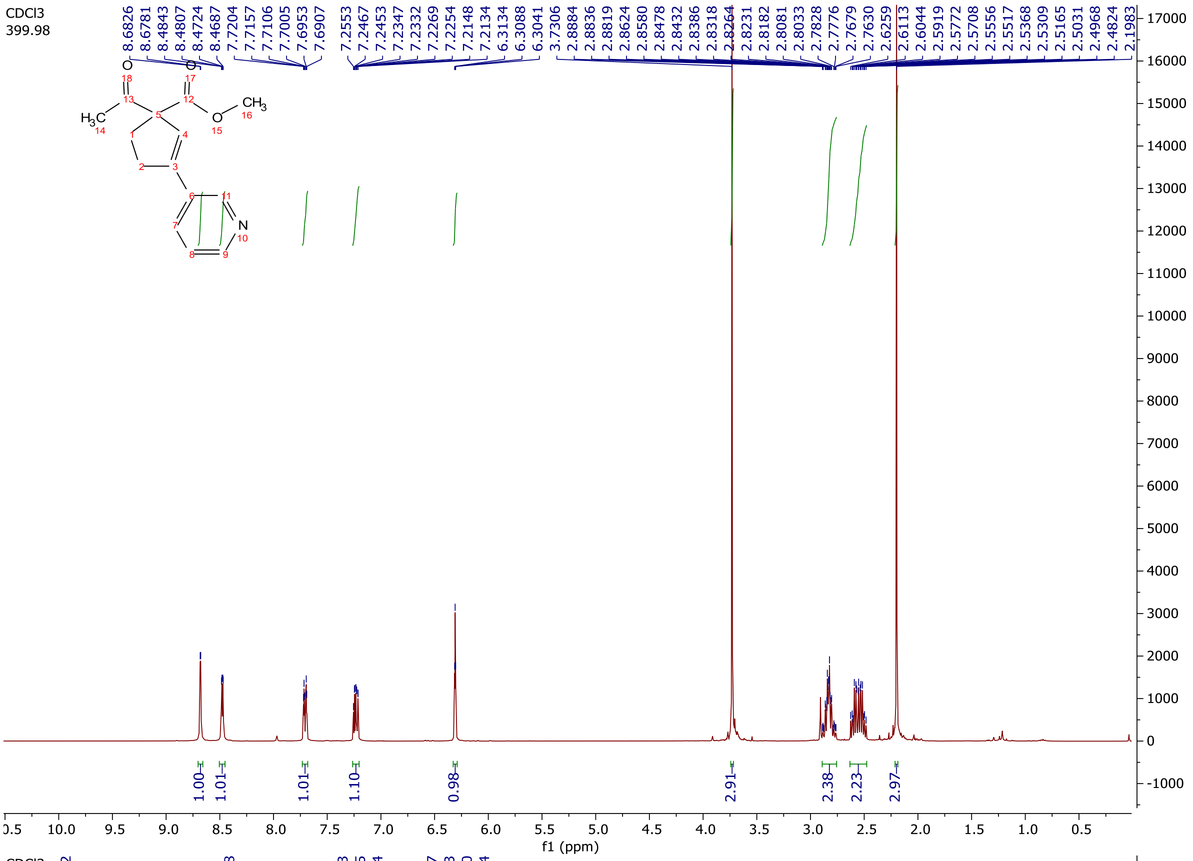

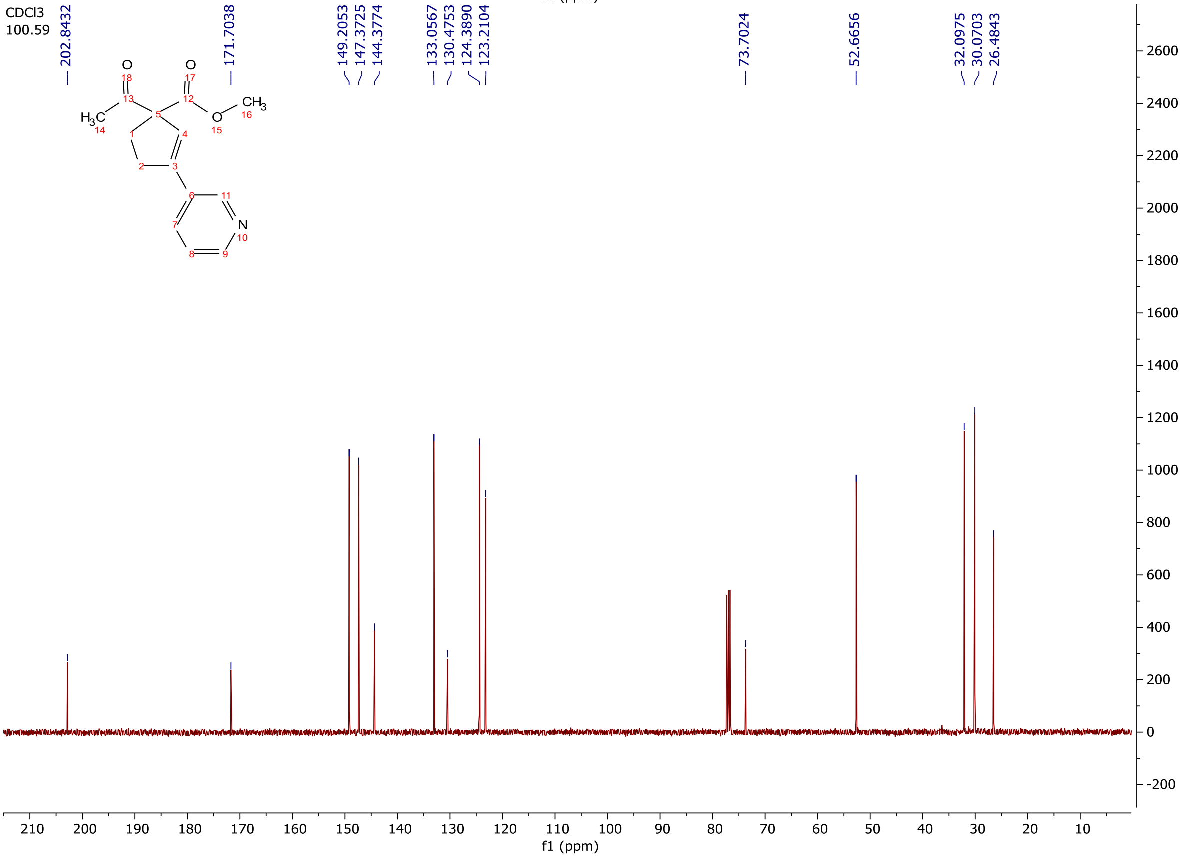

(20) methyl 1-acetyl-3-(pyrimidin-5-yl)cyclopent-2-ene-1-carboxylate

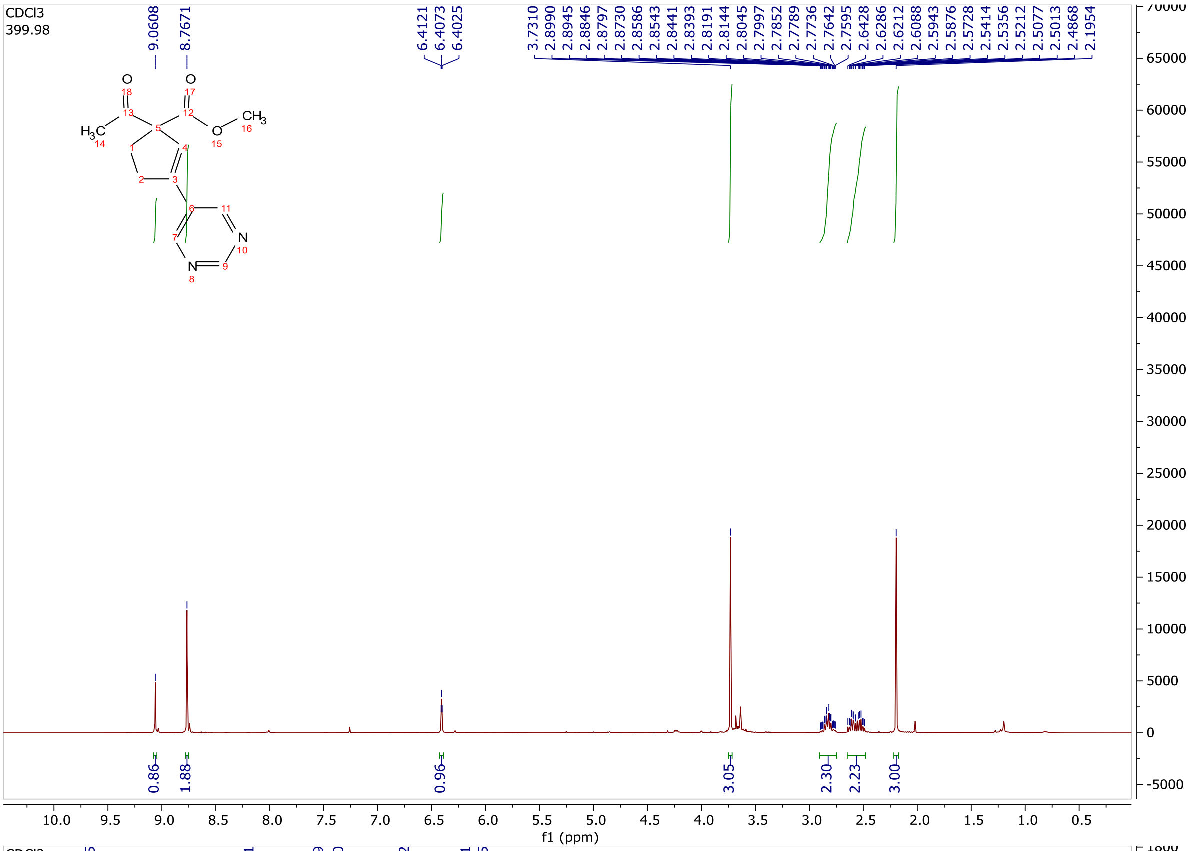

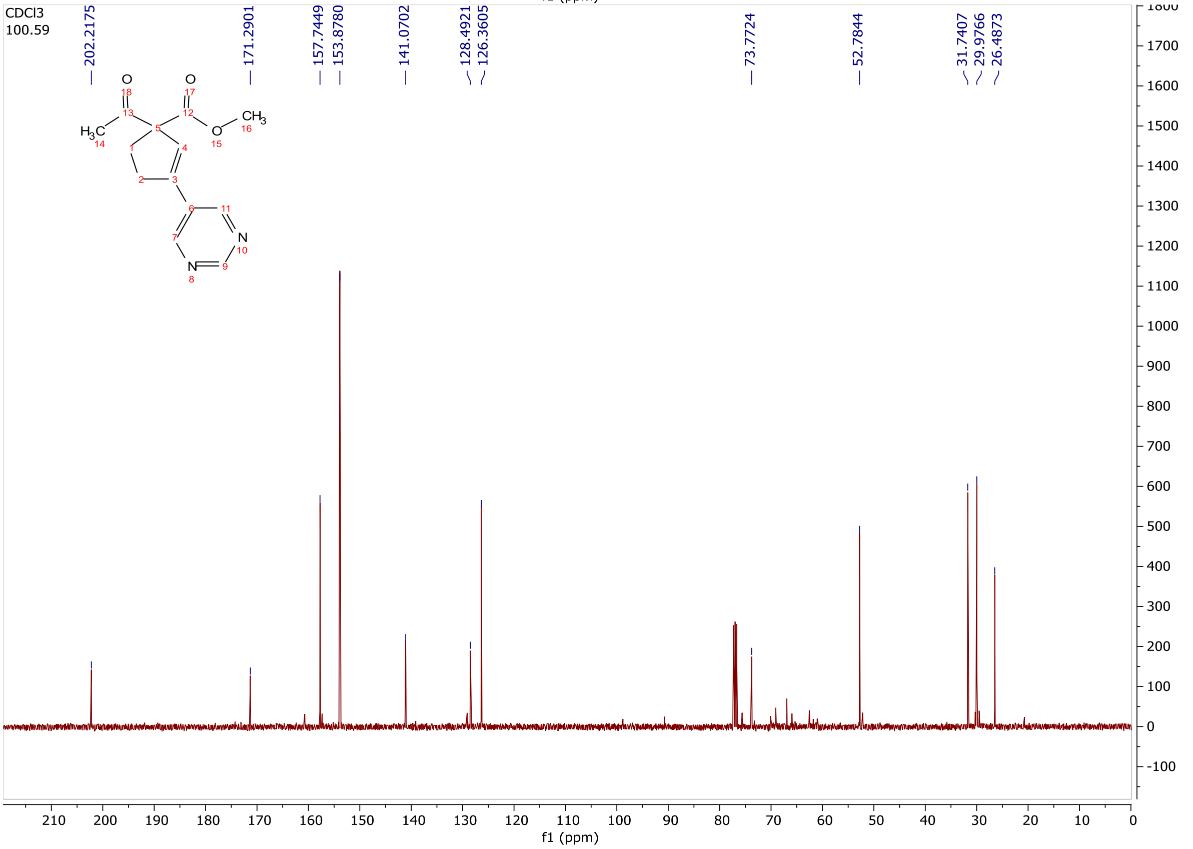

(21) methyl 1-acetyl-3-(naphthalen-1-yl)cyclopent-2-ene-1-carboxylate

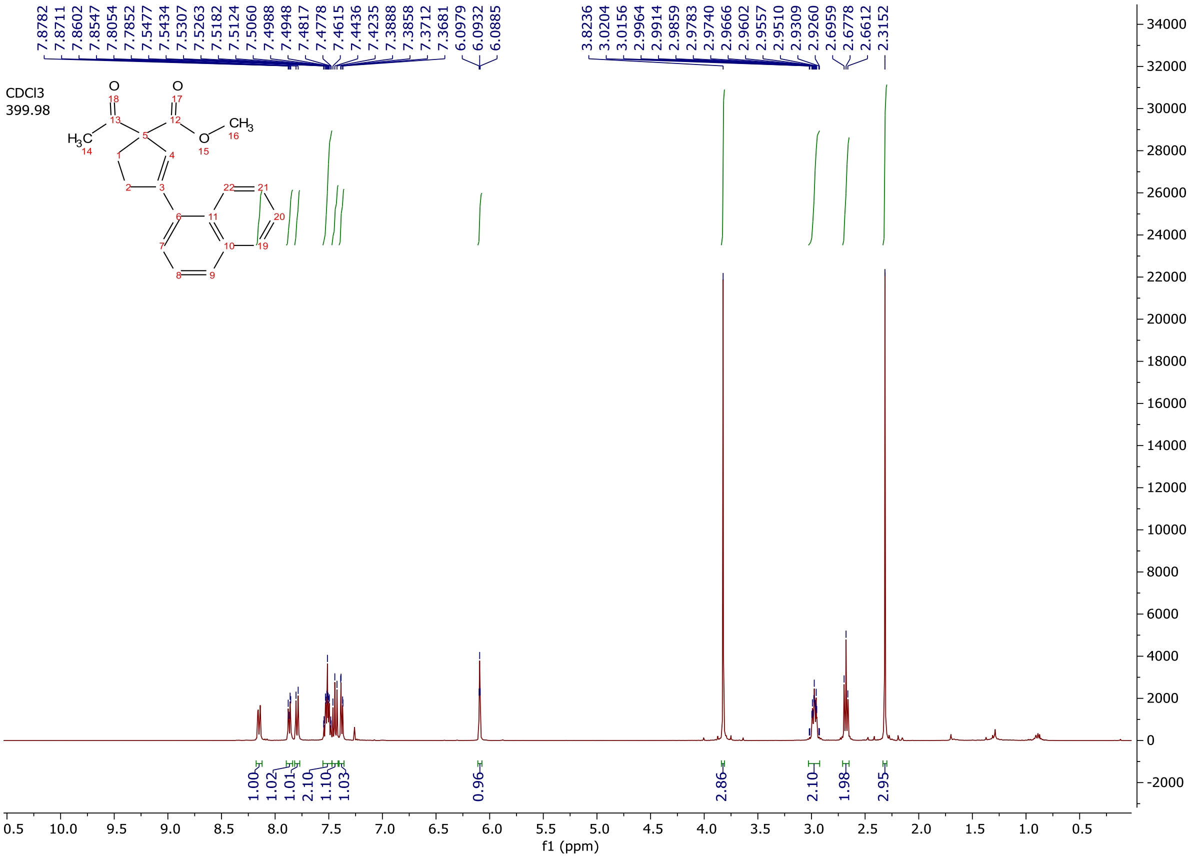

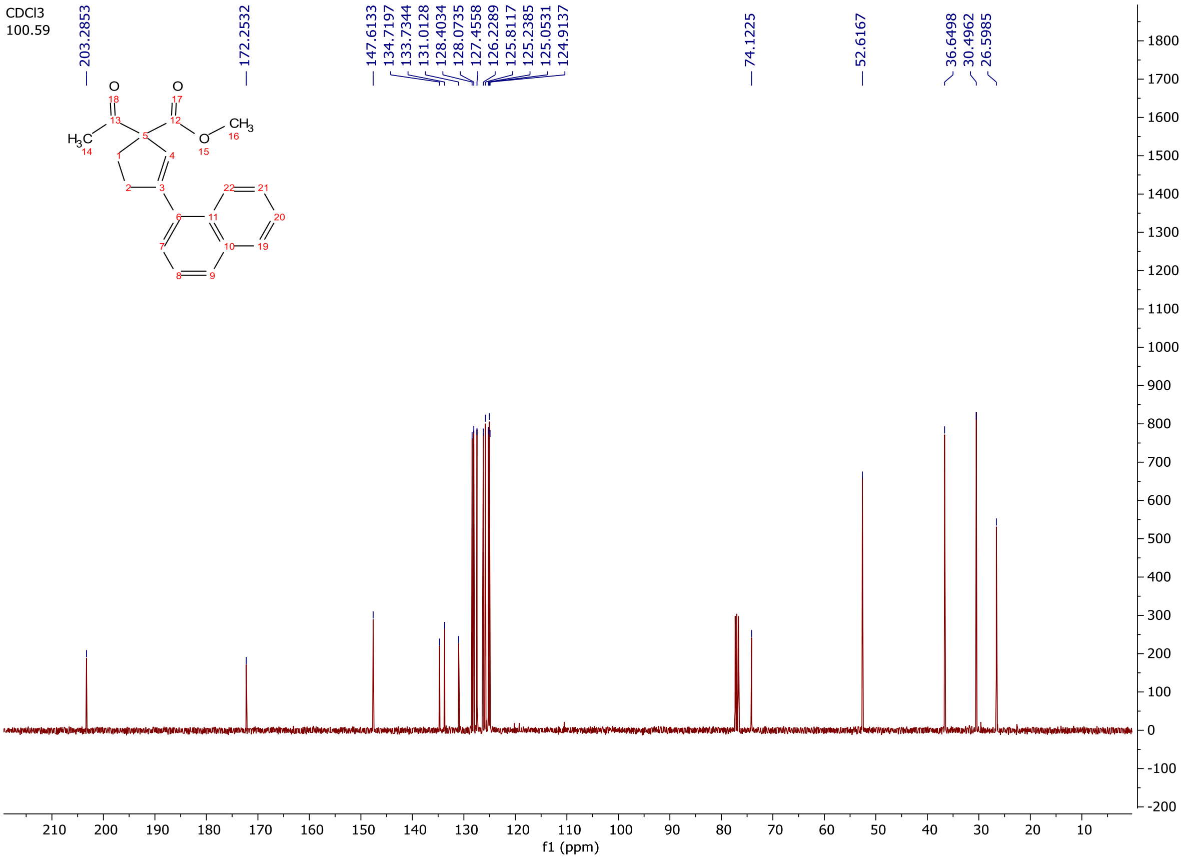

(22) methyl 1-acetyl-3-(naphthalen-2-yl)cyclopent-2-ene-1-carboxylate

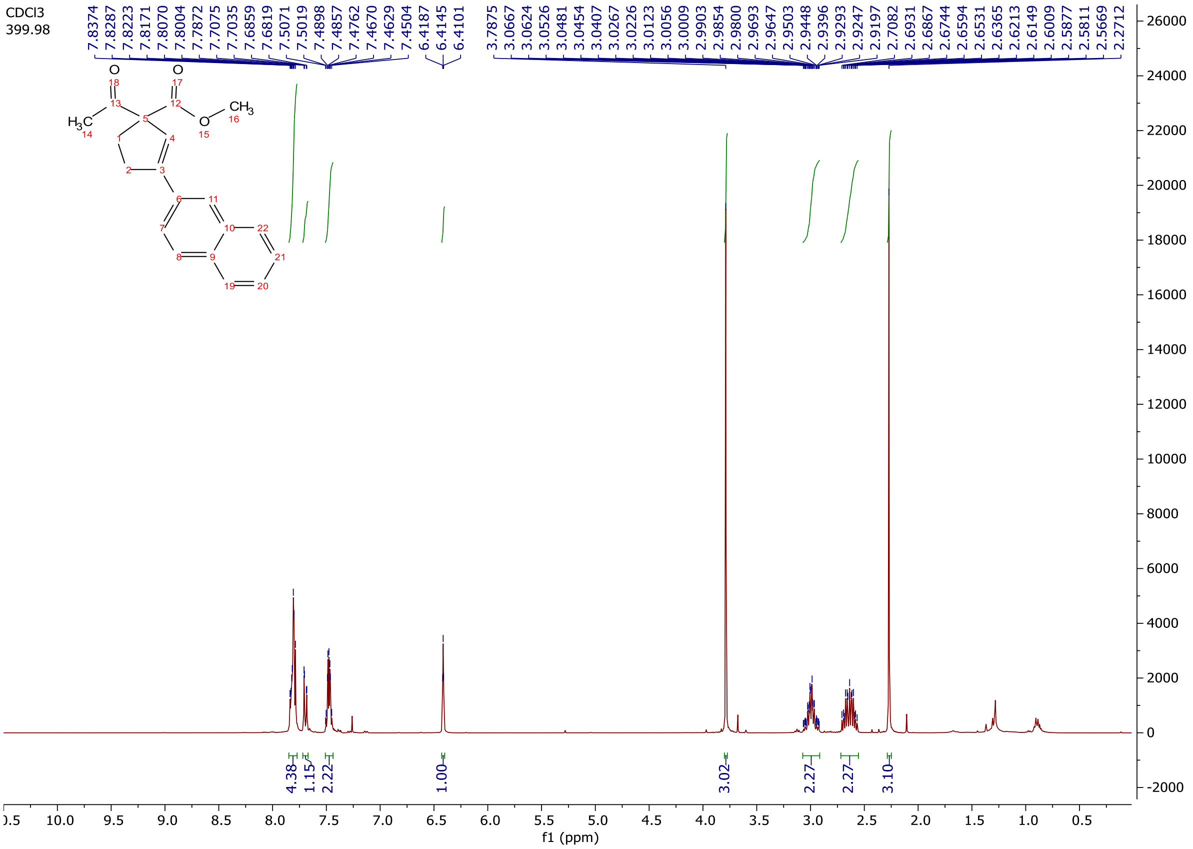

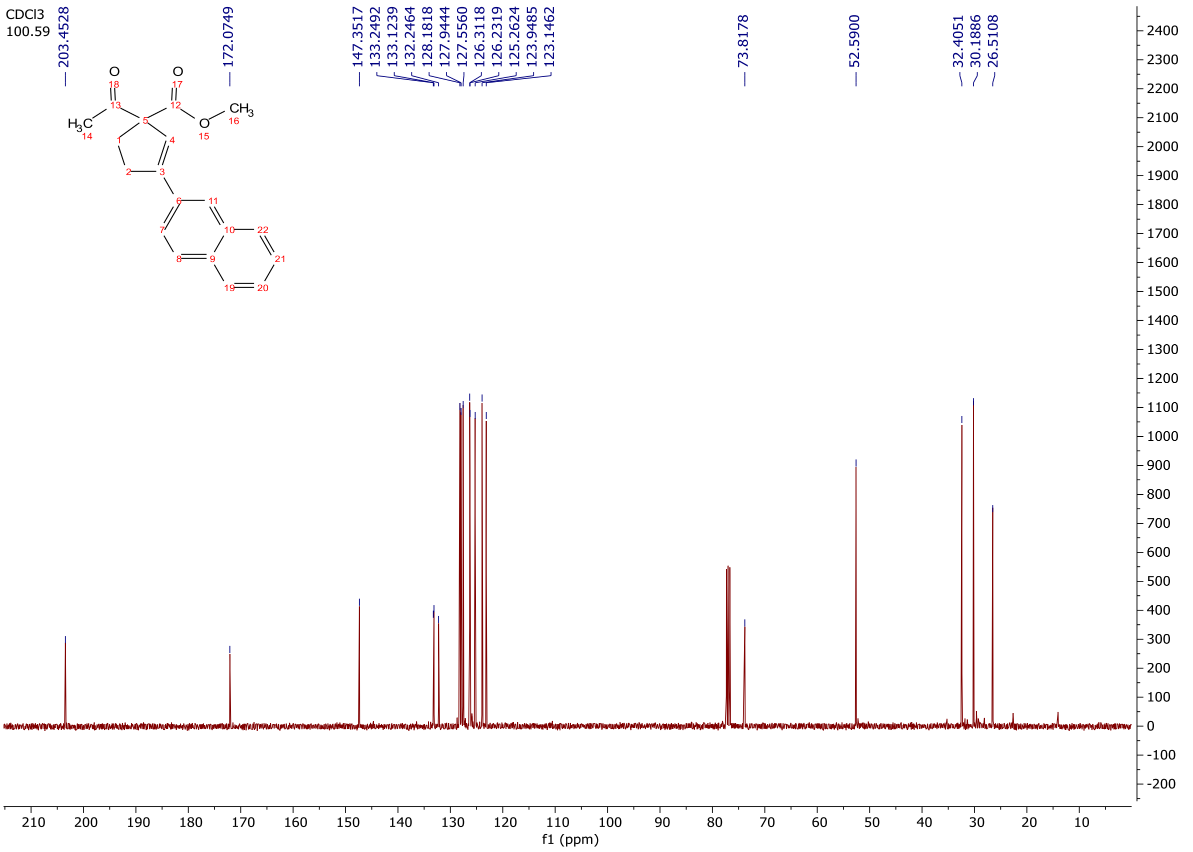

(23) methyl 1-acetyl-3-(phenanthren-9-yl)cyclopent-2-ene-1-carboxylate

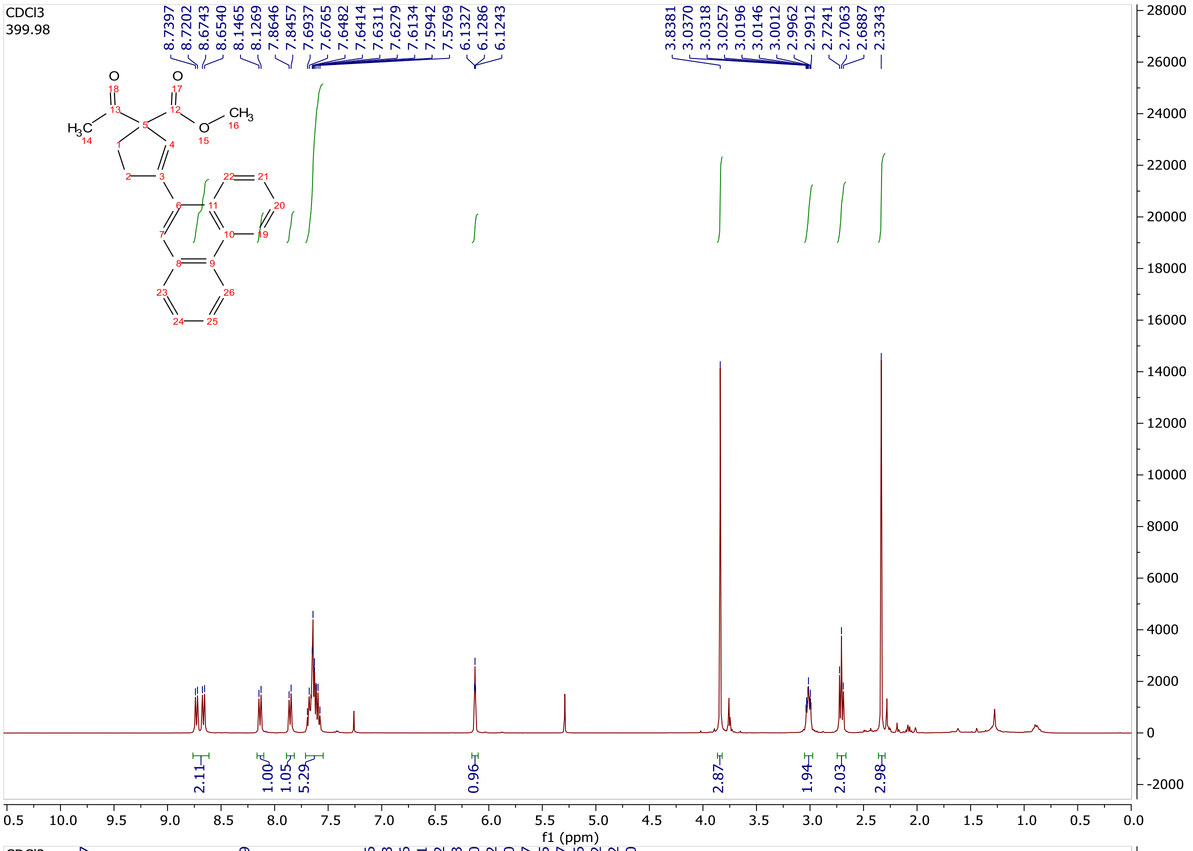

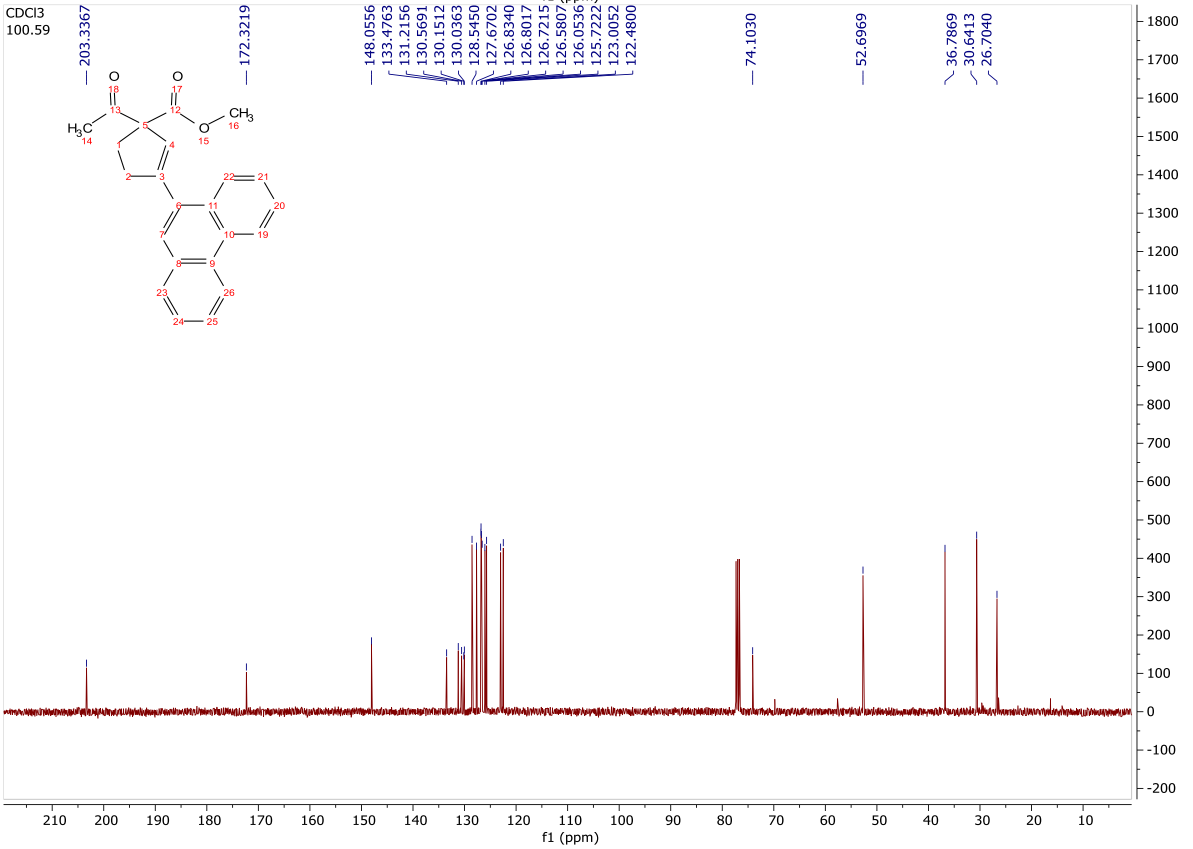

(24) methyl 1-acetyl-3-(benzo[d][1,3]dioxol-5-yl)cyclopent-2-ene-1-carboxylate

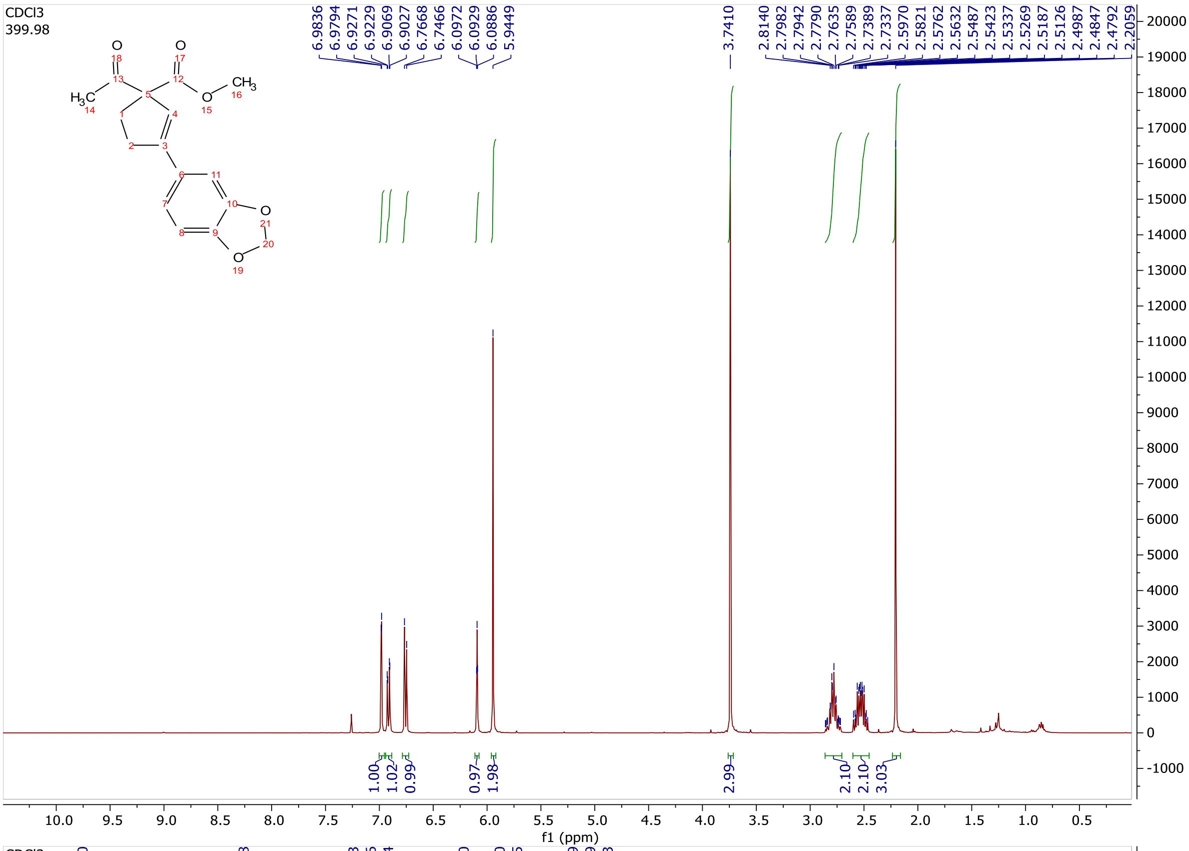

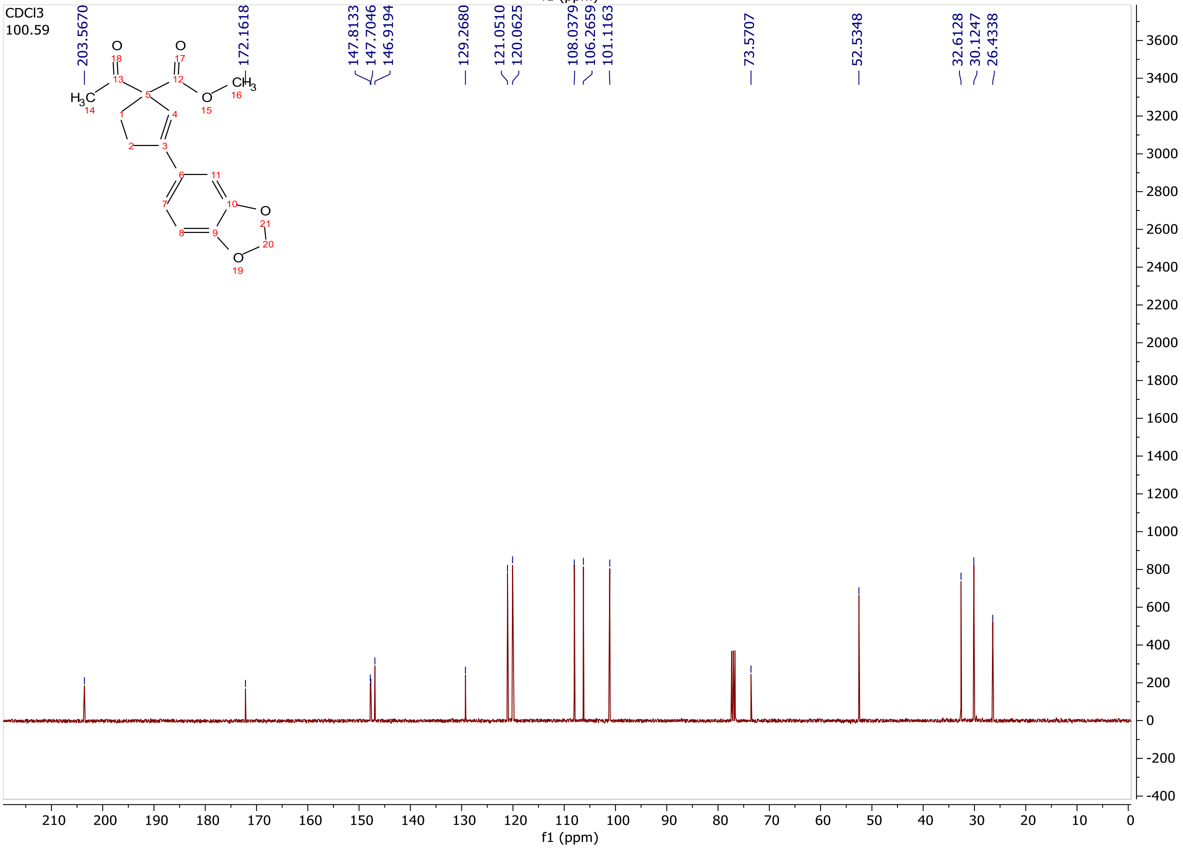

(25) methyl 1-acetyl-3-(benzo[d][1,3]dioxol-5-yl)cyclopent-2-ene-1-carboxylate

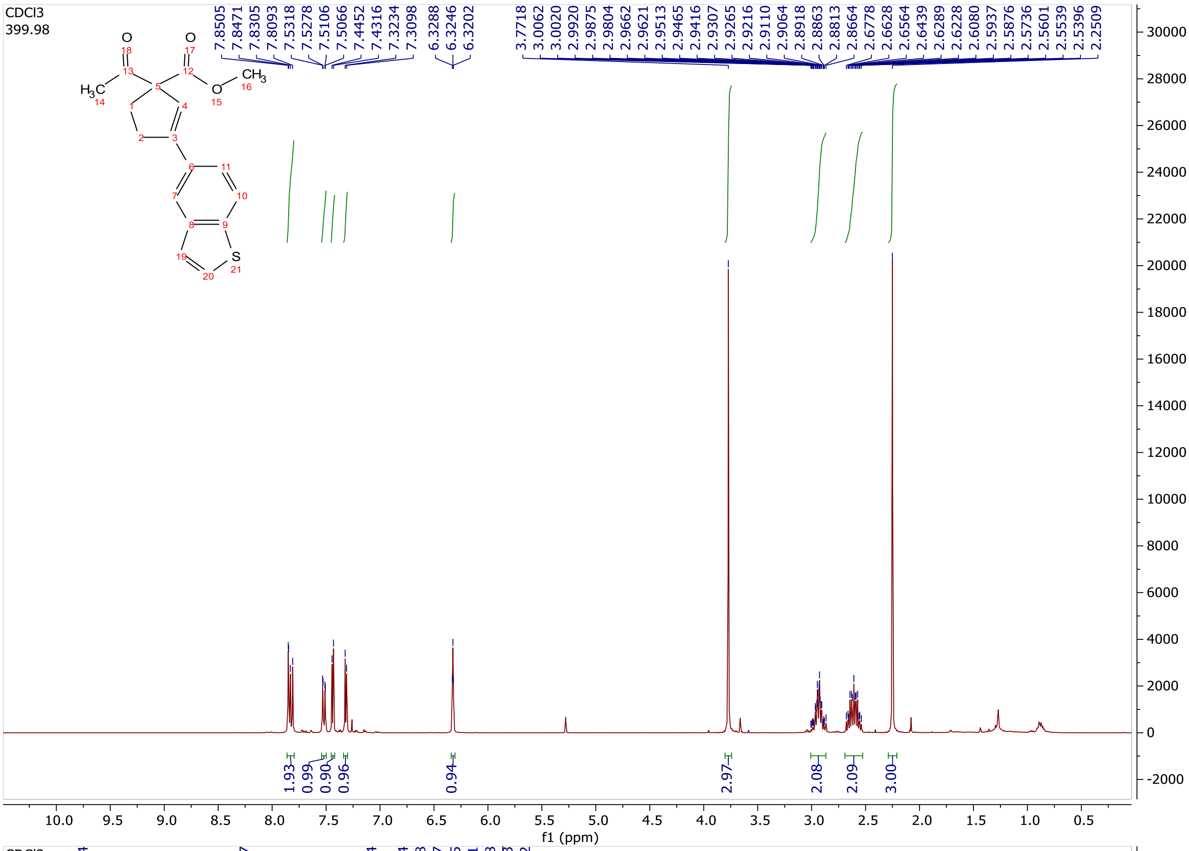

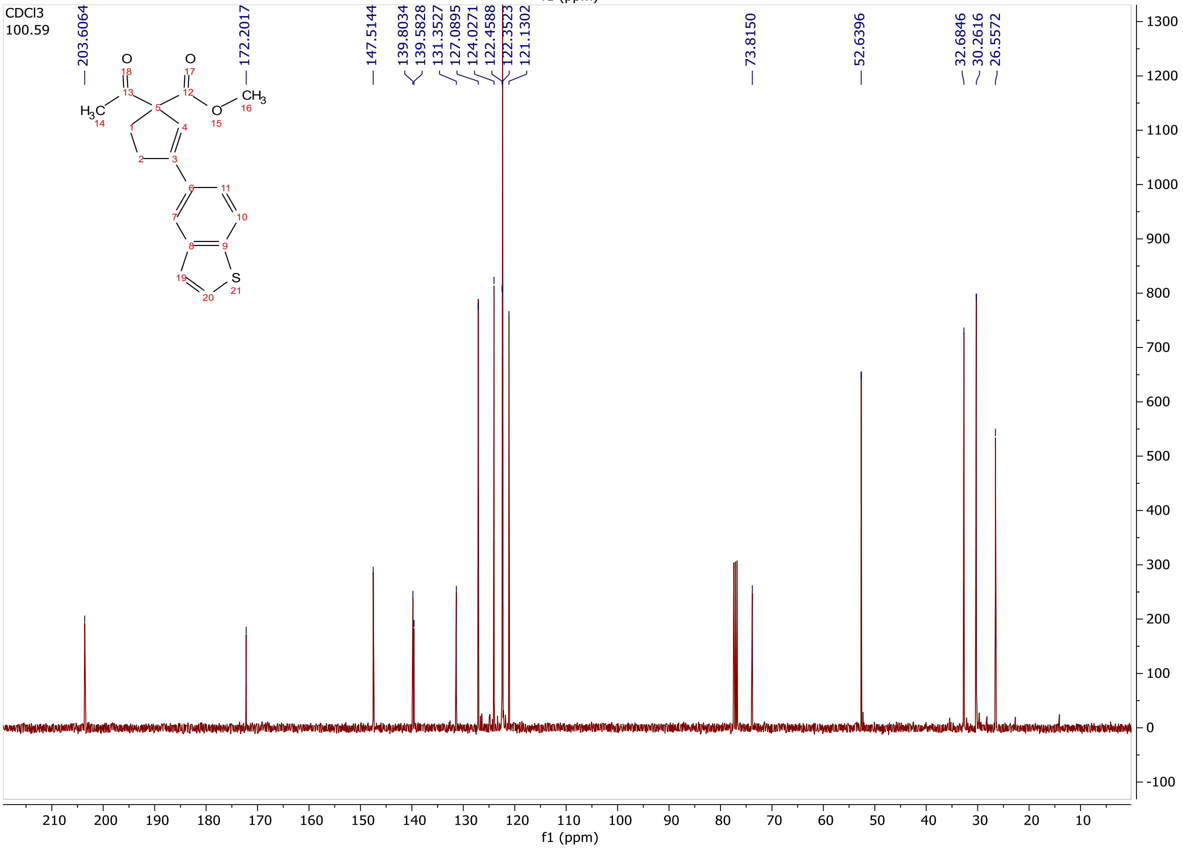

(26) methyl 1-acetyl-3-(2-methylbenzo[d]oxazol-5-yl)cyclopent-2-ene-1-carboxylate

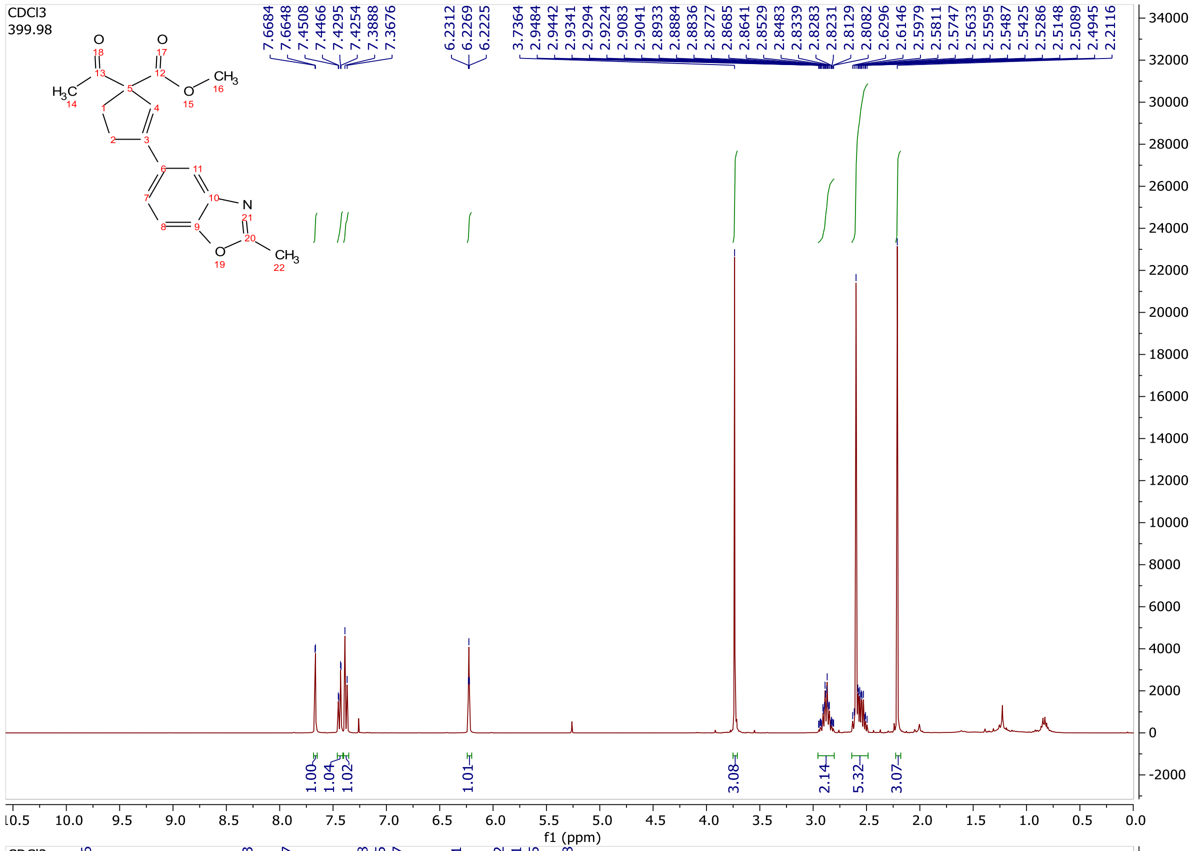

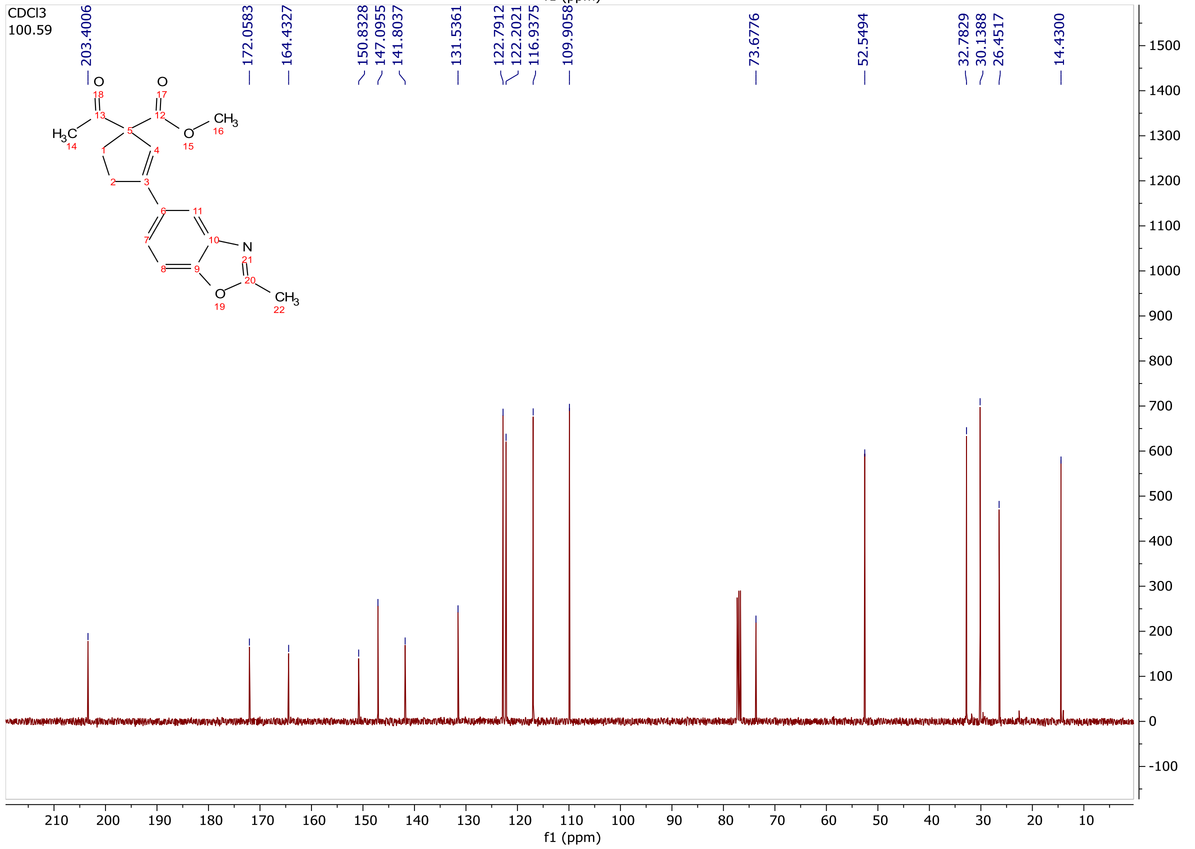

(27) methyl 1-acetyl-3-(2-methylbenzo[d]oxazol-5-yl)cyclopent-2-ene-1-carboxylate

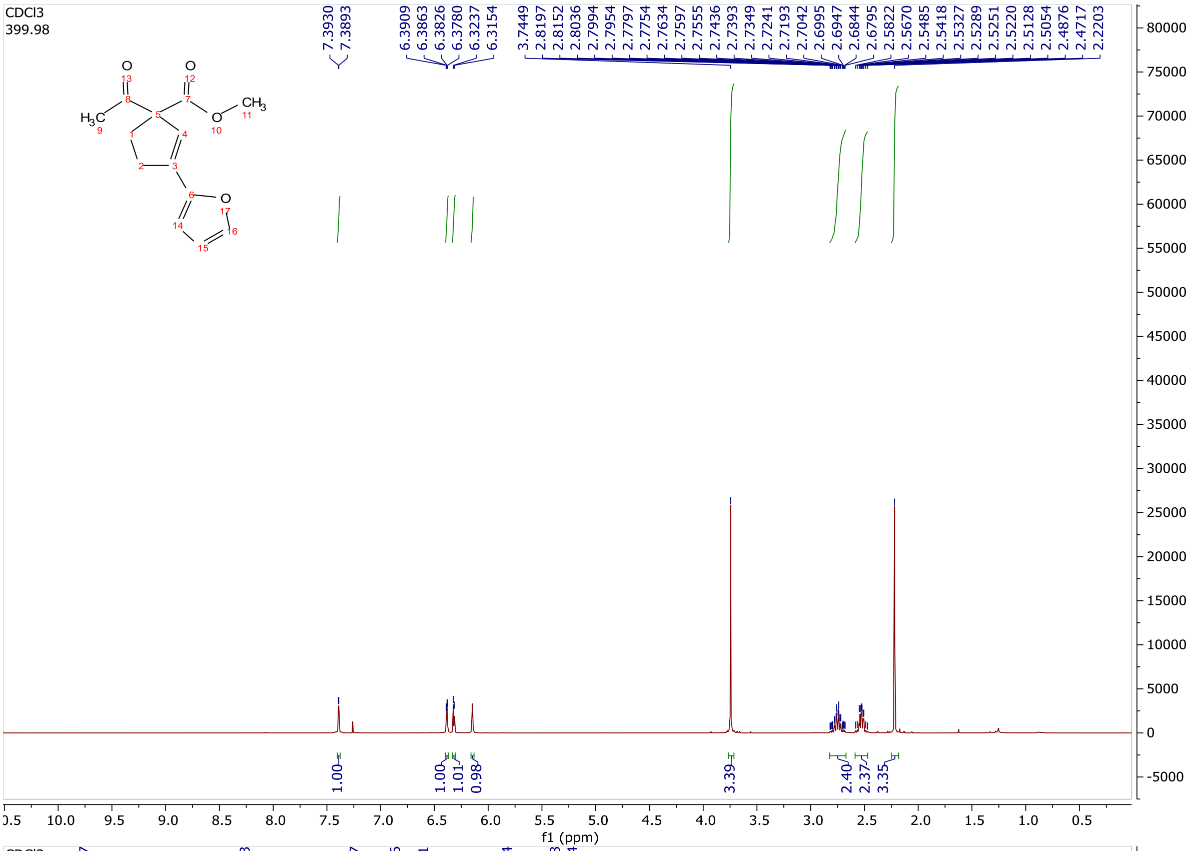

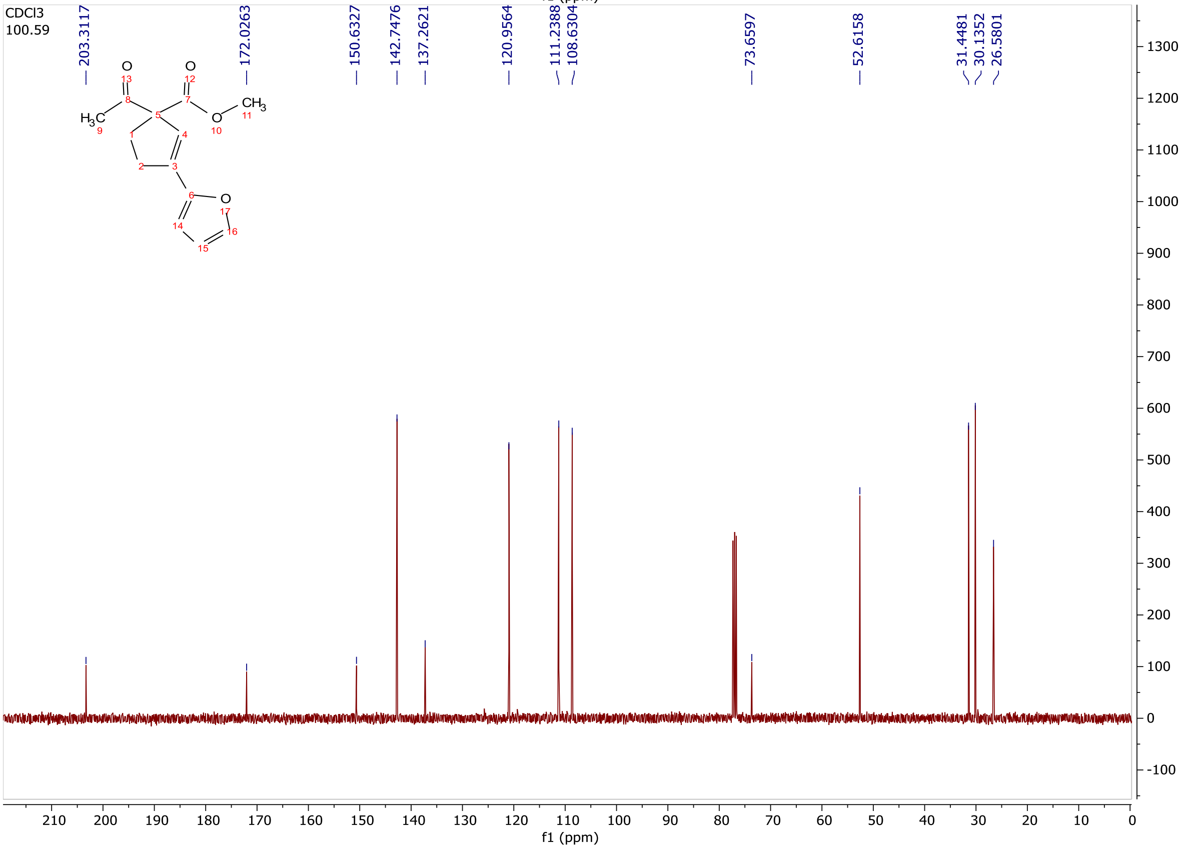

(28) methyl 1-acetyl-3-(furan-3-yl)cyclopent-2-ene-1-carboxylate

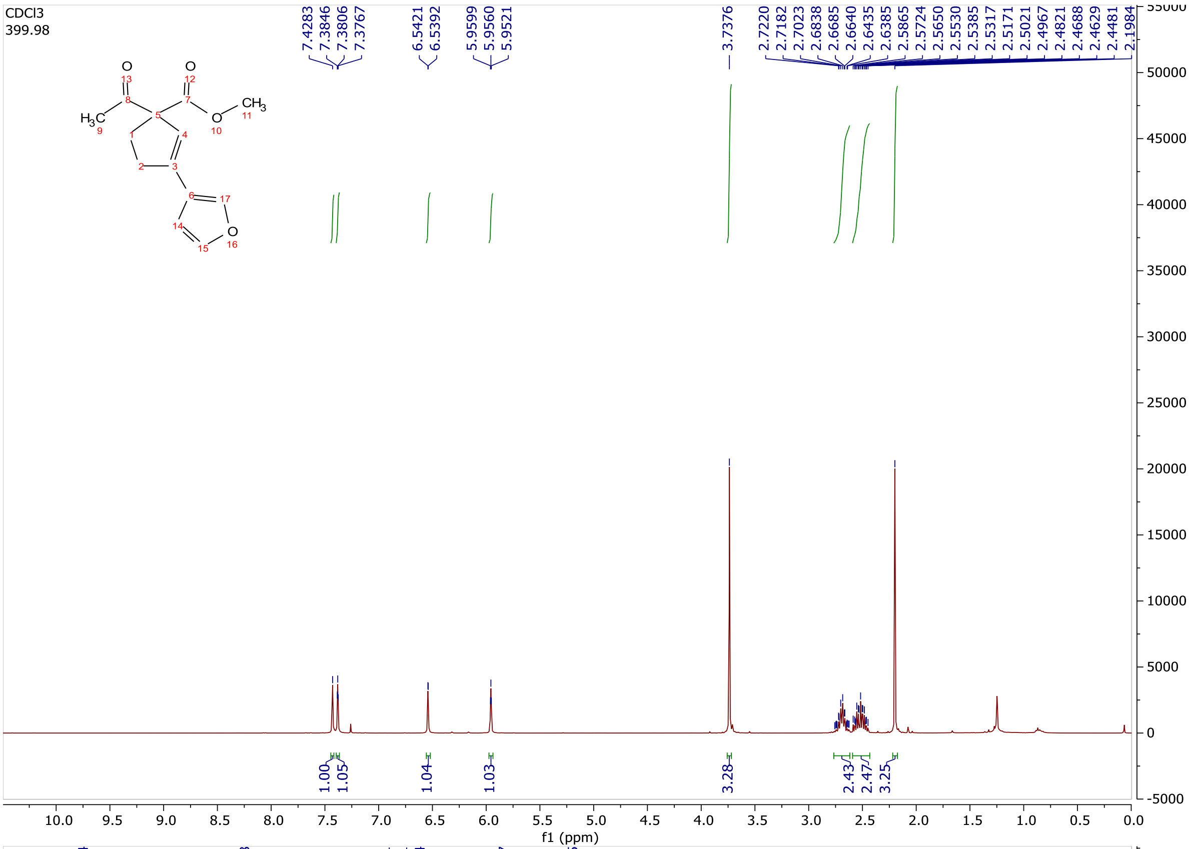

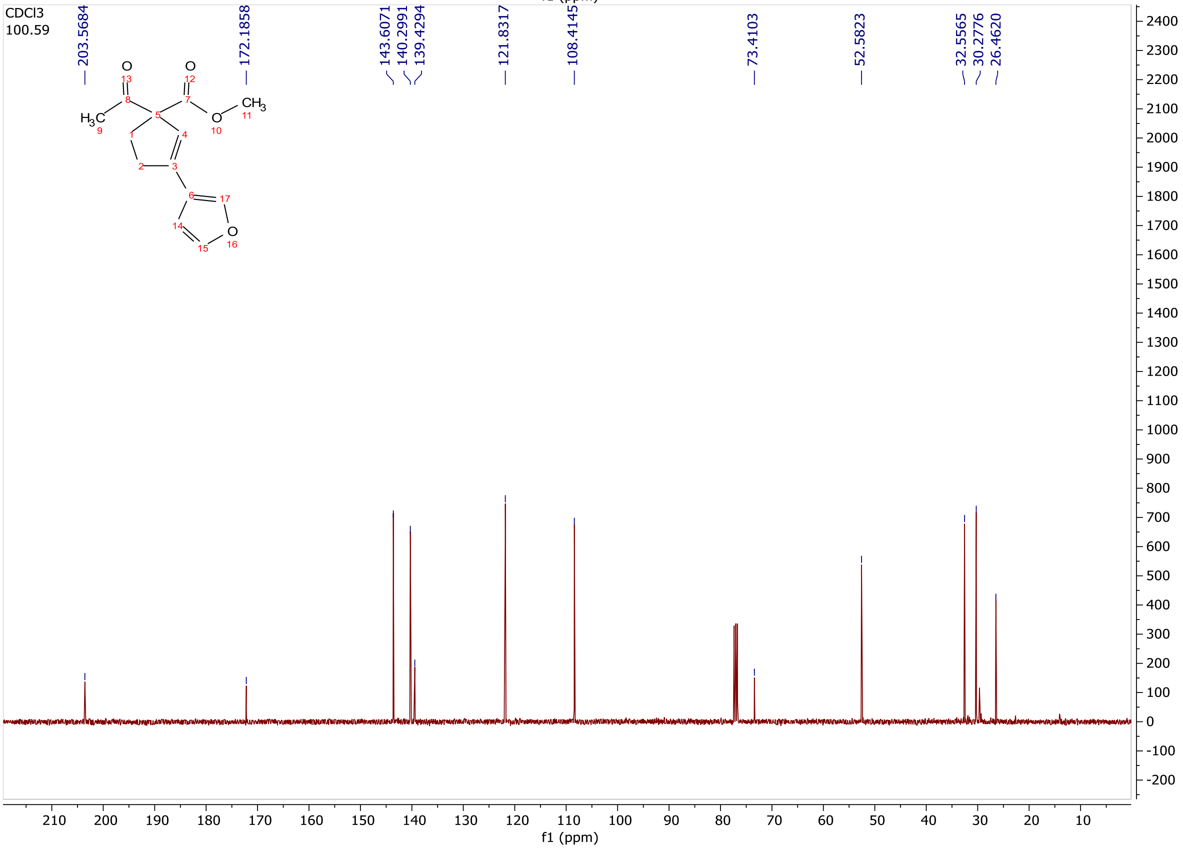

(29) methyl 1-acetyl-3-(thiophen-2-yl)cyclopent-2-ene-1-carboxylate

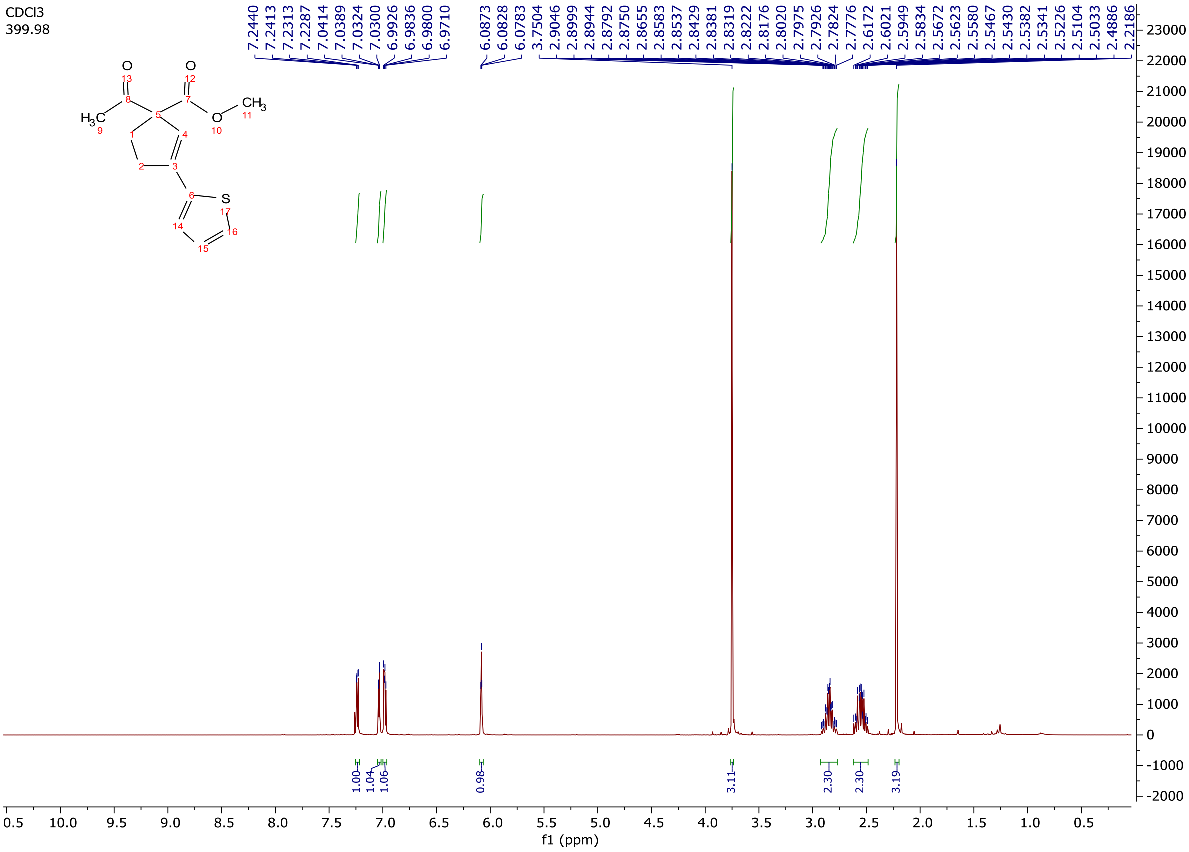

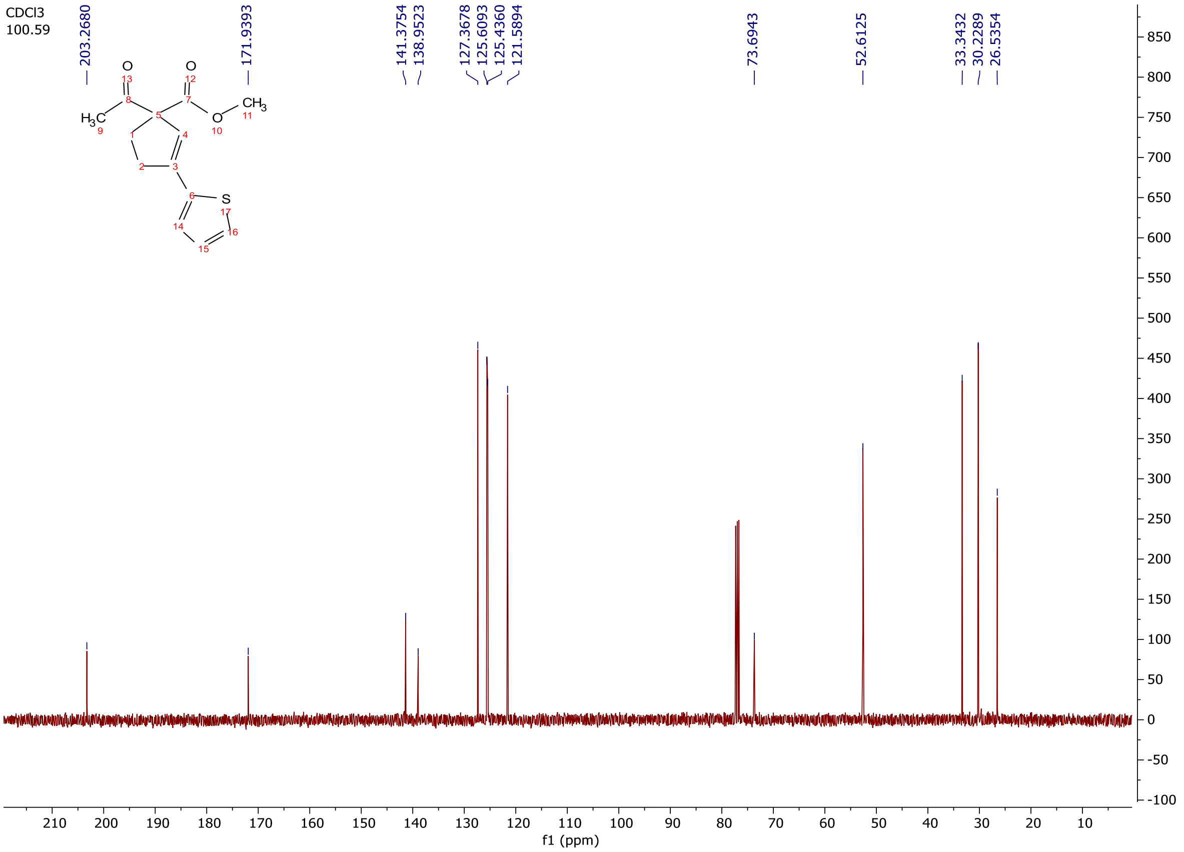

(30) methyl 1-isobutyryl-3-phenylcyclopent-2-ene-1-carboxylate

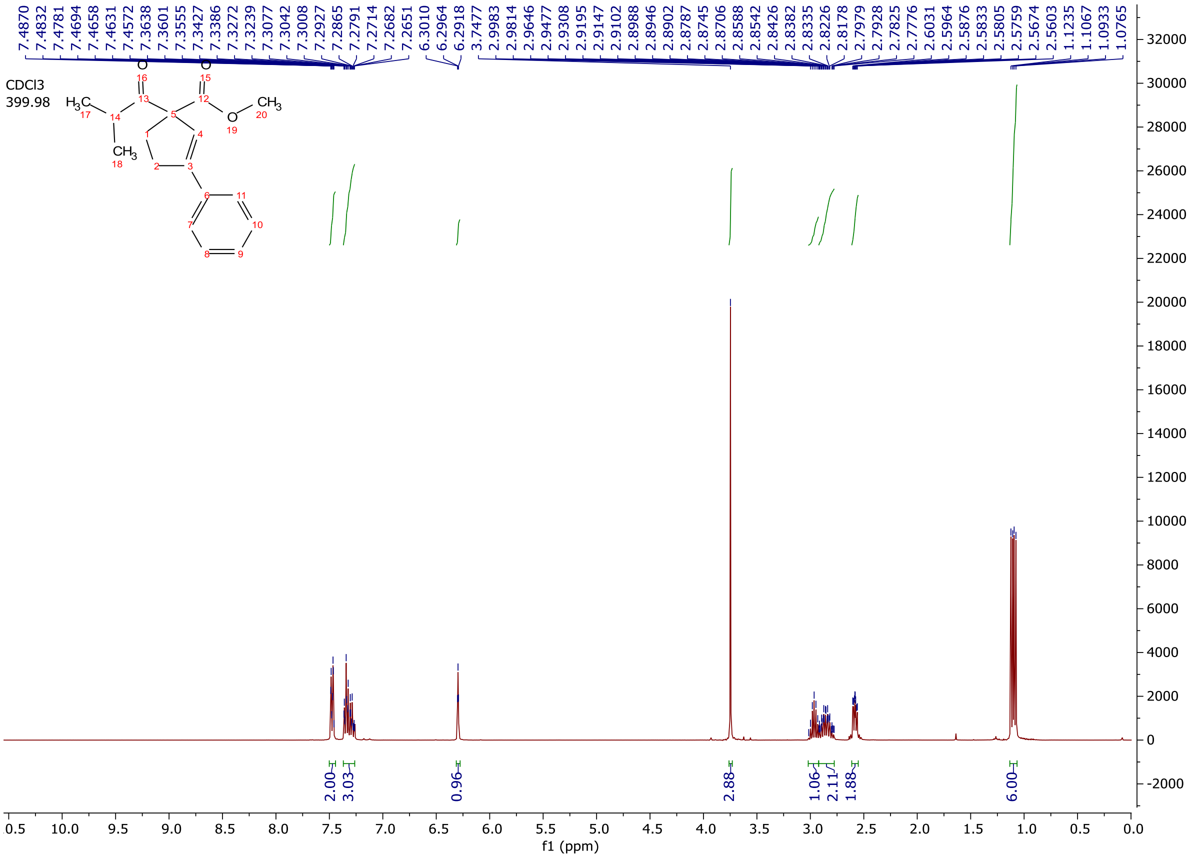

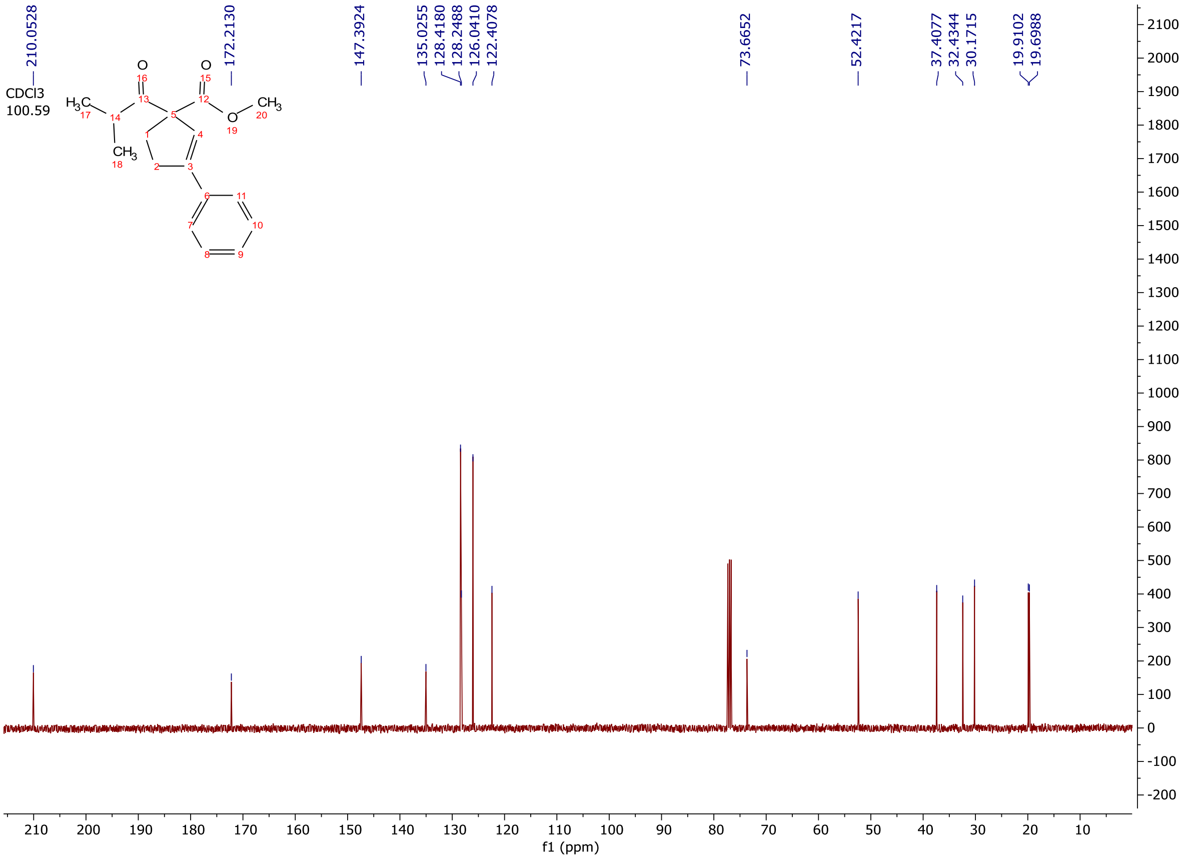

(31) ethyl 1-benzoyl-3-phenylcyclopent-2-ene-1-carboxylate

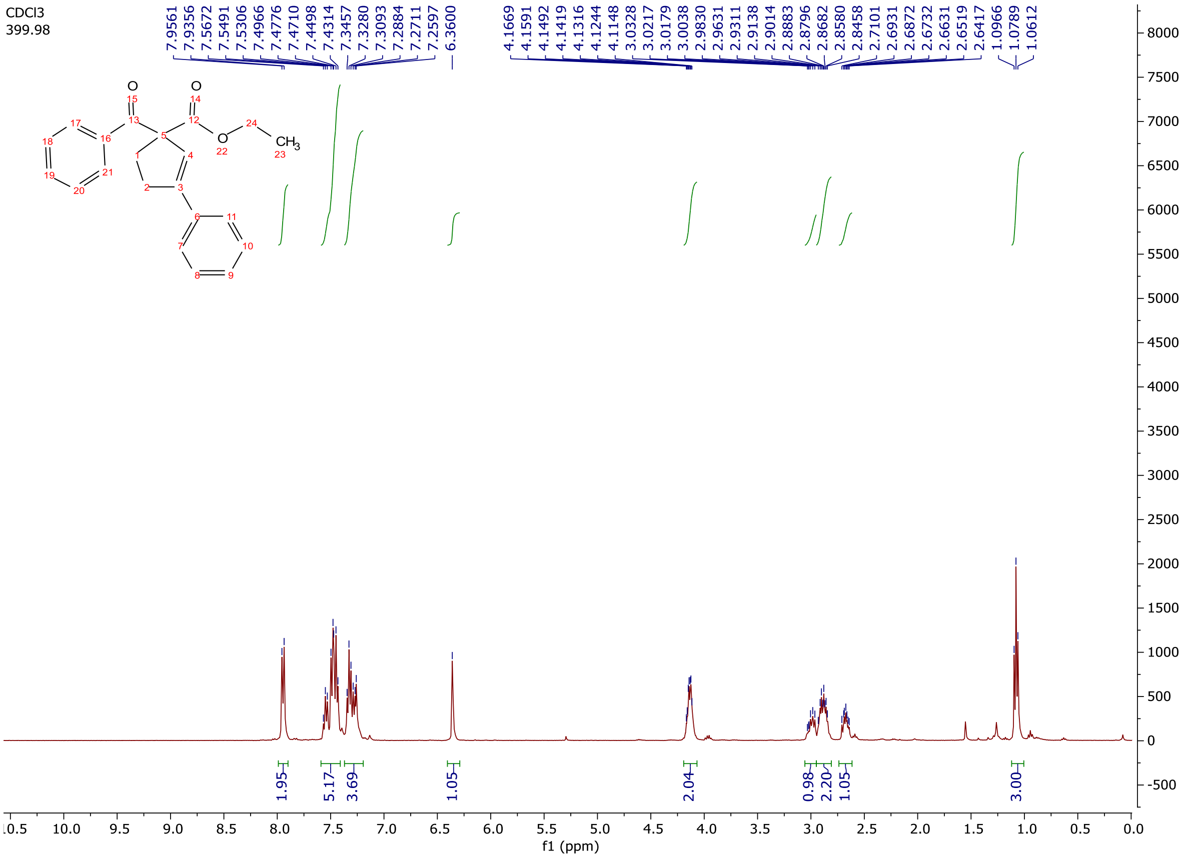

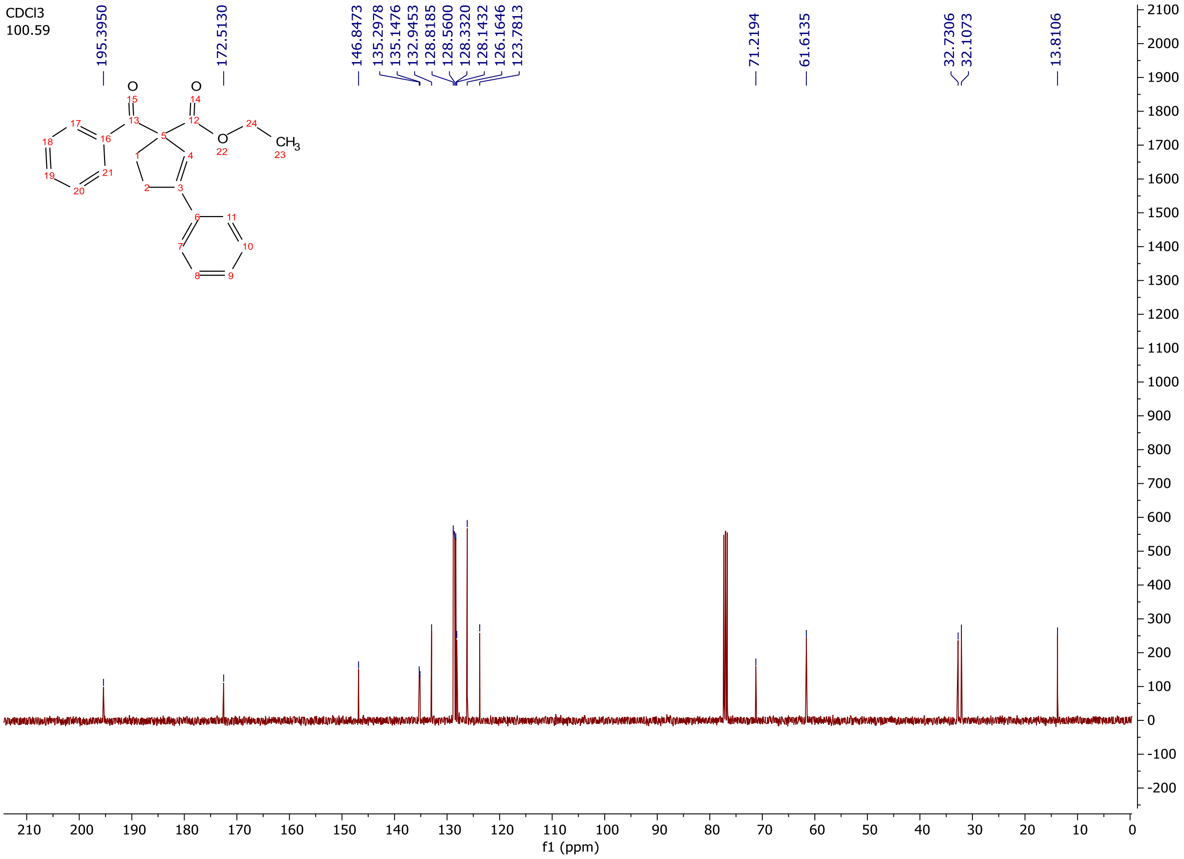

(32) isopropyl 1-acetyl-3-phenylcyclopent-2-ene-1-carboxylate

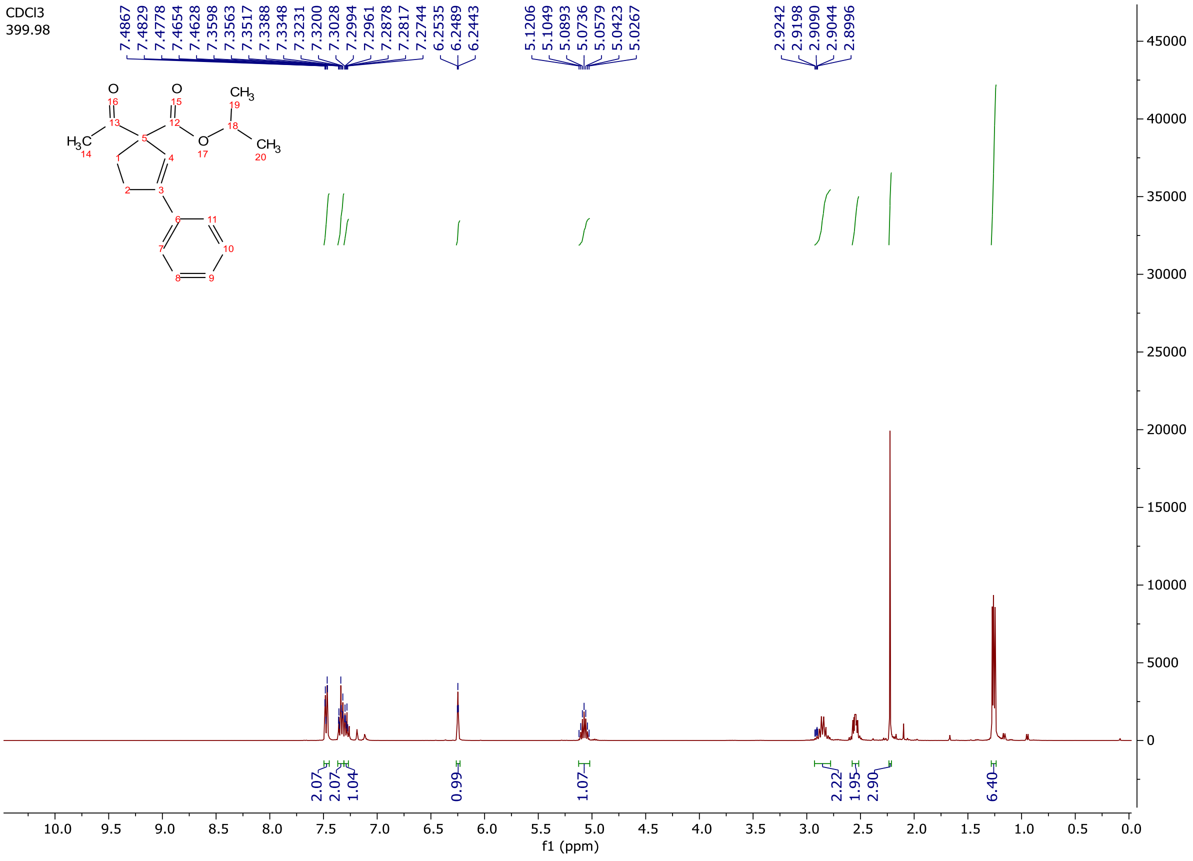

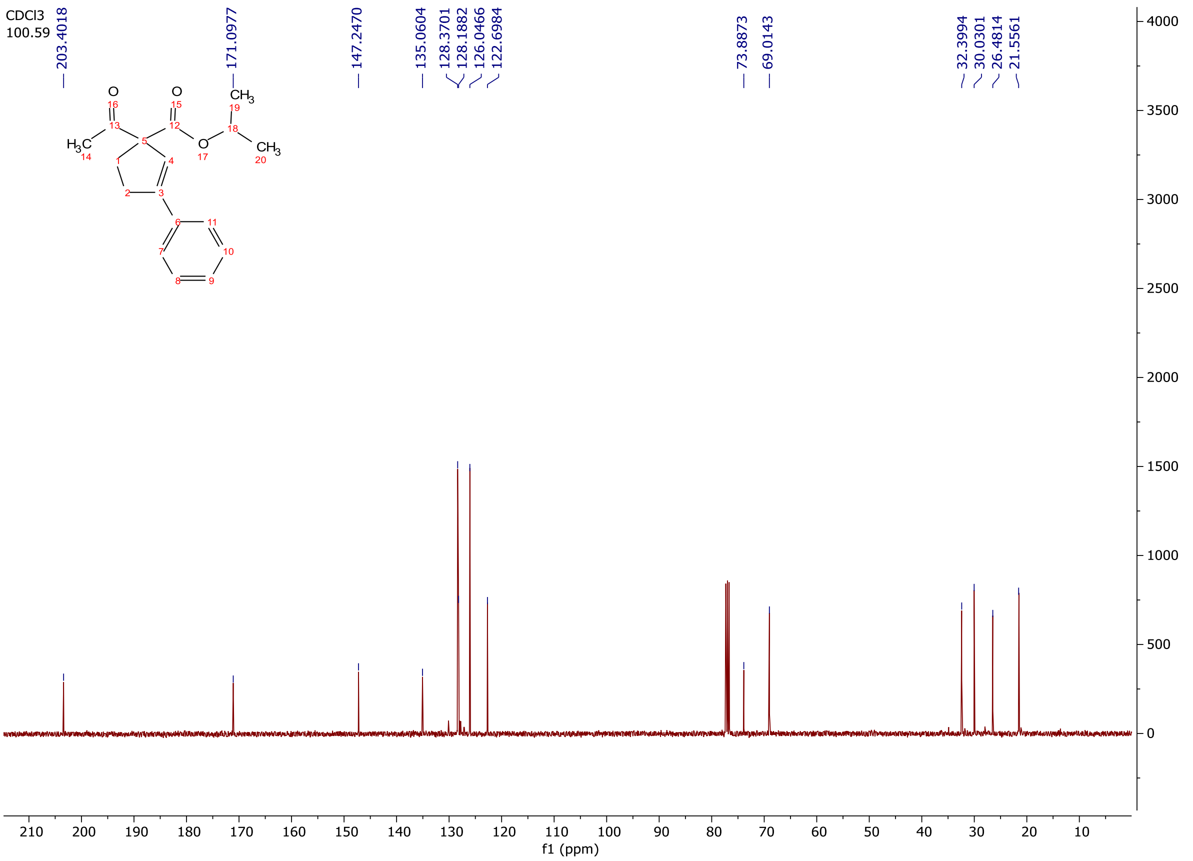

(33) tert-butyl 1-acetyl-3-phenylcyclopent-2-ene-1-carboxylate

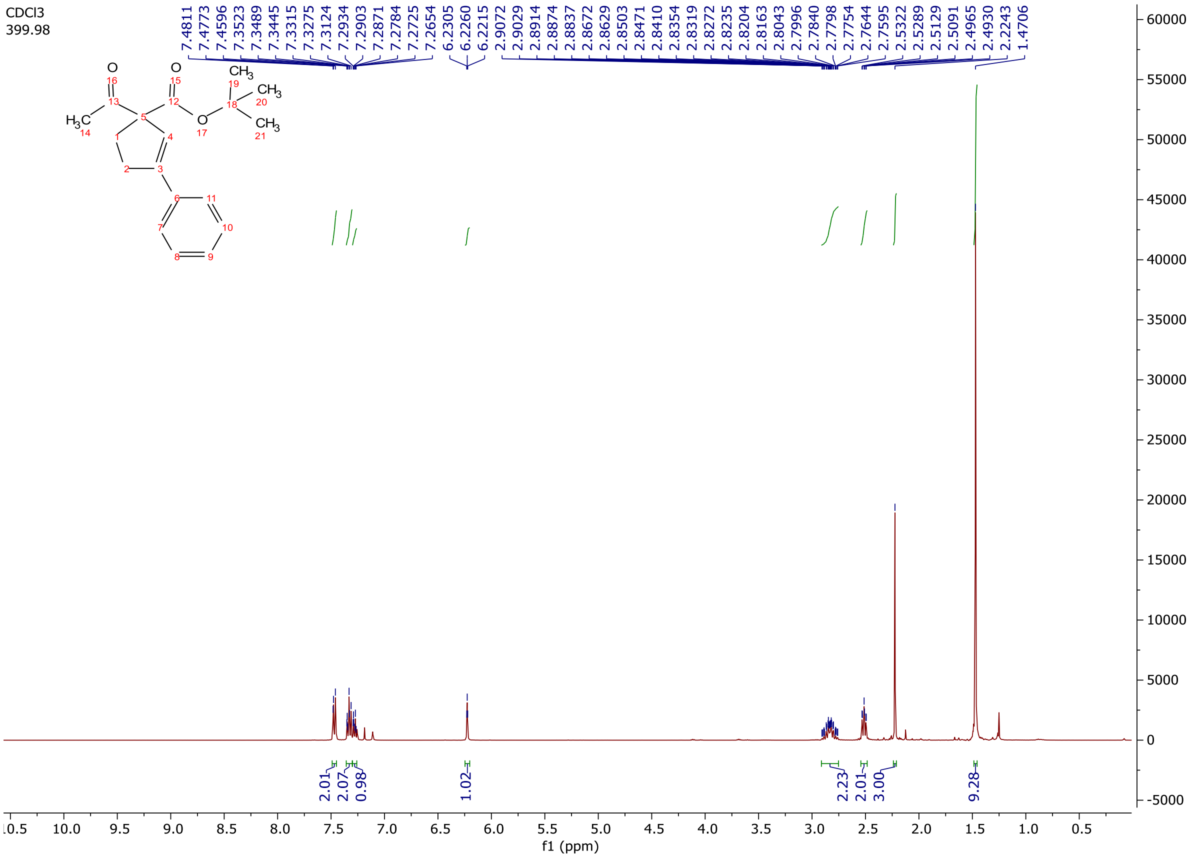

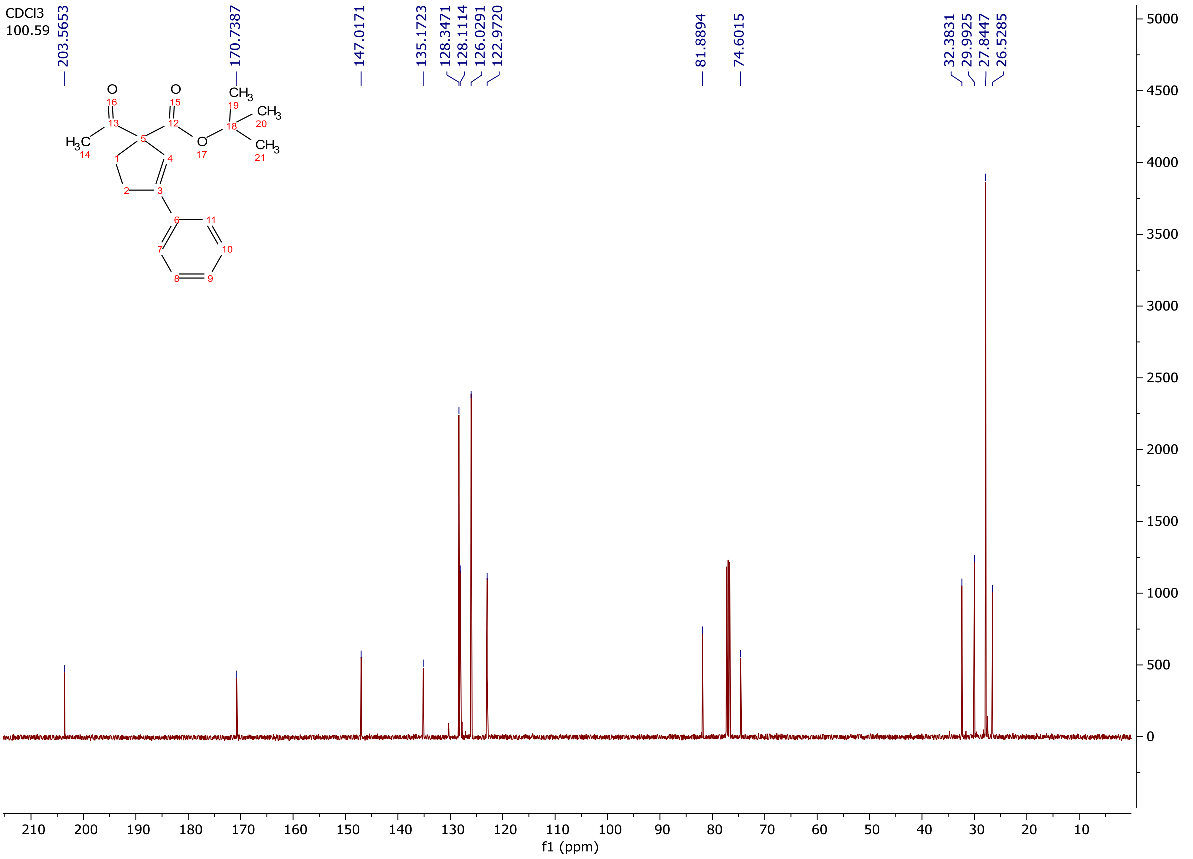

(34) 1-(1-benzoyl-3-phenylcyclopent-2-en-1-yl)ethan-1-one

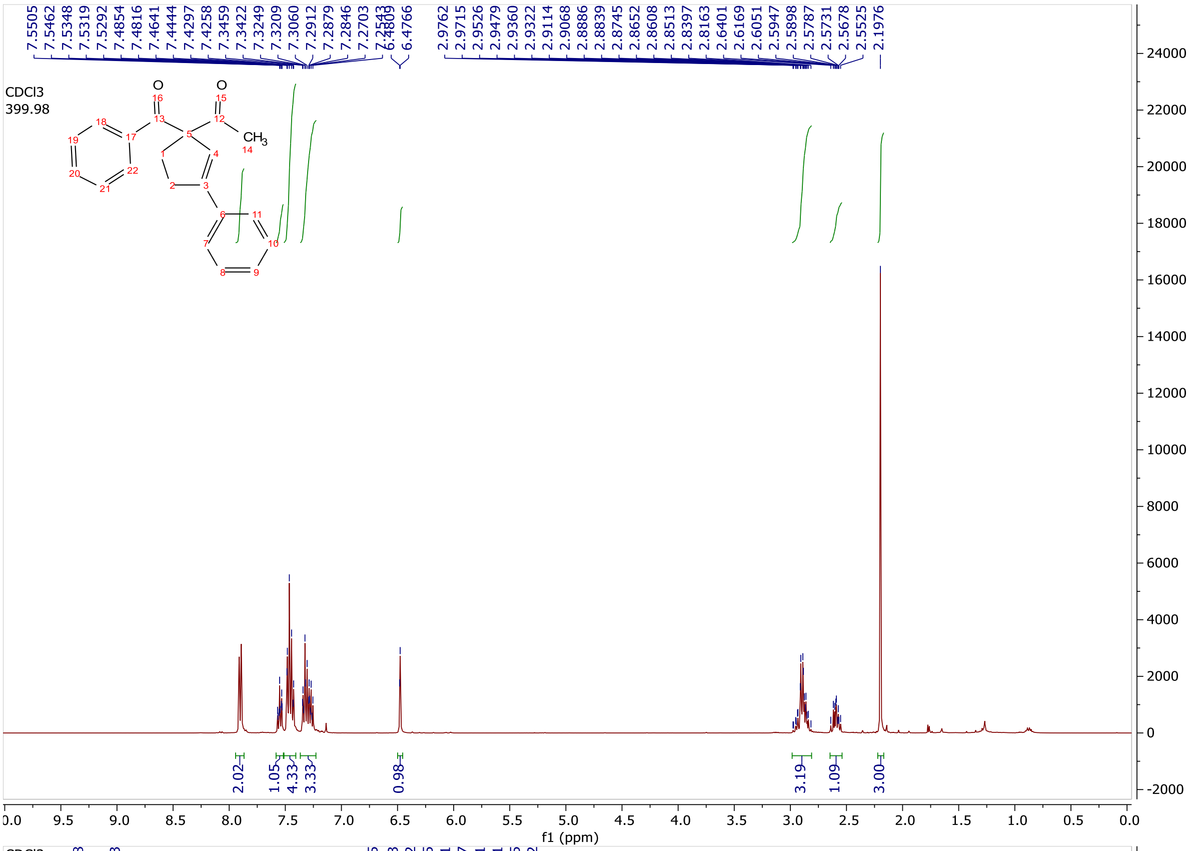

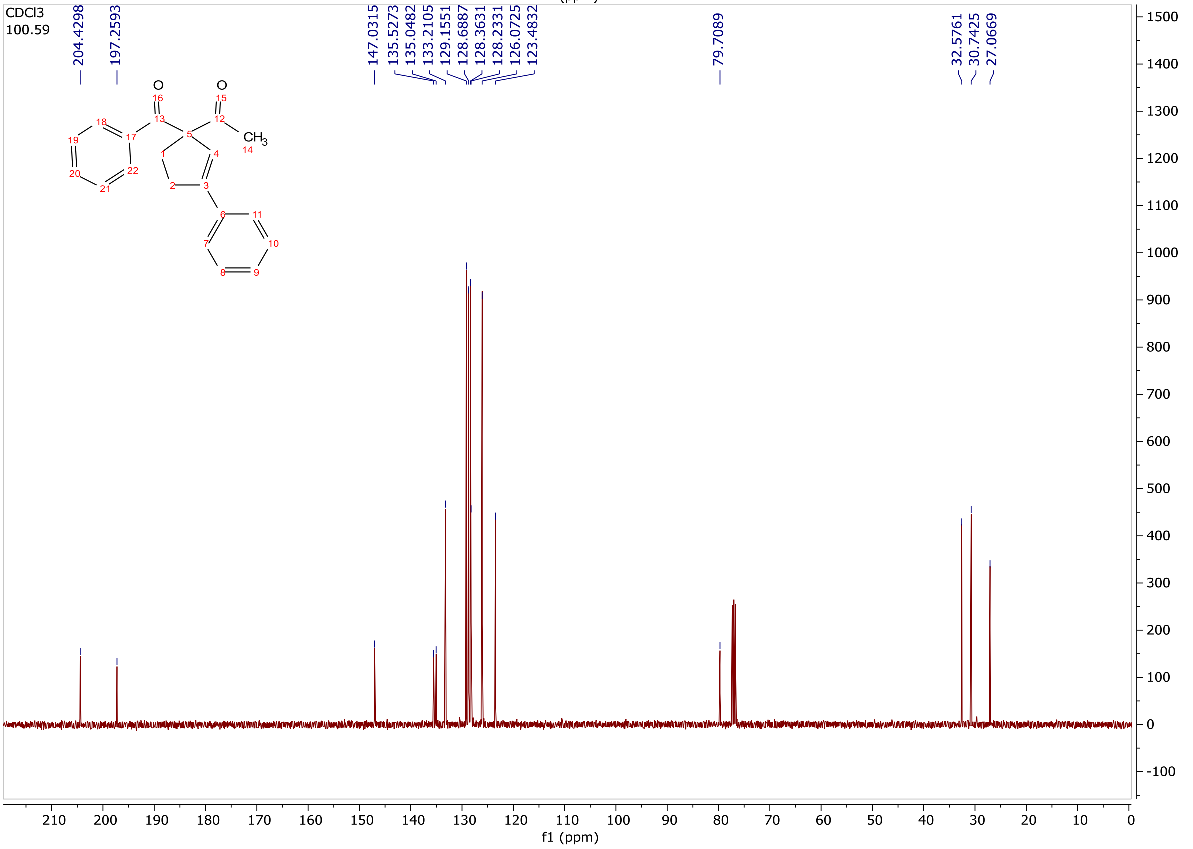

(35) dimethyl 3,3’-(1,4-phenylene)bis(1-acetylcyclopent-2-ene-1-carboxylate)

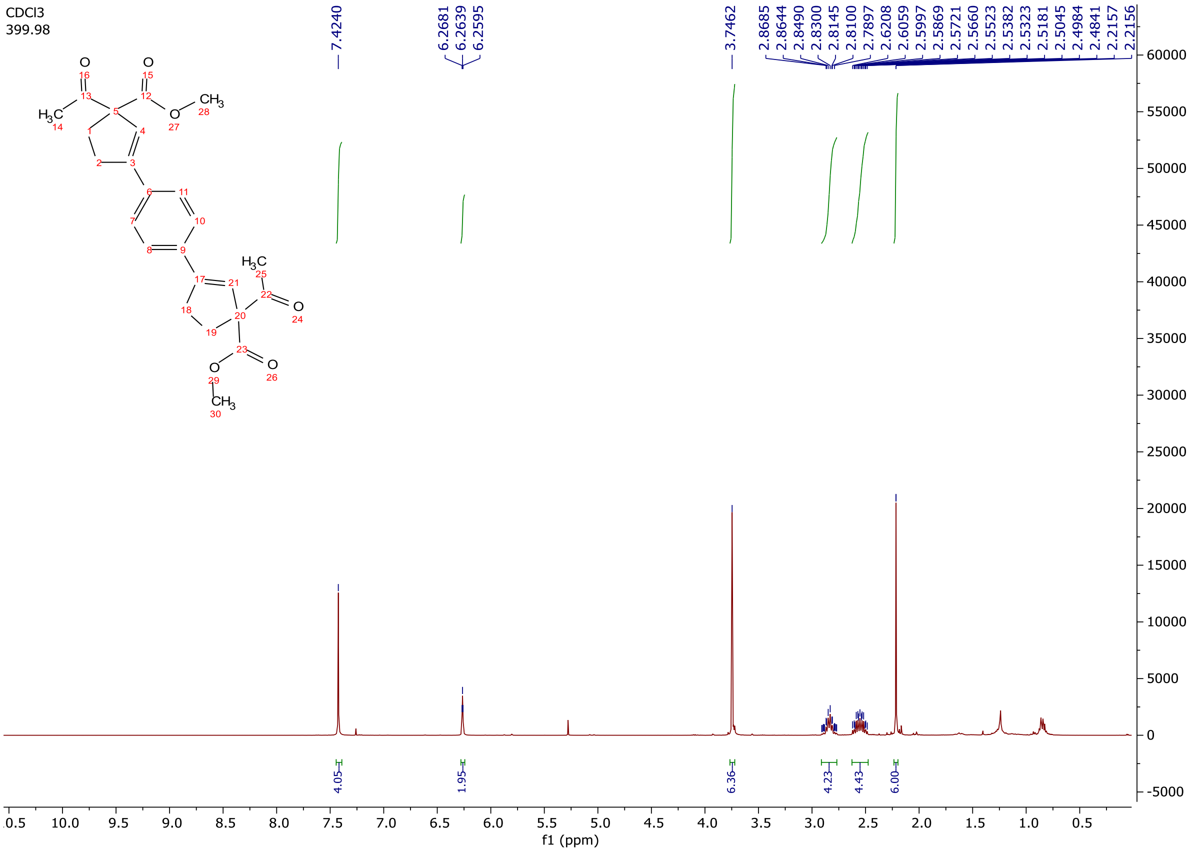

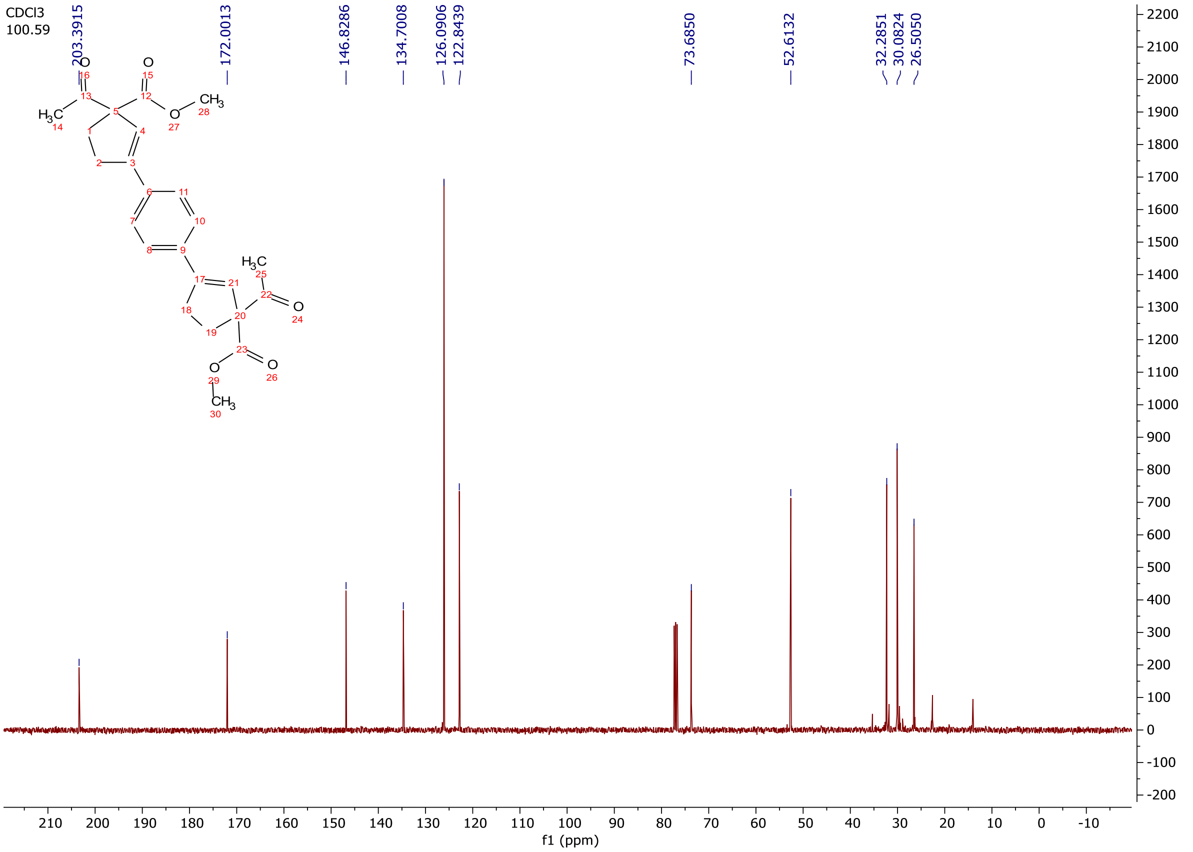

(36) methyl 1-acetyl-3-(cyclohex-1-en-1-yl)cyclopent-2-ene-1-carboxylate

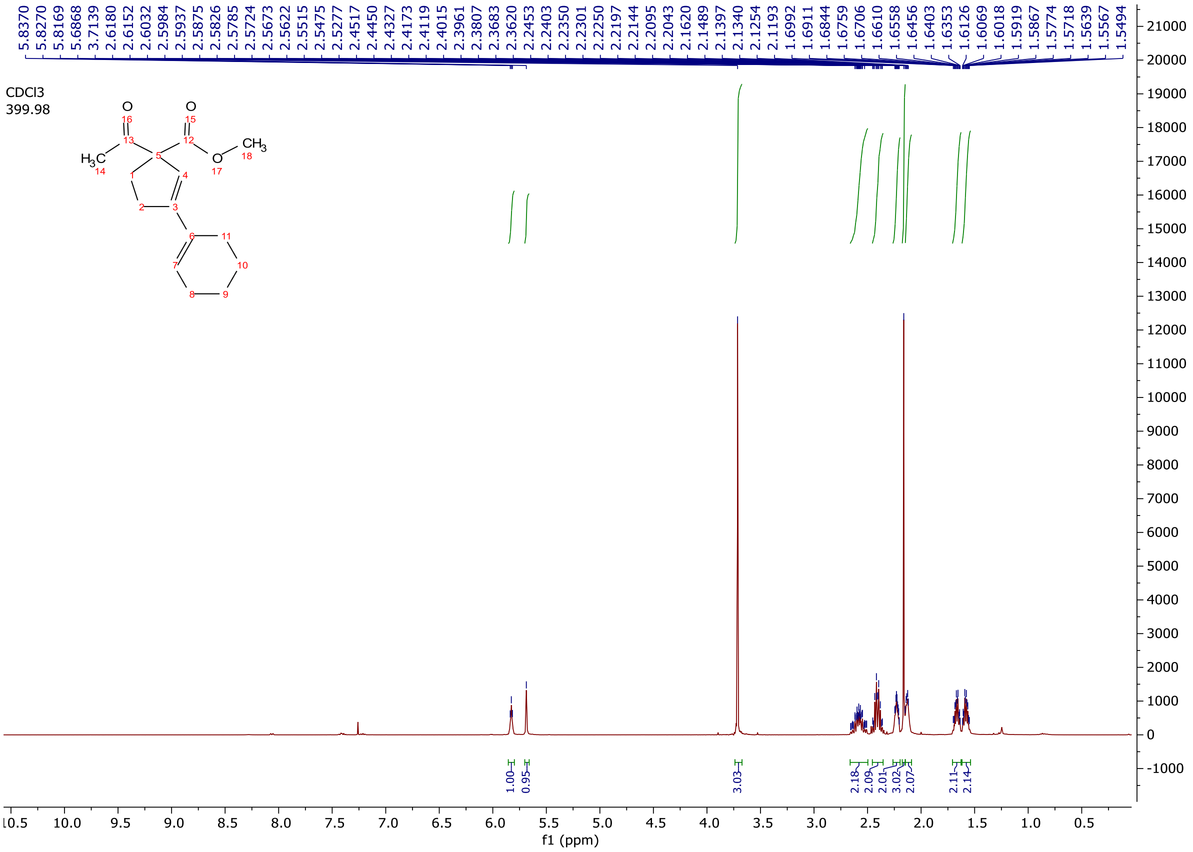

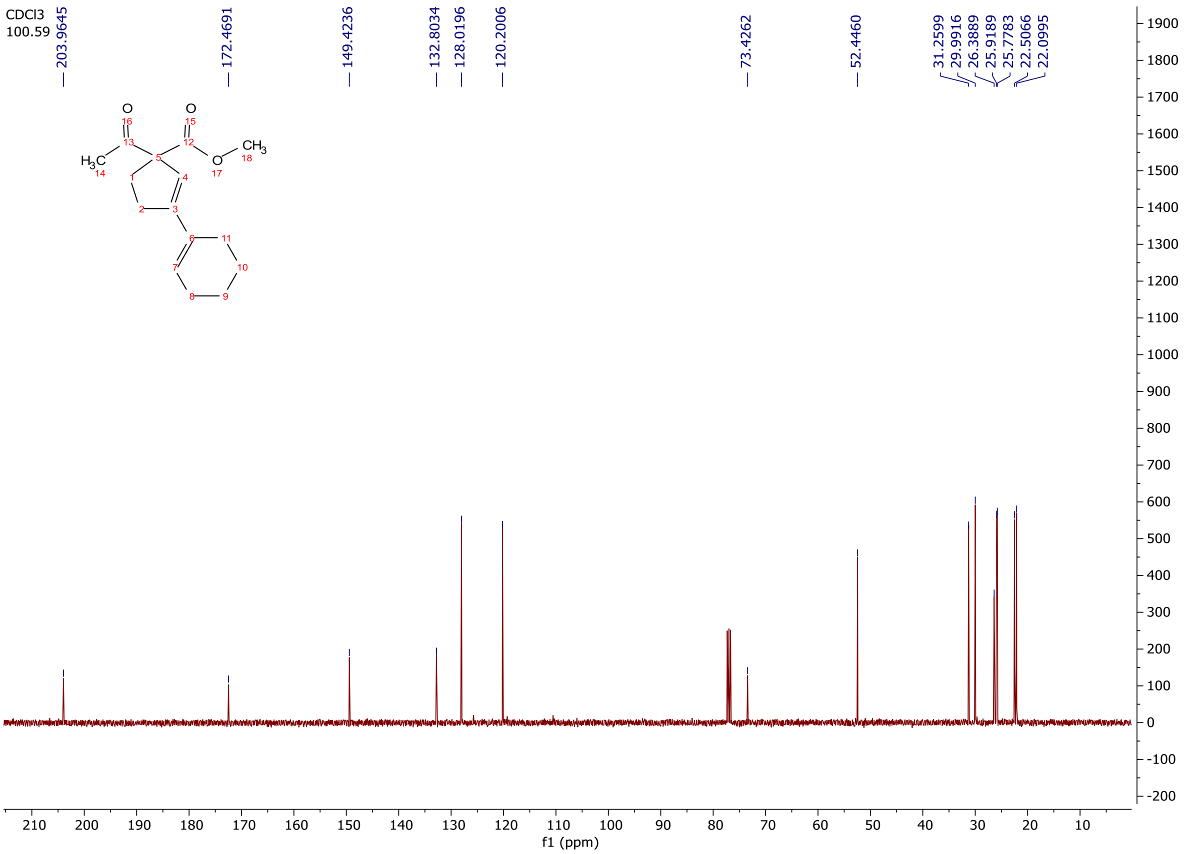

(37) methyl 3-acetyl-[1,1'-bi(cyclopentane)]-1,1'-diene-3-carboxylate

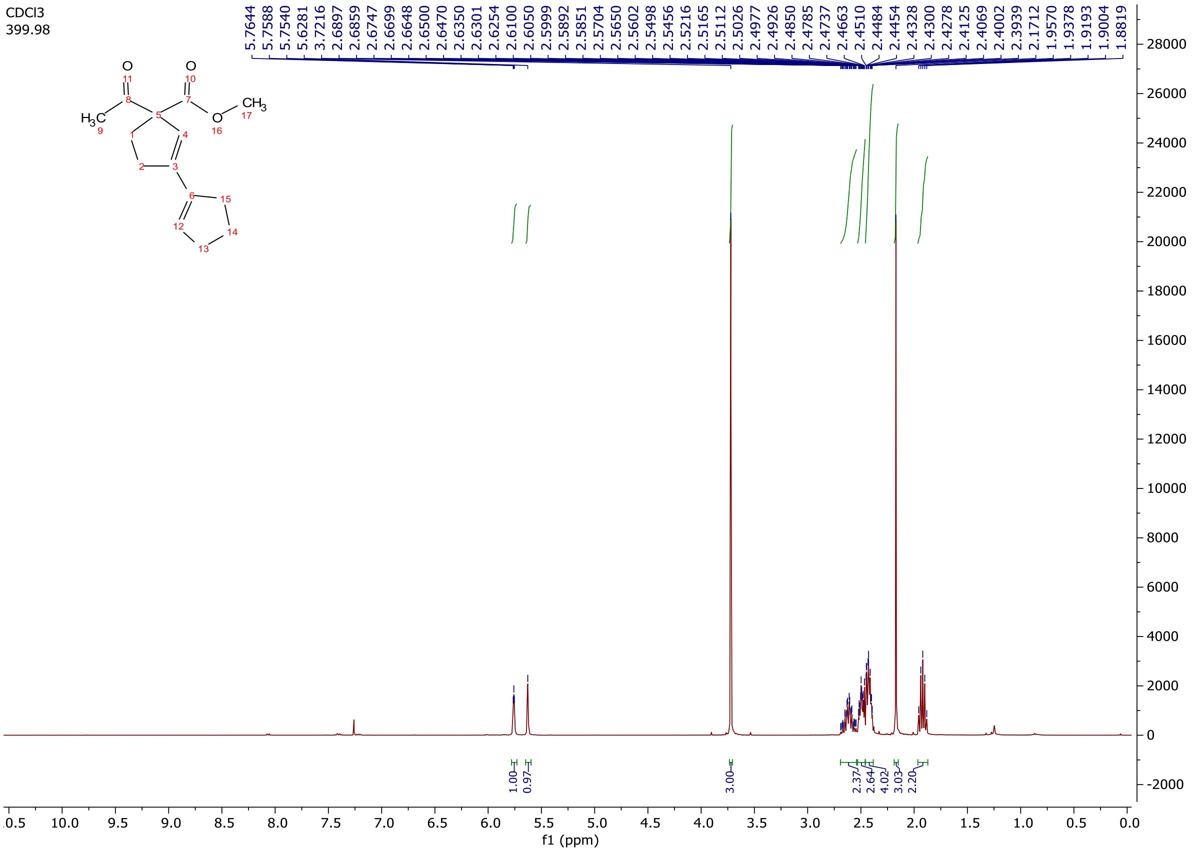

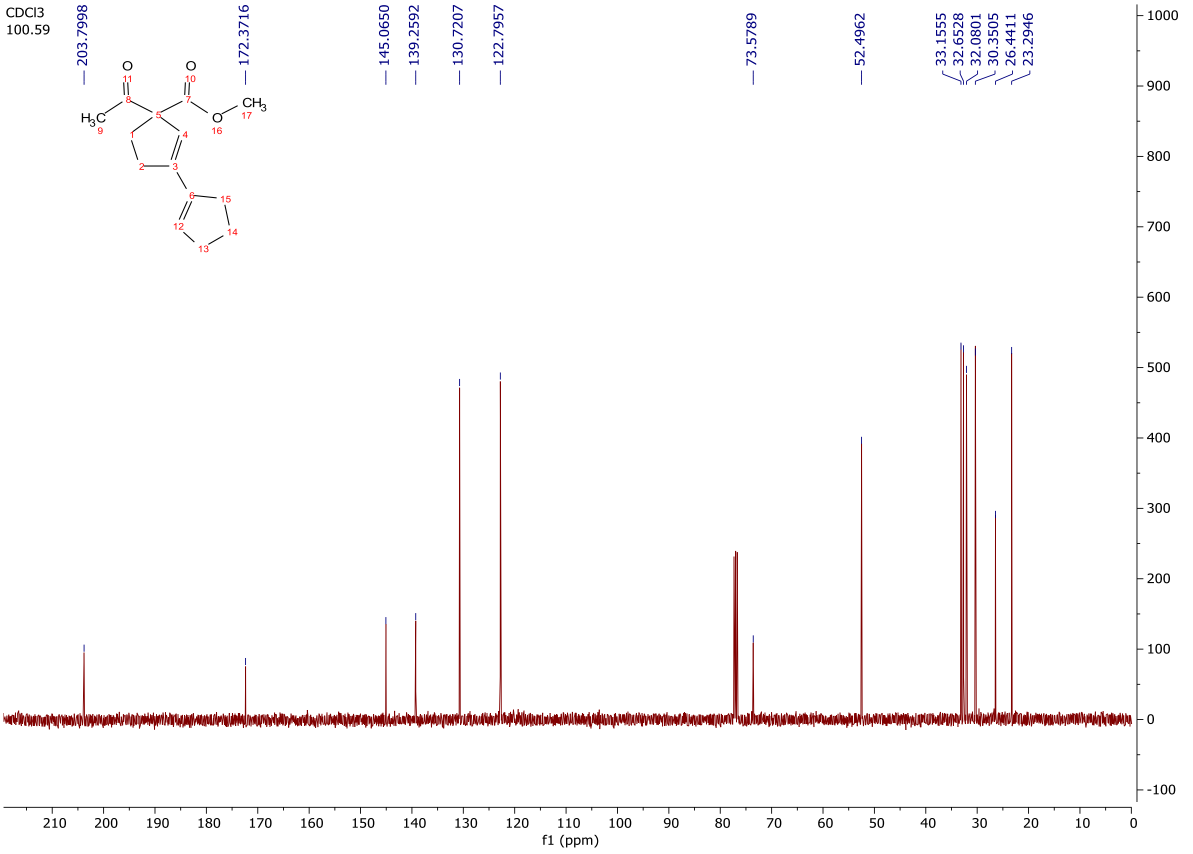

(38) methyl 1-acetyl-3-(2-oxo-2H-chromen-4-yl)cyclopent-2-ene-1-carboxylate

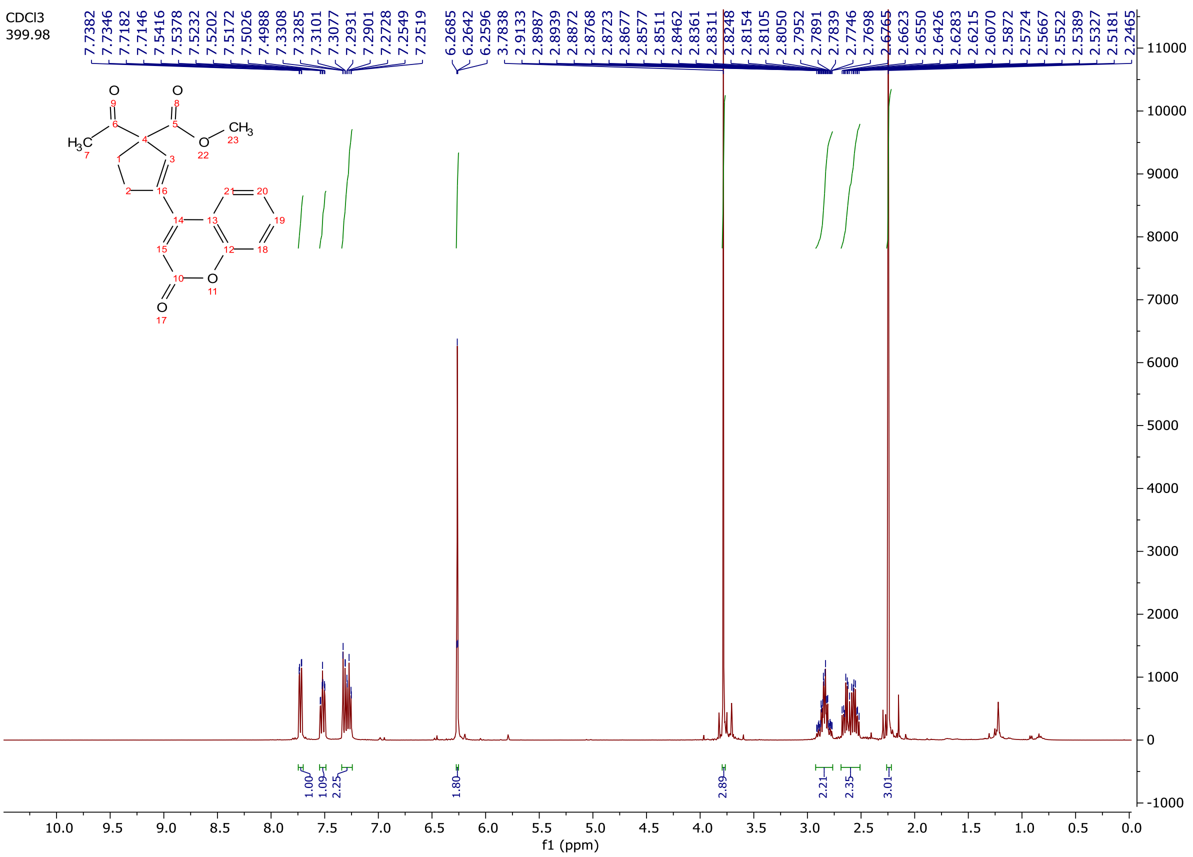

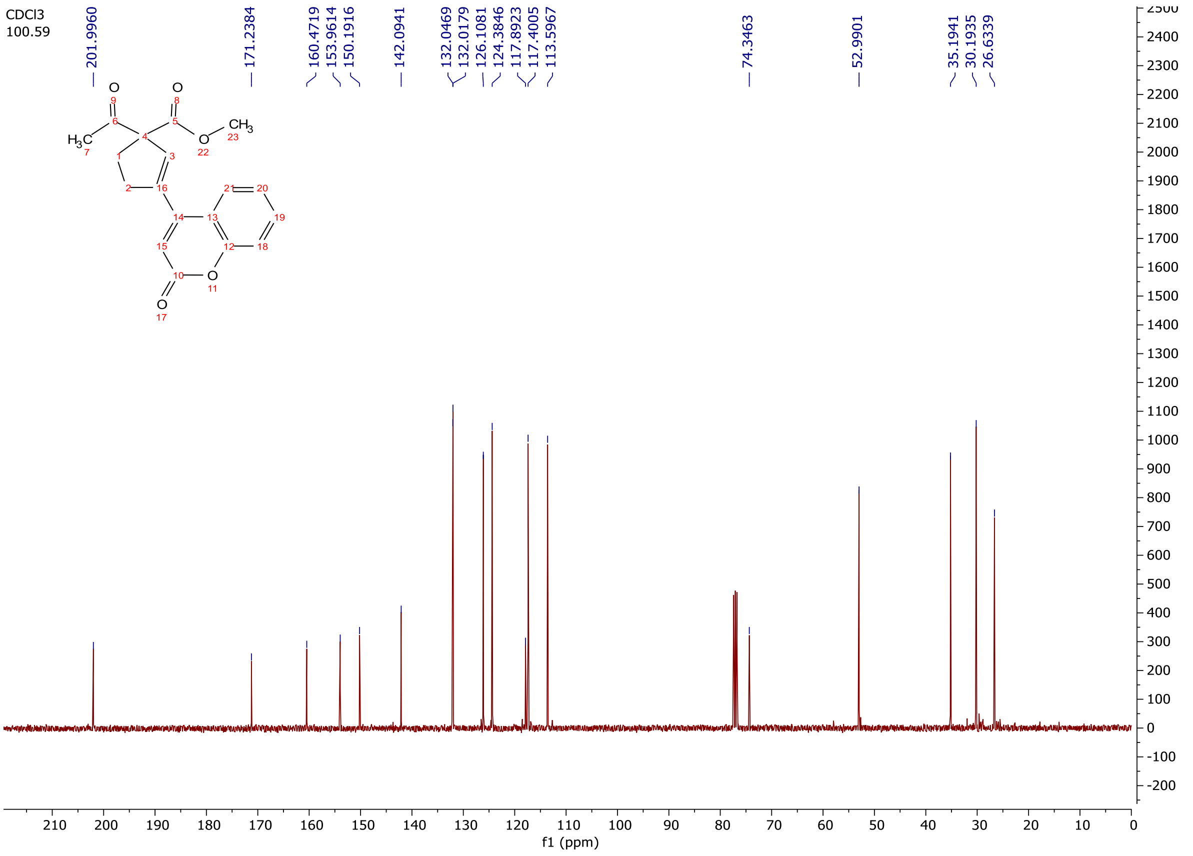

(39) methyl 1-acetyl-3-(4-allyl-2-methoxyphenyl)cyclopent-2-ene-1-carboxylate

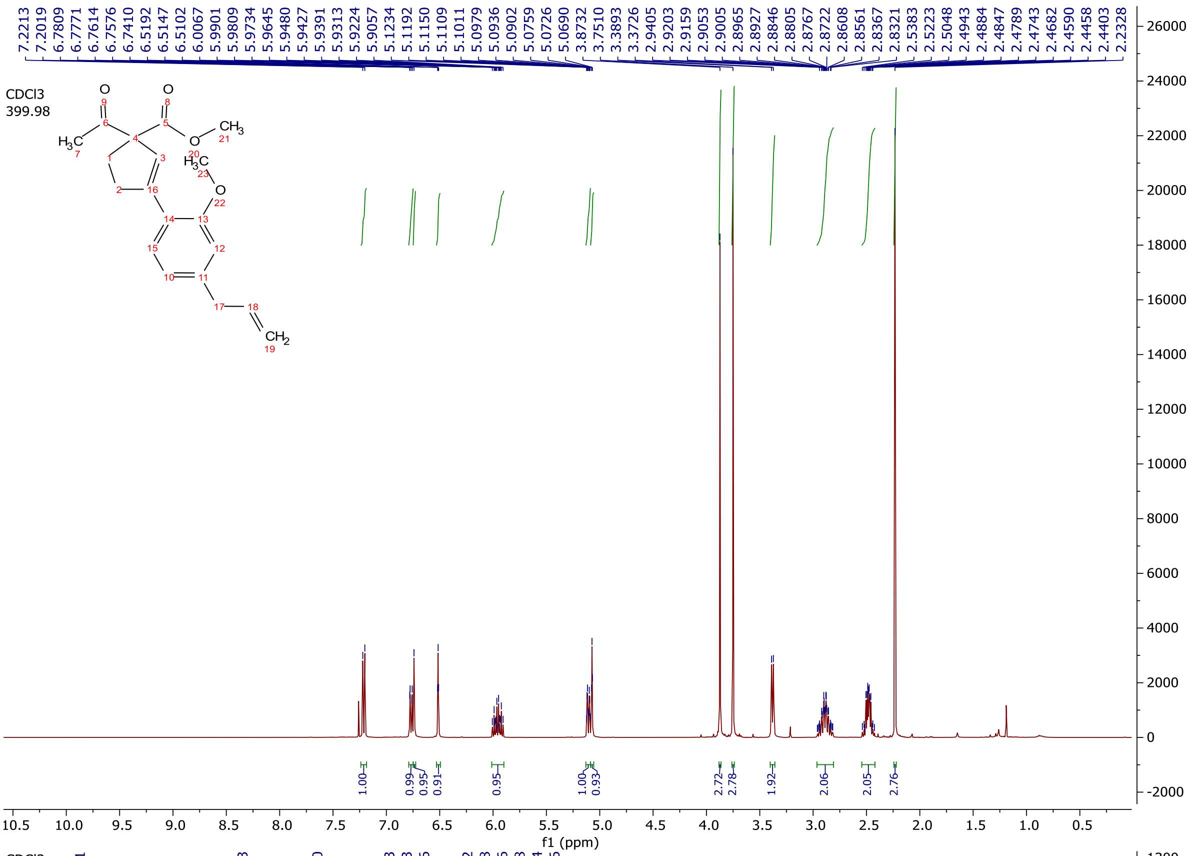

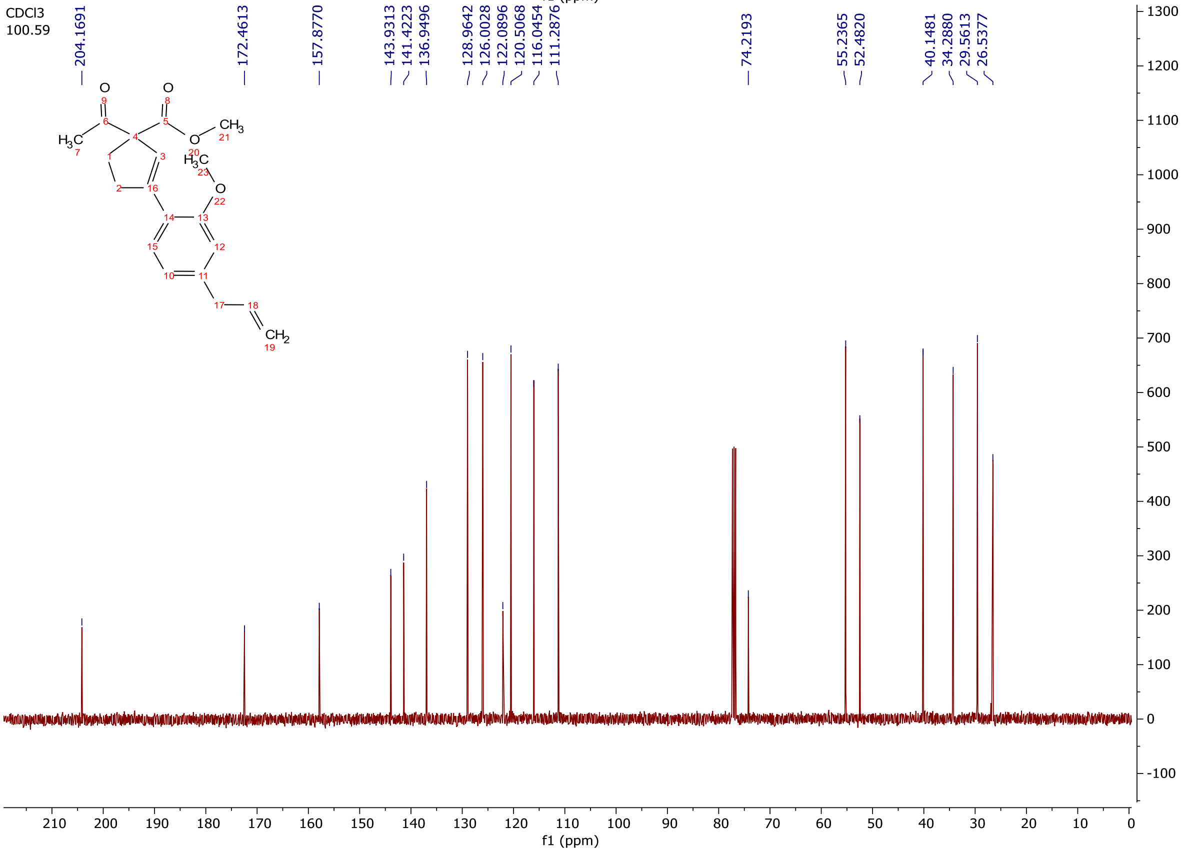

(40) methyl 1-acetyl-3-((8R,9S,13S,14S)-13-methyl-17-oxo-7,8,9,11,12,13,14,15,16,17-decahydro-6H-cyclopenta[a]phenanthren-3-yl)cyclopent-2-ene-1-carboxylate

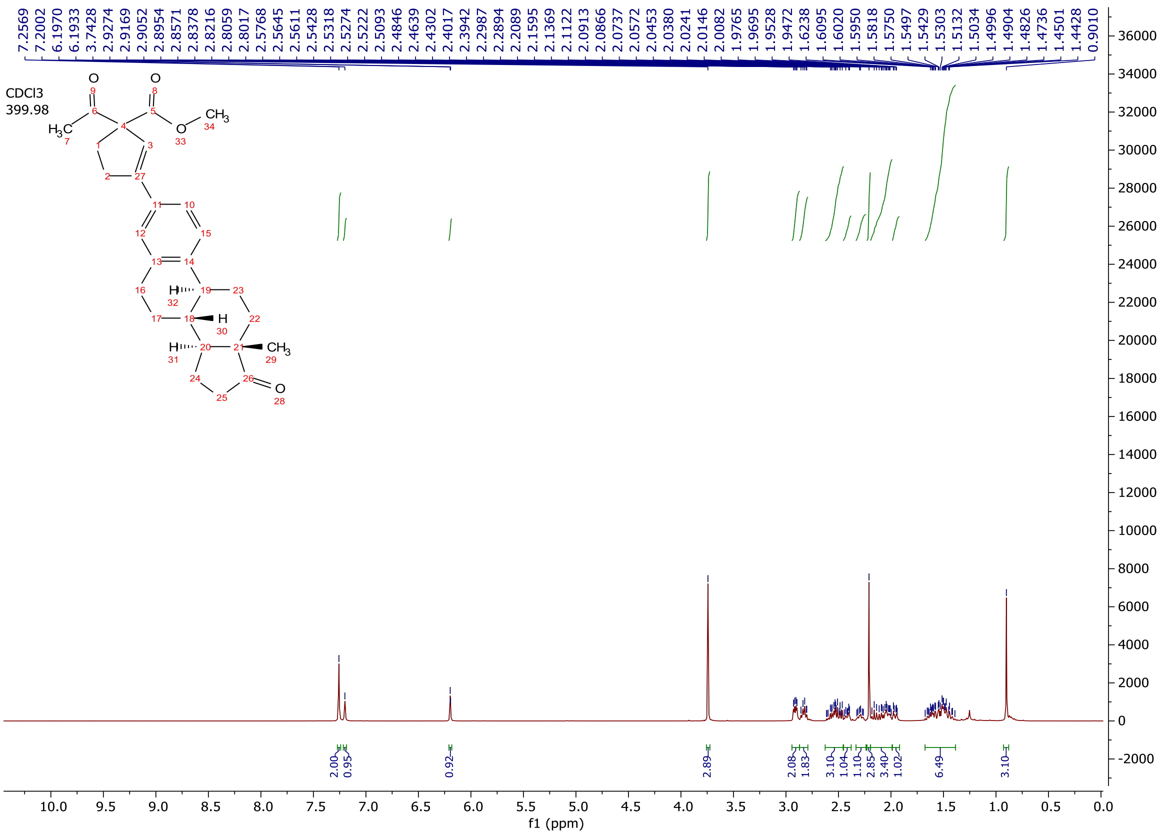

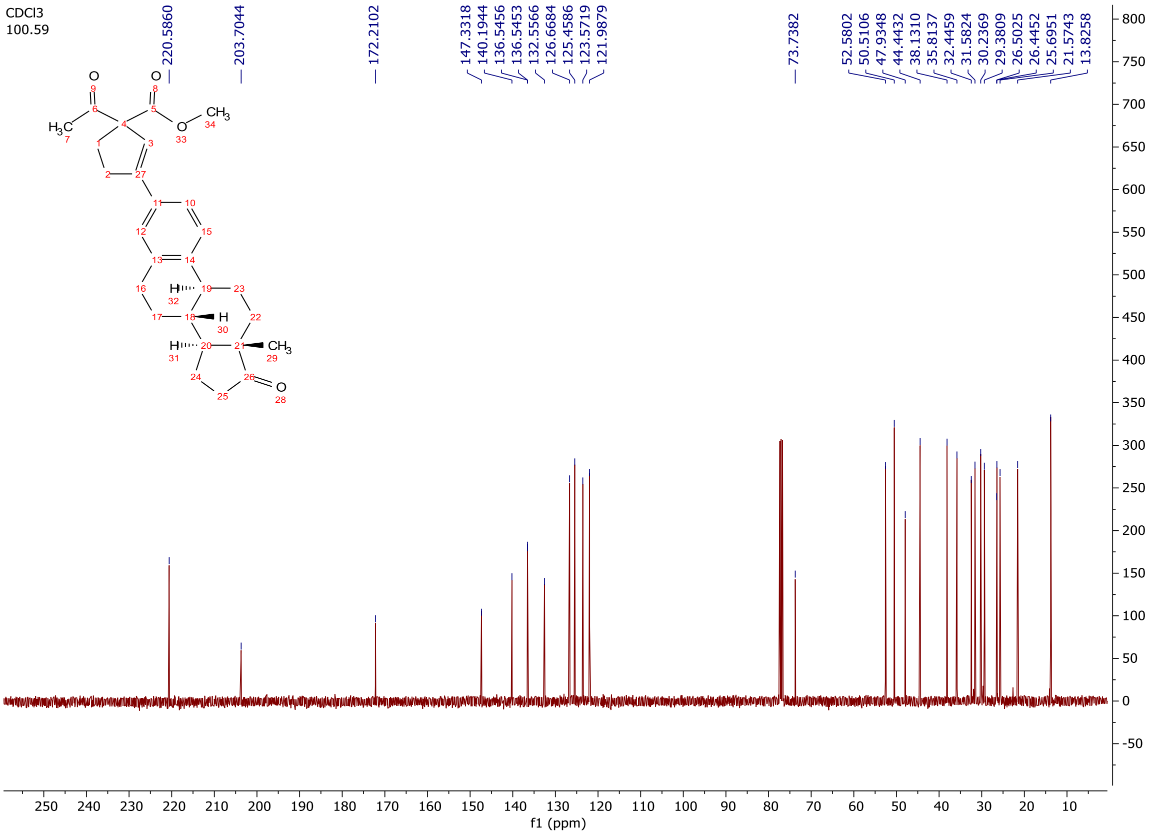

References

(1) Bruno, N. C.; Tudge, M. T.; Buchwald, S. L. Design and Preparation of New Palladium Precatalysts for C–C and C–N Cross-Coupling Reactions. *Chem. Sci.* **2013**, *4* (3), 916–920. https://doi.org/10.1039/C2SC20903A.

(2) Verboom, R. C.; Persson, B. A.; Bäckvall, J.-E. Palladium(II)-Catalyzed Intramolecular 1,4-Oxyacyloxylation of Conjugated Dienes. A Stereocontrolled Route to Fused Six-Membered Lactones and Pyrans. *J. Org. Chem.* **2004**, *69* (9), 3102–3111. https://doi.org/10.1021/jo0357667.

(3) Straathof, N. J. W.; Cramer, S. E.; Hessel, V.; Noël, T. Practical Photocatalytic Trifluoromethylation and Hydrotrifluoromethylation of Styrenes in Batch and Flow. *Angew. Chem. Int. Ed.* **2016**, *55* (50), 15549–15553. https://doi.org/10.1002/anie.201608297.

(4) Rizk, T.; Bilodeau, E. J.-F.; Beauchemin, A. M. Synthesis of Pyridines and Pyrazines Using an Intramolecular Hydroamination-Based Reaction Sequence. *Angew. Chem. Int. Ed.* **2009**, *48* (44), 8325–8327. https://doi.org/10.1002/anie.200903922.

(5) Kołodziejczyk, A.; Domański, S.; Chaładaj, W. Tandem Palladium-Catalyzed 6-Exo-Dig Oxocyclization Coupling of δ-Acetylenic β-Ketoesters with Aryl Bromides and Chlorides: Route to Substituted Dihydropyrans. *J. Org. Chem.* **2018**, *83* (20), 12887–12896. https://doi.org/10.1021/acs.joc.8b01832.

(6) Carney, R. L.; Johnson, W. S. Nonenzymic Biogenetic-like Olefinic Cyclizations. Cyclization of 1-Methyl-6-(Trans, Trans-7,11-Dimethyl-3,7,11-Dodecatrienyl)-2-Cyclohexen-1-Ol. *J. Am. Chem. Soc.* **1974**, *96* (8), 2549–2556. https://doi.org/10.1021/ja00815a038.

(7) Yu, B.; Selkti, M.; Ardisson, J.; Lannou, M.-I.; Sorin, G. Access to Conjugated Enynes via Allenyl Silver Formation/Cyclization/Decarboxylation Reaction Catalyzed by Silver Carbonate(I). *Org. Lett.* **2022**, *24* (31), 5721–5725. https://doi.org/10.1021/acs.orglett.2c02142.

(8) Frisch, M. J.; Trucks, G. W.; Schlegel, H. B.; Scuseria, G. E.; Robb, M. a.; Cheeseman, J. R.; Scalmani, G.; Barone, V.; Petersson, G. a.; Nakatsuji, H.; Li, X.; Caricato, M.; Marenich, a. V.; Bloino, J.; Janesko, B. G.; Gomperts, R.; Mennucci, B.; Hratchian, H. P.; Ortiz, J. V.; Izmaylov, a. F.; Sonnenberg, J. L.; Williams; Ding, F.; Lipparini, F.; Egidi, F.; Goings, J.; Peng, B.; Petrone, A.; Henderson, T.; Ranasinghe, D.; Zakrzewski, V. G.; Gao, J.; Rega, N.; Zheng, G.; Liang, W.; Hada, M.; Ehara, M.; Toyota, K.; Fukuda, R.; Hasegawa, J.; Ishida, M.; Nakajima, T.; Honda, Y.; Kitao, O.; Nakai, H.; Vreven, T.; Throssell, K.; Montgomery Jr., J. a.; Peralta, J. E.; Ogliaro, F.; Bearpark, M. J.; Heyd, J. J.; Brothers, E. N.; Kudin, K. N.; Staroverov, V. N.; Keith, T. a.; Kobayashi, R.; Normand, J.; Raghavachari, K.; Rendell, a. P.; Burant, J. C.; Iyengar, S. S.; Tomasi, J.; Cossi, M.; Millam, J. M.; Klene, M.; Adamo, C.; Cammi, R.; Ochterski, J. W.; Martin, R. L.; Morokuma, K.; Farkas, O.; Foresman, J. B.; Fox, D. J. G16_C01. 2016, p Gaussian 16, Revision C.01, Gaussian, Inc., Wallin.

(9) Grimme, S.; Antony, J.; Ehrlich, S.; Krieg, H. A Consistent and Accurate Ab Initio Parametrization of Density Functional Dispersion Correction (DFT-D) for the 94 Elements H-Pu. *J. Chem. Phys.* **2010**, *132* (15), 154104. https://doi.org/10.1063/1.3382344.

(10) Weigend, F.; Ahlrichs, R. Balanced Basis Sets of Split Valence, Triple Zeta Valence and Quadruple Zeta Valence Quality for H to Rn: Design and Assessment of Accuracy. *Phys. Chem. Chem. Phys.* **2005**, *7* (18), 3297–3305. https://doi.org/10.1039/B508541A.

(11) Marenich, A. V.; Cramer, C. J.; Truhlar, D. G. Universal Solvation Model Based on Solute Electron Density and on a Continuum Model of the Solvent Defined by the Bulk Dielectric Constant and Atomic Surface Tensions. *J. Phys. Chem. B* **2009**, *113* (18), 6378–6396. https://doi.org/10.1021/jp810292n.

(12) CYLview20; Legault, C. Y., Université de Sherbrooke, 2020 (Http://Www.Cylview.Org).
